# Synthesis and applications of highly functionalized 1-halo-3-substituted bicyclo[1.1.1]pentanes†
†Electronic supplementary information (ESI) available. CCDC 1825056–1825060. For ESI and crystallographic data in CIF or other electronic format see DOI: 10.1039/c8sc01355a


**DOI:** 10.1039/c8sc01355a

**Published:** 2018-05-21

**Authors:** Dimitri F. J. Caputo, Carlos Arroniz, Alexander B. Dürr, James J. Mousseau, Antonia F. Stepan, Steven J. Mansfield, Edward A. Anderson

**Affiliations:** a Chemistry Research Laboratory , 12 Mansfield Road , Oxford , OX1 3TA , UK . Email: edward.anderson@chem.ox.ac.uk; b Pfizer Worldwide Research and Development , Eastern Point Road, Groton , CT 06340 , USA; c Pfizer Worldwide Research and Development , 600 Main Street , Cambridge , MA 02139 , USA

## Abstract

Bicyclo[1.1.1]pentanes (BCPs) are important bioisosteres of 1,4-disubstituted arenes, *tert*-butyl and acetylenic groups that can impart physicochemical benefits on drug candidates. Here we describe the synthesis of BCPs bearing carbon and halogen substituents under exceptionally mild reaction conditions, *via* triethylborane-initiated atom-transfer radical addition ring-opening of tricyclo[1.1.1.0^1,3^]pentane (TCP) with alkyl halides. This chemistry displays broad substrate scope and functional group tolerance, enabling application to BCP analogues of biologically-relevant targets such as peptides, nucleosides, and pharmaceuticals. The BCP halide products can be converted to the parent phenyl/*tert*-butyl surrogates through triethylborane-promoted dehalogenation, or to other derivatives including carbonyls, alcohols, and heterocycles.

## Abstract 2

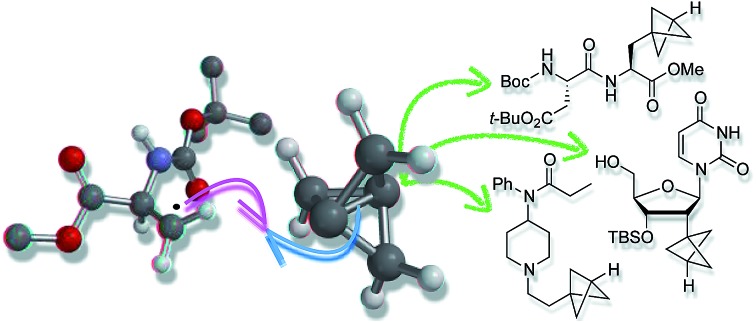
A wide range of halogenated bicyclo[1.1.1]pentanes are accessed by functional group tolerant radical ring-opening of tricyclo[1.1.1.0^1,3^]pentane, using triethylborane as initiator.

## Introduction

**Fig. 1:**
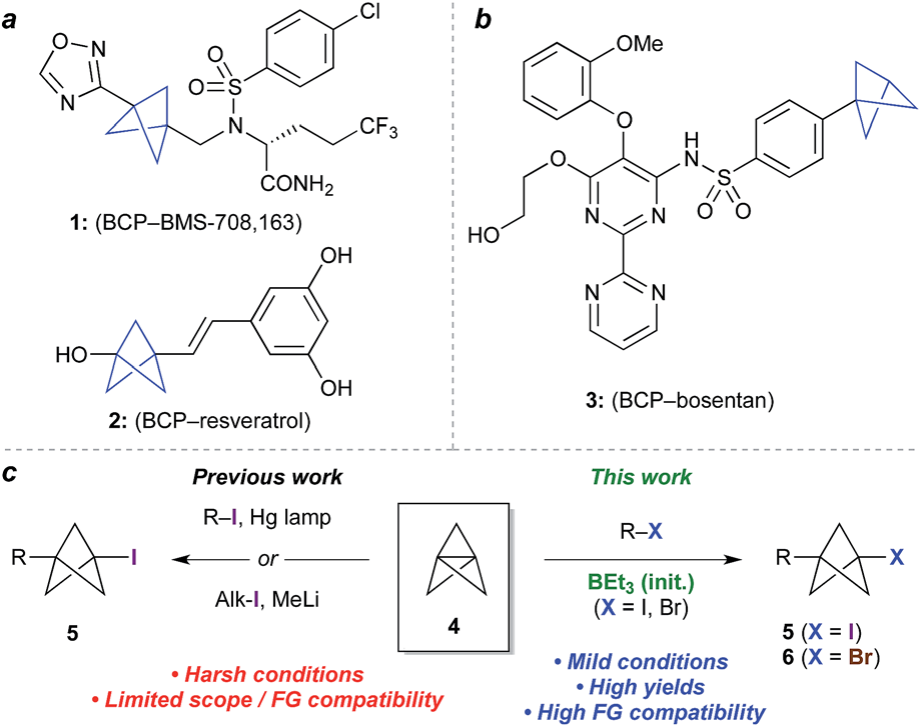
(a) Bicyclo[1.1.1]pentanes (BCPs) as 1,4-disubstituted arene bioisosteres. (b) BCP as a *tert*-butyl bioisostere. (c) Approaches to 1-halo-3-substituted BCPs in previous and current work.

Bioisosteres are important motifs in drug design,^1^ enabling the expansion of chemical and intellectual property space^2^ while also improving biological profiles relative to the parent functionality. Bicyclo[1.1.1]pentanes (BCPs) have shown particularly impressive results in this field as surrogates for 1,4-disubstituted arenes, *tert*-butyl, and alkyne groups, imparting desirable properties such as membrane permeability, solubility and metabolic stability.^3^,^4^ Examples include BCP analogues of the γ-secretase inhibitor BMS-708, 163 (**1**, Fig. 1a)^5^ and resveratrol (**2**),^6^ in which the BCP serves as a bioisostere for a *p*-substituted arene due to its comparable positioning of substituents; and of the pulmonary arterial hypertension agent bosentan (**3**, Fig. 1b), where a monosubstituted BCP replaces a *tert*-butyl group.^7^,^8^ Despite these favourable attributes, access to carbon-substituted BCPs remains a challenge. Their synthesis typically relies on multistep sequences from a small number of available precursors,^9^ or requires relatively harsh conditions,^10^ necessitating early-stage introduction of the BCP and limiting functional group tolerance.

The BCP system is most commonly accessed through ‘strain release’^11^ reactions of tricyclo[1.1.1.0^1,3^]pentane **4** (TCP),^12^ where in addition to classical approaches,^3^,^13^ recent work has seen the development of elegant methods for the synthesis of heteroatom-substituted BCPs.^11^,^14^ The insertion of TCP into C–X bonds would offer an appealing entry to carbon-substituted BCPs, as well as the opportunity for further functionalization of the halide product (**5**, Fig. 1c).^15^ To date however, this process has only been achieved using highly activated reagents (*e.g.* CF_3_I),^13^ or by mercury lamp irradiation of an alkyl or aryl halide,10d,e or methyllithium-promoted alkyl halide addition.10*b* Despite the importance of these contributions, all display limitations in substrate scope, functional group compatibility, or scalability, due to the constraints of the reaction conditions.

Atom transfer radical addition (ATRA) reactions using chemical initiators are an attractive alternative for TCP ring opening, but previously required a large excess of the radical precursor, or suffered from the formation of oligomeric ‘staffane’ byproducts due to multiple insertions into TCP.10d,^13^ Building on our studies of radical-mediated nucleoside alkynylation,^16^ we questioned whether triethylborane could serve as an effective initiator^17^ for the synthesis of 1-halo-3-substituted bicyclopentanes (Fig. 1c). Here we report the development of this method as an efficient and highly functional group tolerant route to halogenated BCPs, the utility of which are illustrated through various derivatizations. Importantly, the mild conditions of this chemistry allow the synthesis of bicyclopentanes that could not be accessed using other methods, and open up opportunities for the late-stage functionalization of more complex molecules.

## Results and discussion

We began our studies using ethyl iodoacetate **7a**, and were delighted to observe complete reaction in just 15 min at room temperature using 1.3 equivalents of TCP and 10 mol% triethylborane (1 M in hexane), with the product iodide **5a** isolated in 83% yield (Table 1, entry 1). A mild exotherm was observed on addition of triethylborane to the reaction, which could be avoided by reducing the temperature (entry 2). The amount of initiator could be decreased to 1 mol% without detriment (entries 2–5), and the quantity of TCP could also be lowered (to 1.1 equiv.), affording **5a** in 92% yield (entry 6). Successful reaction was similarly observed using TCP as a solution in dibutyl ether (0.19 M), which is significant for industrial applications (entry 7). The presence of triethylborane proved crucial, as reactions run in its absence failed to reach completion in the dark or light (entries 8 and 9).^18^ Importantly, no staffane byproducts were observed under the optimized conditions (entry 6).

**Table 1:** Optimization of triethylborane-promoted tricyclopentane ring-opening

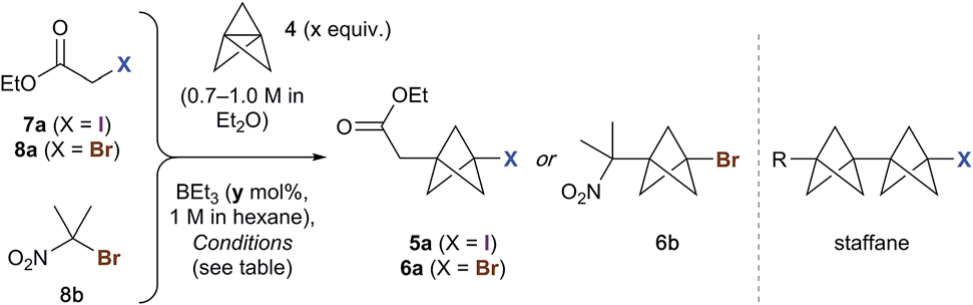						
Entry	Substrate	***x*** (equiv.)	***y*** (mol%)	*T*	*t*	Yield^*a*^ (%)
1	**7a**	1.3	10	rt	15 min	83
2	**7a**	1.3	10	0 °C	15 min	87
3	**7a**	1.3	5	0 °C	15 min	89
4	**7a**	1.3	1	0 °C	15 min	95
5	**7a**	1.3	0.5	0 °C	15 min	(67 : 33)
6	**7a**	1.1	1	0 °C	15 min	**92**
7	**7a**	2.0	10	rt	16 h	98^*b*^
8	**7a**	2.0	0	rt	20 h	(60 : 40)^*c*^
9	**7a**	2.0	0	rt	20 h	(33 : 67)
10	**8a**	1.3	10	rt	20 h	47^*d*^
11	**8b**	1.3	10	rt	15 min	67
12	**8b**	1.3	10	0 °C	15 min	**73**
13	**8b**	1.3	1	0 °C	15 min	(60 : 40)

^*a*^Isolated yields. Figures in parentheses indicate incomplete reactions, and the ratio of starting material to product as judged by ^1^H NMR spectroscopic analysis of the crude reaction mixture.

^*b*^Reaction carried out in Bu_2_O (0.19 M).

^*c*^Reaction carried out in the dark.

^*d*^Isolated as a 6 : 1 mixture of **6a** : staffane.

Use of the equivalent bromoacetate **8a** led to incomplete conversion, or significant staffane formation (entry 10).^19^ However, we were pleased to find that 2-bromo-2-nitropropane **8b** underwent rapid reaction with 1.3 equivalents of TCP to afford **6b** in 67% yield (entry 11).^20^ As with **7a**, full conversion was maintained on cooling the reaction to 0 °C (73%, entry 12); however, lowering the quantity of triethylborane resulted in incomplete reaction (entry 13). The success of substrate **8b** presumably reflects a more efficient bromine atom abstraction by the bicyclopentyl radical in the propagation step compared to ester **8a**, due to the greater stability of the tertiary nitro-substituted radical.

**Fig. 2:**
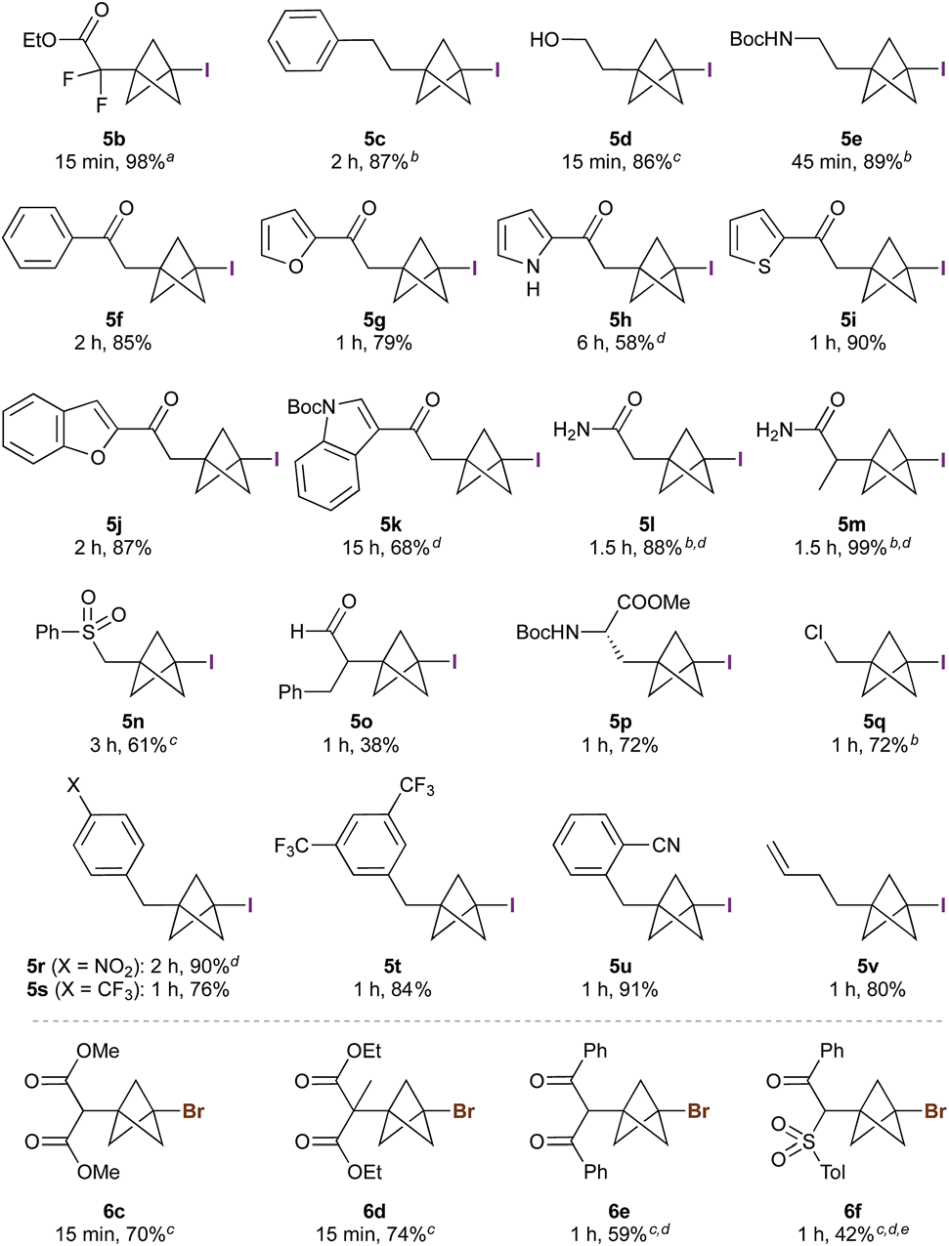
Synthesis of 1-iodo- and 1-bromo-3-substituted BCPs. All reactions performed using 2 equiv. tricyclo[1.1.1.0^1,3^]pentane (TCP) and 10 mol% BEt_3_ (1 M in hexane) at room temperature, unless indicated otherwise. ^*a*^1.1 equiv. TCP, 1 mol% BEt_3_, 0 °C. ^*b*^1.3 equiv. TCP, rt. ^*c*^1.3 equiv. TCP, 0 °C. ^*d*^Co-solvent added to solubilize the substrate: MeOH for **5m**, **n**; CH_2_Cl_2_ for **5h**, **k**, **r** and **6e**, **f**. ^*e*^5% staffane observed.

The scope of the triethylborane-initiated ATRA was next explored using organohalides featuring a variety of functional groups (Fig. 2).^21^ While the optimized conditions (Table 1, entry 6) translated smoothly to ethyl difluoroiodoacetate, giving bench-stable BCP **5b** in 98% yield,^22^ incomplete reactions were observed in other cases. To ensure consistency, the substrate screen was therefore conducted using 10 mol% BEt_3_ and 1.3–2.0 equivalents of TCP at either 0 °C or room temperature, according to the requirements of the substrate. Alkyl iodides showed excellent reactivity, tolerating groups such as hydroxyls and carbamates (**5c–e**). α-Iodoketones gave high yields across a range of substrates, including heteroaromatics (**5f–k**). Other electron-withdrawing groups such as amides and sulfones also proved viable (**5l–5n**),^20^ including the sensitive but potentially valuable aldehyde **5o**. The formation of **5p** is particularly notable due to the potential utility of BCP analogues of amino acids;^23^ no racemization was observed in this reaction. Even chloroiodomethane was found to be a suitable substrate, delivering chloromethyl BCP **5q** in good yield. Benzyl halides also underwent smooth reaction, providing electron-withdrawing substituents were present on the arene (which presumably accelerates the halide abstraction step from the intermediate BCP radical, **5r–5u**).^20^ The involvement of radical intermediates was supported by the use of (iodomethyl)cyclopropane, which exclusively gave the ring-opened product **5v**. Finally, other electron-deficient bromides proved suitable partners, affording products **6c–f** in short reaction times; for these substrates, the presence of two electron-withdrawing groups proved essential for efficient bromine atom abstraction.

**Scheme 1:**
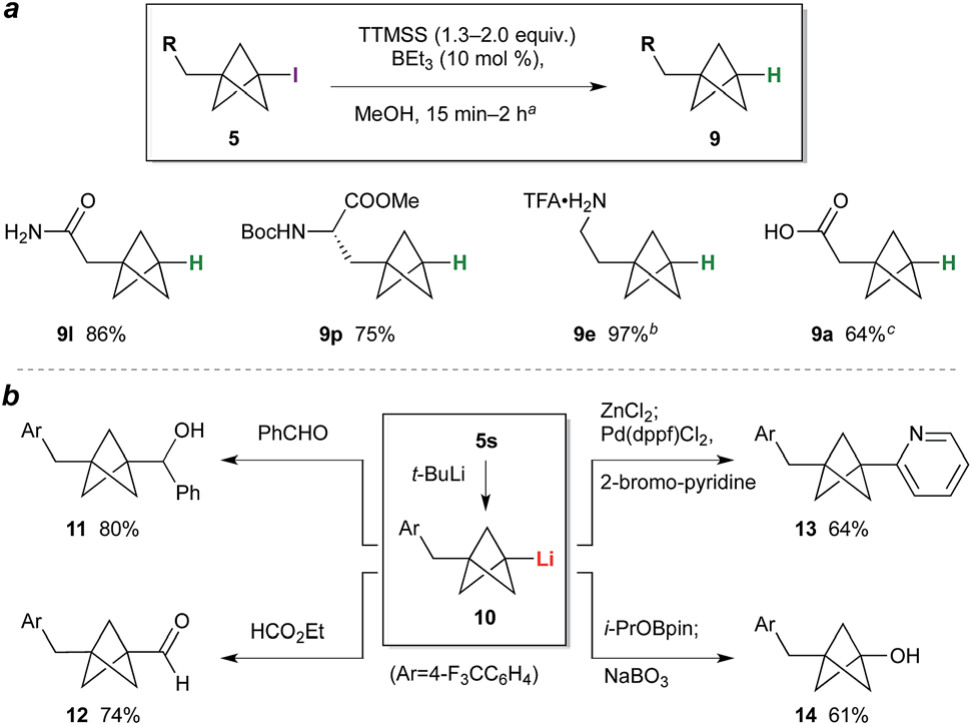
(a) BCP deiodination. ^*a*^See the ESI† for experimental conditions for each substrate. ^*b*^Deiodination product from **5e** treated with CF_3_CO_2_H (yield over two steps). ^*c*^Iodide **7a** processed through TCP ring opening, deiodination, and hydrolysis (NaOH, MeOH) without purification. (b) BCP iodide functionalization.

While the BCP iodide products are stable towards storage at –20 °C (particularly if crystalline), in some cases gradual coloration was observed at ambient temperature. Deiodination was expected to enhance BCP stability, and would simultaneously access the parent phenyl/*tert*-butyl bioisostere. We questioned whether triethylborane could again be used as initiator for this C–I bond reduction; pleasingly, the use of 1.3 equivalents of tris(trimethylsilyl)silane (TTMSS) as a non-toxic hydrogen atom source^24^ and 10 mol% BEt_3_ effected smooth deiodination of various BCP iodides (Scheme 1a), giving amide **9l**, a BCP analogue of phenylalanine (**9p**), and trifluoroacetate salt **9e**, the free base of the latter being a BCP equivalent of the monoaminergic neuromodulator phenethylamine. Given the use of the same initiator for both radical processes, a one-pot reaction sequence was also performed in which BCP **9a** was prepared directly from **7a** in a single operation (64%). Transformation of the iodide into substituents other than hydrogen was also explored (Scheme 1b): lithiation of **5s**,10*b* followed by reaction of the resulting organolithium **10** with benzaldehyde or ethyl formate, gave alcohol **11** and aldehyde **12** respectively. Alternatively, transmetalation to the organozinc and cross-coupling with 2-bromopyridine afforded pyridyl BCP **13** (64%).10a,b Pleasingly, the synthesis of a phenol bioisostere^6^ (**14**) could also be accomplished by a borylation quench, followed by oxidation with sodium perborate.

**Scheme 2:**
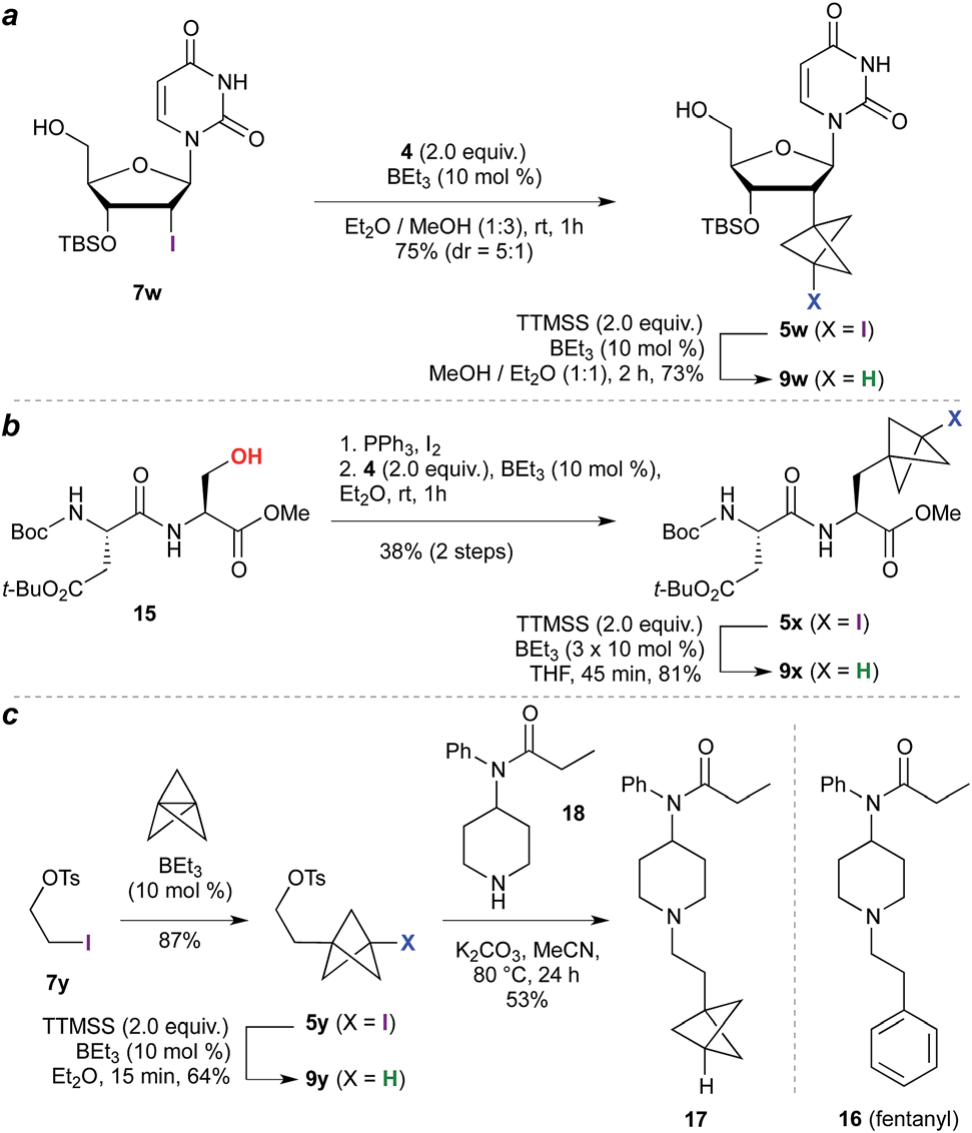
Synthesis of nucleoside, dipeptide, and pharmaceutical BCP analogues.

The high functional group tolerance and mild reaction conditions suggested that this methodology could be exploited in the functionalization of more complex organic molecules. For example, 2′-iodouridine derivative **7w** (Scheme 2a) underwent successful ATRA reaction to generate **5w** (75%, 5 : 1 dr), with deiodination delivering the 2′-deoxy-2′-BCP nucleoside **9w** (73%). More generally, the availability of iodides from hydroxyl groups provides a multitude of opportunities for bioisostere installation: Appel reaction of the aspartate–serine dipeptide **15** (Scheme 2b) afforded iodide **5x**, which on ATRA reaction and subsequent TTMSS reduction generated BCP-functionalized dipeptide **9x**. This sequence corresponds to a three step conversion of a serine residue to a potential phenylalanine equivalent, and here delivers a BCP analogue of aspartame. Finally, we targeted application of the methodology to the opioid receptor agonist fentanyl (**16**, Scheme 2c). Synthesis of its BCP analogue **17** was accomplished from iodotosylate **7y** where, pleasingly, the primary alkyl tosylate was tolerated in the initial high-yielding TCP ring-opening (87%). TTMSS-mediated reduction of the iodide product **5y** afforded the useful BCP building block **9y**, which underwent alkylation with norfentanyl **18** to afford fentanyl analogue **17** (53%).^20^

**Fig. 3:**
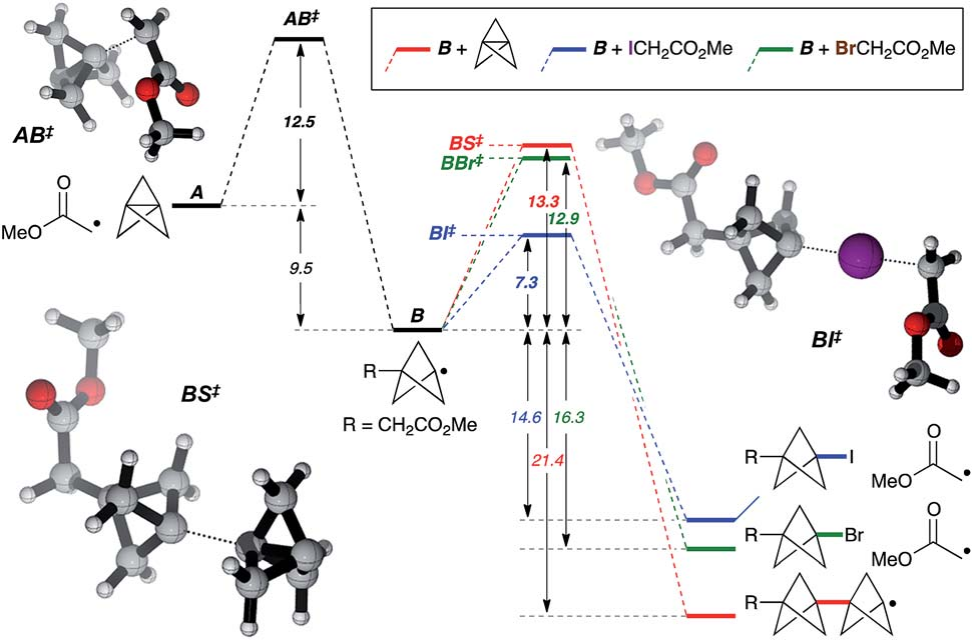
Theoretical analysis of the reaction pathway. Calculations carried out at the ROMO62x/def2tzvp level. Relative energies are in kcal mol^–1^. See the ESI for details.†

While the mechanism of this C–X addition reaction likely involves a radical pathway, not least due to the cyclopropane ring fragmentation observed in the formation of **5v** (Fig. 2), it is not immediately obvious why reaction propagation *via* halogen atom abstraction should proceed so efficiently, with avoidance of staffane formation. To explore this, we examined the reaction of α-iodo- and α-bromomethyl acetate with TCP from a theoretical perspective at the ROM062x/def2tzvp level^25^ (Fig. 3, *in vacuo*).^26^ Exergonic addition of the α-carbonyl radical to TCP (**A**) was found to proceed *via* transition state **AB^‡^** (Δ*G*^‡^ = 12.5 kcal mol^–1^). Reaction of the resultant bicyclopentyl radical **B** with α-iodo- or α-bromomethyl acetate (iodine/bromine atom transfer, **BI^‡^**/**BBr^‡^**), or with TCP (leading to a staffane radical, **BS^‡^**), was computed. Predicted activation barriers of 7.3 and 13.3 kcal mol^–1^ for iodine atom abstraction and TCP capture respectively suggest that the former productive propagation step is significantly favoured over oligomerization (ΔΔ*G*^‡^ = 6.1 kcal mol^–1^). In contrast, a barrier of 12.9 kcal mol^–1^ for bromine atom abstraction indeed suggests staffane formation to be competitive (ΔΔ*G*^‡^ = 0.4 kcal mol^–1^), which is consistent with experimental observations (Table 1), and reflects the influence of C–X bond strength in the propagation phase of the reaction.

## Conclusions

In conclusion, we have developed an efficient triethylborane-promoted radical-based method to synthesize 1-halo-3-substituted bicyclo[1.1.1]pentanes from readily available iodide and bromide starting materials. The reaction displays broad substrate scope, and functional group compatibility that is not achievable using other methods, enabling application to the functionalization of complex substrates such as nucleosides, peptides and drug-like molecules. The carbon–halogen bond is easily reduced under similarly mild conditions to reveal the parent bioisostere, or functionalized *via* methods such as Negishi cross-coupling or oxidation. In addition to offering a new entry to highly-functionalized bicyclopentanes, the C–X bond offers much potential for subsequent transformations; investigations to this end are underway.

## Conflicts of interest

There are no conflicts to declare.

## Supplementary Material

Supplementary information

Crystal structure data
