# Supplementary material for: Synthesis and applications of highly functionalized 1-halo-3-substituted bicyclo[1.1.1]pentanes
Source: Chem Sci. 2018 May 21;9(23):5295–300. doi: 10.1039/c8sc01355a (PMC6001403; doi:10.1039/c8sc01355a)

Electronic Supplementary Material (ESI) for Chemical Science.
This journal is © The Royal Society of Chemistry 2018

Synthesis and Applications of Highly Functionalized 1-Halo-3-Substituted Bicyclo[1.1.1]pentanes

Dimitri F. J. Caputo,[†] Carlos Arroniz,[†] Alexander B. Dürr,[†] James J. Mousseau,[§] Antonia F. Stepan,[¶] Steven J. Mansfield[†] and Edward A. Anderson*[†]

[†] Chemistry Research Laboratory, University of Oxford, 12 Mansfield Road, Oxford, OX1 3TA, U.K.

§ Pfizer Worldwide Research and Development, Eastern Point Road, Groton, CT 06340, United States

[¶] Pfizer Worldwide Research and Development, 600 Main Street, Cambridge, MA 02139, United States

Supporting Information

1. General Experimental Considerations 2

2. General Procedures 3

3. Optimization of triethylborane-promoted ATRA reactions 5

4. Synthesis and characterization of 1-halo-3-substituted BCPs 8

5. Substrate limitations 25

6. Reduction of 1-halo-3-substituted BCPs 26

7. Functionalization of 1-halo-3-substituted BCPs 29

8. Synthesis and characterization of nucleoside, dipeptide, and pharmaceutical BCP analogues 33

9. Synthesis and characterisation of substrates 40

10. Computational Details 62

11. X-ray Crystallography 81

12. References 85

13. NMR spectra 87

1. General Experimental Considerations

NMR Spectroscopy: Proton (^{1}H), carbon (^{13}C) and fluorine (^{19}F) NMR spectra were recorded on a Bruker AVIIIHD 400 nanobay (400 MHz) spectrometer. Proton, carbon and fluorine chemical shifts are quoted in ppm. ^{1}H NMR spectra were recorded using an internal deuterium lock for the residual protons in $CDCl_3$ (δ 7.26), CD_3OD (δ 3.31) and C_6D_6 (δ 7.16). ^{13}C NMR spectra were recorded using an internal deuterium lock in $CDCl_3$ (δ 77.0), CD_3OD (δ 49.0) and C_6D_6 (δ 128.1). Assignments were determined either on the basis of unambiguous chemical shift or coupling patterns, COSY, HMBC, HSQC and/or NOESY experiments. Peak multiplicities are defined as: s = singlet, d = doublet, t = triplet, q = quartet, m = multiplet, br. = broad; coupling constants (*J*) are reported to the nearest 0.1 Hz.

Infrared Spectroscopy: Infrared spectra were recorded on a Bruker Tensor 27 FT-IR spectrometer with the sample being prepared as a thin film on a diamond ATR module. Absorption maxima (ν_{max}) are quoted in wavenumbers (cm^{-1}).

Mass Spectroscopy: Low resolution mass spectra were recorded on a Micromass LCT Premier Open Access using electrospray ionisation (ESI). Accurate mass (HRMS) data was determined under conditions of ESI, EI and CI on a Bruker MicroTOF. High resolution values are calculated to 4 decimal places from the molecular formula, and all values are within a tolerance of 5 ppm.

Melting Points: Melting points were obtained using a Griffin melting point apparatus and are uncorrected.

X-ray crystallography: Details of instrumentation and techniques are reported in Section 10.

Reagents, solvents and techniques: All reagents were used directly as supplied. Solvents were either used as commercially supplied, or as purified by standard techniques. Anhydrous Et_2O was obtained from solvent dispenser units having been passed through an activated alumina column under argon. Unless otherwise stated, non-aqueous reactions were performed under air.

Reactions were monitored by thin layer chromatography on pre-coated aluminium-backed plates (Merck Kieselgel 60 with fluorescent indicator UV254). Spots were visualised by quenching of UV fluorescence or by staining with potassium permanganate or vanillin, and retention factors are reported with the solvent system in parentheses. Flash column chromatography was performed on silica gel obtained from Merck (Silica gel Si 60, 0.040-0.063 mm) under a positive pressure of nitrogen, using the stated solvent system.

2. General Procedures

General Procedure 1: Atom-transfer radical addition of halides to tricyclo[1.1.1.0^{1,3}]propane.

To a screw-capped vial containing the specified halide (1.0 equiv.) was added tricyclo[1.1.1.0^{1,3}]propane (1.1-2.0 equiv., solution in Et_2O). The vial was capped and the mixture was stirred at the indicated temperature for 3 min. BEt_3 (1-10 mol %, 1 M in hexane) was then added to the solution via syringe (needle tip in the solution), and the mixture was stirred as specified in the individual procedure. Once complete as judged by ^1H NMR spectroscopic analysis of an aliquot, the reaction mixture was concentrated and purified by column chromatography or recrystallization.

General Procedure 2. Finkelstein reaction.

Adapted from the procedure described by Zhou et al.[1] To a solution of the specified alkyl bromide (1.0 equiv.) in acetone was added sodium iodide (1.5 equiv.). The resulting mixture was stirred in the dark until TLC showed completion. The mixture was diluted with water and Et_2O and the layers were separated. The aqueous layer was extracted twice with Et_2O. The combined organic phases were washed with $Na_2S_2O_3$ (10 % *aq.*) and brine, dried ($MgSO_4$) and concentrated *in vacuo*. When specified, the residue was purified by column chromatography or recrystallization.

General Procedure 3. Appel reaction.

To a solution of the specified alcohol in CH_2Cl_2 was added triphenylphosphine and, if specified, imidazole. Iodine was added portionwise and the reaction mixture was stirred under an inert atmosphere at rt (quantities and reaction time specified in each procedure). The mixture was then diluted with water and CH_2Cl_2, and the aqueous phase was extracted twice with CH_2Cl_2. The combined organics were washed with $Na_2S_2O_3$ (10% *aq.*) and brine, dried ($MgSO_4$) and concentrated *in vacuo*. The residue was purified by column chromatography.

General Procedure 4. Copper oxide mediated iodination

According to the procedure described by Yin et al.[2] To a solution of the specified methylketone (1.0 equiv.) in MeOH were added CuO (1.0 equiv.) and I_2 (1.0 equiv.). The reaction mixture was stirred for 5 min, then refluxed for 2-3 h, and then concentrated *in vacuo*. The residue was taken up in EtOAc, filtered, and the organic filtrate washed with $Na_2S_2O_3$

(10% aq.,), dried ($MgSO_4$) and concentrated *in vacuo*. The residue was purified by column chromatography.

General comments: Halide substrates could generally be prepared in one step from commercial starting materials, via iodination or bromination of the corresponding alcohols, or reaction with enolates or their derivatives. Since many of these substrates are already known in the literature, their synthesis and characterisation are presented at the end of this manuscript.

Tricyclo[1.1.1.0^{1,3}]pentane (TCP), 4

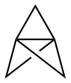

According to the procedure described by Gianatassio et al.[3] To a flame-dried round-bottom flask equipped with a stirrer bar was added 1,1-dibromo-2,2-bis(chloromethyl)cyclopropane (5.0 g, 16.9 mmol, 1.0 equiv.). The reaction vessel was evacuated and back-filled with argon three times, and then anhydrous Et_2O (10 mL) was added. The reaction vessel was cooled to −45 °C (dry ice / isopropanol bath). Phenyllithium (17.8 mL, 1.9 M in Bu_2O, 33.7 mmol, 2.0 equiv.) was added dropwise over 15 min at –45 °C, and the resulting mixture was stirred for 15 min at −45 °C. The cooling bath was replaced with an ice bath, and the reaction mixture was warmed to to 0 °C, and then stirred at this temperature for 2 h. The mixture was then distilled at room temperature (10 mbar) using a rotary evaporator, the receiving flask of which was immersed in a dry ice/acetone bath. The TCP-containing distillate (10 mL, TCP concentration 0.91 M in Et_2O, 54%) was transferred in a flame-dried septum-sealed bottle under inert atmosphere, and stored at −20 °C. The yield was determined by ^1H NMR spectroscopy with 1,2-dichloroethane as an internal standard. The concentration of the TCP solution ranged between 0.62 M and 1.10 M, with yields of 45-61%.

3. Optimization of triethylborane-promoted ATRA reactions.

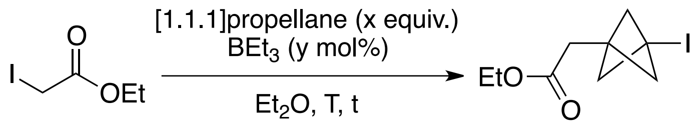

7a **5a**

Entry	x (equiv.)	y (mol %)	T	t	Yield (SM / P)
1	2.0	10	rt	20h	90%[a]
2	2.0	10	rt	15 min	n.d. (0 : 100)[a]
3	1.3	10	rt	15 min	(17 : 83)
4	1.3	10	0 °C	15 min	**87%**
5	1.3	5	0 °C	15 min	**89%**
6	1.3	1	0 °C	15 min	**95%**
7	1.3	0.5	0 °C	15 min	(66 : 33)
8	1.2	1	0 °C	15 min	**94%**
9	1.1	1	0 °C	15 min	**92%**
10	2.0	0	rt	20 h	(61:39)[a]
11	2.0	0	rt	20 h	(36 : 64)

a. The reaction was carried out in the dark

Table S1: Optimization of ethyl iodoacetate ATRA

Ethyl 2-(3-iodobicyclo[1.1.1]pentan-1-yl)acetate, 5a

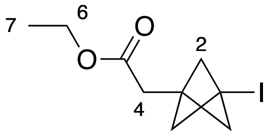

Ethyl iodoacetate (59 μL, 0.50 mmol, 1.0 equiv.), TCP (0.61 mL, 0.903 M in Et_2O, 0.55 mmol, 1.1 equiv.) and BEt_3 (50 μL, 50 μmol, 10 mol %) were submitted to **General Procedure 1** at room temperature for 15 min at 0 °C. Purification by column chromatography (SiO_2, pentane / Et_2O, 95:5) afforded **5a** (129 mg, 0.46 mmol, 92%) as a yellow oil.

$\mathbf{R_f}$ = 0.18 (pentane / Et_2O, 95 :5)

^{1}H NMR (400 MHz, $CDCl_3$) δ 4.12 (2H, q, J = 7.1 Hz, H6), 2.53 (2H, s, H4), 2.31 (6H, s, H2), 1.25 (3H, t, J = 7.1 Hz, H7).

^{13}C NMR (101 MHz, $CDCl_3$) δ 170.3, 60.9, 60.6, 43.6, 37.4, 14.3, 6.1.

HRMS (CI^+) Found $[M+H]^+$ = 281.0033; $C_9H_{14}IO_2$ requires 281.0033.

IR (film) ν_{max}/cm^{-1} 2979, 1732, 1369, 1280, 1175, 843.

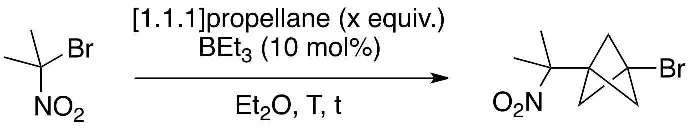

8b **6b**

Entry	x (equiv.)	T	t	Yield (SM / P)
1	2.0	rt	20h	**68%**[a]
2	2.0	rt	5 min	(0 : 100)[a]
3	1.1	rt	5 min	(8 : 92)[a]
4	1.1	rt	15 min	(11 : 89)[a]
5	1.5	rt	15 min	(0 : 100)[a]
6	1.4	rt	15 min	(0 : 100)[a]
7	1.3	rt	15 min	(3 : 97)[a]
8	1.3	rt	15 min	(3 : 97)
9	1.3	0°C	15 min	**73%**
10	1.2	0°C	15 min	(1 : 99)

a. The reaction was carried out in the dark

Table S2: Optimization of 2-bromo-2-nitropropane ATRA

1-bromo-3-(2-nitropropan-2-yl)bicyclo[1.1.1]pentane, 6b

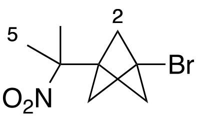

2-bromo-2-nitropropane (53 μL, 0.50 mmol, 1.0 equiv.), TCP (0.70 mL, 0.93 M in Et_2O, 0.65 mmol, 1.3 equiv.) and BEt_3 (50 μL, 1 M in hexane, 50 μmol, 10 mol %) were submitted to **General Procedure 4** at 0 °C for 15 min. Recrystallization from hot petroleum ether afforded **6b** (86 mg, 0.37 mmol, 73%) as a white needles. These crystals were suitable for X-ray diffraction (see Section 10).

m.p. = 79-80°C

R_f = 0.25 (petroleum ether / Et_2O, 9:1)

^{1}H NMR (400 MHz, $CDCl_3$) δ 2.22 (6H, s, H2), 1.56 (6H, s, H5).

^{13}C NMR (101 MHz, $CDCl_3$) δ 86.7, 56.4, 46.0, 34.8, 23.4.

HRMS (ESI$^+$, CI$^+$, EI$^+$) Not found.

IR (film) ν_{max}/cm^{-1} 3005, 1549, 1371, 1356, 1200, 861.

The structure of **6b** was unambiguously confirmed by X-ray crystallography.

Note on oxygen content of the reaction mixture:

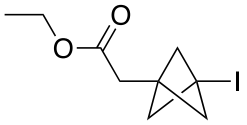

5a

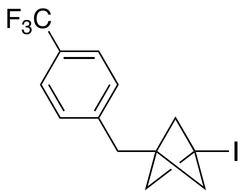

5s

Reaction of **7a** under N_2 atmosphere, otherwise according to Table S1 Entry 6, proceeded to completion in 15 min. Repetition of this reaction using a freeze-thaw degassed solution of tricyclo[1.1.1.0^{1,3}]pentane also led to complete conversion in 15 min. Although surprising from a reaction initiation perspective, we hypothesize that this reaction is, simply, extremely efficient in the propagation phase.

In contrast, reaction of **7s** (see below) under an N_2 atmosphere, otherwise according to the conditions in Figure S1 (see below), showed a significant retardation compared to the reaction under air (1 h, 76%), instead requiring a 20 h reaction time to reach completion. An equivalent yield of **7s** was obtained. Repetition of this reaction using a freeze-thaw degassed solution of tricyclo[1.1.1.0^{1,3}]pentane proceeded at an equivalent, slower rate – completion again being reached after 20 h reaction time.

We believe that this latter result is indeed supportive of a reaction initiation process requiring oxygen, i.e. reaction of O_2 with BEt_3.

4. Synthesis and characterization of 1-halo-3-substituted BCPs

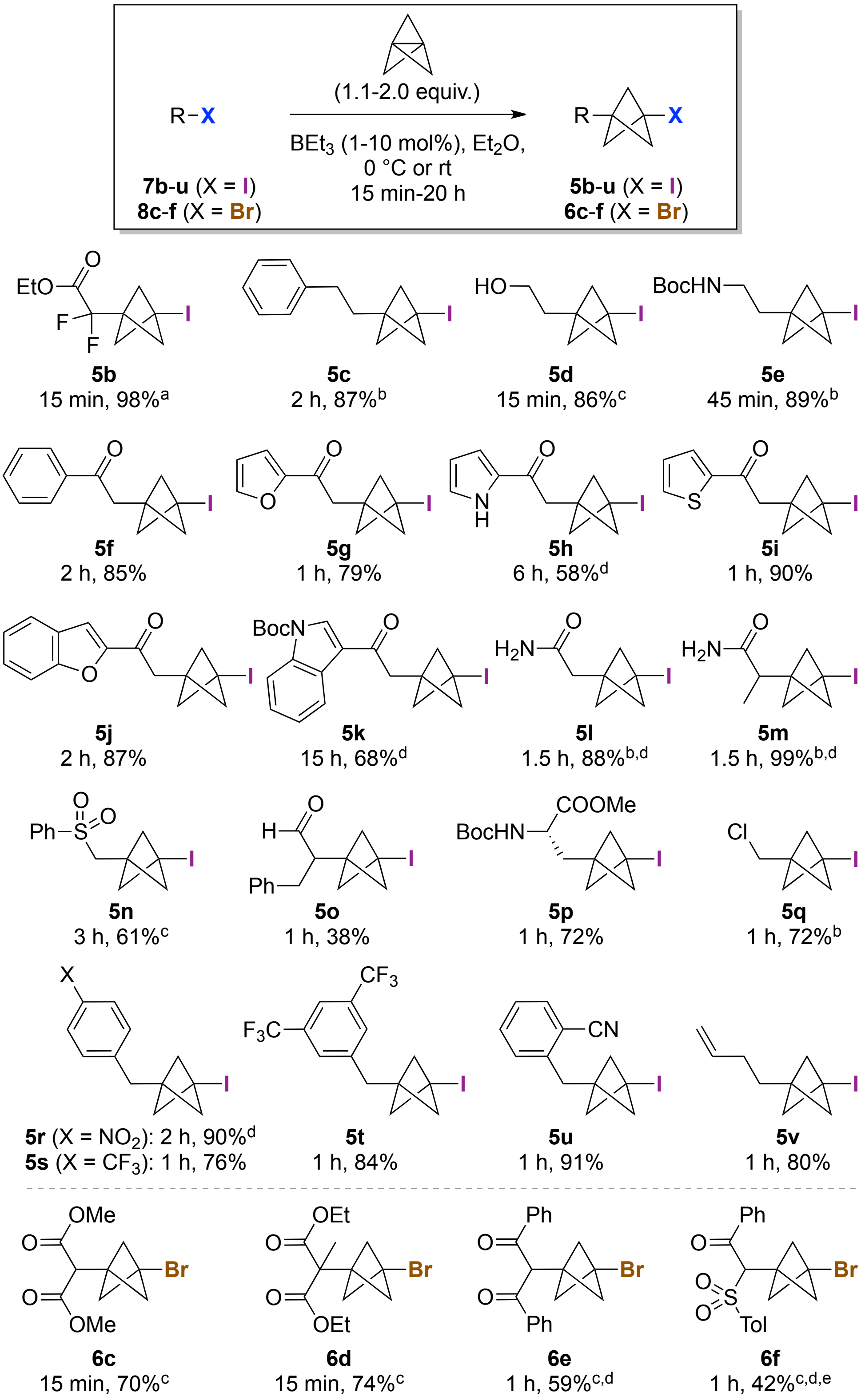

All reactions performed using 2 equiv. TCP and 10 mol% BEt_3 at room temperature, unless indicated otherwise. [a] 1.1 equiv. TCP, 1 mol% BEt_3, 0 °C. [b] 1.3 equiv. TCP, rt. [c] 1.3 equiv. TCP, 0 °C. [d] Co-solvent added to solubilize the substrate: MeOH for **5m**,**n**; CH_2Cl_2 for **5h**,**k**,**r** and **6e**,**f**. [e] 5% staffane observed.

Figure S1: Synthesis of 1-halo-3-substituted BCPs

Ethyl 2,2-difluoro-2-(3-iodobicyclo[1.1.1]pentan-1-yl)acetate, 5b

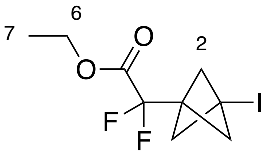

Ethyl iododifluoroacetate **7b** (74 μL, 0.50 mmol, 1.0 equiv.), TCP (0.76 mL, 0.725 M in Et_2O, 0.55 mmol, 1.1 equiv.) and BEt_3 (5 μL, 1 M in hexane, 1 mol %, 5 μmol) were submitted to **General Procedure 1** at 0 °C for 15 min. Purification by column chromatography (SiO_2, petroleum ether / Et_2O, 95:5) afforded **5b** (156 mg, 0.49 mmol, 98%) as a colourless oil.

$\mathbf{R_f}$ = 0.38 (petroleum ether / Et_2O, 9:1)
^{1}H NMR (400 MHz, $CDCl_3$) δ 4.26 (2H, q, *J* = 7.1 Hz, H6), 2.37 (6H, s, 3 × H2), 1.28 (3H, t, *J* = 7.1 Hz, H7).
^{13}C NMR (101 MHz, $CDCl_3$) δ 162.6 (t, *J* = 32.8 Hz), 110.7 (t, *J* = 252.2 Hz), 63.2, 57.9 (t, *J* = 2.9 Hz), 46.4 (t, *J* = 32.7 Hz), 14.2, 3.8 (t, *J* = 2.4 Hz).
^{19}F NMR (377 MHz, $CDCl_3$) δ –109.1.
HRMS (CI^+) Found $[M+H]^+$ = 316.9843; $C_9H_{11}F_2IO_2$ requires 316.9845.
IR (film) ν_{max}/cm^{-1} 2984, 1765, 1372, 1301, 1197, 1155, 1123, 1063, 1027, 918, 862, 828, 713.

This reaction was also conducted on a 5.00 mmol scale using ethyl iododifluoroacetate **7b** (0.74 mL, 5.00 mmol, 1.0 equiv.), TCP (7.6 mL, 0.725 M in Et_2O, 5.50 mmol, 1.1 equiv.) and BEt_3 (50 μL, 1 M in hexane, 1 mol %, 50 μmol), for 15 min at 0 °C. This afforded **5b** in 94% yield (1.50 g).

1-iodo-3-phenethylbicyclo[1.1.1]pentane, 5c

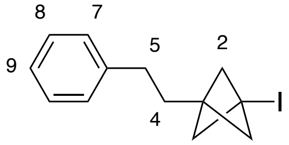

7c (72 μL, 0.50 mmol, 1.0 equiv.), TCP (0.82 mL, 0.79 M in Et_2O, 0.65 mmol, 1.3 equiv.) and BEt_3 (50 μL, 50.0 μmol, 10 mol %) were submitted to **General Procedure 1** at 0 °C for 2 h in the dark. Purification by recrystallization from methanol (cooled to −20 °C) afforded **5c** (130 mg, 0.44 mmol, 87%) as white crystals.

m.p. = 51-52°C
$\mathbf{R_f}$ = 0.65 (petroleum ether / Et_2O / triethylamine, 99:1:1)

^{1}**H NMR** (400 MHz, C_6D_6) δ 7.15-7.09 (2H, m, H8), 7.08-7.02 (1H, m, H9), 6.91-6.85 (2H, m, H7), 2.21-2.13 (2H, m, H5), 1.87 (6H, s, H2), 1.44-1.35 (2H, m, H4).

^{13}**C NMR** (101 MHz, C_6D_6) δ 141.6, 128.6, 128.4, 126.3, 60.4, 48.2, 33.3, 33.1, 7.4.

HRMS (CI^+) Found $[M+H]^+$ = 299.0295, $C_{13}H_{16}I$ requires 299.0291.

IR (film) ν_{max}/cm^{-1} 3025, 2988, 2911, 2875, 1603, 1496, 1453, 1172, 1135, 1009, 980, 836, 699.

2-(3-iodobicyclo[1.1.1]pentan-1-yl)ethan-1-ol, 5d

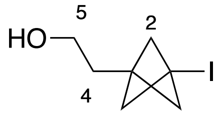

2-iodoethanol (39 μL, 0.50 mmol, 1.0 equiv.), TCP (0.82 mL, 0.79 M in Et_2O, 0.65 mmol, 1.3 equiv.) and BEt_3 (50 μL, 50 μmol, 10 mol %) were submitted to **General Procedure 1** at 0 °C for 15 min. Purification by column chromatography (SiO_2, petroleum ether / Et_2O, 1:0 to 0:1) afforded **5d** (96 mg, 0.40 mmol, 80%) as a white solid that proved unstable on prolonged storage.

m.p. = 44-45°C

$\mathbf{R_f}$ = 0.19 (petroleum ether / EtOAc, 8:2)

^{1}**H NMR** (400 MHz, $CDCl_3$) δ 3.63 (2H, t, *J* = 6.5 Hz, H5), 2.25 (6H, s, H2), 1.78 (2H, t, *J* = 6.5 Hz, H4).

^{13}**C NMR** (101 MHz, $CDCl_3$) δ 61.1, 60.9, 46.3, 34.8, 7.3.

HRMS (EI^+) Found $[M–I]^+$ = 111.0802; $C_7H_{11}O$ requires 111.0804.

IR (film) ν_{max}/cm^{-1} 3327, 2988, 2912, 2876, 1446, 1424, 1173, 1126, 1101, 1043, 981, 853.

***tert*-Butyl (2-(3-iodobicyclo[1.1.1]pentan-1-yl)ethyl)carbamate, 5e**

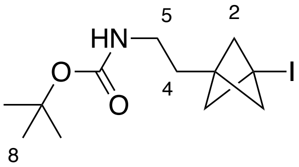

7e (271 mg, 1.00 mmol, 1.0 equiv.), TCP (1.22 mL, 1.07 M in Et_2O, 1.30 mmol, 1.3 equiv.) and BEt_3 (100 μL, 1.0 M in hexane, 100 μmol, 10 mol %) were submitted to **General Procedure 1** at room temperature for 45 min. Purification by column chromatography (SiO_2, petroleum ether / Et_2O, 3:1) afforded **5e** (303 mg, 0.90 mmol, 90%) as a white solid.

R_f = 0.40 (petroleum ether / Et_2O, 3:1)

m.p. = 63-65 °C

^{1}H NMR (400 MHz, $CDCl_3$) δ_H 4.44 (1H, br s, NH), 3.15-3.01 (2H, m, H5), 2.23 (6H, s, H2), 1.71 (2H, t, J = 7.3 Hz, H4), 1.43 (9H, s, *t*-Bu).

^{13}C NMR (101 MHz, $CDCl_3$) δ_C 155.8, 79.5, 60.7, 46.5, 38.4, 32.3, 28.5, 7.1.

HRMS (ESI^+) Found $[2M+H]^+$ = 675.1154; $C_{24}H_{41}I_2N_2O_4$ requires 675.1150

IR (film) ν_{max}/cm^{-1} 3317, 1683, 1172, 835, 752.

2-(3-Iodobicyclo[1.1.1]pentan-1-yl)-1-phenylethan-1-one, 5f

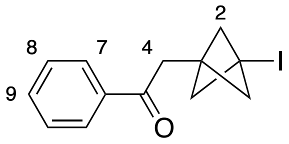

7f (74 mg, 0.30 mmol, 1.0 equiv.), TCP (0.80 mL, 0.73 M in Et_2O, 0.60 mmol, 2.0 equiv.) and BEt_3 (30 µL, 1.0 M in hexane, 30 µmol, 10 mol %) were submitted to **General Procedure 1** at room temperature for 2 h. Purification by column chromatography (SiO_2, petroleum ether / Et_2O, 98:2→96:4) afforded **5f** (80 mg, 0.26 mmol, 85%) as a white solid.

R_f = 0.16 (petroleum ether / Et_2O, 97:3)

m.p. = 68-70 °C

^{1}H NMR (400 MHz, $CDCl_3$) δ 7.91-7.84 (2H, m, H7), 7.61-7.54 (1H, m, H9), 7.49-7.43 (2H, m, H8), 3.20 (2H, s, H4), 2.34 (6H, s, H2).

^{13}C NMR (101 MHz, $CDCl_3$) δ 197.3, 136.8, 133.5, 128.8, 128.3, 61.4, 44.1, 40.8, 6.6.

HRMS (CI^+) Found $[M+H]^+$ = 313.0081; $C_{13}H_{14}IO$ requires 313.0084.

IR (film) ν_{max}/cm^{-1} 3007, 1686, 1170, 1101, 835.

1-(Furan-2-yl)-2-(3-iodobicyclo[1.1.1]pentan-1-yl)ethan-1-one, 5g

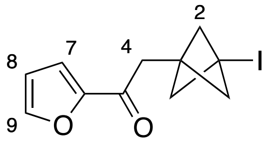

7g (70 mg, 0.30 mmol, 1.0 equiv.), TCP (0.80 mL, 0.73 M in Et_2O, 0.60 mmol, 2.0 equiv.) and BEt_3 (30 µL, 1.0 M in hexane, 30 µmol, 10 mol %) were submitted to **General Procedure 1** at room temperature for 1 h. Purification by column chromatography (SiO_2, petroleum ether / Et_2O, 95:5→90:10) afforded **5g** (72 mg, 0.24 mmol, 79%) as a white solid.

R_f = 0.32 (petroleum ether / Et_2O, 7:3)

m.p. = 51-53 °C

^{1}H NMR (400 MHz, $CDCl_3$) δ 7.58 (1H, dd, J = 1.7, 0.8 Hz, H9), 7.16 (1H, dd, J = 3.6, 0.8 Hz, H7), 6.54 (1H, dd, J = 3.6, 1.7 Hz, H8), 3.04 (2H, s, H4), 2.32 (6H, s, H2).

^{13}C NMR (101 MHz, $CDCl_3$) δ 186.1, 152.6, 146.8, 117.6, 112.6, 61.3, 44.0, 41.1, 6.3.

HRMS (CI^+) Found $[M+H]^+$ = 302.9873; $C_{11}H_{12}IO_2$ requires 302.9876.

IR (film) ν_{max}/cm^{-1} 2981, 1668, 1468, 1160, 836, 788.

2-(3-Iodobicyclo[1.1.1]pentan-1-yl)-1-(1H-pyrrol-2-yl)ethan-1-one, 5h

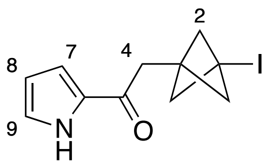

7h (35 mg, 0.15 mmol, 1.0 equiv.), TCP (0.37 mL, 0.82 M in Et_2O, 0.30 mmol, 2.0 equiv.) and BEt_3 (15 μL, 1.0 M in hexane, 15 μmol, 0.1 equiv.) in CH_2Cl_2 (0.3 mL) were submitted to **General Procedure 1** at room temperature for 6 h. Purification by column chromatography (SiO_2, petroleum ether / Et_2O, 8:2) afforded **5h** (26 mg, 0.09 mmol, 58%) as a white solid.

R_f = 0.39 (petroleum ether / Et_2O, 7:3)

m.p. = 105-107 °C

^{1}H NMR (400 MHz, $CDCl_3$) δ_H 9.83 (1H, br s, NH), 7.11-7.01 (1H, m, H9), 6.87-6.78 (1H, m, H7), 6.28 (1H, dt, J = 3.7, 2.5 Hz, H8), 2.98 (2H, s, H4), 2.32 (6H, s, H2).

^{13}C NMR (101 MHz, $CDCl_3$) δ_C 187.1, 131.9, 125.5, 117.2, 111.0, 61.4, 44.5, 40.7, 6.5.

HRMS (CI^+) Found $[M+H]^+$ = 302.0024; $C_{11}H_{13}INO$ requires 302.0036.

IR (film) ν_{max}/cm^{-1} 3287, 1629, 1172, 833, 727.

2-(3-Iodobicyclo[1.1.1]pentan-1-yl)-1-(thiophen-2-yl)ethan-1-one, 5i

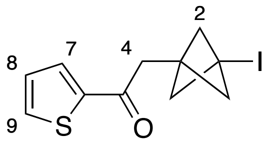

7i (76 mg, 0.30 mmol, 1.0 equiv.), TCP (0.80 mL, 0.73 M in Et_2O, 0.60 mmol, 2.0 equiv.) and BEt_3 (30 μL, 1.0 M in hexane, 30 μmol, 0.1 equiv.) were submitted to **General Procedure 1** at room temperature for 1 h. Purification by column chromatography (SiO_2, petroleum ether / Et_2O, 95:5→90:10) afforded **5i** (86 mg, 0.27 mmol, 90%) as a white solid.

R_f = 0.37 (petroleum ether / Et_2O, 8:2)

m.p. = 70-72 °C

^{1}H NMR (400 MHz, $CDCl_3$) δ 7.66 (1H, dd, *J* = 5.0, 1.1 Hz, H9), 7.62 (1H, dd, *J* = 3.8, 1.1 Hz, H7), 7.13 (1H, dd, *J* = 5.0, 3.8 Hz, H8), 3.12 (2H, s, H4), 2.34 (6H, s, H2).

^{13}C NMR (101 MHz, $CDCl_3$) δ 189.9, 144.2, 134.4, 132.4, 128.4, 61.3, 44.1, 41.9, 6.2.

HRMS (CI^+) Found $[M+H]^+$ = 318.9647; $C_{11}H_{11}IOS$ requires 318.9648.

IR (film) ν_{max}/cm^{-1} 2910, 1660, 1421, 1221, 1170, 828, 741.

1-(Benzofuran-2-yl)-2-(3-iodobicyclo[1.1.1]pentan-1-yl)ethan-1-one, 5j

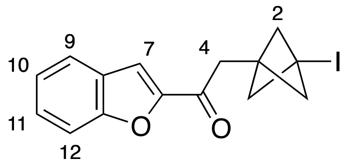

7j (72 mg, 0.25 mmol, 1.0 equiv.), TCP (0.70 mL, 0.73 M in Et_2O, 0.50 mmol, 2.0 equiv.) and BEt_3 (25 µL, 1.0 M in hexane, 25 µmol, 10 mol %) were submitted to **General Procedure 1** at room temperature for 2 h. Purification by column chromatography (SiO_2, petroleum ether / Et_2O, 95:5) afforded **5j** (76 mg, 0.22 mmol, 87%) as a white solid.

R_f = 0.60 (petroleum ether / Et_2O, 7:3)

m.p. = 82-84 °C

^{1}H NMR (400 MHz, $CDCl_3$) δ 7.72 (1H, dt, *J* = 8.0, 1.0 Hz, H9), 7.57 (1H, dd, *J* = 8.4, 1.0 Hz, H12), 7.52-7.46 (2H, m, H11, H7), 7.33 (1H, ddd, *J* = 8.0, 7.1, 1.0 Hz, H10), 3.19 (2H, s, H4), 2.37 (6H, s, H2).

^{13}C NMR (101 MHz, $CDCl_3$) δ 188.1, 155.8, 152.4, 128.7, 127.1, 124.2, 123.5, 113.3, 112.6, 61.3, 43.9, 41.4, 6.2.

HRMS (CI^+) Found $[M+H]^+$ = 353.0041; $C_{15}H_{14}IO_2$ requires 353.0033.

IR (film) ν_{max}/cm^{-1} 1671, 1550, 1180, 1166, 759.

***tert*-Butyl 3-(2-(3-iodobicyclo[1.1.1]pentan-1-yl)acetyl)-1H-indole-1-carboxylate, 5k**

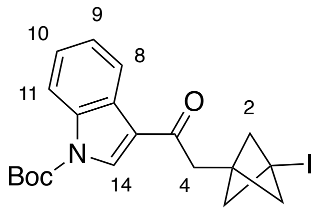

7k (58 mg, 0.15 mmol, 1.0 equiv.), TCP (0.37 mL, 0.82 M in Et_2O, 0.30 mmol, 2.0 equiv.) and BEt_3 (15 μL, 1.0 M in hexane, 15 μmol, 10 mol %) in CH_2Cl_2 (0.3 mL) were submitted to **General Procedure 1** at room temperature for 15 h. Purification by column chromatography (SiO_2, petroleum ether / Et_2O, 95:5) afforded **5k** (46 mg, 0.10 mmol, 68%) as a white solid.

R_f = 0.44 (petroleum ether / Et_2O, 7:3)

m.p. = 130-132 °C

^{1}H NMR (400 MHz, $CDCl_3$) δ 8.38-8.33 (1H, m, H8), 8.14 (1H, s, H14), 8.12-8.07 (1H, m, H11), 7.42-7.32 (2H, m, H10, H9), 3.10 (2H, s, H4), 2.37 (6H, s, H2), 1.72 (9H, s, *t*-Bu).

^{13}C NMR (101 MHz, $CDCl_3$) δ 193.1, 149.2, 135.7, 132.3, 127.5, 125.8, 124.7, 122.8, 120.3, 115.1, 85.8, 61.4, 44.4, 42.4, 28.3, 6.5.

HRMS (CI^+) Found $[M+H]^+$ = 452.0717; $C_{20}H_{23}INO_3$ requires 452.0717.

IR (film) ν_{max}/cm^{-1} 1744, 1451, 1130, 844, 761.

2-(3-Iodobicyclo[1.1.1]pentan-1-yl)acetamide, 5l

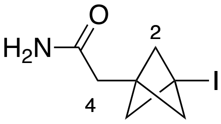

2-iodoacetamide (92 mg, 0.50 mmol, 1.0 equiv.), TCP (0.82 mL, 0.79 M in Et_2O, 0.65 mmol, 1.3 equiv.) and BEt_3 (50 μL, 1.0 M in hexane, 50 μmol, 10 mol %) in MeOH (0.3 mL) were submitted to **General Procedure 1** at room temperature for 1.5 h. Purification by column chromatography (SiO_2, EtOAc) afforded **5l** (111 mg, 0.44 mmol, 88%) as a white solid.

R_f = 0.19 (EtOAc)

m.p. = 118-120 °C

^{1}H NMR (400 MHz, $CDCl_3$) δ 5.61 (1H, br s, NH), 5.34 (1H, br s, NH), 2.44 (2H, s, H4), 2.33 (6H, s, H2).

^{13}C NMR (101 MHz, $CDCl_3$) δ_C 171.7, 61.0, 44.2, 39.2, 5.9.

HRMS (CI^+) Found $[M+H]^+$ = 251.9876; $C_7H_{11}INO$ requires 251.9880.

IR (film) ν_{max}/cm^{-1} 3353, 3167, 1658, 1629, 1404, 1177, 842, 661.

2-(3-Iodobicyclo[1.1.1]pentan-1-yl)propanamide, 5m

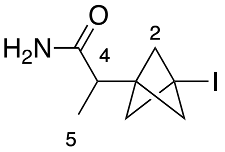

7m (60 mg, 0.30 mmol, 1.0 equiv.), TCP (0.50 mL, 0.79 M in Et_2O, 0.39 mmol, 1.3 equiv.) and BEt_3 (30 µL, 1.0 M in hexane, 30 µmol, 10 mol %) in MeOH (0.5 mL) were submitted to **General Procedure 1** at room temperature for 1.5 h. Purification by column chromatography (SiO_2, EtOAc) afforded **5m** as a white solid (79 mg, 0.30 mmol, 99%). Recrystallization by slow evaporation of a solution in $CDCl_3$ afforded crystals that were suitable for X-ray diffraction (see Section 10).

R_f = 0.28 (EtOAc)
m.p. = 108-110 °C
^{1}H NMR (400 MHz, $CDCl_3$) δ 5.29 (2H, br s, NH_2), 2.50 (1H, q, J = 6.9 Hz, H4), 2.26 (6H, s, H2), 1.10 (3H, d, J = 6.9 Hz, H5).
^{13}C NMR (101 MHz, $CDCl_3$) δ 174.8, 59.2, 48.9, 42.7, 14.2, 6.0.
HRMS (CI^+) Found $[M+H]^+$ = 266.0035; $C_8H_{13}INO$ requires 266.0036.
IR (film) ν_{max}/cm^{-1} 3424, 3170, 1657, 1172, 834.

1-Iodo-3-((phenylsulfonyl)methyl)bicyclo[1.1.1]pentane, 5n

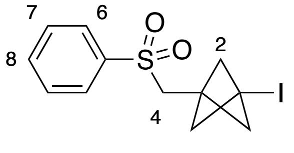

7n (142 mg, 0.50 mmol, 1.0 equiv.), TCP (0.82 mL, 0.79 M in Et_2O, 0.65 mmol, 1.3 equiv.) and BEt_3 (50 µL, 50.0 µmol, 10 mol %) were submitted to **General Procedure 1** at 0 °C for 3 h. Purification by column chromatography (SiO_2, petroleum ether / Et_2O, 1:0 to 0:1) afforded **5n** (107 mg, 0.31 mmol, 61%) as a white solid.

R_f = 0.16 (petroleum ether / Et_2O, 7:3)
m.p. = 111-112 °C
^{1}H NMR (400 MHz, $CDCl_3$) δ 7.92-7.86 (2H, m, H6), 7.71-7.64 (1H, m, H8), 7.61-7.54 (2H, m, H7), 3.34 (2H, s, H4), 2.32 (6H, s, 3 × H2).
^{13}C NMR (101 MHz, $CDCl_3$) δ 139.8, 134.1, 129.6, 127.9, 61.2, 57.1, 40.6, 5.0.
HRMS (ESI^+) Found $[M+Na]^+$ = 370.9573; $C_{12}H_{13}O_2{}^{127}I^{23}Na^{32}S$ requires 370.9573.
IR (film) ν_{max}/cm^{-1} 2995, 2912, 1446, 1302, 1282, 1184, 1145, 1082, 857, 743, 688.

2-(3-Iodobicyclo[1.1.1]pentan-1-yl)-3-phenylpropanal, 5o

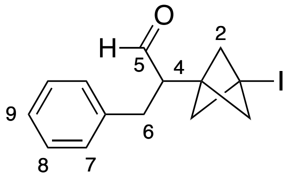

7l (78 mg, 0.30 mmol, 1.0 equiv.), TCP (0.76 mL, 0.79 M in Et_2O, 0.60 mmol, 2.0 equiv.) and BEt_3 (30 μL, 1.0 M in hexane, 30 μmol, 10 mol %) were submitted to **General Procedure 1** at room temperature for 1 h. Purification by column chromatography (SiO_2, petroleum ether / Et_2O, 9:1) afforded **5o** (37 mg, 0.11 mmol, 38%) as a colourless oil.

$\mathbf{R_f}$ = 0.32 (petroleum ether / Et_2O, 9:1)
^{1}H NMR (400 MHz, $CDCl_3$) δ 9.64 (1H, d, J = 2.3 Hz, H5), 7.33-7.26 (2H, m, H8), 7.26-7.20 (1H, m, H9), 7.20-7.12 (2H, m, H7), 3.04 (1H, dd, J = 13.9, 8.5 Hz, H6), 2.95 (1H, ddd, J = 8.5, 5.9, 2.3 Hz, H4), 2.73 (1H, dd, J = 13.9, 5.9 Hz, H6), 2.30 (3H, dd, J = 9.4, 1.6 Hz, H2), 2.25 (3H, dd, J = 9.4, 1.6 Hz, H2).
^{13}C NMR (101 MHz, $CDCl_3$) δ_C 201.2, 138.2, 128.8, 128.8, 126.8, 59.8, 54.6, 46.5, 33.0, 6.4.
HRMS (CI^+) Found $[M+H]^+$ = 327.0242; $C_{14}H_{16}IO$ requires 327.0240.
IR (film) ν_{max}/cm^{-1} 2915, 1724, 1177, 838, 699.

Methyl (*S*)-2-((tert-butoxycarbonyl)amino)-3-(3-iodobicyclo [1.1.1]pentan-1-yl)propanoate, 5p

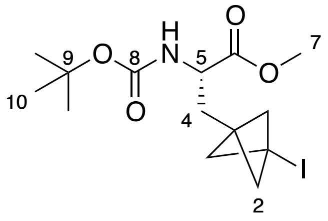

7p (165 mg, 0.50 mmol, 1.0 equiv.), TCP (1.32 mL, 0.76 M in Et_2O, 1.00 mmol, 2.0 equiv.) and BEt_3 (50 μL, 1 M in hexane, 50 μmol, 10 mol %) were submitted to **General Procedure 1** at room temperature for 1 h. Purification by column chromatography (SiO_2, petroleum ether / Et_2O, 8:2) and recrystallization from petroleum ether afforded **5p** (142 mg, 0.36 mmol, 72%) as a white fluffy solid.

$\mathbf{R_f}$ = 0.13 (petroleum ether / Et_2O, 9:1)
m.p. = 83-84°C

^{1}**H NMR** (400 MHz, $CDCl_3$) δ 4.98 (1H, d, *J* = 8.4 Hz, NH), 4.29 (1H, td, *J* = 7.9, 4.4 Hz, H5), 3.73 (3H, s, H7), 2.29-2.20 (6H, m, H2), 2.12 (1H, dd, J = 14.6, 4.0 Hz, H4), 1.86 (1H, dd, *J* = 14.6, 7.6 Hz, H4), 1.44 (9H, s, *t*-Bu).

^{13}**C NMR** (101 MHz, $CDCl_3$) δ 172.8, 155.1, 80.3, 61.0, 52.6, 52.0, 45.5, 34.5, 28.5, 6.4.

HRMS (CI^+) Found $[M+H]^+$ = 396.0679; $C_7H_9O_2$ requires 396.0666.

IR (film) ν_{max}/cm^{-1} 3358, 2978, 1744, 1713, 1509, 1366, 1176.

$[\alpha]_D^{25}$ –0.8 (c = 3.0, MeOH)

Methyl (*R*)-2-((tert-butoxycarbonyl)amino)-3-(3-iodobicyclo [1.1.1]pentan-1-yl)propanoate, 5p′

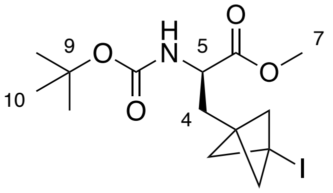

7p′ (165 mg, 1.0 equiv., 0.50 mmol), TCP (1.27 mL, 0.79 M in Et_2O, 2.0 equiv., 1.00 mmol) and BEt_3 (50 uL, 1 M in hexane, 10 mol%, 50 μmol) were submitted to **General Procedure 1**. Purification by column chromatography (pentane / Et_2O, 1:0 to 8:2) and recrystallisation from pentane at –20 °C afforded **5p′** (92 mg, 46%) as a white fluffy solid.

R_f = 0.13 (petroleum ether / Et_2O, 9:1)

m.p. = 91-92 °C

^{1}**H NMR** (400 MHz, $CDCl_3$) δ 4.98 (1H, d, *J* = 8.5 Hz, NH), 4.29 (1H, td, *J* = 8.0, 4.5 Hz, H5), 3.73 (3H, s, H7), 2.28 – 2.19 (6H, m, H2), 2.12 (1H, dd, *J* = 14.6, 4.4 Hz, H4), 1.86 (1H, dd, *J* = 14.6, 7.6 Hz, H4), 1.44 (9H, s, *t*-Bu).

^{13}**C NMR** (101 MHz, $CDCl_3$) δ 172.8, 155.1, 80.3, 61.0, 52.6, 52.0, 45.5, 34.5, 28.5, 6.4.

HRMS (ESI^+) Found $[M+Na]^+$ = 418.0480; $C_{14}H_{22}O_4N^{127}I^{23}Na$ requires 418.0486

IR (film) ν_{max}/cm^{-1} 3360, 2976, 1747, 1715, 1507, 1366, 1178.

$[\alpha]_D^{25}$ 0.7 ° (C = 3.0, MeOH)

HPLC trace for **5p** (Chiralpak IB, 5% IPA in hexane, flow rate = 1.3 mL/min, 254 nm)

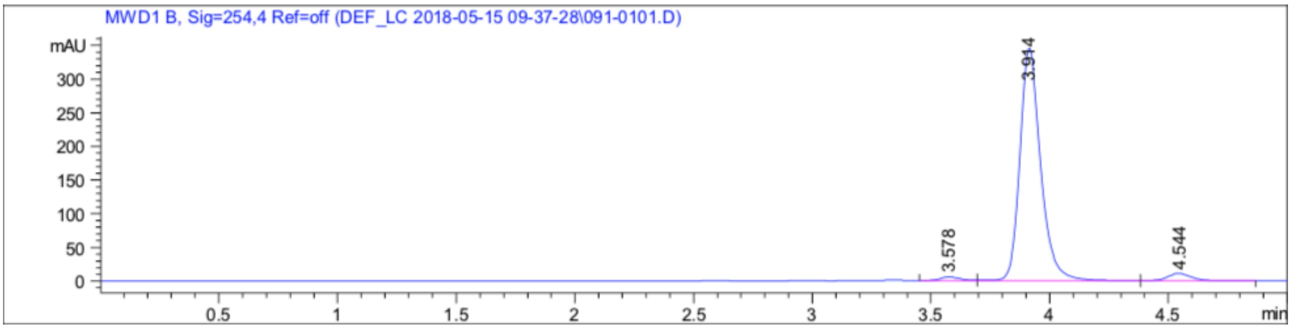

HPLC trace for **5p′** (Chiralpak IB, 5% IPA in hexane, flow rate = 1.3 mL/min, 254 nm)

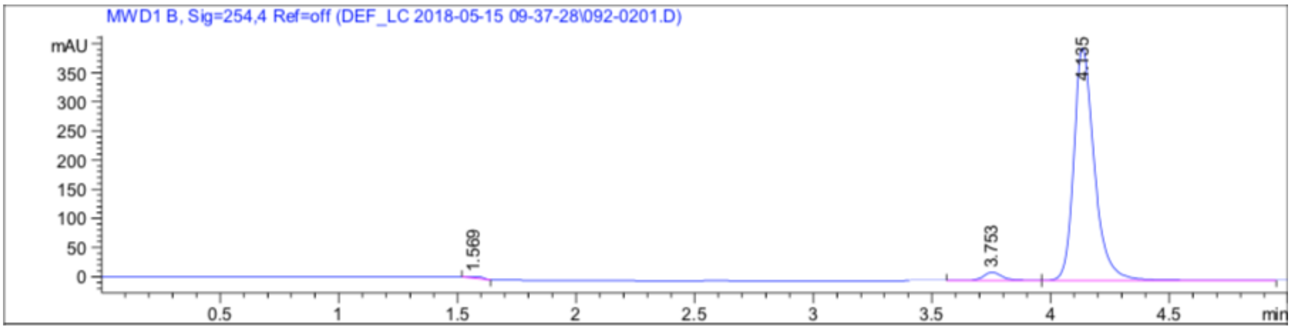

HPLC trace for a mixture of **5p** and **5p′** (Chiralpak IB, 5% IPA in hexane, flow rate = 1.3 mL/min, 254 nm)

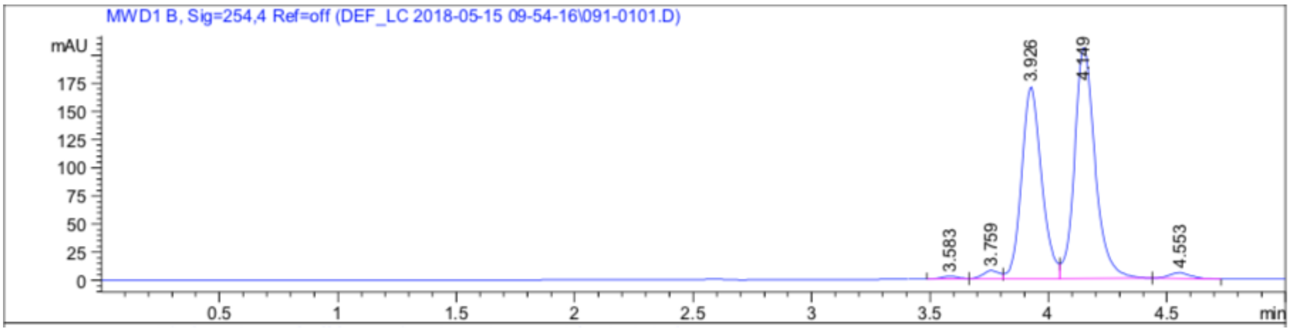

1-Iodo-3-(chloromethyl)bicyclo[1.1.1]pentane, 5q

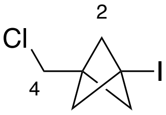

Chloroiodomethane (36 μL, 0.50 mmol, 1.0 equiv.), TCP (1.1 mL, 0.79 M in Et_2O, 0.65 mmol, 31.3 equiv.) and BEt_3 (50 μL, 1 M in hexane, 50 μmol, 10 mol %) were submitted to **General Procedure 1** at room temperature for 1 h. Purification by column chromatography (SiO_2, pentane) afforded **5q** (87 mg, 0.36 mmol, 72%) as a white solid.

$\mathbf{R_f}$ = 0.43 (pentane)

m.p. = 34-35 °C

^{1}H NMR (400 MHz, $CDCl_3$) δ 3.53 (2H, s, H4), 2.30 (6H, s, H2).

^{13}C NMR (101 MHz, $CDCl_3$) δ 59.3, 47.3, 44.1, 6.1.
HRMS (CI^+) Found $[M–I]^+$ = 115.0309; C_6H_8Cl requires 115.0309.
IR (film) ν_{max}/cm^{-1} 2996, 2915, 1265, 1178, 850, 721.

1-Iodo-3-(4-nitrobenzyl)bicyclo[1.1.1]pentane, 5r

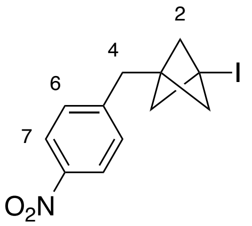

7r (132 mg, 0.50 mmol, 1.0 equiv.), TCP (0.90 mL, 0.90 M in Et_2O, 1.00 mmol, 2.0 equiv.) and BEt_3 (50 μL, 50 μmol, 10 mol %) were submitted to **General Procedure 1** at room temperature for 1 h. Purification by column chromatography (SiO_2, pentane / Et_2O, 95:5) afforded **5r** (148 mg, 0.45 mmol, 90%) as white crystals. Recrystallization from petroleum ether / CH_2Cl_2 afforded crystals that were suitable for X-ray diffraction (see Section 10).

R_f = 0.25 (pentane / Et_2O, 95:5)
m.p. = 76 °C (dec.)
^{1}H NMR (400 MHz, $CDCl_3$) δ 8.19-8.13 (2H, m, H7), 7.24-7.20 (2H, m, H6), 2.92 (2H, s, H4), 2.16 (6H, s, H2).
^{13}C NMR (101 MHz, $CDCl_3$) δ 146.9, 145.9, 129.7, 124.0, 60.2, 47.5, 39.2, 7.0.
HRMS (CI^+) Found $[M+NH_4]^+$ = 347.0256; $C_{12}H_{16}IN_2O_2$ requires 347.0251.
IR (film) ν_{max}/cm^{-1} 2997, 1596, 1514, 1343, 1174, 1105, 854, 831, 700.

1-iodo-3-(4-(trifluoromethyl)benzyl)bicyclo[1.1.1]pentane, 5s

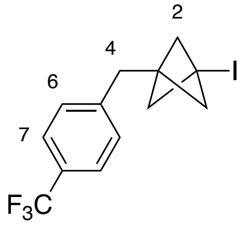

7s (143 mg, 0.50 mmol, 1.0 equiv.), TCP (1.27 mL, 0.79 M in Et_2O, 1.00 mmol, 2.0 equiv.) and BEt_3 (50 μL, 50 μmol, 10 mol %) were submitted to **General Procedure 1** at room temperature for 1 h. Purification by column chromatography (SiO_2, petroleum ether) afforded **5s** (134 mg, 0.38 mmol, 76%) as white crystals. Recrystallization with petroleum ether from CH_2Cl_2 afforded crystals that were suitable for X-ray diffraction (see Section 10).

R_f = 0.48 (petroleum ether)

m.p. = 84-86 °C

^{1}H NMR (400 MHz, $CDCl_3$) δ 7.55 (2H, d, J = 8.0 Hz, H7), 7.17 (2H, d, J = 8.0 Hz, H6), 2.87 (2H, s, H4), 2.16 (6H, s, H2).

^{13}C NMR (101 MHz, $CDCl_3$) δ 142.4, 129.2, 128.9 (q, J = 32.4 Hz), 125.5 (q, J = 3.8 Hz), 124.4 (q, J = 271.9 Hz), 60.3, 47.9, 39.1, 7.6.

^{19}F NMR (376 MHz, $CDCl_3$) δ −62.41.

HRMS (CI^+) Found $[M+NH_4]^+$ = 370.0280; $C_{13}H_{16}F_3IN$ requires 370.0274.

IR (film) ν_{max}/cm^{-1} 2915, 1320, 1157, 1107, 1063, 1016, 843.

1-(3,5-bis(trifluoromethyl)benzyl)-3-iodobicyclo[1.1.1]pentane, 5t

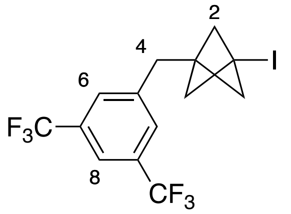

7t (177 mg, 0.50 mmol, 1.0 equiv.), TCP (1.27 mL, 0.79 M in Et_2O, 1.00 mmol, 2.0 equiv.) and BEt_3 (50 μL, 50 μmol, 10 mol %) were submitted to **General Procedure 1** at room temperature for 1 h. Purification by column chromatography (SiO_2, petroleum ether) afforded **5t** (176 mg, 0.42 mmol, 84%) as a yellowish solid.

R_f = 0.30 (petroleum ether)

m.p. = 45-46 °C

^{1}H NMR (400 MHz, $CDCl_3$) δ 7.84-7.64 (1H, m, H8), 7.57-7.40 (2H, m, H6), 2.96 (2H, s, H4), 2.17 (6H, s, H2).

^{13}C NMR (101 MHz, $CDCl_3$) δ 140.7, 132.0 (q, J = 33.2 Hz), 128.9 (q, J = 3.0 Hz), 123.4 (d, J = 272.6 Hz), 120.7 (m), 60.1, 47.5, 39.0, 6.7.

^{19}F NMR (376 MHz, $CDCl_3$) δ -62.88.

HRMS (EI^+) Found $[M–I]^+$ = 293.0762; $C_{14}H_{11}F_6$requires 293.0759.

IR (film) ν_{max}/cm^{-1}2993, 2917, 1378, 1276, 1171, 1128, 981, 923, 895, 844, 729, 707, 682.

2-((3-iodobicyclo[1.1.1]pentan-1-yl)methyl)benzonitrile, 5u

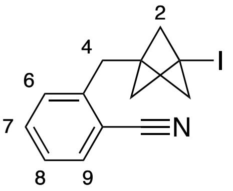

7u (366 mg, 1.50 mmol, 1.0 equiv.), TCP (3.81 mL, 0.79 M in Et_2O, 4.50 mmol, 2.0 equiv.) and BEt_3 (150 µL, 150 µmol, 10 mol %) were submitted to **General Procedure 1** at room temperature for 1 h. Purification by column chromatography (SiO_2, petroleum ether / Et_2O, 1:0→1:1) afforded **5u** (423 mg, 1.37 mmol, 91%) as a white solid.

$\mathbf{R_f}$ = 0.37 (Petroleum ether:Et_2O, 9:1)
m.p. = 99-101 °C
^{1}**H NMR** (400 MHz, $CDCl_3$) δ 7.63 (1H, d, *J* = 7.7 Hz, H9), 7.53 (td, *J* = 7.7, 1.3 Hz, H7), 7.33 (td, *J* = 7.7, 1.3 Hz, H8), 7.19 (1H, d, *J* = 7.7 Hz, H6), 3.07 (2H, s, H4), 2.19 (6H, s, H2).
^{13}**C NMR** (101 MHz, $CDCl_3$) δ 142.2, 133.1, 133.0, 130.1, 127.2, 118.0, 112.6, 60.4, 47.7, 37.7, 7.0.
HRMS (CI^+) Found $[M+NH_4]^+$ = 327.0355; $C_{13}H_{16}IN_2$ requires 327.0352.
IR (film) ν_{max}/cm^{-1} 2914, 2877, 2221, 1482, 1444, 1173, 979, 840, 760.

1-(But-3-en-1-yl)-3-iodobicyclo[1.1.1]pentane, 5v

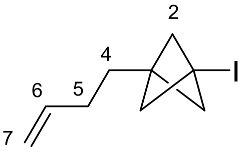

7v (182 mg, 1.00 mmol, 1.0 equiv.), TCP (1.50 mL, 0.89 M in Et_2O, 1.30 mmol, 1.3 equiv.) and BEt_3 (100 µL, 1.0 M in hexane, 100 µmol, 10 mol %) were submitted to **General Procedure 1** at room temperature for 1 h. Purification by column chromatography (SiO_2, pentane) afforded **5v** (199 mg, 0.800 mmol, 80%) as a colorless liquid.

$\mathbf{R_f}$ = 0.62 (pentane)
^{1}**H NMR** (400 MHz, $CDCl_3$) δ_H 5.76 (1H, ddt, *J* = 16.8, 10.2, 6.5 Hz, H6), 5.03-4.91 (2H, m, H7), 2.20 (6H, s, H2), 2.07-1.96 (2H, m, H5), 1.66-1.57 (2H, m, H4).
^{13}**C NMR** (101 MHz, $CDCl_3$) δ_C 138.1, 115.0, 60.7, 48.4, 31.4, 31.1, 7.9.
HRMS (ESI) Found $[M+NH_4]^+$ = 266.0406; $C_9H_{17}IN$ requires 266.0400.
IR (film) ν_{max}/cm^{-1} 2987, 1172, 834.

Dimethyl 2-(3-bromobicyclo[1.1.1]pentan-1-yl)malonate, 6c

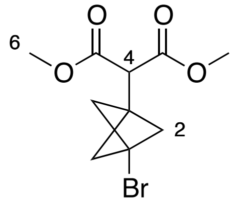

8c (132 μL, 1.00 mmol, 1.0 equiv.), TCP (1.4 mL, 0.90 M in Et_2O, 1.30 mmol, 1.3 equiv.) and triethylborane (100 μL, 1 M in hexane, 100 μmol, 10 mol %) were submitted to **General Procedure 1** at 0 °C for 15 min. Purification by column chromatography (SiO_2, petroleum ether / Et_2O, 9:1) afforded **6c** (244 mg, 0.88 mmol, 88%) as a colourless oil.

R$_f$ = 0.14 (petroleum ether/Et2O, 9:1)

^{1}H NMR (400 MHz, $CDCl_3$) δ 3.76 (6H, s, H6), 3.66 (1H, s, H4), 2.33 (6H, s, H2).

^{13}C NMR (101 MHz, $CDCl_3$) δ 167.4, 58.5, 52.7, 52.3, 38.1, 35.9.

HRMS (CI^+) Found $[M+H]^+$ = 210.9604; $C_{10}H_{14}{}^{79}BrO_4$ requires 277.0070

IR (film) ν_{max}/cm^{-1} 2955, 1735, 1219, 1052, 1022, 868.

Diethyl 2-(3-bromobicyclo[1.1.1]pentan-1-yl)-2-methylmalonate, 6d

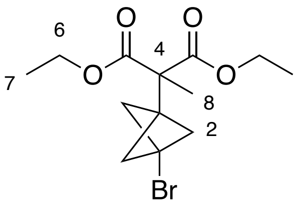

8d (165 mg, 0.5 mmol, 1.0 equiv.), TCP (0.90 mL, 0.725 M in Et_2O, 0.50 mmol, 1.3 equiv.) and BEt_3 (50 μL, 1 M in hexane, 0.05 mmol, 10 mol %) were submitted to **General Procedure 1**. Purification by column chromatography (SiO_2, petroleum ether/Et_2O, 95:5) afforded **6d** (119 mg, 0.37 mmol, 74%) as a colourless oil.

R$_f$ = 0.28 (petroleum ether / Et_2O, 9:1)

^{1}H NMR (400 MHz, $CDCl_3$) δ 4.18 (4H, m, H6), 2.27 (6H, s, H2), 1.38 (3H, s, H8), 1.25 (6H, t, *J* = 7.1 Hz, H7).

^{13}C NMR (101 MHz, $CDCl_3$) δ 170.1, 61.6, 57.4, 54.2, 43.0, 36.4, 18.9, 14.3.

HRMS (CI^+) Found $[M+H]^+$ = 319.0528; $C_{13}H_{19}{}^{79}BrO_4$ requires 319.0540.

IR (film) ν_{max}/cm^{-1} 2981, 1730, 1258, 1188, 1154, 1094, 861.

2-(3-bromobicyclo[1.1.1]pentan-1-yl)-1,3-diphenylpropane-1,3-dione, 6e

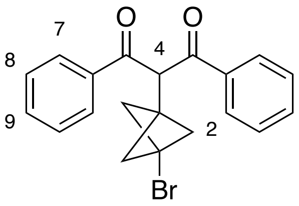

2-bromo-1,3-diphenylpropane-1,3-dione (152 mg, 0.500 mmol, 1.0 equiv.), TCP (0.720 mL, 0.90 M in Et_2O, 1.00 mmol, 1.3 equiv.) and BEt_3 (50 µL, 1 M in hexane, 50 µmol, 10 mol %) were submitted to **General Procedure 1** at 0 °C for 1 h. Purification by column chromatography (SiO_2, petroleum ether / EtOAc, 95:5) afford **6e** (112 mg, 0.30 mmol, 59%) as an off-white solid.

R$_f$ = 0.45 (petroleum ether / EtOAc, 8:2)
m.p. =119-120 °C
^{1}H NMR (400 MHz, $CDCl_3$) δ 7.90 (4H, dd, J = 8.4, 1.4 Hz, H7), 7.63-7.54 (2H, m, H9), 7.51-7.41 (4H, m, H8), 5.53 (1H, s, H4), 2.35 (6H, s, H2).
^{13}C NMR (101 MHz, $CDCl_3$) δ 193.7, 136.0, 134.0, 129.1, 128.6, 59.2, 56.2, 39.1, 36.5.
HRMS (CI^+) Found $[M+H]^+$ = 369.0491; $C_{20}H_{18}{}^{79}BrO_2$ requires 369.0412.
IR (film) ν_{max}/cm^{-1} 2920, 1691, 1672, 1331, 1274, 1174, 866, 822, 756, 691.

2-(3-bromobicyclo[1.1.1]pentan-1-yl)-1-phenyl-2-tosyl-ethan-1-one, 6f

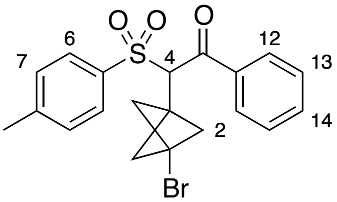

8f (177 mg, 0.50 mmol, 1.0 equiv.), TCP (0.73 mL, 0.90 M in Et_2O, 0.65 mmol, 1.3 equiv.) and BEt_3 (50 µL, 1M in hexane, 50 µmol, 10 mol %) in CH_2Cl_2 (2 mL) were submitted to **General Procedure 1** at 0 °C for 1h. Purification by column chromatography (SiO_2, petroleum ether / EtOAc, 9:1) and recrystallization from CH_2Cl_2 with pentane afforded **6f** (132 mg, 0.31 mmol, 42% yield as determined by ^{1}H NMR spectroscopy, 95:5 mixture with the corresponding staffane derivative) as white crystals.

R$_f$ = 0.18 (Petroleum ether / EtOAc, 8:2)
m.p. = 136-137 °C
^{1}H NMR (400 MHz, $CDCl_3$) δ 7.78-7.74 (2H, m, H12), 7.72-7.67 (2H, m, H6), 7.61-7.55 (1H, m, H14), 7.45-7.39 (2H, m, H13), 7.28-7.23 (2H, m, H7), 5.30 (1H, s, H4), 2.39 (3H, s, H9), 2.37 (3H, dd, J = 9.4, 1.9 Hz, H2), 2.30 (3H, dd, J = 9.4, 1.9 Hz, H2).

^{13}**C NMR** (101 MHz, $CDCl_3$) δ 190.5, 145.7, 136.5, 135.2, 134.3, 129.8, 129.7, 129.0, 128.7, 69.3, 59.4, 37.1, 35.8, 21.8.

HRMS (ESI$^+$) Found $[M+H]^+$ = 419.0298; $C_{20}H_{20}{}^{79}BrO_3S$ requires 419.0311.

IR (film) ν_{max}/cm^{-1} 3011, 2922, 1683, 1596, 1579, 1402, 1324, 1149, 1018, 996, 749, 729, 706.

Staffane derivative: **HRMS** (ESI$^+$) Found $[M+Na]^+$ = 507.0585; $C_{25}H_{25}O_3BrNaS$ requires 507.0600.

5. Substrate limitations

The following substrates proved unreactive to ATRA reaction conditions:

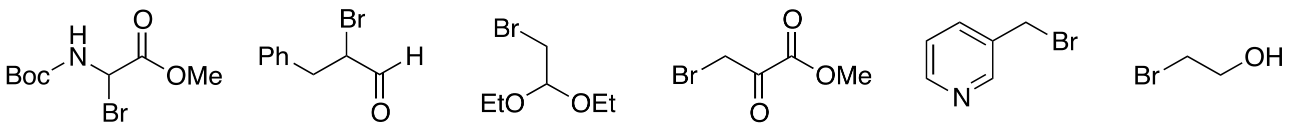

The following substrates failed to reach complete conversion. For bromoethylacetate, a 1:7 ratio of starting material (SM) to product (P) was observed after two iterations of the procedure (40 h in total*)*. For TMS propargyl bromide, a 1:3 ratio of SM:P was observed after 20 h.

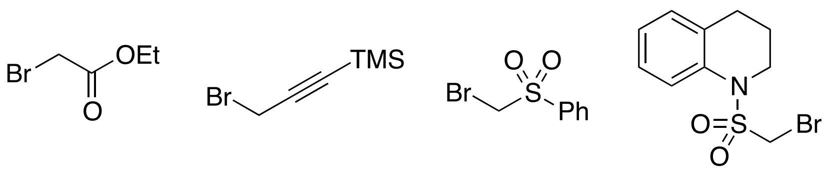

Attempts to effect TCP ring opening as a final step in the synthesis of fentanyl (using the piperidine substrate shown below) were also unsuccessful. We suspect that amine coordination to triethylborane may inhibit the intitation process, as this result contrasts with the successful formation of products **5e**, **5p** and **5x**, which feature Boc-protected amines; and also the tolerance of amides (**5l**, **5m**).

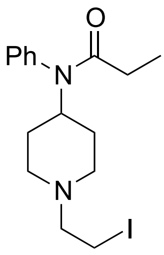

6. Reduction of 1-halo-3-substituted BCPs

2-(Bicyclo[1.1.1]pentan-1-yl)acetamide, 9l

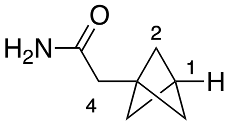

To a solution of **5l** (178 mg, 0.710 mmol, 1.0 equiv.) in MeOH (1.8 mL) at room temperature were added tris(trimethylsilyl)silane (284 μL, 0.920 mmol, 1.3 equiv.) and triethylborane (70 μL, 1.0 M in hexane, 70 μmol, 10 mol %). After stirring for 2 h, the reaction mixture was concentrated and the crude product was washed with pentane (0.5 mL). Purified by column chromatography (SiO_2, EtOAc) afforded **9l** (76 mg, 0.61 mmol, 86%) as a brown solid.

R_f = 0.22 (petroleum ether / Et_2O, 95:5)
m.p. = 142-144 °C
^{1}H NMR (400 MHz, $CDCl_3$) δ 5.66 (br s, 1H, NH), 5.41 (br s, 1H, NH), 2.51 (s, 1H, H1), 2.39 (s, 2H, H4), 1.81 (s, 6H, H2).
^{13}C NMR (101 MHz, $CDCl_3$) δ 173.4, 51.3, 41.5, 40.7, 27.9.
HRMS (CI^+) Found $[M+H]^+$ = 126.0914; $C_7H_{12}NO$ requires 126.0913.
IR (film) ν_{max}/cm^{-1} 3358, 3184, 2963, 1656, 1627, 1432, 1270, 710.

Methyl (*S*)-3-(bicyclo[1.1.1]pentan-1-yl)-2-((tert-butoxycarbonyl)amino)propanoate, 9p

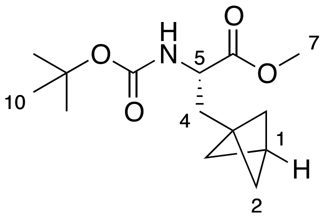

To a solution of iodide **5p** (790 mg, 2.00 mmol, 1.0 equiv.) and 2,6-lutidine (0.700 mL, 6.00 mmol, 3.0 equiv.) in THF (4 mL) was added tris(trimethylsilyl)silane (1.23 mL, 4.00 mmol, 2.0 equiv.) and BEt_3 (200 μL, 1 M in hexane, 200 μmol, 10 mol %). The resulting mixture was stirred for 15 min at room temperature and then concentrated. Purification by column chromatography (SiO_2, petroleum ether / Et_2O, 9:1) and recrystallization from pentane (–20 °C) afforded **9p** (406 mg, 1.51 mmol, 75%) as white crystals.

R_f = 0.23 (petroleum ether / Et_2O, 8:2)
m.p. = 67-68 °C

^{1}H NMR (400 MHz, $CDCl_3$) δ 4.93 (1H, d, *J* = 8.5 Hz, NH), 4.29 (1H, td, *J* = 8.1, 4.5 Hz, H5), 3.72 (3H, s, H7), 2.44, (1H, s, H1), 1.97 (1H, dd, *J* = 14.6, 4.5 Hz, H4), 1.76 (1H, dd, *J* = 14.6, 7.9 Hz, H4), 1.71 (6H, s, H2), 1.44 (9H, s, *t*-Bu).
^{13}C NMR (101 MHz, $CDCl_3$) δ 173.5, 155.2, 79.9, 52.3, 52.3, 51.2, 42.8, 35.0, 28.5, 28.3.
HRMS (ESI$^+$) Found $[M+Na]^+$ = 292.1517; $C_{14}H_{23}O_4N^{23}Na$ requires 292.1519.
IR (film) ν_{max}/cm^{-1} 3352, 2979, 2952, 2905, 2870, 1736, 1689, 1677, 1536, 1246, 1208, 1195, 1156.
$[\alpha]_D^{25}$ −15.1 (c = 3.0, MeOH)

2-(Bicyclo[1.1.1]pentan-1-yl)ethan-1-aminium 2,2,2-trifluoroacetate, 9e

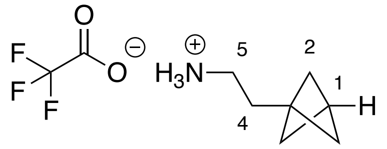

To a solution of **5e** (242 mg, 0.720 mmol, 1.0 equiv.) in methanol (1.8 mL) at room temperature was added tris(trimethylsilyl)silane (288 μL, 0.940 mmol, 1.3 equiv.) and triethylborane (70 μL, 1.0 M in hexane, 70 μmol, 10 mol %). After stirring for 30 min, the reaction mixture was concentrated. The residue dissolved with CH_2Cl_2 (1 mL) and trifluoroacetic acid (1 mL) was added dropwise at room temperature. After stirring for 14 h, the reaction mixture was concentrated *in vacuo* and the crude product was washed with pentane (5 x 0.25 mL). The resulting oil was dried under vacuum at 100 °C for 1 h to afford **9e** (158 mg, 0.70 mmol, 97%) as a thick colourless oil.

^{1}H NMR (400 MHz, CD_3OD) δ 2.89-2.84 (2H, m, H5), 2.48 (1H, s, H1), 1.81-1.78 (2H, m, H4), 1.75 (6H, s, H2).
^{13}C NMR (101 MHz, CD_3OD) δ 162.5 (q, J_{CF} = 35.4 Hz), 117.9 (q, J_{CF} = 291.8 Hz), 51.2, 43.4, 38.5, 31.3, 28.7.
^{19}F NMR (376 MHz, $CDCl_3$) δ_F −77.0.
HRMS (ESI$^+$) Found $[M]^+$ = 112.1119; $C_7H_{14}N$ requires 112.1121.
IR (film) ν_{max}/cm^{-1} 2967, 1668, 1198, 1137, 756, 667.

2-(Bicyclo[1.1.1]pentan-1-yl)acetic acid, 9a

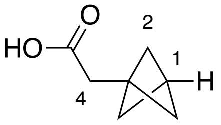

To a solution of **7a** (296 μL, 2.50 mmol, 1.0 equiv.) and TCP (3.44 mL, 0.80 M in Et_2O,

2.75 mmol, 1.1 equiv.) at 0 °C was added BEt_3 (25 μL, 1 M in hexane, 25 μmol, 1 mol %), and the resulting mixture was stirred at 0 °C for 15 min. Tris(trimethylsilyl)silane (1.00 mL, 3.25 mmol, 1.3 equiv.) and BEt_3 (250 μL, 1 M in hexane, 250 μmol, 10 mol %) were added, and the mixture was stirred at room temperature for 15 min (caution: exotherm). Sodium hydroxide (500 mg, 5.0 equiv., 12.5 mmol) in methanol (5 mL) was added (caution: exotherm) and the mixture was refluxed for 30 min, and then concentrated. Water (5 mL) was added and the mixture was extracted with Et_2O (3 × 10 mL). The aqueous phase was acidified to pH 1 with conc. HCl, extracted with Et_2O (3 × 20 mL), and the combined organics were dried ($MgSO_4$) and concentrated. Purification by column chromatography (SiO_2, CH_2Cl_2 / methanol, 95:5) afforded **9a** (204 mg, 1.62 mmol, 64%) as a pungent colourless oil.

R_f = 0.25 (CH_2Cl_2 / methanol, 9:1)
^{1}H NMR (400 MHz, $CDCl_3$) δ 2.51 (2H, s, H4), 2.49 (1H, s, H1), 1.82 (6H, s, H2).
^{13}C NMR (101 MHz, $CDCl_3$) δ 178.1, 51.3, 40.7, 38.3, 28.2.
HRMS (ESI$^-$) Found $[M-H]^-$ = 125.0608; $C_7H_9O_2$ requires 125.0608.
IR (film) ν_{max}/cm^{-1} 2968, 2909, 2874, 1706, 1509, 1407, 1300, 1258, 1229, 1201.

7. Functionalization of 1-halo-3-substituted BCPs

Phenyl(3-(4-(trifluoromethyl)benzyl)bicyclo[1.1.1]pentan-1-yl)methanol, 11

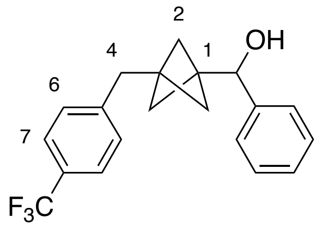

Adapted from the procedure described by Messner et al.[4] To a solution of **5s** (50 mg, 0.14 mmol, 1.0 equiv.) in Et_2O (0.5 mL) at –78 °C was added *tert*-BuLi (0.18 mL, 1.7 M in pentane, 0.31 mmol, 2.2 equiv.) dropwise. The resulting yellow solution was stirred for 1 h at –78 °C, then benzaldehyde (43 μL, 0.43 mmol, 3.0 equiv.) was added at –78 °C. The resulting solution was stirred at –78 °C for 1 h, and then quenched with HCl (1 mL, 1 M aq.,). The aqueous phase was extracted with Et_2O (3 × 3 mL). The combined organics were washed with $NaHCO_3$ (3 mL, aq., sat.), and brine, and then dried ($MgSO_4$) and concentrated *in vacuo*. Purification by column chromatography (SiO_2, pentane / Et_2O, 8:2) afforded the title compound as a white solid (38 mg, 0.114 mmol, 80%).

R_f = 0.13 (pentane / Et_2O, 8:2)

m.p. = 62-63 °C

^{1}H NMR (500 MHz, $CDCl_3$) δ 7.53 (2H, d, *J* = 8.0 Hz, H7), 7.36-7.31 (2H, m, Ar*H*), 7.30-7.26 (1H, m, H14), 7.25-7.22 (2H, m, Ar*H*), 7.18 (2H, d, *J* = 8.0 Hz, H6), 4.70 (1H, s, H10), 2.83 (2H, s, H4), 1.89 (1H, s, O*H*), 1.50 (3H, dd, *J* = 9.6, 1.6 Hz, H2), 1.45 (3H, dd, *J* = 9.6, 1.6 Hz, H2).

^{13}C NMR (126 MHz, $CDCl_3$) δ 143.6, 141.7, 128.3 (q, *J* = 32.2 Hz), 128.2, 127.5, 126.0, 125.3 (q, *J* = 3.8 Hz), 124.5 (q, *J* = 271.8 Hz), 73.9, 48.0, 43.9, 40.2, 39.2.

^{19}F NMR (377 MHz, $CDCl_3$) δ –62.23.

HRMS (ESI^-) Found $[M-H]^-$ = 331.1317; $C_{20}H_{18}OF_3$ requires 331.1315.

IR (film) ν_{max}/cm^{-1} 3406, 2967, 1324, 1255, 1162, 1118, 1066, 1019, 704.

3-(4-(Trifluoromethyl)benzyl)bicyclo[1.1.1]pentane-1-carbaldehyde, 12

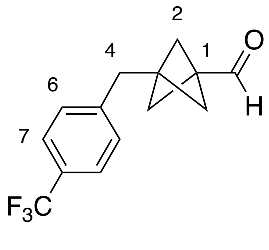

Adapted from the procedure described by Messner et al.[5] To a solution of **5s** (50 mg, 0.14 mmol, 1.0 equiv.) in Et_2O (0.5 mL) at –78 °C was added *tert*-BuLi (0.18 mL, 1.7 M in pentane, 0.31 mmol, 2.2 equiv.) dropwise. The resulting yellow solution was stirred for 1 h at –78 °C, and then added dropwise by syringe into a stirred solution of ethyl formate (34 µL, 0.43 mmol, 3.0 equiv.) in Et_2O (0.5 mL) at –78 °C. The resulting solution was stirred at –78 °C for 1 h, and then quenched with HCl (1 mL, 1 M aq.). The aqueous phase was extracted with Et_2O (3 × 3 mL). The combined organic phases were washed with $NaHCO_3$ (3 mL, aq., sat.) and brine, dried ($MgSO_4$) and concentrated *in vacuo*. Purification by column chromatography (SiO_2, pentane / Et_2O, 8:2) afforded the title compound as a pale yellow oil (27 mg, 0.106 mmol, 74%).

R_f = 0.31 (pentane / Et_2O, 8:2)
^{1}H NMR (500 MHz, $CDCl_3$) δ 9.52 (1H, s, C*H*O), 7.55 (2H, d, *J* = 8.0 Hz, H7), 7.20 (2H, d, *J* = 8.0 Hz, H6), 2.87 (2H, s, H4), 1.86 (6H, s, H2).
^{13}C NMR (126 MHz, CDCl3) δ 198.8, 142.7, 129.3, 128.7 (q, *J* = 32.4 Hz), 125.5 (q, *J* = 3.7 Hz), 124.4 (q, *J* = 271.8 Hz), 50.4, 44.9, 40.9, 38.9.
^{19}F NMR (377 MHz, $CDCl_3$) δ -62.32.
HRMS (EI^+) Found $[M+H]^+$ = 255.0995; $C_{14}H_{14}OF_3$ requires 255.0991.
IR (film) ν_{max}/cm^{-1} 2977, 1711, 1323, 1163, 1116, 1066, 1019.

2-(3-(4-(trifluoromethyl)benzyl)bicyclo[1.1.1]pentan-1-yl)pyridine, 13

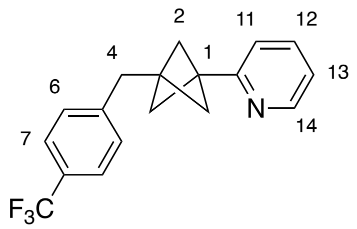

Adapted from the procedure described by Messner et al.[4] To a solution of **5s** (106 mg, 0.300 mmol, 1.0 equiv.) in Et_2O (1 mL) at –78 °C was added *tert*-BuLi (0.45 mL, 1.7 M in pentane, 0.75 mmol, 2.5 equiv.) dropwise. The resulting yellow solution was stirred for 1 h at –78 °C, then $ZnCl_2$ (0.40 mL, 1.9 M in 2-MeTHF, 0.75 mmol, 2.5 equiv.) was added dropwise. The resulting mixture was warmed to 0 °C and stirred for 30 min. In a separate vessel was added Pd(dppf)Cl_2 (11 mg, 15 µmol, 5 mol%), 2-bromopyridine (60 µL, 0.63 mmol, 2.1 equiv.) and THF (1 mL). The resulting suspension was sonicated for 3 min and added quickly to the stirred solution of the organozinc reagent at 0 °C. The solution turned dark green instantly. The vial was heated at 60 °C for 15 h (upon heating, the solution quickly turned olive green,

then yellow). After this time, the reaction mixture was cooled to rt and quenched with NH_4Cl (2 mL, aq., sat.). The aqueous phase was extracted with EtOAc (3 × 5 mL), and the combined organics were washed with NH_4Cl (5 mL, aq., sat.), and brine, then they were dried ($MgSO_4$) and concentrated *in vacuo*. Purification by column chromatography (SiO_2, pentane / EtOAc, 1:0→0:1) afforded the title compound as an orange waxy solid (57 mg, 0.188 mmol, 64%).

R_f = 0.10 (pentane / EtOAc, 9:1)

^{1}H NMR (400 MHz, $CDCl_3$) δ 8.52 (1H, ddd, J = 4.9, 1.8, 1.0 Hz, H14), 7.60-7.53 (3H, m, H7, H12), 7.25 (2H, d, J = 7.6 Hz, H6), 7.14-7.06 (2H, m, H11, H13), 2.93 (2H, s, H4), 1.97 (6H, s, H2).

^{13}C NMR (126 MHz, $CDCl_3$) δ 159.7, 149.4, 143.6, 136.3, 129.4, 128.4 (q, J = 32.3 Hz), 125.3 (q, J = 3.8 Hz), 124.5 (q, J = 271.8 Hz), 121.6, 120.7, 51.9, 43.3, 39.2, 38.9.

^{19}F NMR (377 MHz, $CDCl_3$) δ -62.27.

HRMS (ESI$^+$) Found $[M+H]^+$ = 304.1306; $C_{18}H_{17}NF_3$ requires 304.1308.

IR (film) ν_{max}/cm^{-1} 2969, 1589, 1323, 1268, 1163, 1116, 1066, 1019, 859, 823, 747.

3-(4-(trifluoromethyl)benzyl)bicyclo[1.1.1]pentan-1-ol, 14

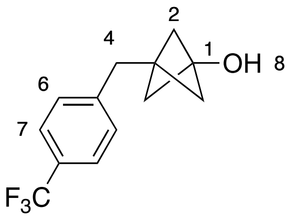

To a solution of **5s** (50 mg, 0.142 mmol, 1.0 equiv.) in PhMe/THF (4:1, 1 mL) at –78°C was added 2-isopropoxy-4,4,5,5-tetramethyl-1,3,2-dioxaborolane (32 µL, 0.156 mmol, 1.1 equiv.) and dropwise *t*BuLi (96 µL, 1.7 M in pentane, 0.163 mmol, 1.15 equiv.). After stirring for 1 h, the reaction mixture was quenched with H_2O (1 mL), extracted three times with Et_2O, dried ($MgSO_4$) and concentrated. The residue dissolved with THF/H_2O (1:1, 2.8 mL) and sodium perborate monohydrate (43 mg, 0.426 mmol, 3 equiv.) was added at room temperature. After stirring for 21 h, the reaction mixture was quenched with H_2O (2 mL), extracted three times with Et_2O, dried ($MgSO_4$) and concentrated. Purification by column chromatography (SiO_2, pentane / Et_2O, 7:3) afforded **14** (21 mg, 0.87 mmol, 61%) as a colourless oil.

R_f = 0.47 (pentane / Et_2O, 1:1)

^{1}H NMR (400 MHz, $CDCl_3$) δ 7.53 (2H, d, J = 7.9 Hz, H7), 7.19 (2H, d, J = 7.9 Hz, H6), 2.91 (2H, s, H4), 2.41 (1H, br s, H8), 1.73 (6H, s, H2).

^{13}C NMR (101 MHz, $CDCl_3$) δ 143.9, 129.3, 128.6 (q, *J* = 32.1 Hz), 125.4 (q, *J* = 3.9 Hz), 124.5 (q, *J* = 271.7 Hz), 63.6, 53.8, 36.4, 31.3.

^{19}F NMR (376 MHz, $CDCl_3$) δ −62.30.

HRMS (EI^+) Found $[M]^+$ = 242.0912; $C_{13}H_{13}F_3O$ requires 242.0913.

IR (film) ν_{max}/cm^{-1} 3278, 2974, 1323, 1246, 1114, 1065, 634.

8. Synthesis and characterization of nucleoside, dipeptide, and pharmaceutical BCP analogues

3′-*O*-(*Tert*-butyldimethylsilyl)-2′-(3-iodobicyclo[1.1.1]pentan-1-yl)-2′-deoxyuridine, 5w *and* 3′-*O*-(*Tert*-butyldimethylsilyl)-2′-(3-iodobicyclo[1.1.1]pentan-1-yl)-2′-deoxyarabinouridine, 5w'

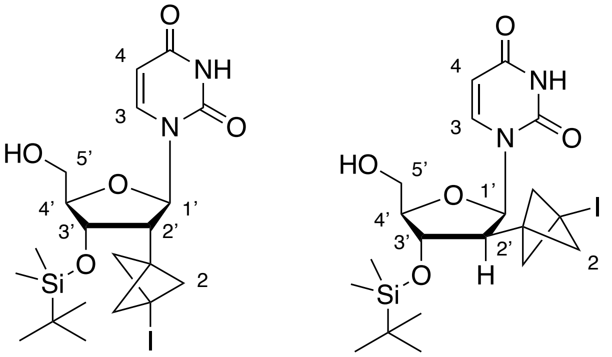

7w (234 mg, 0.500 mmol, 1.0 equiv.), TCP (1.27 mL, 0.79 M in Et_2O, 1.00 mmol, 2.0 equiv.) and BEt_3 (50 μL, 1 M in hexane, 50 μmol, 10 mol %) in MeOH (4 mL) were submitted to **General Procedure 1** at room temperature for 2 h. ^{1}H NMR spectroscopic analysis of the crude material indicated a 5:1 dr of **5w** : **5w'**. Purification by column chromatography (SiO_2, petroleum ether / EtOAc, 1:1→0:1) afforded **5w** (203 mg, 0.380 mmol, 75%) as a white foam and **5w'** (16 mg, 30 μmol, 6%) as a white foam.

Data for **5w**:

R$_f$ = 0.15 (petroleum ether / EtOAc, 1:1)

^{1}H NMR (400 MHz, CD_3OD) δ 7.96 (1H, d, *J* = 8.2 Hz, H3), 6.13 (1H, d, *J* = 8.6 Hz, H1'), 5.69 (1H, d, *J* = 8.2 Hz, H4), 4.45 (1H, dd, *J* = 5.0, 1.7 Hz, H3'), 3.92 (1H, td, *J* = 3.3, 1.7 Hz, H4'), 3.76-3.63 (2H, m, H5'), 2.54 (1H, dd, *J* = 8.6, 4.9 Hz, H2'), 2.30 (3H, dd, *J* = 9.3, 1.5 Hz, H2), 2.24 (3H, dd, *J* = 9.3, 1.6 Hz, H2)., 0.96 (9H, s, Si*t*-Bu), 0.16 (3H, s, SiMe), 0.15 (3H, s, SiMe).

^{13}C NMR (101 MHz, CD_3OD) δ 165.8, 152.2, 142.2, 103.3, 88.9, 87.4, 75.8, 62.6, 61.9, 50.7, 45.6, 26.4, 18.7, 7.6, -3.9, -4.4.

HRMS (ESI$^+$) Found $[M+Na]^+$ = 557.0936; $C_{20}H_{31}O_5N_2{}^{127}I^{28}SiNa$ requires 557.0939.

IR (film) ν_{max}/cm^{-1} 2981, 2887, 1686, 1462, 1383, 1253, 1182, 1091, 946, 832, 775.

$[\alpha]_D^{25}$ −5.6 (c = 1.0, MeOH)

The stereochemistry of C2' was determined by 2D ^{1}H NOESY data. Correlations were observed between H2' and H5', H2' and H3, H2 and H1'.

Data for **5w'**:

R_f = 0.18 (petroleum ether / EtOAc, 1:1), [UV, vanillin]

^{1}H NMR (400 MHz, CD_3OD) δ 7.94 (1H, d, *J* = 8.1 Hz, H3), 5.99 (1H, d, *J* = 6.7 Hz, H1'), 5.75 (1H, d, *J* = 8.1 Hz, H4), 4.33 (1H, t, *J* = 5.5 Hz, H3'), 3.87 (1H, dd, *J* = 13.6, 4.1 Hz, H4'), 3.82-3.74 (2H, m, H5'), 2.78 (1H, dd, *J* = 6.7, 5.4 Hz, H2'), 2.22-2.11 (6H, m, H2), 0.91 (9H, s, Si*t*-Bu), 0.16 (6H, s, $SiMe_2$).

^{13}C NMR (101 MHz, CD_3OD) δ 166.0, 152.0, 143.3, 102.1, 87.2, 86.8, 74.2, 61.7, 61.0, 53.9, 47.0, 26.3, 18.8, 6.9, -3.7, -4.0.

HRMS (ESI^+) Found $[M+H]^+$ = 535.1119; $C_{20}H_{32}O_5N_2{}^{127}I^{28}Si$ requires 535.1120.

IR (film) ν_{max}/cm^{-1} 3400, 3000, 2954, 2928, 2857, 1686, 1464, 1187, 1139, 1086, 838, 777.

$[\boldsymbol{\alpha}]_{\boldsymbol{D}}^{\boldsymbol{25}}$ 64.2 (c = 1.0, MeOH).

The stereochemistry of C2' was determined by 2D ^{1}H NOESY data. Correlations were observed between H2 and H3.

3′-*O*-(*Tert*-butyldimethylsilyl)-2′-(bicyclo[1.1.1]pentan-1-yl)-2′-deoxyuridine, 9w

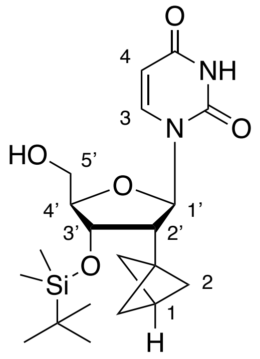

To a solution of **5w** (18.2 mg, 0.034 mmol, 1.0 equiv.) in MeOH/Et_2O (1:1, 1 mL) was added tris(trimethylsilyl)silane (21 μL, 68 μmol, 2.0 equiv.), then BEt_3 (3.4 μL, 3.4 μmol, 10 mol %) was added via syringe into the solution. The resulting mixture was stirred for 15 min at room temperature, and then concentrated. Purification by column chromatography (SiO_2, petroleum ether / EtOAc, 1:1 → 0:1) afforded **9w** (10.2 mg, 0.025 mmol, 73%) as an off-white solid.

R_f = 0.15 (petroleum ether / EtOAc, 1:1)

m.p. = 180 °C (decomp.)

^{1}H NMR (400 MHz, MeOD) δ 7.91 (1H, d, *J* = 8.1 Hz, H3), 6.14 (1H, d, *J* = 8.9 Hz, H1'), 5.68 (1H, d, *J* = 8.1 Hz, H4), 4.41 (1H, dd, *J* = 4.9, 1.6 Hz, H3'), 3.87 (1H, td, *J* = 3.6, 1.6 Hz, H4'), 3.64 (2H, dd, *J* = 3.6, 1.2 Hz, H5'), 2.40 (1H, s, H1), 2.34 (1H, dd, *J* = 8.9, 4.9 Hz, H2'), 1.81 (3H, dd, *J* = 9.5, 1.6 Hz, H2), 1.76 (3H, dd, *J* = 9.5, 1.6 Hz, H2), 0.92 (9H, s, Si*t*-Bu), 0.11 (3H, s, SiMe), 0.10 (3H, s, SiMe).
^{13}C NMR (101 MHz, MeOD) δ 165.9, 152.3, 142.5, 103.1, 89.1, 87.8, 76.1, 62.9, 52.1, 50.5, 42.5, 30.6, 26.4, 18.7, -3.9, -4.3.
HRMS (ESI$^+$) Found $[M+Na]^+$ = 431.1971; $C_{20}H_{32}O_5N_2{}^{23}Na^{28}Si$ requires 431.1973.
IR (film) ν_{max}/cm^{-1} 3412, 2960, 2928, 2858, 1688, 1464, 1259, 1203, 1059, 834, 811, 777.
$[\alpha]_D^{25}$ 24.3 (c = 0.86, MeOH)

Boc-Asp(OtBu)-β-(3-iodobicyclo[1.1.1]pentan-1-yl)Ala-OMe, 5x

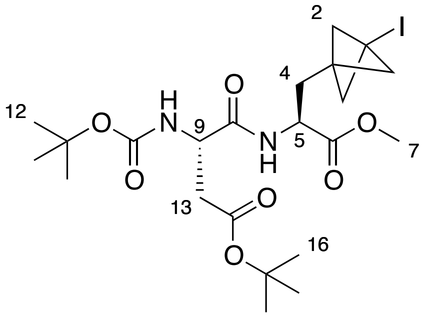

Adapted from the procedure described by Koseki et al.[6] To a solution of **15** (570 mg, 1.46 mmol, 1.0 equiv.) in CH_2Cl_2 (10 mL) was added imidazole (149 mg, 2.19 mmol, 1.5 equiv.) and PPh_3 (574 mg, 2.19 mmol, 1.5 equiv.). The resulting solution was cooled to 0 °C and iodine (556 mg, 2.19 mmol, 1.5 equiv.) was added in five portions. The resulting mixture was stirred at 0 °C for 1 h, and then diluted with water (10 mL). The phases were separated, and the aqueous phase was extracted with CH_2Cl_2 (2 × 15 mL). The combined organic phases were washed with $Na_2S_2O_3$ (aq., 10 wt%, 20 mL), dried (Na_2SO_4) and concentrated. The resulting solid was washed with pentane (2 × 10 mL) and Et_2O (2 × 10 mL), and the combined filtrates were concentrated to afforded a 0.41:1 mixture of Ph_3PO and iodinated dipeptide as an orange oil (386 mg, ~52%), which was used without further purification.
The resulting oil (386 mg, ~771 μmol, 1.0 equiv.), TCP (1.93 mL, 0.8 M in Et_2O, 1.54 mmol, 2.0 equiv.) and BEt_3 (77 μL, 1 M in hexane, 77 μmol, 10 mol %) were submitted to **General Procedure 1** for 1 h at room temperature. Purification by column chromatography (SiO_2, petroleum ether / EtOAc, 1:0→7:3) afforded **5x** (312 mg, 0.55 mmol, 38% over two steps) as a white solid.

R$_f$ = 0.19 (petroleum ether/EtOAc, 8:2)

m.p. = 105-106 °C

^{1}H NMR (400 MHz, $CDCl_3$) δ 7.07 (1H, d, *J* = 7.9 Hz, NH), 5.73 (1H, d, *J* = 8.5 Hz, NH), 4.53 (td, *J* = 7.5, 4.3 Hz, H5), 4.45 (q, *J* = 5.5 Hz, H9), 3.71 (3H, s, H7), 2.88 (1H, dd, *J* = 17.1, 4.5 Hz, H13), 2.57 (1H, dd, *J* = 17.1, 6.3 Hz, H13), 2.22 (6H, s, H2), 2.12 (1H, dd, *J* = 14.8, 4.3 Hz, H4), 1.93 (1H, dd, *J* = 14.7, 7.3 Hz, H4), 1.47 (9H, s, *t*-Bu), 1.45 (9H, s, *t*-Bu).

^{13}C NMR (101 MHz, $CDCl_3$) δ 171.9, 171.5, 170.8, 155.7, 82.0, 80.7, 61.0, 52.6, 50.9, 50.7, 45.3, 37.0, 34.0, 28.5, 28.2, 6.3.

HRMS (ESI^+) Found $[M+H]^+$ = 567.1544; $C_{22}H_{36}O_7N_2{}^{127}I$ requires 567.1562.

IR (film) ν_{max}/cm^{-1} 3320, 2978, 2919, 1721, 1666, 1520, 1437, 1392, 1367, 1295, 1248, 1179, 1158, 1049, 1024, 842, 734

$[\alpha]_D^{25}$ = 29.3 (c 1.0, $CHCl_3$).

Boc-Asp(OtBu)-β-(bicyclo[1.1.1]pentan-1-yl)Ala-OMe, 9x

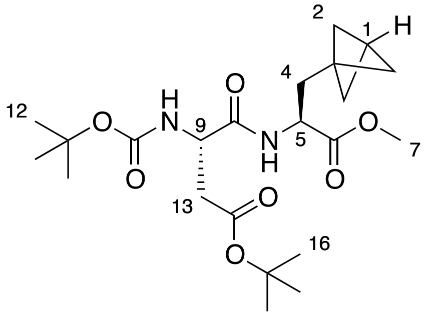

To a solution of **5x** (56.6 mg, 100 μmol, 1.0 equiv.) in THF (0.2 mL) was added tris(trimethylsilyl)silane (62 μmol, 0.20 mmol, 2.0 equiv.). BEt_3 (10 μL, 1 M in hexane, 10 μmol, 10 mol %) was added and the resulting mixture was stirred at room temperature for 15 min. Further triethylborane (2 x 10 mol %) was added at 15 minute intervals until the reaction was judged complete by TLC. The reaction mixture was then diluted with Et_2O (5 mL), and the organic phase was washed with $NaHCO_3$ (aq., sat., 5 mL). The aqueous phase was extracted with Et_2O (2 × 5 mL) and the combined organics were washed with brine (10 mL), dried (Na_2SO_4) and concentrated. Purification by column chromatography (SiO_2, petroleum ether / EtOAc, 9:1→8:2) afforded **9x** (36.5 mg, 83 μmol, 83%) as a colourless oil.

R$_f$ = 0.22 (petroleum ether / EtOAc, 8:2)

^{1}H NMR (400 MHz, $CDCl_3$) δ 7.00 (1H, d, *J* = 6.8 Hz, NH), 5.72 (1H, d, *J* = 7.4 Hz, NH), 4.51 (2H, m, H5, H9), 3.70 (3H, s, H7), 2.85 (1H, dd, *J* = 17.0, 4.6 Hz, H13), 2.58 (1H, dd, *J*

= 17.0, 6.6 Hz, H13), 2.43 (1H, s, H1), 1.97 (1H, dd, J = 14.7, 4.5 Hz, H4), 1.84 (1H, dd, J = 14.7, 7.3 Hz, H4), 1.71 (6H, s, H2), 1.45 (9H, s, t-Bu), 1.44 (9H, s, t-Bu).
^{13}C NMR (101 MHz, $CDCl_3$) δ 172.4, 171.5, 170.7, 155.6, 81.8, 80.4, 52.4, 51.2, 50.7, 42.6, 37.4, 34.4, 29.8, 28.5, 28.2, 24.0.
HRMS (ESI$^+$) Found $[M+H]^+$ = 441.2593; $C_{22}H_{37}O_7N_2$ requires 441.2595.
IR (film) ν_{max}/cm^{-1} 3324, 2970, 2908, 2871, 1721, 1668, 1520, 1456, 1437, 1392, 1367, 1281, 1248, 1202, 1155, 1049, 1025, 847, 777.
$[\alpha]_D^{25}$ = 12.9 (c 1.0, $CHCl_3$)

2-(3-Iodobicyclo[1.1.1]pentan-1-yl)ethyl 4-methylbenzenesulfonate, 5y

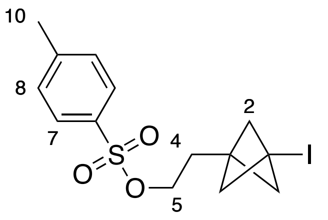

7y (1.10 g, 3.37 mmol, 1.0 equiv.), TCP (5.48 mL, 0.8 M in Et_2O, 4.38 mmol, 1.3 equiv.) and BEt_3 (337 μL, 1 M in hexane, 337 μmol, 10 mol %) were submitted to **General Procedure 1** at room temperature for 3 h. Purification by column chromatography (SiO_2, petroleum ether / Et_2O, 1:0→1:1) afforded **5y** (1.23 g, 3.14 mmol, 92%) as a white solid.

R_f = 0.20 (petroleum ether/Et_2O, 9:1)
m.p. = 66-67 °C
^{1}H NMR (400 MHz, $CDCl_3$) δ 7.78 (2H, d, J = 8.0 Hz, H7), 7.36 (2H, d, J = 8.0 Hz, H8), 4.00 (2H, t, J = 6.2 Hz, H5), 2.46 (3H, s, H10), 2.19 (6H, s, H2), 1.88 (2H, t, J = 6.2 Hz, H4).
^{13}C NMR (101 MHz, $CDCl_3$) δ 145.1, 133.0, 130.1, 128.0, 68.0, 60.7, 45.4, 31.2, 21.8, 6.4.
HRMS (ESI$^+$) Found $[M+Na]^+$ = 414.9835; $C_{14}H_{17}O_3{}^{127}I^{23}Na^{32}S$ requires 414.9835.
IR (film) ν_{max}/cm^{-1} 2989, 2915, 1598, 1448, 1360, 1307, 1176, 1097, 1022, 963, 936, 896, 837, 816, 778, 733, 663.

2-(Bicyclo[1.1.1]pentan-1-yl)ethyl 4-methylbenzenesulfonate, 9y

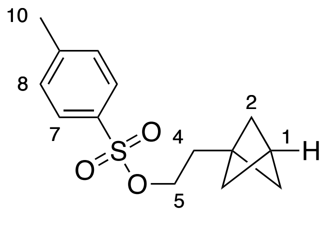

To a solution of **5y** (1.23 g, 3.14 mmol, 1.0 equiv.) in THF (6 mL) were added tris(trimethylsilyl)silane (1.93 mL, 6.28 mmol, 2.0 equiv.) and then BEt_3 (314 μL, 1 M in hexane, 314 μmol, 10 mol %, syringed inside the reaction mixture). After stirring for 15 min, Et_2O (20 mL) was added, the organic phase was washed with $NaHCO_3$ (aq., sat., 15 mL) and the phases were separated. The aqueous was extracted with Et_2O (2 × 5 mL) and the combined organic phases were washed with brine (20 mL), dried (Na_2SO_4) and concentrated. Purification by column chromatography (SiO_2, petroleum ether / Et_2O, 100:0→95:5) afforded **9y** (1.01 g, 3.79 mmol, 64%) as a colourless oil.

$\mathbf{R_f}$ = 0.36 (petroleum ether/Et_2O, 9:1)
^{1}H NMR (400 MHz, $CDCl_3$) δ 7.78 (2H, d, *J* = 7.9 Hz, H8), 7.34 (2H, d, *J* = 7.9 Hz, H7), 4.01 (2H, t, *J* = 6.7 Hz, H5), 2.44 (3H, s, H10), 2.41 (1H, s, H1), 1.76 (2H, t, *J* = 6.7 Hz, H4), 1.64 (6H, s, H2).
^{13}C NMR (101 MHz, $CDCl_3$) δ 144.8, 133.2, 129.9, 128.0, 68.9, 50.8, 42.4, 31.6, 28.1, 21.8.
HRMS (ESI$^+$) Found $[M+Na]^+$ = 289.0867; $C_{14}H_{18}O_3{}^{23}Na^{32}S$ requires 289.0869.
IR (film) ν_{max}/cm^{-1} 2964, 2908, 2870, 1598, 1358, 1189, 1176, 975, 941, 921.

***N*-(1-(2-(bicyclo[1.1.1]pentan-1-yl)ethyl)piperidin-4-yl)-*N*-phenylpropionamide (BCP-fentanyl), 17**

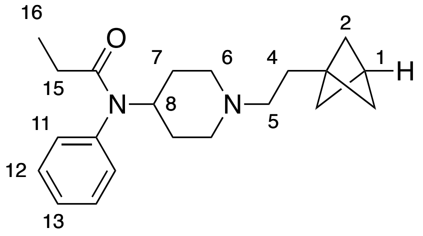

To a vial containing **9y** (93 mg, 0.35 mmol, 1.0 equiv.) was added a solution of norfentanyl **18** (89 mg, 0.35 mmol, 1.1 equiv.) in CH_3CN (1.5 mL). K_2CO_3 (96 mg, 0.70 mmol, 2.0 equiv.) was added and the resulting mixture was refluxed for 24 h. The mixture was cooled to room temperature and concentrated. The residue was dissolved in CH_2Cl_2 (5 mL) and water (5 mL), and the phases were separated. The aqueous phase was extracted with CH_2Cl_2 (3 × 5 mL) and the combined organic phases were dried (Na_2SO_4) and concentrated. Purification by column chromatography (SiO_2, CH_2Cl_2 / CH_3OH, 8:2) afforded **17** as a pale yellow solid (64 mg, 0.20 mmol, 53%). Recrystallization form hot petroleum ether afforded pale yellow crystals that were suitable for X-ray diffraction (see Section 8, p 52).

$\mathbf{R_f}$ = 0.23 (CH_2Cl_2/EtOAc, 1:1)

m.p. = 84-85 °C

^{1}H NMR (400 MHz, $CDCl_3$) δ 7.42-7.28 (3H, m, H13, H12), 7.05 (2H, dd, *J* = 7.8, 1.7 Hz, H11), 4.64 (1H, tt, *J* = 12.2, 4.0 Hz, H8), 2.88 (2H, dt, *J* = 12.4, 3.1 Hz, H6), 2.41 (1H, s, H1), 2.27-2.17 (2H, m, H5), 2.08-1.97 (2H, m, H6), 1.91 (2H, q, *J* = 7.4 Hz, H15), 1.81-1.70 (2H, m, 2 × H7), 1.60 (6H, s, H2), 1.55-1.46 (2H, m, H4), 1.37 (2H, qd, *J* = 12.4, 4.0 Hz, H7), 1.00 (3H, t, *J* = 7.4 Hz, H16).

^{13}C NMR (101 MHz, $CDCl_3$) δ 173.6, 139.0, 130.5, 129.4, 128.3, 55.9, 53.2, 52.3, 50.4, 44.0, 30.7, 30.0, 28.6, 27.8, 9.7.

HRMS (ESI^+) Found $[M+H]^+$ = 327.2425; $C_{21}H_{31}ON_2$ requires 327.2431.

IR (film) ν_{max}/cm^{-1} 2959, 2867, 2766, 2360, 1659, 1596, 1495, 1450, 1375, 1340, 1260, 1194, 1093, 1056, 1018, 776, 746, 705.

9. Synthesis and characterisation of substrates

Ethyl iodoacetate, 7a

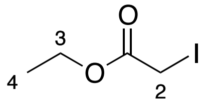

Ethyl bromoacetate (330 µL, 3.00 mmol, 1.0 equiv.) and sodium iodide (673 mg, 4.50 mmol, 1.5 equiv.) in acetone (10 mL) were submitted to **General Procedure 2** (12 h), which afforded **7a** (568 mg, 2.65 mmol, 88%) as a yellow oil.

R$_f$ = 0.46 (Petroleum ether / EtOAc, 9:1), [UV].
^{1}H NMR (400 MHz, $CDCl_3$) δ 4.19 (2H, q, *J* = 7.1 Hz, H3), 3.67 (2H, s, H2), 1.27 (3H, t, *J* = 7.1 Hz, H4).
^{13}C NMR (101 MHz, $CDCl_3$) δ 168.9, 62.2, 14.0, −5.1.
HRMS (CI^+) Found $[M+NH_4]^+$ = 231.9833; $C_4H_{11}IO_2N$ requires 231.9828.
Spectroscopic data in agreement with that reported previously.[7]

1-Iodo-2-phenylethane, 7c

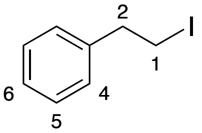

2-phenylethanol (490 µL, 4.09 mmol, 1.0 equiv.), triphenylphosphine (1.40 g, 5.32 mmol, 1.3 equiv.) iodine (1.45 g, 5.73 mmol, 1.4 equiv.) and imidazole (418 mg, 6.14 mmol, 1.5 equiv.) in CH_3CN (4 mL) and Et_2O (5 mL) were submitted to **General Procedure 3** (12 h). Purification by column chromatography (SiO_2, petroleum ether / EtOAc, 9:1) afforded **7c** (881 mg, 3.79 mmol, 93%) as a yellowish oil.

R$_f$ = 0.90 (petroleum ether / EtOAc, 9:1), [UV].
^{1}H NMR (400 MHz, $CDCl_3$) δ 7.28-7.22 (2H, m, H5), 7.22-7.16 (1H, m, H6), 7.14-7.10 (2H, m, H4), 3.28 (2H, t, *J* = 8.0 Hz, H2), 3.11 (2H, t, *J* = 8.0 Hz, H1).
^{13}C NMR (101 MHz, $CDCl_3$) δ 140.7, 128.8, 128.5, 127.0, 40.5, 5.8.
HRMS (CI^+) Found $[M+NH_4]^+$ = 250.0090; $C_8H_{13}IN$ requires 250.0087.
Spectroscopic data in agreement with that reported previously.[8]

tert-Butyl (2-iodoethyl)carbamate, 7e

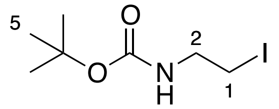

To a solution of 2-aminoethanol (1.00 mL, 16.3 mmol, 1.0 equiv.) in CH_2Cl_2 (20 mL) at room temperature was added dropwise a solution of di-*tert*-butyl dicarbonate (3.90 g, 18.0 mmol, 1.1 equiv.) in CH_2Cl_2 (5 mL). After stirring for 3 h, the reaction was quenched with $NaHCO_3$ (25 mL, aq., sat.). The organic layer was separated, dried ($MgSO_4$) and concentrated. The resulting residue, triphenylphosphine (5.10 g, 19.4 mmol, 1.2 equiv.) and iodine (4.90 g, 19.4 mmol, 1.2 equiv.) in CH_2Cl_2 (25 mL) were submitted to **General Procedure 3** (3 h). Purification by column chromatography (SiO_2, petroleum ether / Et_2O, 97:3→90:10) afforded **7e** (1.49 g, 5.50 mmol, 34%) as a pale yellow solid.

$\mathbf{R_f}$ = 0.62 (petroleum ether / Et_2O, 2:1)
m.p. = 38-40 °C
^{1}H NMR (400 MHz, $CDCl_3$) δ_H 4.95 (1H, br s, NH), 3.51-3.37 (2H, m, H2), 3.22 (2H, t, J = 6.5 Hz, H1), 1.43 (9H, s, *t*-Bu).
^{13}C NMR (101 MHz, $CDCl_3$) δ_C 155.6, 79.9, 43.1, 28.5, 6.1.
HRMS (ESI^+) Found $[M+H]^+$ = 272.0151; $C_7H_{15}INO_2$ requires 247.0147.
IR (film) ν_{max}/cm^{-1} 3340, 1687, 1506, 1249, 1160.
Spectroscopic data in agreement with that reported previously.[9]

2-Iodo-1-phenylethan-1-one, 7f

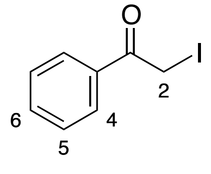

2-bromo-1-phenylethan-1-one (199 mg, 1.00 mmol, 1.0 equiv), sodium iodide (225 mg, 1.50 mmol, 1.5 equiv.) in acetone (1 mL) were submitted to **General Procedure 2** (18 h) to afford **7f** (227 mg, 0.93 mmol, 93%) as a yellow oil.
^{1}H NMR (400 MHz, $CDCl_3$) δ_H 8.02-7.97 (2H, m, H4), 7.64-7.57 (1H, m, H6), 7.52-7.46 (2H, m, H5), 4.37 (2H, s, H2)
^{13}C NMR (101 MHz, $CDCl_3$) δ_C 193.0, 133.9, 133.6, 129.1, 128.9, 1.9
HRMS (EI^+) Found $[M]^+$ = 245.9541; C_8H_7IO requires 245.9536.

IR (film) ν_{max}/cm^{-1} 1670, 1269, 984, 700.

Spectroscopic data in agreement with that reported previously. [10]

1-(Furan-2-yl)-2-iodoethan-1-one, 7g

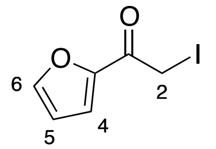

2-acetylfuran (300 μL, 3.00 mmol, 1.0 equiv.), CuO (240 mg, 3.00 mmol, 1.0 equiv.) and I_2 (762 mg, 3.00 mmol, 1.0 equiv.) in MeOH (12 mL) were submitted to **General Procedure 4**. Purification by column chromatography (SiO_2, petroleum ether / Et_2O, 8:2) afforded **7g** (605 mg, 2.56 mmol, 85%) as a brown oil.

$\mathbf{R_f}$ = 0.36 (petroleum ether / Et_2O, 7:3)

^{1}H NMR (400 MHz, $CDCl_3$) δ_H 7.62 (1H, dd, J = 1.7, 0.8 Hz, H6), 7.31 (1H, dd, J = 3.6, 0.8 Hz, H4), 6.58 (1H, dd, J = 3.6, 1.7 Hz, H5), 4.23 (2H, s, H2).

^{13}C NMR (101 MHz, $CDCl_3$) δ_C 182.2, 150.0, 147.1, 119.0, 113.0, 0.7.

HRMS (EI^+) Found $[M]^+$ = 235.9323; $C_6H_5IO_2$ requires 235.9329.

IR (film) ν_{max}/cm^{-1} 2980, 1660, 1461, 1292, 762.

2-Iodo-1-(1H-pyrrol-2-yl)ethan-1-one, 7h

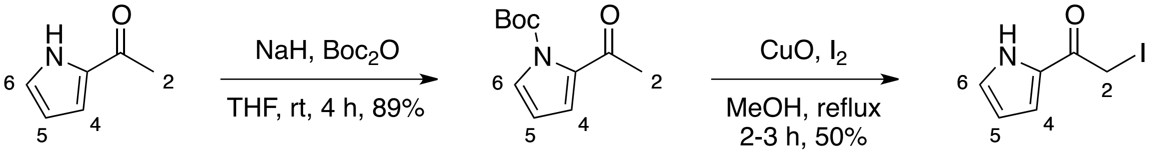

S1 **7h**

To a solution of 2-acetylpyrrole (500 mg, 4.58 mmol, 1.0 equiv.) and di-*tert*-butyl dicarbonate (1.10 g, 5.04 mmol, 1.1 equiv.) in THF (60 mL) at room temperature, was added NaH (350 mg, 8.75 mmol, 1.9 equiv.) as a suspension in THF (40 mL) dropwise. After stirring for 4 h, the suspension was diluted with EtOAc (100 mL) and the reaction was quenched with NH_4Cl (aq., sat., 30 mL). The organic layer was washed with NH_4Cl (aq., sat., 3 × 30 mL), dried ($MgSO_4$) and concentrated. Purification by column chromatography (SiO_2, petroleum ether / Et_2O, 7:3) afforded *tert*-butyl 2-acetyl-1H-pyrrole-1-carboxylate **S1** (848 mg, 4.05 mmol, 89%) as a colourless oil.

S1 (209 mg, 1.00 mmol, 1.0 equiv.), CuO (80 mg, 1.0 mmol, 1.0 equiv.) and I_2 (254 mg, 1.00 mmol, 1.0 equiv.) in MeOH (4 mL) were submitted to **General Procedure 4**.

Purification by column chromatography (SiO_2, petroleum ether / Et_2O, 9:1 to 7:3) afforded **7h** (118 mg, 0.500 mmol, 50%) as a yellow solid.

Data for **S1:**

R_f = 0.49 (petroleum ether / Et_2O, 7:3)

^{1}H NMR (400 MHz, $CDCl_3$) δ_H 7.31 (1H, ddd, J = 3.0, 1.6, 0.7 Hz, H6), 6.85 (1H, ddd, J = 3.6, 1.6, 0.7 Hz, H4), 6.16 (1H, ddd, J = 3.6, 3.0, 0.7 Hz, H5), 2.44 (3H, s, H2), 1.57 (9H, s, *t*-Bu).

^{13}C NMR (101 MHz, $CDCl_3$) δ_C 188.6, 149.1, 134.3, 128.1, 121.3, 110.1, 85.0, 28.1, 27.7.

Spectroscopic data in agreement with that reported previously.[11]

Data for **7h**:

R_f = 0.22 (petroleum ether / Et_2O, 7:3)

m.p. = 122-124 °C

^{1}H NMR (400 MHz, $CDCl_3$) δ_H 9.82 (1H, br s, NH), 7.10 (1H, td, J = 2.5, 1.3 Hz, H6), 7.02 (ddd, J = 3.9, 2.5, 1.3 Hz, H4), 6.32 (1H, dt, J = 3.9, 2.5 Hz, H5), 4.21 (2H, s, H2).

^{13}C NMR (101 MHz, $CDCl_3$) δ_C 183.5, 128.6, 126.6, 118.0, 111.4, 1.0.

HRMS (CI^+) Found $[M+H]^+$ = 235.9564; C_6H_7INO requires 235.9567.

IR (film) ν_{max}/cm^{-1} 3256, 1624, 1395, 1050, 747.

2-Iodo-1-(thiophen-2-yl)ethan-1-one, 7i

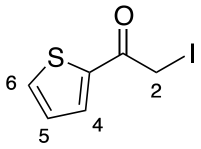

2-bromo-1-(thiophen-2-yl)ethan-1-one (205 mg, 1.00 mmol, 1.0 equiv.), sodium iodide (225 mg, 1.50 mmol, 1.5 equiv.) in acetone (1 mL) were submitted to **General Procedure 2** (18 h) to afford **7i** (226 mg, 0.90 mmol, 90%) as a brown oil.

^{1}H NMR (400 MHz, $CDCl_3$) δ_H 7.80 (1H, dd, J = 3.9, 1.1 Hz, H6), 7.69 (1H, dd, J = 5.0, 1.1 Hz, H4), 7.16 (1H, dd, J = 5.0, 3.9 Hz, H5), 4.30 (3H, s, H2).

^{13}C NMR (101 MHz, $CDCl_3$) δ_C 186.1, 140.5, 135.2, 133.6, 128.5, 1.5.

HRMS (EI^+) Found $[M]^+$ = 251.9099; C_6H_5IOS requires 251.9100.

IR (film) ν_{max}/cm^{-1} 1645, 1409, 1278, 721.

Spectroscopic data in agreement with that reported previously.[10]

1-(Benzofuran-2-yl)-2-iodoethan-1-one, 7j

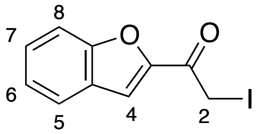

1-(benzofuran-2-yl)-2-bromoethan-1-one (239 mg, 1.0 mmol, 1.0 equiv.), sodium iodide (225 mg, 1.5 mmol, 1.5 equiv.) in acetone (1 mL) were submitted to **General Procedure 2** (18 h) to afford **7j** (207 mg, 0.72 mmol, 72%) as a pale yellow solid.

m.p. = 103-105 °C

^{1}H NMR (400 MHz, $CDCl_3$) δ_H 7.72 (1H, dt, J = 8.0, 1.0 Hz, H5), 7.64 (1H, d, J = 1.0 Hz, H4), 7.59 (1H, dq, J = 8.4, 1.0 Hz, H8), 7.51 (1H, ddd, J = 8.4, 7.1, 1.0 Hz, H7), 7.33 (1H, ddd, J = 8.0, 7.1, 1.0 Hz, H6), 4.36 (2H, s, H2).

^{13}C NMR (101 MHz, $CDCl_3$) δ_C 184.1, 155.9, 149.9, 129.0, 127.2, 124.3, 123.6, 114.7, 112.7, 0.8.

HRMS (EI^+) Found $[M]^+$ = 285.9190; $C_{10}H_7IO_2$ requires 285.9485.

IR (film) ν_{max}/cm^{-1} 1658, 1552, 1299, 1022, 747.

Spectroscopic data in agreement with that reported previously.[12]

***tert*-Butyl 3-(2-iodoacetyl)-1H-indole-1-carboxylate, 7k**

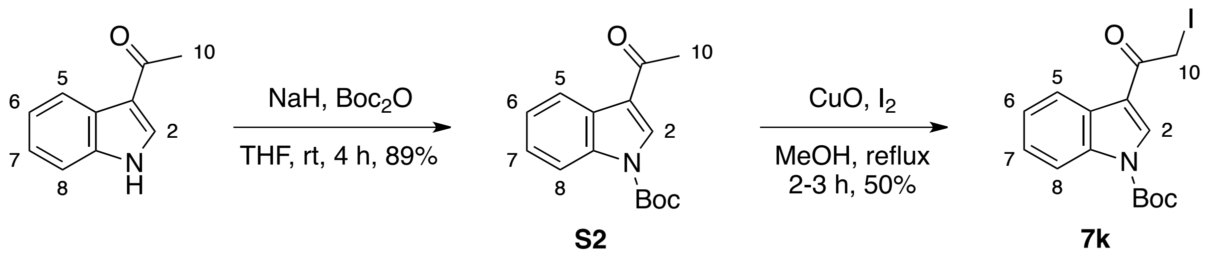

To a solution of 3-acetylindole (729 mg, 4.58 mmol, 1.0 equiv.) and di-*tert*-butyl dicarbonate (1.10 g, 5.04 mmol, 1.1 equiv.) in THF (60 mL) at room temperature was added a suspension of NaH (350 mg, 8.75 mmol, 1.9 equiv.) in THF (40 mL) dropwise. After stirring for 3 h, the suspension was diluted with EtOAc (100 mL) and quenched with NH_4Cl (aq., sat., 30 mL). The organic phase was washed with NH_4Cl (aq., sat., 3 × 30 mL), dried ($MgSO_4$) and concentrated. Purification by column chromatography (SiO_2, petroleum ether / Et_2O, 7:3) afforded *tert*-Butyl 3-acetyl-1H-indole-1-carboxylate **S2** (982 mg, 3.79 mmol, 83%) as a white solid.

S2 (259 mg, 1.00 mmol, 1.0 equiv.), CuO (80 mg, 1.0 mmol, 1.0 equiv.) and I_2 (254 mg, 1.00 mmol, 1.0 equiv.) in MeOH (4 mL) were submitted to **General Procedure 4**. Purification by column chromatography (SiO_2, petroleum ether / Et_2O, 7:3 to 1:1) afforded **7k** (247 mg, 0.64 mmol, 64%) as a yellow oil.

Data for **S2:**
R$_f$ = 0.44 (petroleum ether / Et_2O, 7:3)
m.p. = 142–144 °C
^{1}H NMR (400 MHz, $CDCl_3$) δ_H 8.40-8.35 (1H, m, H8), 8.23 (1H, s, H2), 8.15-8.08 (1H, m, H5), 7.42-7.32 (2H, m, H7, H6), 2.57 (3H, s, H2), 1.72 (9H, s, *t*-Bu).
^{13}C NMR (101 MHz, $CDCl_3$) δ_C 194.0, 149.3, 135.7, 132.5, 127.5, 125.6, 124.5, 122.8, 120.8, 115.1, 85.5, 28.3, 27.9.
HRMS (CI^+) Found $[M+H]^+$ = 260.1290; $C_{15}H_{18}NO_3$ requires 260.1281.
IR (film) ν_{max}/cm^{-1} 1656, 1169, 749.

Data for **7k:**
R$_f$ = 0.40 (petroleum ether / Et_2O, 95:5)
m.p. = 140-142 °C
^{1}H NMR (400 MHz, $CDCl_3$) δ_H 8.35-8.31 (2H, m, H2, H8), 8.15-8.07 (1H, m, H5), 7.44-7.33 (2H, m, H7, H6), 4.30 (2H, s, H2), 1.72 (9H, s, *t*-Bu).
^{13}C NMR (101 MHz, $CDCl_3$) δ_C 188.9, 149.1, 135.7, 132.9, 127.6, 126.0, 124.8, 122.9, 116.9, 115.2, 86.0, 28.3, 3.1.
HRMS (CI^+) Found $[M+H]^+$ = 386.0233; $C_{15}H_{17}INO_3$ requires 386.0248.
IR (film) ν_{max}/cm^{-1} 1730, 1653, 1367, 1157, 748.

2-Iodopropanamide, 7m

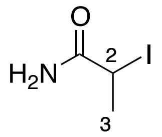

To a solution of sodium iodide (450 mg, 3.00 mmol, 1.5 equiv.) in acetone (2 mL) at room temperature was added 2-bromopropionamide (304 mg, 2.00 mmol, 1.0 equiv.). After stirring for 17 h, the reaction mixture was concentrated and the residue taken up in 1:1 H_2O / EtOAc (10 mL). The resulting layers were separated and the aqueous phase was extracted with EtOAc (3 x 5 mL). The combined organic layers were washed with $Na_2S_2O_3$ (10% aq., 10

mL), dried ($MgSO_4$) and concentrated afforded **7m** (307 mg, 1.54 mmol, 77%) as a white solid, which required no further purification.

m.p. = 150-152 °C
^{1}H NMR (400 MHz, CD_3OD) δ_H 4.57 (1H, q, J = 7.0 Hz, H2), 1.87 (3H, d, J = 7.0 Hz, H3).
^{13}C NMR (101 MHz, CD_3OD) δ_C 176.8, 24.2, 16.6.
HRMS (CI^+) Found $[M+H]^+$ = 199.9567; C_3H_7INO requires 199.9567.
IR (film) ν_{max}/cm^{-1} 3335, 3165, 1686, 1410, 1060, 626.

((Iodomethyl)sulfonyl)benzene, 7n

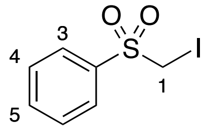

According to the procedure of Pospisil et al.[8] To a suspension of sodium benzenesulfinate (821 mg, 5.00 mmol, 1.0 equiv.) in DMF (10 mL) was added diiodomethane (484 μL, 6.00 mmol, 1.2 equiv.) dropwise. The resulting mixture was stirred at room temperature for 17 h. Brine (100 mL) was added and the aqueous phase was extracted with EtOAc (3 × 20 mL). The combined organic phases were washed with brine (50 mL), dried ($MgSO_4$) and concentrated *in vacuo*. Purification by column chromatography (SiO_2, petroleum ether / EtOAc, 10:1→2:1) afforded **7n** (893 mg, 3.17 mmol, 63%) as a yellow solid.

R_f = 0.45 (petroleum ether / EtOAc, 7:3)
m.p. = 49-50°C
^{1}H NMR (400 MHz, $CDCl_3$) δ 8.00-7.95 (2H, m, H3), 7.74-7.68 (1H, m, H5), 7.63-7.57 (2H, m, H4), 4.47 (2H, s, H1).
^{13}C NMR (101 MHz, $CDCl_3$) δ 136.1, 134.7, 129.5, 129.1, 16.9.
HRMS (ESI^+) Found $[M+Na]^+$ = 304.9104; $C_7H_7O_2{}^{127}I^{23}Na^{32}S$ requires 304.9104.
Spectroscopic data in agreement with that reported previously.[13]

2-Iodo-3-phenylpropanal, 7o

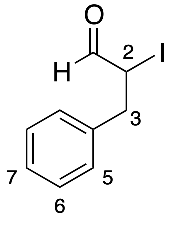

To a solution of L-proline (254 mg, 2.20 mmol, 20 mol %) and *N*-iodosuccinimide (1.60 g, 14.2 mmol, 1.3 equiv.) in dichloromethane (22 mL) was added hydrocinnamaldehyde (1.45 mL, 11.0 mmol, 1.0 equiv.). After stirring the solution for 1 h at room temperature, the reaction mixture was filtered through a short pad of silica (CH_2Cl_2 eluent, 150 mL) and then concentrated *in vacuo* to afforde **7o** (1.94 g, 7.46 mmol, 68%) as a brown oil.

^{1}H NMR (400 MHz, $CDCl_3$) δ_H 9.25 (1H, d, J = 2.6 Hz, ArH), 7.33-7.19 (3H, m, ArH), 7.19-7.11 (2H, m, H5), 4.66 (1H, td, J = 7.5, 2.6 Hz, H2), 3.45 (1H, dd, J = 14.6, 7.5 Hz, H3), 3.16 (1H, dd, J = 14.6, 7.5 Hz, H3).
^{13}C NMR (101 MHz, $CDCl_3$) δ_C 191.1, 138.2, 129.1, 128.9, 127.4, 38.7, 36.0.
Spectroscopic data in agreement with that reported previously.[9]

Methyl (*R*)-2-((tert-butoxycarbonyl)amino)-3-iodopropanoate, 5p

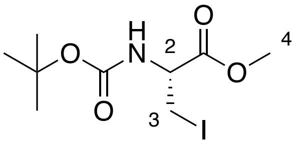

According to the procedure described by Koseki et al.[6] To a solution of triphenylphosphine (1.97 g, 7.50 mmol, 1.5 equiv.) and imidazole (511 mg, 7.5 mmol, 1.5 equiv.) in CH_2Cl_2 (5 mL) at 0 °C was added iodine (1.90 g, 7.50 mmol, 1.5 equiv.) portionwise. The resulting mixture was stirred for 10 min at room temperature and then cooled to 0 °C. Boc-Ser-OMe (1.10 g, 5.00 mmol, 1.0 equiv.) in CH_2Cl_2 (5 mL) was added dropwise, and the resulting mixture was stirred at 0 °C for 2 h, and then concentrated. Purification by column chromatography (SiO_2, petroleum ether / Et_2O, 9:1) afforded **5p** (1.35 g, 4.10 mmol, 81%) as a white solid.

R_f = 0.10 (petroleum ether / Et_2O, 9:1)
m.p. = 41-42°C
^{1}H NMR (400 MHz, $CDCl_3$) δ 5.36 (1H, d, J = 7.7 Hz, NH), 4.51 (1H, dt, J = 7.7, 3.9 Hz, H2), 3.78 (3H, s, H4), 3.63-3.48 (2H, m, H3), 1.44 (9H, s, *t*-Bu).
^{13}C NMR (101 MHz, $CDCl_3$) δ 170.2, 154.9, 80.6, 53.8, 53.1, 28.4, 8.0.
HRMS (CI^+) Found $[M+H]^+$ = 330.0201; $C_9H_{17}INO_4$ requires 330.0197.
IR (film) ν_{max}/cm^{-1} 3370, 2977, 1748, 1712, 1498, 1366, 1345, 1210, 1159.
$[\alpha]_D^{25}$ – 3.7 (c = 3.0, MeOH).

Methyl (*R*)-2-((tert-butoxycarbonyl)amino)-3-iodopropanoate, 5p′

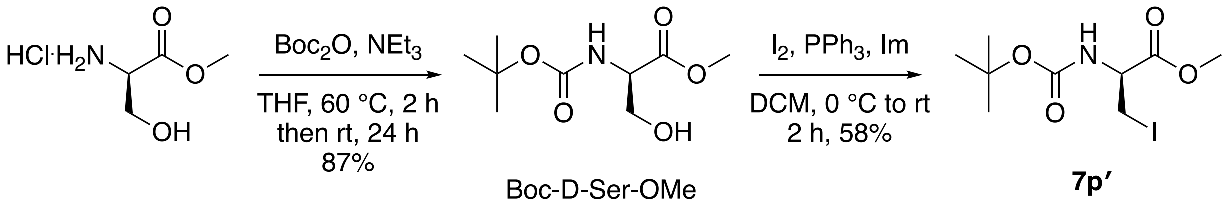

Adapted from the procedure described by Danner et al. [14] To a solution of HCl·H-D-Ser-OMe (1.10 g, 1.0 equiv., 7.1mmol) in THF (25 mL) was added triethylamine (2.1 mL, 2.1 equiv., 15 mmol) and the resulting mixture was cooled to 0 °C. Di-*tert*-butyl-di-carbonate (1.6 mL, 0.99 equiv., 7.0 mmol) was added dropwise at 0 °C and the reaction was stirred at 60 °C for 2 h and at rt for 24 h. The reaction mixture was concentrated and diluted with Et_2O (20 mL) and water (20 mL). The phases were separated and the aqueous was extracted with Et_2O (2 × 20 mL). The combined organics were washed with HCl (aq., 1 M, 20 mL), $NaHCO_3$ (aq., sat., 20 mL) and brine (20 mL), dried ($MgSO_4$) and concentrated *in vacuo*. The residue was purified by column chromatography (pentane / ethyl acetate, 4:6) to afford Boc-D-Ser-OMe (1.36 g, 6.20 mmol, 87%) as a colourless oil.

To a solution of Boc-D-Ser-OMe (1.25 g, 1.0 equiv., 5.70 mmol), triphenylphosphine (2.24 g, 1.5 equiv., 8.55 mmol) and imidazole (582 mg, 1.5 equiv., 8.55 mmol) in CH_2Cl_2 (20 mL) at 0 °C was added iodine (2.17 g, 1.5 equiv., 8.55 mmol) in three portions. The resulting mixture was stirred at 0 °C for 15 min and rt for 2 h. Water (20 mL) was added, the phases were separated and the aqueous was extracted with CH_2Cl_2 (2 × 20 mL). The combined organics were washed with $Na_2S_2O_3$ (aq., 10 %, 20 mL) and brine (20 mL), dried ($MgSO_4$) and concentrated *in vacuo*. Purification by column chromatography (pentane / Et_2O, 9:1 to 8:2) and recrystallisation from pentane at −20 °C afforded **7p′** (1.10 g, 3.34 mmol, 58%) as white fluffy crystals.

Data for Boc-D-Ser-OMe:

^{1}H NMR (400 MHz, $CDCl_3$) δ 5.55 (1H, d, *J* = 8.0 Hz, NH), 4.36 (1H, dt, *J* = 8.4, 3.9 Hz, H2), 4.00 – 3.89 (1H, m, H3), 3.89 – 3.81 (1H, m, H3), 3.75 (3Hs, H4), 2.89 (1H, br t, *J* = 5.9 Hz, OH), 1.43 (9H, s, *t*-Bu).

^{13}C NMR (101 MHz, $CDCl_3$) δ 171.4, 155.8, 80.3, 63.4, 55.7, 52.6, 28.3.

MS (ESI+) 242.0 [M+Na$^+$]

$[\boldsymbol{a}]_{\boldsymbol{D}}^{\mathbf{25}}$ 15.7 ° (c = 3.0, MeOH)

Spectroscopic data in agreement with that reported previously. [15]

Data for **7p′**:

R$_f$ = 0.10 (petroleum ether / Et_2O, 9:1)
m.p. = 48-49 °C
^{1}H NMR (400 MHz, $CDCl_3$) δ 5.35 (1H, d, *J* = 6.1 Hz, NH), 4.51 (1H, dt, *J* = 7.9, 4.0 Hz, H2), 3.79 (3H, d, *J* = 1.0 Hz, H4), 3.62 – 3.50 (2H, m, H3), 1.45 (9H, s, *t*-Bu).
^{13}C NMR (101 MHz, $CDCl_3$) δ 170.2, 155.0, 80.6, 53.8, 53.1, 28.4, 8.0.
HRMS (ESI^+) Found $[M+Na]^+$ = 352.0014; $C_9H_{16}O_4N^{127}I^{23}Na$ requires 352.0016
IR (film) ν_{max}/cm^{-1} 3377, 2977, 2915, 2878, 1748, 1714, 1500, 1346, 1366, 1162.
$[\alpha]_D^{25}$ 3.8 ° (c = 3.0, MeOH)

1-(iodomethyl)-4-nitrobenzene, 7r

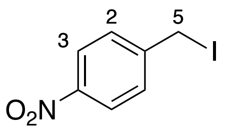

4-Nitrobenzyl bromide (664 mg, 3.07 mmol, 1.0 equiv.) and sodium iodide (691 mg, 4.61 mmol, 1.5 equiv.) in acetone (5 mL) were submitted to **General Procedure 2** (12 h). Recrystallisation from CH_2Cl_2 / pentane afforded **7r** (350 mg, 1.33 mmol, 43%) as pale yellow crystals.

R$_f$ = 0.22 (petroleum ether / Et_2O, 9:1)
m.p. = 121-122 °C
^{1}H NMR (400 MHz, $CDCl_3$) δ 8.16 (2H, d, *J* = 8.7 Hz, H3), 7.52 (2H, d, *J* = 8.8 Hz, H2), 4.48 (2H, s, H5).
^{13}C NMR (101 MHz, $CDCl_3$) δ 147.4, 146.9, 129.7, 124.2, 2.2.
HRMS (CI^+) Found $[M+NH_4]^+$ = 280.9787; $C_7H_{10}IN_2O_2$ requires 280.9781.
Spectroscopic data in agreement with that reported previously.[16]

1-(iodomethyl)-4-(trifluoromethyl)benzene, 7s

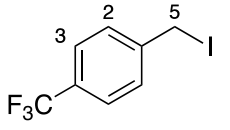

4-(Trifluoromethyl)benzyl bromide (1.20 g, 5.00 mmol, 1.0 equiv.) and sodium iodide (1.12 g, 7.50 mmol, 1.5 equiv.) in acetone (10 mL) were submitted to **General Procedure 2** (12 h).

Purification by column chromatography (SiO_2, pentane) afforded **7s** as a white solid (1.37 g, 4.79 mmol, 95%).

R$_f$ = 0.42 (pentane)
m.p. = 38-39 °C
^{1}H NMR (400 MHz, $CDCl_3$) δ 7.56 (2H, d, *J* = 8.2 Hz, H3), 7.48 (2H, d, *J* = 8.2 Hz, H2), 4.46 (2H, s, H5).
^{13}C NMR (101 MHz, $CDCl_3$) δ 143.3 (q, *J* = 1.6 Hz), 129.9 (q, *J* = 32.6 Hz), 129.0, 125.8 (q, *J* = 3.8 Hz), 123.9 (q, *J* = 272.1 Hz), 3.2.
Spectroscopic data in agreement with that reported previously.[17]

1-(iodomethyl)-3,5-bis(trifluoromethyl)benzene, 7t

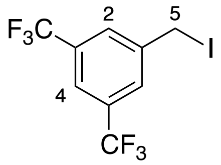

1-(bromomethyl)-3,5-bis(trifluoromethyl)benzene (916 μL, 5.00 mmol, 1.0 equiv.) and sodium iodide (1.12 g, 7.50 mmol, 1.5 equiv.) in acetone (5 mL) were submitted to **General Procedure 2** (12 h) to afford **7t** (1.68 g, 4.75 mmol, 94%) as a yellowish solid.

R$_f$ = 0.32 (pentane)
m.p. = 27-28 °C
^{1}H NMR (400 MHz, $CDCl_3$) δ 7.81 (2H, s, H2), 7.76 (1H, s, H4), 4.49 (2H, s, H5).
^{13}C NMR (101 MHz, $CDCl_3$) δ 142.1, 132.4 (q, *J* = 33.5 Hz), 128.9 (q, *J* = 3.1 Hz), 123.1 (q, *J* = 273 Hz), 121.9 (m), 1.4.
^{19}F NMR (377 MHz, $CDCl_3$) δ −63.03.
HRMS (EI^+) Found $[M–I]^+$=227.0294; $C_9H_5F_6$ requires 227.0290.
IR (film) ν_{max}/cm^{-1} 1375, 1275, 1183, 1156, 1117, 924, 897, 855, 730, 702, 682, 648.

2-(Iodomethyl)benzonitrile, 7u

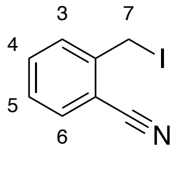

2-(Bromomethyl)benzonitrile (273 mg, 1.39 mmol, 1.0 equiv.) and sodium iodide (313 mg, 2.09 mmol, 1.5 equiv.) in acetone (5 mL) were submitted to **General Procedure 2** (12 h). Purification by column chromatography (SiO_2, petroleum ether / Et_2O, 1:0→1:1) afforded **7u** (287 mg, 1.18 mmol, 84%) as a white solid.

R$_f$ = 0.37 (petroleum ether / Et_2O, 9:1)
m.p. = 71-72 °C (lit. 76.5-78.5 °C)[18]
^{1}H NMR (400 MHz, $CDCl_3$) δ 7.62 (1H, d, *J* = 7.6 Hz, H6), 7.57–7.50 (2H, m, H4, H5), 7.36 (1H, ddd, *J* = 7.7, 6.3, 2.5 Hz, H3). 4.60 (2H, s, H7).
^{13}C NMR (101 MHz, $CDCl_3$) δ 143.0, 133.4, 133.4, 130.1, 128.5, 117.0, 111.9, 0.4.
HRMS (CI$^+$) Found $[M+NH_4]^+$ = 260.9890; $C_8H_{10}IN_2$ requires 280.9883.
IR (film) ν_{max}/cm^{-1} 2981, 2225, 1485, 1450, 1432, 1220, 1156, 1072, 962, 771, 743.

(Iodomethyl)cyclopropane, 7v

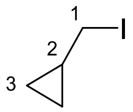

(Bromomethyl)cyclopropane (1.00 g, 7.41 mmol, 1.0 equiv), sodium iodide (6.00 g, 40.0 mmol, 5.4 equiv.) in acetone (10 mL) were submitted to **General Procedure 2** (17 h) to afford **5v** (629 mg, 3.46 mmol, 47%) as a colourless oil.

R$_f$ = 0.66 (pentane)
^{1}H NMR (400 MHz, $CDCl_3$) δ_H 3.13 (2H, d, *J* = 7.7 Hz, H1), 1.38-1.23 (1H, m, H2), 0.87-0.77 (2H, m, H3), 0.35-0.26 (2H, m, H3).
^{13}C NMR (101 MHz, $CDCl_3$) δ_C 16.1, 14.2, 11.1.
HRMS (CI$^+$) Found $[M+H]^+$ = 182.9666; C_4H_8I requires 182.9665.
IR (film) ν_{max}/cm^{-1} 3002, 1427, 1174, 1017, 825.
Spectroscopic data in agreement with that reported previously. [19]

Dimethyl bromomalonate, 8c

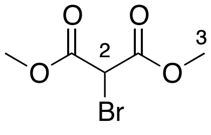

According to the procedure described by Wolfe et al.[20] To a solution of dimethyl malonate (3.40 mL, 30.0 mmol, 1.0 equiv.) in $CHCl_3$ (50 mL) was added *N*-bromosuccinimide (5.90 g,

33.0 mmol, 1.1 equiv.) and *p*-toluenesulfonic acid monohydrate (1.10 g, 6.00 mmol, 0.2 equiv.), and the resulting mixture was stirred for 2 h at 70 °C. The reaction was then cooled to room temperature, water was added, and the phases were separated. The aqueous phase was extracted with CH_2Cl_2 (2 × 50 mL), and the combined organic phases were washed with $Na_2S_2O_3$ (10% aq., 50 mL) and brine, dried ($MgSO_4$) and concentrated *in vacuo*. Purification by column chromatography (SiO_2, petroleum ether / Et_2O, 9:1) afforded **8c** (2.00 g, 9.48 mmol, 31%) as a colourless oil.

R_f = 0.15 (petroleum ether / Et_2O, 9:1)
^{1}H NMR (400 MHz, $CDCl_3$) δ 4.85 (1H, s, H2), 3.82 (6H, s, OMe).
^{13}C NMR (101 MHz, $CDCl_3$) δ 165.1, 54.1, 41.7.
IR (film) ν_{max}/cm^{-1} 2959, 1738, 1436, 1289, 1248, 1213, 1017, 915, 893, 787, 721, 652.
HRMS (ESI$^+$) Found $[M+H]^+$ = 210.9604; $C_5H_8O_4{}^{79}Br$ requires 210.9601.
Spectroscopic data in agreement with that reported previously.[20]

Diethyl 2-bromo-2-methylmalonate, 8d

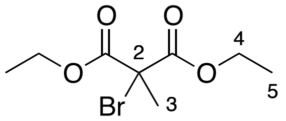

According to the procedure described by Curran et al.[21] To a solution of diethyl methylmalonate (852 μL, 5.00 mmol, 1.0 equiv.) in CCl_4 (10 mL) was added N-bromosuccinimide (1.33 g, 7.50 mmol, 1.5 equiv.). The mixture was refluxed for 12 h, then cooled to 0 °C and filtered. The filtrate was concentrated. Distillation of the residue (2.0 mbar, 115 °C) afforded **8d** as a colourless oil (846 mg, 3.34 mmol, 66%).

R_f = 0.36 (petroleum ether / Et_2O, 9:1)
^{1}H NMR (400 MHz, $CDCl_3$) δ 4.27 (4H, q, *J* = 7.1 Hz, H4), 2.07 (3H, s, H3), 1.29 (6H, t, *J* = 7.1 Hz, H5).
^{13}C NMR (101 MHz, $CDCl_3$) δ 167.6, 63.2, 56.8, 26.9, 14.0.
HRMS (CI$^+$) Found $[M+H]^+$ = 253.0066; $C_8H_{14}{}^{79}BrO_4$ requires 253.0070.
IR (film) ν_{max}/cm^{-1} 2986, 1741, 1446, 1378, 1300, 1259, 1223, 1115, 1069, 1017, 859, 646.

2-Bromo-1,3-diphenylpropane-1,3-dione, 8e

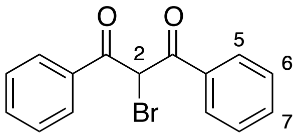

According to the procedure described by Izumisawa et al.[22] To a solution of 1,3-diphenylpropane-1,3-dione (2.24 g, 10.0 mmol, 1.0 equiv.) in CH_2Cl_2 (50 mL) was added *N*-bromosuccinimide (1.96 g, 11.0 mmol, 1.1 equiv.) and *p*-toluenesulfonic acid monohydrate (380 mg, 2.00 mmol, 0.2 equiv.) and the resulting mixture was stirred for 15 min at rt. Water was added and the phases were separated. The aqueous phase was extracted twice with CH_2Cl_2 and the combined organics were washed with $Na_2S_2O_3$ (10% *aq.*) and brine, dried ($MgSO_4$) and concentrated. Recrystallisation from hot cyclohexane afforded **8e** (2.02 g, 6.66 mmol, 74%) as white crystals.

m.p. = 86-87 °C.

R$_f$ = 0.37 (petroleum ether / EtOAc, 7:3)

^{1}H NMR (400 MHz, $CDCl_3$) δ 8.04-7.94 (4H, m, H5), 7.65-7.55 (2H, m, H7), 7.54-7.42 (4H, m, H6), 6.57 (1H, s, H2).

^{13}C NMR (101 MHz, $CDCl_3$) δ 189.1, 134.4, 133.9, 129.4, 129.1, 52.8.

HRMS (ESI$^+$) Found $[M+H]^+$ = 303.0017; $C_{15}H_{12}{}^{79}BrO_2$ requires 303.0015.

Spectroscopic data in agreement with that reported previously.[23]

2-Bromo-1-phenyl-2-tosylethan-1-one, 8f

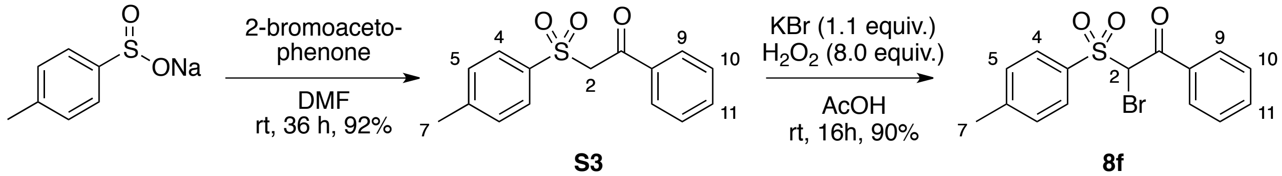

According to the procedure described by Suryakiran et al.[24] To a suspension of sodium toluenesulfinate (1.00 g, 5.60 mmol, 1.0 equiv.) in DMF (10 mL) was added 2-bromoacetophenone (1.10 g, 5.60 mmol, 1.0 equiv.). The mixture was stirred at room temperature for 36 h. Water (150 mL) was added and the aqueous layer was extracted three times with CH_2Cl_2. The combined organic phases were washed with water and brine, dried ($MgSO_4$) and concentrated afforded **S3** (1.42 g, 5.18 mmol, 92%) as a white powder that required no further purification.

According to the procedure described by Suryakiran et al.[25] To a suspension of **S3** (400 mg, 1.46 mmol, 1.0 equiv.) and potassium bromide (191 mg, 1.60 mmol, 1.1 equiv.) in AcOH (1.5 mL) and water (0.5 mL) was added hydrogen peroxide (710 µL, 47 wt% solution in water, 11.6 mmol, 8.0 equiv.) and the resulting mixture was stirred at room temperature for 18 h. Water (20 mL) was added and the aqueous layer was extracted three times with EtOAc. The combined organic phases were washed with water and brine, dried ($MgSO_4$) and concentrated. Purification by column chromatography (SiO_2, petroleum ether / EtOAc, 8:2) afforded **8f** (464 mg, 1.31 mmol, 90%) as a white powder.

Data for **S3:**

m.p. = 103-104°C

R$_f$ = 0.23 (Petroleum ether / EtOAc, 8:2)

^{1}H NMR (400 MHz, $CDCl_3$) δ 7.96-7.90 (2H, m, H9), 7.76 (2H, d, *J* = 8.4 Hz, H4), 7.64-7.58 (1H, m, H11), 7.50-7.43 (2H, m, H10), 7.32 (2H, d, *J* = 8.1 Hz, H5), 4.72 (2H, s, H2), 2.43 (3H, s, H7).

^{13}C NMR (101 MHz, $CDCl_3$) δ 188.2, 145.4, 135.8, 134.4, 129.9, 129.4, 128.9, 128.7, 77.2, 63.6, 21.8.

MS (ESI+): 297.0 $[M + Na]^+$, 571.1 $[2M + Na]^+$.

Spectroscopic data in agreement with that reported previously.[26]

Data for **8f**:

m.p. = 154-155 °C.

R$_f$ = 0.22 (petroleum ether / Et_2O, 8:2), [UV, vanillin]

^{1}H NMR (400 MHz, $CDCl_3$) δ 8.02-7.97 (2H, m, H9), 7.87-7.82 (2H, m, H4), 7.68-7.62 (1H, m, H11), 7.54-7.48 (2H, m, H10), 7.40-7.35 (2H, m, H5), 6.23 (1H, s, H2), 2.47 (3H, s, H7).

^{13}C NMR (101 MHz, $CDCl_3$) δ 186.8, 146.6, 134.9, 134.4, 131.8, 131.1, 129.7, 129.6, 129.1, 60.2, 22.0.

HRMS (ESI+) Found $[M+Na]^+$ = 374.9657 ; $C_{15}H_{13}O_3{}^{79}BrNaS$ requires 374.9661.

IR (film) ν_{max}/cm^{-1} 3067, 2974, 1688, 1595, 1449, 1329, 1269, 1153, 1084, 974, 818, 748, 685, 556.

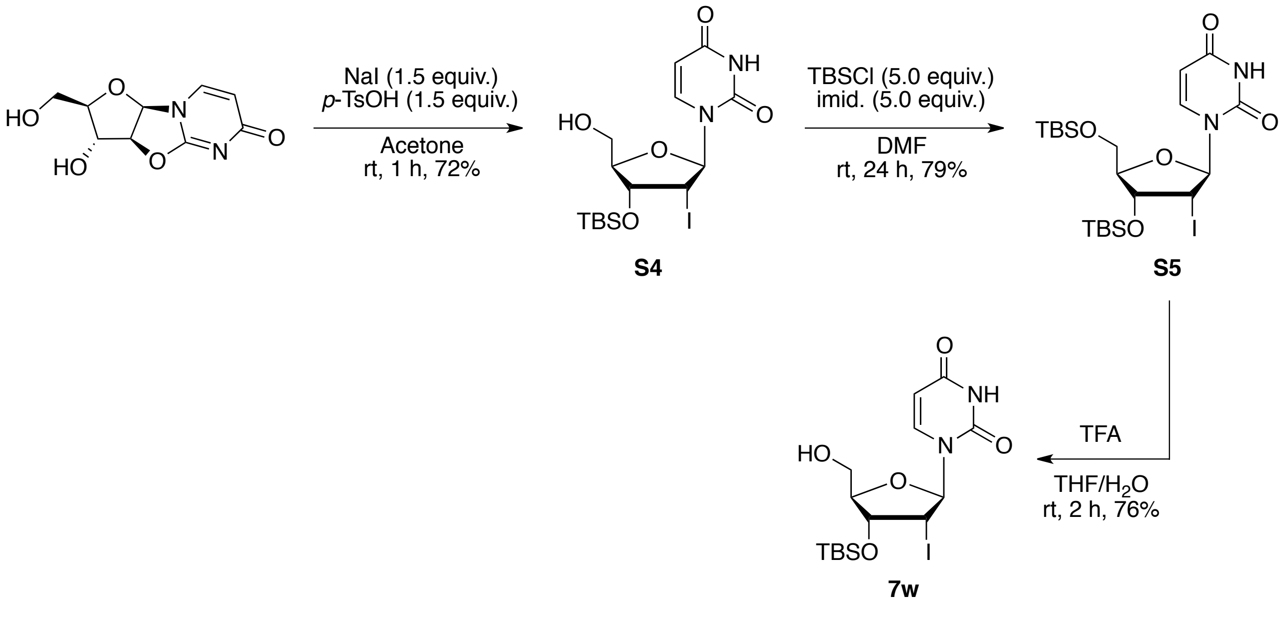

Scheme S1: Synthesis of **7w**

2′-iodo-2′-deoxyuridine, S4

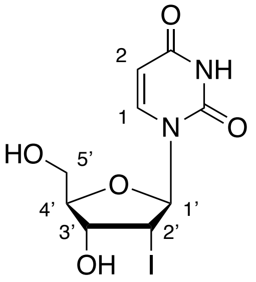

According to the procedure described by Haugland et al.[27] To a solution of O2,2'-cyclouridine (1.13 g, 5.00 mmol, 1.0 equiv.) in acetone (50 mL) was added sodium iodide (1.12 g, 7.50 mmol, 1.5 equiv.) and para-toluenesulfonic acid (1.43 g, 7.50 mmol, 1.5 equiv.). The resulting mixture was stirred at 50 °C for 5h, then cooled to room temperature and filtered. The solid was washed with acetone, and the filtrate was concentrated. The residue was diluted with acetone (7 mL) and washed with $Na_2S_2O_3$ (aq. sat., 7 mL). The aqueous phase was extracted with acetone (2 × 5 mL) and the combined organic phases were concentrated. Purification by column chromatography (SiO_2, CH_2Cl_2 / MeOH, 95:5→80:20) afforded **S4** (1.28 g, 3.61 mmol, 72%) as an off-white foam.

^{1}H NMR (400 MHz, MeOD) δ 7.97 (1H, d, *J* = 8.1 Hz, H1), 6.31 (1H, d, *J* = 7.4 Hz, H1'), 5.70 (1H, d, *J* = 8.1 Hz, H2), 4.49 (1H, dd, *J* = 7.4, 5.1 Hz, H2'), 4.08 (1H, q, *J* = 3.0 Hz, H4'), 3.92 (1H, dd, *J* = 5.1, 3.2 Hz, H3'), 3.79 (1H, dd, *J* = 12.2, 3.0 Hz, H5'), 3.72 (1H, dd, *J* = 12.2, 2.9 Hz, H5').

^{13}C NMR (101 MHz, MeOD) δ 165.9, 152.3, 141.8, 103.2, 91.9, 87.2, 72.2, 62.3, 32.4.

HRMS (ESI$^+$) Found $[M+Na]^+$ = 376.9614; $C_9H_{11}O_5N_2{}^{127}I^{23}Na$ requires 376.9505.

Spectroscopic data in accordance to that reported previously.[27]

3′,5′-di(*O*-(*tert*-butyldimethylsilyl))-2′-iodo-2′-deoxyuridine, S5

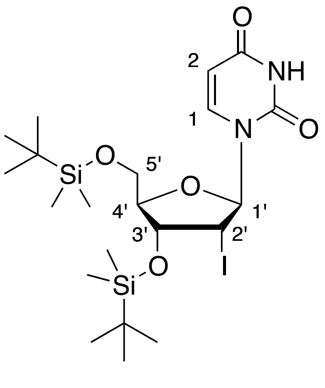

Adapted from the procedure described by Seamon et al.[28] To a solution of **S4** (1.10 g, 3.11 mmol, 1.0 equiv.) in DMF (15 mL) was added *tert*-butylchlorodimethylsilane (2.34 g, 15.6

mmol, 5.0 equiv.) and imidazole (1.06 g, 15.6 mmol, 5.0 equiv.), and the resulting mixture was stirred at room temperature for 24 h. Water (200 mL) was added and the aqueous phase was extracted with Et_2O (3 x 50 mL). The combined organics were washed LiCl (10% aq., 2 x 50 mL) and brine (50 mL), dried ($MgSO_4$) and concentrated. Purification by column chromatography (SiO_2, petroleum ether / Et_2O, 1:0→0:1) afforded **S5** (1.43 g, 2.45 mmol, 79%) as a white foam.

R_f = 0.23 (petroleum ether / Et_2O, 1:1)

^{1}H NMR (400 MHz, $CDCl_3$) δ 8.96 (1H, s, NH), 7.86 (1H, d, *J* = 8.2 Hz, H1), 6.38 (1H, d, *J* = 5.6 Hz, H1'), 5.71 (1H, dd, *J* = 8.2, 2.2 Hz, H2), 4.28 (1H, t, J = 5.6 Hz, H2'), 4.11 (1H, dt, *J* = 4.0, 2.0 Hz, H4'), 3.95 (1H, dd, *J* = 11.7, 2.4 Hz, H5'), 3.82 (app t, *J* = 4.5 Hz, H3'), 3.75 (1H, dd, *J* = 11.7, 1.8 Hz, H5'), 0.93 (9H, s, Si*t*-Bu), 0.92 (9H, s, Si*t*-Bu), 0.17 (3H, s, SiMe), 0.12 (3H, s, SiMe), 0.11 (3H, s, SiMe), 0.10 (3H, s, SiMe).

^{13}C NMR (101 MHz, $CDCl_3$) δ 163.1, 150.3, 139.5, 102.8, 91.2, 86.0, 71.3, 62.1, 32.4, 26.0, 25.9, 18.5, 18.3, -4.2, -4.4, -5.4, -5.4.

HRMS (CI^+) Found $[M+Na]^+$ = 605.1327; $C_{21}H_{39}O_5N_2{}^{127}I^{23}NSi_2$ requires 605.1334.

IR (film) ν_{max}/cm^{-1} 2955, 2929, 2858, 1698, 1625, 1461, 1254, 1112, 835, 779.

3′-*O*-(*tert*-butyldimethylsilyl)-2′-iodo-2′-deoxyuridine, 7w

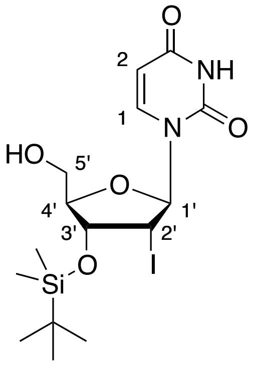

Adapted form the procedure described by Sun et al.[29] To a solution of **S5** (1.43 g, 2.45 mmol, 1.0 equiv.) in THF (20 mL) was added a solution of trifluoroacetic acid (5 mL) in water (5 mL) at 0 °C. The resulting mixture was allowed to stir at room temperature for 2 h, and then quenched cautiously with solid $NaHCO_3$. Water (50 mL) was added, and the aqueous phase was extracted with Et_2O (3 x 25 mL). The combined organic phases were washed with $NaHCO_3$ (aq. sat., 25 mL) and brine, dried ($MgSO_4$) and concentrated. Purification by column chromatography (SiO_2, CH_2Cl_2 / EtOAc, 1:0→0:1) afforded **7w** (825 mg, 1.76 mmol, 71%) as a white solid.

R_f = 0.27 (CH_2Cl_2 / EtOAc, 1:1)

m.p. = 190 °C (decomp.)

^{1}H NMR (400 MHz, MeOD) δ 7.99 (1H, d, *J* = 8.1 Hz, H1), 6.35 (1H, d, *J* = 7.4 Hz, H1'), 5.75 (1H, d, *J* = 8.1 Hz, H2), 4.58 (1H, dd, *J* = 7.4, 4.5 Hz, H2'), 4.13-4.08 (2H, m, H3', H4'), 3.82 (1H, dd, *J* = 12.2, 3.1 Hz, H5'), 3.74 (1H, dd, *J* = 12.2, 2.7 Hz, H5'), 0.99 (9H, s, Si*t*-Bu), 0.24 (3H, s, SiMe), 0.19 (3H, s, SiMe).

^{13}C NMR (101 MHz, MeOD) δ 165.9, 152.4, 141.7, 103.3, 92.1, 87.9, 73.7, 62.0, 31.7, 26.4, 19.0,

-4.3, -4.4.

HRMS (ESI$^+$) Found $[M+H]^+$ = 469.0649; $C_{15}H_{26}O_5N_2{}^{127}I^{28}Si$ requires 469.0650.

IR (film) ν_{max}/cm^{-1} 3405, 3222, 3062, 2955, 2923, 2856, 1690, 1676, 1462, 1169, 1128, 1102, 1025.

$[\alpha]_D^{25}$ 27.9 (c = 1.0, MeOH)

2-iodoethyl 4-methylbenzenesulfonate, 7y

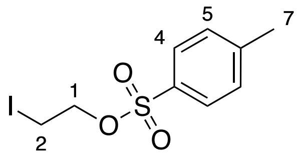

Adapted from the procedure described by Wegert et al.[30] To a round-bottomed flask charged with pyridine (2 mL) at 0 °C was added *p*-toluenesulfonyl chloride (1.91 g, 10.0 mmol, 1.0 equiv.). The resulting mixture was stirred at 0 °C for 5 min, then 2-iodoethanol (780 μL, 10.0 mmol, 1.0 equiv.) was added dropwise. The solution was allowed to warm to rt, and stirred for 2 h, then cooled to 0 °C. HCl (aq., 5 M, 10 mL) was added, and the aqueous phase was extracted with Et_2O (3 × 10 mL). The combined organic phases were washed with $Na_2S_2O_3$ (aq., 10 wt%, 10 mL) and brine (10 mL), dried (Na_2SO_4) and concentrated. Purification by column chromatography (SiO_2, petroleum ether / Et_2O, 1:0→0:1) afforded **7y** (1.28 g, 3.92 mmol, 39%) as a yellow oil.

R_f = 0.18 (petroleum / Et_2O, 9:1)

^{1}H NMR (400 MHz, $CDCl_3$) δ 7.80 (2H, d, *J* = 7.3 Hz, H4), 7.36 (2H, d, *J* = 8.0 Hz, H5), 4.23 (2H, t, *J* = 7.4 Hz, H1), 3.26 (2H, t, *J* = 7.3 Hz, H2), 2.46 (3H, s, H7).

^{13}C NMR (101 MHz, $CDCl_3$) δ 145.3, 132.8, 130.1, 128.1, 69.7, 21.8, -1.3.

HRMS (ESI$^+$) Found $[M+Na]^+$ = 348.9367; $C_9H_{11}O_3{}^{127}I^{23}Na^{32}S$ requires 348.9366.

IR (film) $ν_{max}/cm^{-1}$ 1597, 1358, 1189, 1173, 1096, 939, 862, 814, 741, 662.

Boc-Asp(O*t*-Bu)-Ser-OMe, 15

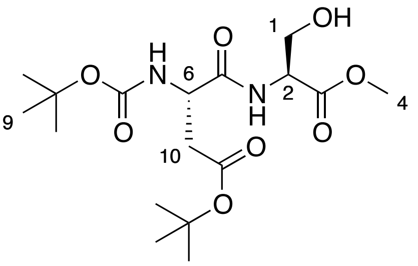

Adapted from the procedure described by Pu et al.[31] To a suspension of HCl.H-Ser-OMe (422 mg, 4.00 mmol, 1.0 equiv.) in EtOH (10 mL) was added *N*-methylmorpholine (1.32 mL, 12.0 mmol, 3.0 equiv.), a solution of Boc-Asp(O*t*-Bu)-OH (1.19 g, 4.12 mmol, 1.03 equiv.), and HOBt (82 mg, 0.60 mmol, 30 mol %) in EtOH (10 mL). The resulting mixture was cooled to 0 °C and stirred at this temperature for 15 min. EDCI (920 mg, 4.80 mmol, 1.2 equiv.) was added in three portions at 0 °C, and the resulting mixture warmed to room temperature and stirred for 12 h. Water (40 mL) was added, and the aqueous phase was extracted with EtOAc (3 x 25 mL). The combined organic phases were washed with pH 2 buffer solution (2 × 25 mL), NaHCO3 (aq., sat., 25 mL) and brine (25 mL), dried ($MgSO_4$) and concentrated. Purification by column chromatography (SiO_2, petroleum ether / EtOAc, 1:1→7:3) afforded **15** (1.53 g, 3.92 mmol, 97%) as a white foam.

$\mathbf{R_f}$ = 0.17 (petroleum ether/EtOAc, 1:1)

^{1}H NMR (400 MHz, $CDCl_3$) δ 7.34 (1H, d, *J* = 7.4 Hz, NH), 5.68 (1H, d, *J* = 8.6 Hz, NH), 4.57 (1H, dt, *J* = 7.2, 3.4 Hz, H2), 4.46 (1H, dt, *J* = 9.7, 5.6 Hz, H6), 4.00-3.82 (2H, m, H1), 3.74 (3H, s, H4), 3.23 (1H, t, *J* = 6.5 Hz, OH), 2.89 (1H, dd, *J* = 17.0, 5.4 Hz, H10), 2.63 (1H, dd, *J* = 17.0, 5.4 Hz, H10), 1.42 (9H, s, *t*-Bu), 1.40 (9H, s, *t*-Bu).

^{13}C NMR (101 MHz, $CDCl_3$) δ 171.4, 171.3, 170.7, 155.6, 82.1, 80.7, 62.4, 55.1, 52.8, 51.0, 37.8, 28.3, 28.1.

HRMS (ESI^+) Found $[M+Na]^+$ = 413.1899; $C_{17}H_{30}O_8N_2{}^{23}Na$ requires 413.1894.

IR (film) $ν_{max}/cm^{-1}$ 3339, 2978, 2935, 1726, 1673, 1523, 1368, 1293, 1249, 1230, 1161.

$[\boldsymbol{a}]_{\boldsymbol{D}}^{\mathbf{25}}$ = 35.1 (c 1.1, $CHCl_3$)

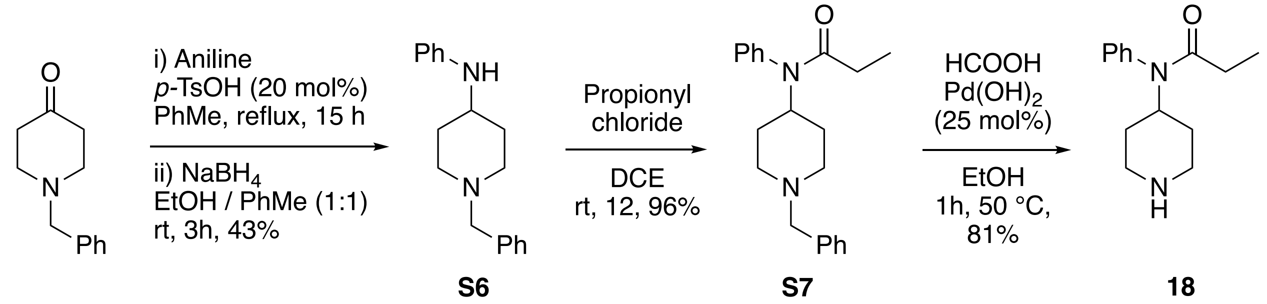

Scheme S2: Synthesis of **18**

1-benzyl-*N*-phenylpiperidin-4-amine, S6

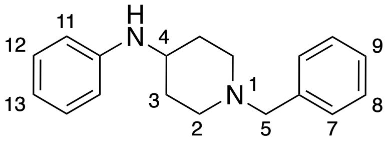

According to the procedure described by Brine et al.[32] To a solution of *N*-benzyl-4-piperidone (1.85 mL, 10.0 mmol, 1.0 equiv.) in toluene (50 mL) was added *p*-toluenesulfonic acid monohydrate (38 mg, 0.20 mmol, 0.2 equiv.) and aniline (1.00 mL, 11.0 mmol, 1.1 equiv.), and the mixture was stirred under reflux for 15 h using a Dean-Stark apparatus. The mixture was cooled to room temperature and EtOH (50 mL) was added. Sodium borohydride (380 mg, 10.0 mmol, 1.0 equiv.) was added portionwise and the resulting mixture was stirred at room temperature for 3 h. Water (20 mL) was added dropwise and the mixture was stirred for 4 h at rt, then it was acidified to pH 3 with HCl (10%, aq.) and washed with toluene (3 × 25 mL). The aqueous phase was basified with NaOH (50%, aq.), and extracted with CH_2Cl_2 (3 × 50 mL). The combined organics were dried ($MgSO_4$) and concentrated. Recrystallization from Et_2O with petroleum ether afforded **S6** (1.15 g, 4.31 mmol, 43%) as yellow crystals.

$\mathbf{R_f}$ = 0.25 (petroleum ether / EtOAc, 1:1)

m.p. = 81-82 °C

^{1}H NMR (400 MHz, $CDCl_3$) δ 7.38 (2H, app s, H7 or H8), 7.37 (2H, s app, H7 or H8), 7.34-7.28 (1H, m, H9), 7.25-7.16 (2H, m, H12), 6.73 (1H, tt, *J* = 7.3, 1.1 Hz, H13), 6.67-6.60 (2H, m, H11), 3.58 (2H, s, H5), 3.55 (1H, br s, NH), 3.34 (1H, dq, *J* = 10.7, 5.3 Hz, H4), 2.90 (2H, dt, *J* = 11.9, 4.0 Hz, H2), 2.19 (2H, td, *J* = 11.5, 2.6 Hz, H2), 2.13-2.04 (2H, m, H3), 1.59-1.45 (2H, m, H3).

^{13}C NMR (101 MHz, $CDCl_3$) δ 147.2, 138.5, 129.4, 129.2, 128.3, 127.1, 117.2, 113.3, 63.2, 52.5, 50.0, 32.7.

HRMS (ESI^+) Found $[M+H]^+$ = 267.1854; $C_{18}H_{23}N_2$ requires 267.1856.

Spectroscopic data in agreement with that reported previously.[33]

N-(1-benzylpiperidin-4-yl)-*N*-phenylpropionamide, S7

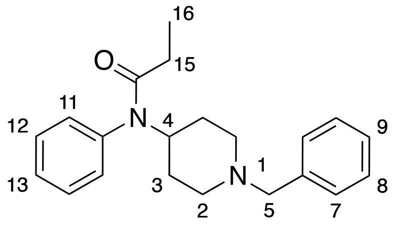

According to the procedure described by Gupta et al.[33] To a solution of **S6** (799 mg, 3.00 mmol, 1.0 equiv.) in 1,2-dichloroethane (5 mL) was added propionyl chloride (786 uL, 9.00 mmol, 3.0 equiv.) dropwise at rt. The resulting mixture was stirred at room temperature for 12 h, and then quenched with NaOH (4%, aq.). The phases were separated and the aqueous phase was extracted with CH_2Cl_2 (2 × 5 mL). The combined organic phases were dried (Na_2SO_4) and concentrated *in vacuo*. Purification by column chromatography (SiO_2, petroleum ether / EtOAc, 1:0→0:1) afforded **S7** (930 mg, 2.89 mmol, 96%) as a yellow oil.

R_f = 0.26 (petroleum ether / EtOAc, 1:1)
^{1}H NMR (400 MHz, $CDCl_3$) δ 7.38-7.25 (3H, m, H12, H13), 7.25-7.08 (5H, m, H7, H8, H9), 7.03-6.94 (2H, m, H11), 4.59 (1H, tt, *J* = 12.2, 3.9 Hz, H4), 3.36 (2H, s, H5), 2.87 (2H, m, H2), 2.03 (2H, td, *J* = 12.0, 2.3 Hz, H2), 1.84 (2H, q, *J* = 7.5 Hz, H15), 1.74-1.58 (2H, m, H3), 1.31 (2H, qd, *J* = 12.3, 3.9 Hz, H3), 0.93 (3H, t, *J* = 7.5 Hz, H16).
^{13}C NMR (101 MHz, CDCl3) δ 173.6, 141.0, 139.1, 138.4, 130.5, 129.4, 129.2, 128.3, 128.3, 127.1, 63.2, 53.2, 52.4, 30.7, 28.6, 9.7.
HRMS (ESI^+) Found $[M+H]^+$ = 323.2110; $C_{21}H_{27}ON_2$ requires 323.2118.
Spectroscopic data in agreement with that reported previously.[33]

N-phenyl-*N*-(piperidin-4-yl)propionamide (Norfentanyl), 18

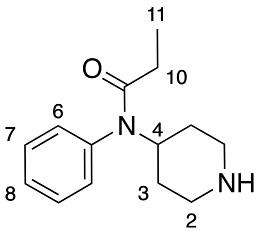

Adapted from the procedure described by Sudo et al.[34] To a solution of **S7** (1.78 g, 5.52 mmol, 1.0 equiv.) in EtOH (20 mL) was added $Pd(OH)_2$ (969 mg, 20 wt% over carbon, 1.38 mmol, 25 mol %) and HCOOH (4.17 mL, 110 mmol, 20 equiv.). The resulting mixture was stirred at 50 °C for 1 h, filtered (celite, washed with EtOH, 3 × 20 mL). The filtrate was concentrated. Purification by column chromatography (SiO_2, CH_2Cl_2 / CH_3OH, 8:2) afforded **18** (1.00 g, 4.30 mmol, 77%) as a beige foam.

$\mathbf{R_f}$ = 0.28 (CH_2Cl_2 / CH_3OH, 8:2)
^{1}H NMR (400 MHz, MeOD) δ 7.54-7.43 (3H, m, H7, H8), 7.24 (2H, d, *J* = 7.3 Hz, H6), 4.77 (1H, tt, *J* = 12.2, 3.9 Hz, H4), 3.44-3.35 (2H, m, H2), 3.15-3.05 (1H, m, H2), 2.06 (2H, d, *J* = 13.7 Hz, H3), 1.96 (2H, q, *J* = 7.5 Hz, H10), 1.58 (2H, qd, *J* = 13.3, 4.1 Hz, H3), 0.97 (3H, t, *J* = 7.5 Hz, H11).
^{13}C NMR (101 MHz, MeOD) δ 176.0, 139.4, 131.4, 130.9, 130.2, 51.5, 44.9, 29.3, 28.6, 9.9.
HRMS (ESI^+) Found $[M+H]^+$ = 233.1649; $C_{14}H_{21}ON_2$ requires 233.1648.

10. Computational details

All calculations were performed using Gaussian 09 software.[35] Structural optimizations and frequency calculations were performed with the (RO)B3LYP,[36] (RO)M06-2x,[37] (RO)ωB97xD[38] functional along with the def2tzvp basis set[39] *in vacuo*. Single point energies have been extracted from the same method / basis set combinations indicated above.

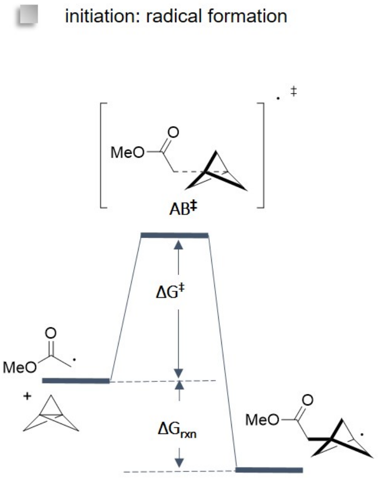

method	ΔG‡	ΔG_{rxn}
ROB3LYP	13.9	7.3
ROM062x	12.5	9.5
ROwb97xd	12.6	16.4

Fig Comp S1. Comparison of computed activation barriers and exergonicities for the initiation step of the radical process depending on the method employed. All numbers in kcal/mol.

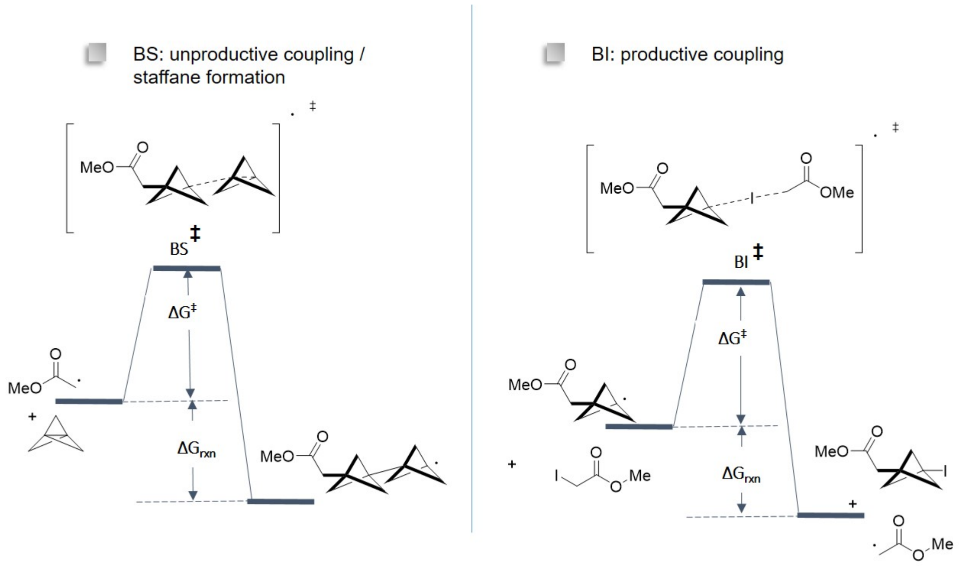

method	ΔG‡ (BS)	ΔG‡ (BI)	ΔΔG‡ (BS - BI)	ΔG_{rxn} (BS)	ΔG_{rxn} (BI)
ROB3LYP	14.2	7.4	6.8	-20.7	-13.8
ROM062x	13.3	7.3	6.1	-21.4	-14.6
ROwb97xd	12.3	9.0	3.3	-29.6	-16.9

Fig Comp S2. Comparison of computed activation barriers and exergonicities for the propagation step (BI) of the radical process and the sideproduct formation (BS) depending on the method employed. All numbers in kcal/mol.

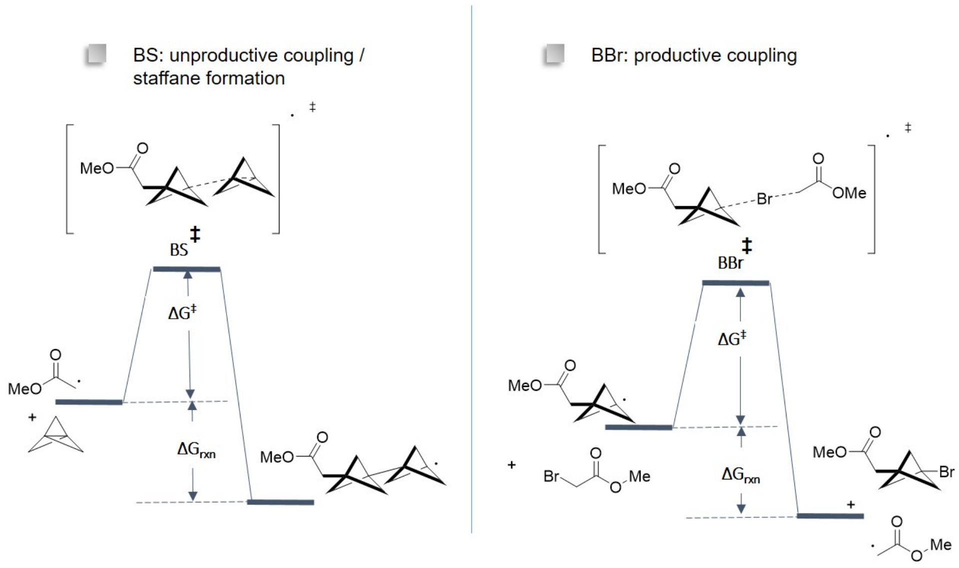

method	ΔG‡ (BS)	ΔG‡ (BBr)	ΔΔG‡ (BS - BBr)	ΔG_{rxn} (BS)	ΔG_{rxn} (BBr)
ROB3LYP	14.2	11.9	2.3	-20.7	-17.3
ROM062x	13.3	12.9	0.4	-21.4	-16.3
ROwb97xd	12.3	15.2	- 2.9	-29.6	-18.3

Fig Comp S3. Comparison of computed activation barriers and exergonicities for the propagation step (BBr) of the radical process and the sideproduct formation (BS) depending on the method employed. All numbers in kcal/mol.

Computed structures: Representation of calculated transition states

AB‡

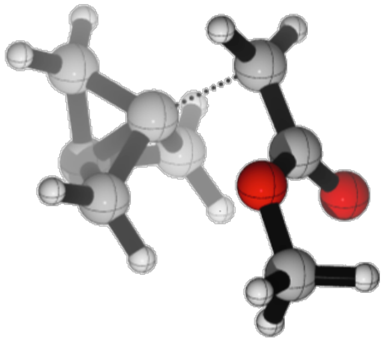

BS‡

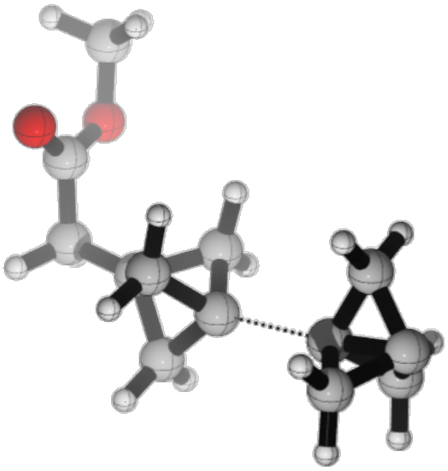

BI‡

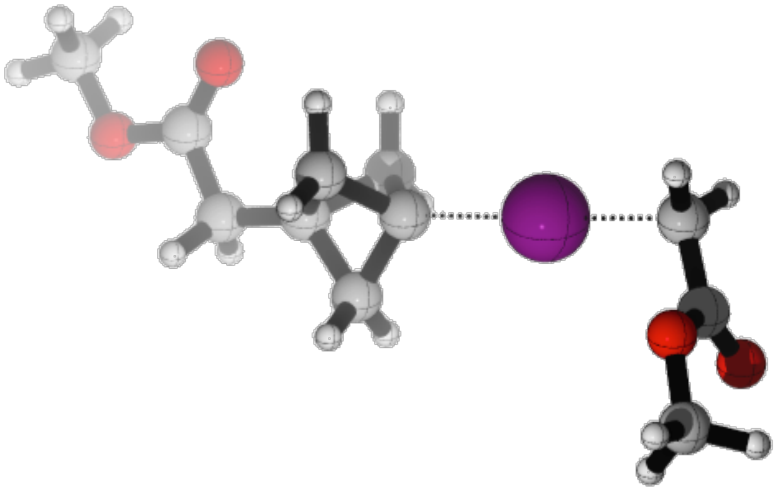

BBr‡

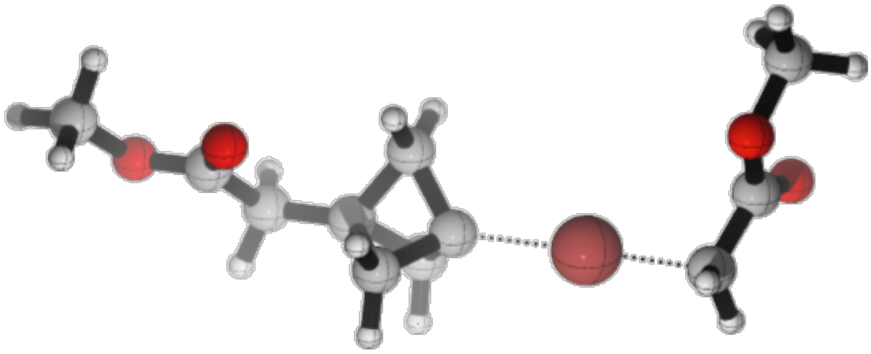

xyz-coordinates and energies of all computed structures

[(acetate-radical)]

i) (RO)B3LYP / def2tzvp

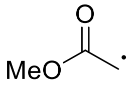

C	-1.784021000	-0.583499000	-0.000003000
H	-2.694796000	-0.005698000	-0.000115000
H	-1.816427000	-1.662338000	-0.000392000
C	-0.525261000	0.125935000	0.000094000
O	-0.411190000	1.335009000	-0.000017000
O	0.533004000	-0.722805000	0.000116000
C	1.824612000	-0.101265000	-0.000081000
H	2.542524000	-0.917028000	-0.000479000
H	1.950802000	0.520454000	-0.886480000
H	1.951406000	0.519959000	0.886614000

```
Zero-point correction=                           0.076066 (Hartree/Particle)
 Thermal correction to Energy=                    0.081850
 Thermal correction to Enthalpy=                  0.082794
 Thermal correction to Gibbs Free Energy=         0.046817
 Sum of electronic and zero-point Energies=           -267.761272
 Sum of electronic and thermal Energies=              -267.755488
 Sum of electronic and thermal Enthalpies=            -267.754544
 Sum of electronic and thermal Free Energies=         -267.790521

E[(RO)B3LYP/def2TZVP] = -267.837337601
```

ii) (RO)M06-2x / def2tzvp

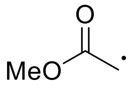

C	-1.786969000	-0.570050000	0.000001000
H	-2.687771000	0.020613000	-0.000071000
H	-1.824839000	-1.646983000	-0.000002000
C	-0.516315000	0.126350000	0.000004000
O	-0.389637000	1.327073000	0.000007000
O	0.526442000	-0.726214000	0.000015000
C	1.808247000	-0.106046000	-0.000015000
H	2.531575000	-0.915996000	-0.000205000
H	1.928330000	0.517134000	-0.885758000
H	1.928498000	0.516838000	0.885918000

```
Zero-point correction=                           0.077087 (Hartree/Particle)
 Thermal correction to Energy=                    0.082866
 Thermal correction to Enthalpy=                  0.083810
 Thermal correction to Gibbs Free Energy=         0.047694
 Sum of electronic and zero-point Energies=           -267.638577
 Sum of electronic and thermal Energies=              -267.632798
 Sum of electronic and thermal Enthalpies=            -267.631854
 Sum of electronic and thermal Free Energies=         -267.667970

E[(RO)M06-2x/def2TZVP] = -267.715663649
```

iii) (RO)ωB97xD / def2tzvp

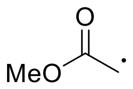

C	-1.781130000	-0.578062000	-0.000002000
H	-2.688672000	0.005155000	0.000015000
H	-1.816844000	-1.656978000	-0.000030000
C	-0.518224000	0.125866000	0.000001000
O	-0.402344000	1.329483000	-0.000002000
O	0.529320000	-0.719964000	0.000009000
C	1.812001000	-0.104547000	-0.000002000
H	2.532718000	-0.917955000	0.000096000
H	1.940591000	0.516951000	-0.886561000
H	1.940521000	0.517135000	0.886437000

```
Zero-point correction=                           0.076495 (Hartree/Particle)
 Thermal correction to Energy=                    0.082500
 Thermal correction to Enthalpy=                  0.083445
 Thermal correction to Gibbs Free Energy=         0.046240
 Sum of electronic and zero-point Energies=           -267.665908
 Sum of electronic and thermal Energies=              -267.659903
 Sum of electronic and thermal Enthalpies=            -267.658959
 Sum of electronic and thermal Free Energies=         -267.696164

E[(RO)wB97xD/def2TZVP] = -267.742403375
```

[tcp]

i) (RO)B3LYP / def2tzvp

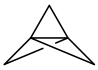

C	-0.854051000	-0.975927000	-0.000222000
C	0.000017000	-0.000052000	-0.782989000
H	-1.924401000	-0.811153000	-0.000193000
H	-0.548666000	-2.014944000	-0.000201000
C	1.272366000	-0.251483000	-0.000208000
H	2.019345000	0.532600000	-0.000464000
H	1.664902000	-1.260826000	-0.000242000
C	-0.418366000	1.227408000	-0.000209000
H	0.259239000	2.072138000	-0.000461000
H	-1.471011000	1.482035000	-0.000238000
C	0.000134000	0.000079000	0.783927000

```
Zero-point correction=                           0.093075 (Hartree/Particle)
 Thermal correction to Energy=                    0.097056
 Thermal correction to Enthalpy=                  0.098000
 Thermal correction to Gibbs Free Energy=         0.066775
 Sum of electronic and zero-point Energies=           -193.986565
 Sum of electronic and thermal Energies=              -193.982584
 Sum of electronic and thermal Enthalpies=            -193.981640
 Sum of electronic and thermal Free Energies=         -194.012865

E[(RO)B3LYP/def2TZVP] = -194.059299611
```

ii) (RO)M06-2x / def2tzvp

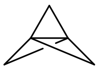

C	0.982367000	0.841060000	-0.000210000
C	-0.000020000	0.000306000	-0.771841000
H	2.015339000	0.518157000	-0.000123000
H	0.823687000	1.911601000	-0.000138000
C	-1.219685000	0.429944000	-0.000205000
H	-2.066783000	-0.243703000	-0.000360000
H	-1.457815000	1.485622000	-0.000211000
C	0.237405000	-1.271206000	-0.000182000
H	-0.557930000	-2.005105000	-0.000417000
H	1.244082000	-1.668463000	-0.000213000
C	-0.000164000	0.000211000	0.772681000

```
Zero-point correction=                           0.094352 (Hartree/Particle)
 Thermal correction to Energy=                    0.098244
 Thermal correction to Enthalpy=                  0.099188
 Thermal correction to Gibbs Free Energy=         0.068099
 Sum of electronic and zero-point Energies=           -193.908868
 Sum of electronic and thermal Energies=              -193.904976
 Sum of electronic and thermal Enthalpies=            -193.904032
 Sum of electronic and thermal Free Energies=         -193.935120

E[(RO)M06-2x/def2TZVP] = -194.003219431
```

iii) (RO)ωB97xD / def2tzvp

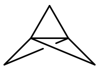

C	-1.282890000	0.179562000	-0.000290000
C	-0.000246000	0.000102000	-0.777903000
H	-1.730346000	1.166014000	-0.000053000
H	-1.983900000	-0.646214000	-0.000069000
C	0.485872000	-1.200435000	-0.000312000
H	1.551524000	-1.394590000	-0.000301000
H	-0.144437000	-2.081325000	-0.000137000
C	0.797169000	1.020823000	-0.000316000
H	1.875168000	0.914477000	-0.000327000
H	0.432654000	2.040857000	-0.000137000
C	-0.000015000	0.000078000	0.778992000

```
Zero-point correction=                           0.093688 (Hartree/Particle)
 Thermal correction to Energy=                    0.097653
 Thermal correction to Enthalpy=                  0.098597
 Thermal correction to Gibbs Free Energy=         0.067401
 Sum of electronic and zero-point Energies=           -193.919158
 Sum of electronic and thermal Energies=              -193.915194
 Sum of electronic and thermal Enthalpies=            -193.914249
 Sum of electronic and thermal Free Energies=         -193.945445

E[(RO)wB97xD/def2TZVP] = -194.012846414
```

[AB‡]

i) (RO)B3LYP / def2tzvp

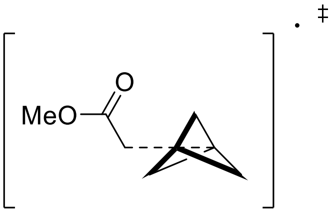

C	-2.755980000	-0.243405000	-0.770710000	C	0.456755000	-1.133583000	-1.035593000
C	-2.650210000	0.621741000	0.473956000	H	0.073840000	-2.124257000	-0.851664000
H	-3.239109000	-1.207675000	-0.664697000	H	0.362432000	-0.712655000	-2.024401000
H	-2.945601000	0.261061000	-1.710970000	C	1.499304000	-0.660756000	-0.155125000
C	-1.457388000	1.419566000	-0.027022000	O	1.814487000	-1.167542000	0.904452000
H	-0.822916000	1.884381000	0.717903000	O	2.088163000	0.475131000	-0.628297000
H	-1.589215000	1.993741000	-0.936408000	C	3.128525000	1.016987000	0.190629000
C	-1.815063000	-0.379186000	1.256224000	H	3.481965000	1.903921000	-0.329617000
H	-1.191090000	0.003609000	2.054343000	H	2.748907000	1.280947000	1.178196000
H	-2.254922000	-1.350556000	1.448503000	H	3.939859000	0.298606000	0.310600000
C	-1.370169000	-0.090003000	-0.165863000				

```
Zero-point correction=                           0.170754 (Hartree/Particle)
 Thermal correction to Energy=                    0.181244
 Thermal correction to Enthalpy=                  0.182188
 Thermal correction to Gibbs Free Energy=         0.132134
 Sum of electronic and zero-point Energies=           -461.742614
 Sum of electronic and thermal Energies=              -461.732123
 Sum of electronic and thermal Enthalpies=            -461.731179
 Sum of electronic and thermal Free Energies=         -461.781233

E[(RO)B3LYP/def2TZVP] = -461.913367074
```

ii) (RO)M06-2x / def2tzvp

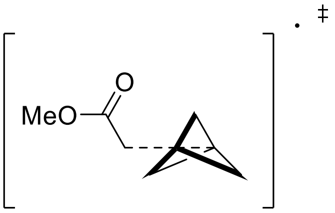

C	-2.667640000	0.095105000	-0.831862000	C	0.395433000	-1.270931000	-1.036490000
C	-2.407148000	0.716932000	0.522817000	H	0.004120000	-2.241892000	-0.779320000
H	-3.332589000	-0.759192000	-0.860725000	H	0.362051000	-0.938246000	-2.062119000
H	-2.732258000	0.767720000	-1.678222000	C	1.370938000	-0.695950000	-0.139263000
C	-1.080352000	1.331177000	0.138857000	O	1.573138000	-1.049283000	1.001459000
H	-0.376917000	1.526338000	0.939882000	O	2.002775000	0.367181000	-0.690205000
H	-1.079612000	2.047782000	-0.672914000	C	2.934469000	1.018817000	0.162328000
C	-1.809191000	-0.528466000	1.136782000	H	3.364946000	1.825890000	-0.423872000
H	-1.121216000	-0.402170000	1.963129000	H	2.435277000	1.414947000	1.047350000
H	-2.440043000	-1.407969000	1.178713000	H	3.710152000	0.324903000	0.485228000
C	-1.303376000	-0.116900000	-0.224363000				

```
Zero-point correction=                           0.172849 (Hartree/Particle)
 Thermal correction to Energy=                    0.183156
 Thermal correction to Enthalpy=                  0.184100
 Thermal correction to Gibbs Free Energy=         0.134789
 Sum of electronic and zero-point Energies=           -461.545031
 Sum of electronic and thermal Energies=              -461.534723
 Sum of electronic and thermal Enthalpies=            -461.533779
 Sum of electronic and thermal Free Energies=         -461.583090

E[(RO)M06-2x/def2TZVP] = -461.717879384
```

iii) (RO)ωB97xD / def2tzvp

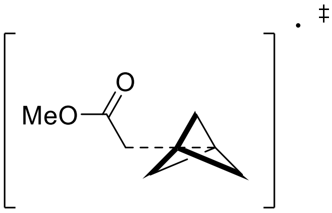

C	-1.707527000	-0.366162000	1.245967000
C	-2.507398000	0.687686000	0.501248000
H	-1.010543000	-0.029288000	2.003577000
H	-2.204287000	-1.303906000	1.465502000
C	-2.736405000	-0.172423000	-0.728877000
H	-2.936051000	0.339758000	-1.663035000
H	-3.278025000	-1.100970000	-0.589829000
C	-1.289072000	1.394130000	-0.064401000
H	-1.423473000	1.971098000	-0.971799000
H	-0.582388000	1.810154000	0.644183000
C	-1.321172000	-0.113520000	-0.194871000
C	0.417958000	-1.215447000	-1.035002000
H	0.039324000	-2.200749000	-0.814003000
H	0.353138000	-0.843398000	-2.046053000
C	1.425815000	-0.684639000	-0.146557000
O	1.687828000	-1.113382000	0.955355000
O	2.023178000	0.414017000	-0.662462000
C	2.991304000	1.031144000	0.172717000
H	3.388776000	1.867405000	-0.397336000
H	2.536032000	1.388298000	1.097832000
H	3.788439000	0.331905000	0.426470000

```
Zero-point correction=                           0.172294 (Hartree/Particle)
 Thermal correction to Energy=                    0.182709
 Thermal correction to Enthalpy=                  0.183654
 Thermal correction to Gibbs Free Energy=         0.133907
 Sum of electronic and zero-point Energies=           -461.583156
 Sum of electronic and thermal Energies=              -461.572741
 Sum of electronic and thermal Enthalpies=            -461.571797
 Sum of electronic and thermal Free Energies=         -461.621543
```

E[(RO)wB97xD/def2TZVP] = -461.755449934

[B]

i) (RO)B3LYP / def2tzvp

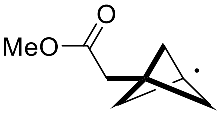

C	-2.613536000	-0.002947000	0.790519000
C	-2.590460000	-0.479962000	-0.673605000
H	-3.103730000	0.947226000	0.994542000
H	-2.810514000	-0.753289000	1.554279000
C	-1.341647000	-1.335304000	-0.384473000
H	-0.701550000	-1.576552000	-1.230576000
H	-1.458863000	-2.168697000	0.305711000
C	-1.695729000	0.713770000	-1.058786000
H	-1.074388000	0.606350000	-1.944681000
H	-2.128400000	1.708088000	-0.967531000
C	-1.152428000	0.073354000	0.253515000
C	0.050151000	0.554242000	1.074538000
H	-0.127924000	1.583546000	1.386894000
H	0.154594000	-0.078232000	1.956173000
C	1.322630000	0.530690000	0.262920000
O	1.676927000	1.402828000	-0.489139000
O	2.011317000	-0.613177000	0.453150000
C	3.219697000	-0.752651000	-0.312077000
H	3.627525000	-1.722359000	-0.040416000
H	3.003168000	-0.712428000	-1.379020000
H	3.922058000	0.041982000	-0.062763000

```
Zero-point correction=                           0.174844 (Hartree/Particle)
 Thermal correction to Energy=                    0.184674
 Thermal correction to Enthalpy=                  0.185618
 Thermal correction to Gibbs Free Energy=         0.137500
 Sum of electronic and zero-point Energies=           -461.777615
 Sum of electronic and thermal Energies=              -461.767785
 Sum of electronic and thermal Enthalpies=            -461.766841
 Sum of electronic and thermal Free Energies=         -461.814959
```

E[(RO)B3LYP/def2TZVP] = -461.952458382

ii) (RO)M06-2x / def2tzvp

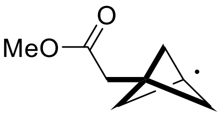

C	-2.594142000	-0.051705000	0.758421000
C	-2.492753000	-0.534778000	-0.693654000
H	-3.136248000	0.874758000	0.932793000
H	-2.771077000	-0.808834000	1.519085000
C	-1.219221000	-1.320927000	-0.359064000
H	-0.536350000	-1.524510000	-1.181136000

H	-1.316442000	-2.149788000	0.338178000
C	-1.655104000	0.695720000	-1.061938000
H	-0.992532000	0.609985000	-1.919317000
H	-2.142026000	1.665284000	-0.987564000
C	-1.135696000	0.092800000	0.265225000
C	0.028760000	0.634432000	1.092021000
H	-0.160190000	1.678857000	1.336937000
H	0.132354000	0.046769000	2.003238000
C	1.284994000	0.554937000	0.269077000
O	1.625906000	1.370301000	-0.543078000
O	1.959781000	-0.579791000	0.501867000
C	3.119325000	-0.774561000	-0.305376000
H	3.539658000	-1.727351000	0.002016000
H	2.846543000	-0.795921000	-1.359909000
H	3.833846000	0.031158000	-0.142904000

```
Zero-point correction=                           0.177225 (Hartree/Particle)
 Thermal correction to Energy=                    0.186784
 Thermal correction to Enthalpy=                  0.187728
 Thermal correction to Gibbs Free Energy=         0.140427
 Sum of electronic and zero-point Energies=           -461.581379
 Sum of electronic and thermal Energies=              -461.571820
 Sum of electronic and thermal Enthalpies=            -461.570875
 Sum of electronic and thermal Free Energies=         -461.618177

E[(RO)M06-2x/def2TZVP] = -461.758603302
```

iii) (RO)ωB97xD / def2tzvp

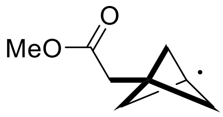

C	-2.601959000	0.037165000	0.753267000
C	-2.531277000	-0.555580000	-0.665359000
H	-3.117558000	0.989187000	0.868105000
H	-2.804220000	-0.654863000	1.569314000
C	-1.272188000	-1.347159000	-0.269953000
H	-0.596665000	-1.637597000	-1.072599000
H	-1.388051000	-2.124553000	0.483328000
C	-1.649825000	0.623089000	-1.113214000
H	-0.995532000	0.462053000	-1.967496000
H	-2.105498000	1.611503000	-1.113710000
C	-1.132999000	0.106691000	0.256670000
C	0.036733000	0.675688000	1.046097000
H	-0.148299000	1.730027000	1.248749000
H	0.135411000	0.133678000	1.986424000
C	1.307389000	0.567856000	0.245339000
O	1.692825000	1.384902000	-0.545736000
O	1.943232000	-0.585897000	0.487197000
C	3.123363000	-0.816018000	-0.277394000
H	3.501396000	-1.782697000	0.044386000
H	2.893499000	-0.832188000	-1.342858000
H	3.861641000	-0.036984000	-0.088048000

```
Zero-point correction=                           0.177171 (Hartree/Particle)
 Thermal correction to Energy=                    0.186874
 Thermal correction to Enthalpy=                  0.187818
 Thermal correction to Gibbs Free Energy=         0.139896
 Sum of electronic and zero-point Energies=           -461.630485
 Sum of electronic and thermal Energies=              -461.620782
 Sum of electronic and thermal Enthalpies=            -461.619838
 Sum of electronic and thermal Free Energies=         -461.667759

E[(RO)wB97xD/def2TZVP] = -461.807655339
```

[BS‡]

i) (RO)B3LYP / def2tzvp

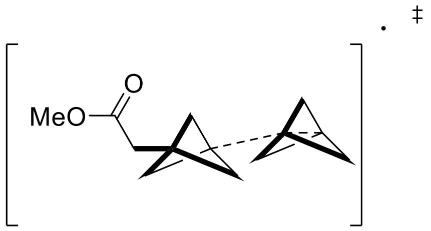

C	-0.123323000	-0.528785000	1.089402000
C	-0.260010000	0.378246000	-0.887578000
C	0.284815000	-1.721282000	-0.687771000
H	0.052765000	-1.359189000	1.771231000
H	-0.400663000	0.385608000	1.609499000
C	-0.918152000	-0.857669000	-0.207049000
H	-0.205604000	0.362233000	-1.975191000
H	-0.539167000	1.355733000	-0.497768000
H	0.374619000	-1.872480000	-1.762763000
H	0.488223000	-2.627237000	-0.118389000
C	-2.388027000	-1.283596000	-0.296826000
C	0.831330000	-0.395788000	-0.116704000
C	3.598231000	1.671021000	0.278118000
C	4.730324000	0.657321000	0.131616000
C	4.094950000	-0.355411000	1.080589000
C	4.142987000	0.016785000	-1.123322000
H	3.460941000	2.378108000	-0.531681000
H	3.421292000	2.074265000	1.268430000

H	4.385322000	-1.392833000	0.961829000	C	-3.307385000	-0.232624000	0.274242000
H	3.940056000	-0.041763000	2.106339000	O	-3.545527000	-0.087111000	1.446708000
H	4.435500000	-1.004162000	-1.339646000	O	-3.813926000	0.563886000	-0.690437000
H	4.029793000	0.651360000	-1.994628000	C	-4.669342000	1.626444000	-0.240113000
C	3.190428000	0.238749000	0.027336000	H	-4.131343000	2.290979000	0.435121000
H	-2.524228000	-2.196613000	0.283914000	H	-4.973559000	2.157744000	-1.137808000
H	-2.641370000	-1.478669000	-1.339043000	H	-5.537914000	1.222250000	0.278743000

```
Zero-point correction=                           0.268150 (Hartree/Particle)
 Thermal correction to Energy=                    0.283161
 Thermal correction to Enthalpy=                  0.284106
 Thermal correction to Gibbs Free Energy=         0.221141
 Sum of electronic and zero-point Energies=           -655.758218
 Sum of electronic and thermal Energies=              -655.743206
 Sum of electronic and thermal Enthalpies=            -655.742262
 Sum of electronic and thermal Free Energies=         -655.805227

E[(RO)B3LYP/def2TZVP] = -656.026366956
```

ii) (RO)M06-2x / def2tzvp

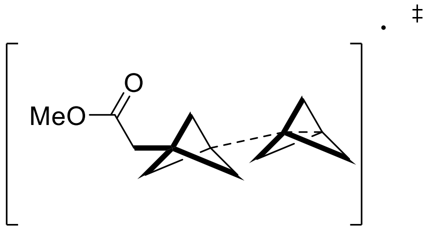

C	0.072638000	0.700412000	1.076908000	H	-2.787988000	-2.387800000	-0.285303000
C	0.224414000	-0.123736000	-0.927191000	H	-2.864689000	-1.858927000	1.467118000
C	-0.210389000	1.989921000	-0.653435000	H	-4.533203000	1.240616000	0.714124000
H	-0.079552000	1.510464000	1.787071000	H	-3.838044000	0.165339000	2.024025000
H	0.286737000	-0.253470000	1.554186000	H	-4.436572000	0.572176000	-1.503998000
C	0.912136000	1.040512000	-0.176027000	H	-3.665240000	-1.030012000	-1.943851000
H	0.208418000	-0.055070000	-2.013126000	C	-3.008052000	-0.201336000	0.003065000
H	0.441556000	-1.126607000	-0.562779000	H	2.607803000	2.187903000	0.483030000
H	-0.253724000	2.183990000	-1.723292000	H	2.714944000	1.609712000	-1.204614000
H	-0.383145000	2.876125000	-0.046196000	C	3.160950000	0.151597000	0.298680000
C	2.407110000	1.356003000	-0.190826000	O	3.375959000	-0.104193000	1.451879000
C	-0.837912000	0.674958000	-0.158991000	O	3.516603000	-0.655528000	-0.712099000
C	-3.093634000	-1.635696000	0.432031000	C	4.159803000	-1.867166000	-0.323889000
C	-4.403179000	-0.914572000	0.157173000	H	3.503192000	-2.456410000	0.315366000
C	-4.031334000	0.315388000	0.968917000	H	4.376273000	-2.399001000	-1.245455000
C	-3.938481000	-0.328010000	-1.165521000	H	5.078321000	-1.650905000	0.220093000

```
Zero-point correction=                           0.271257 (Hartree/Particle)
 Thermal correction to Energy=                    0.285966
 Thermal correction to Enthalpy=                  0.286910
 Thermal correction to Gibbs Free Energy=         0.224541
 Sum of electronic and zero-point Energies=           -655.485306
 Sum of electronic and thermal Energies=              -655.470598
 Sum of electronic and thermal Enthalpies=            -655.469654
 Sum of electronic and thermal Free Energies=         -655.532022

E[(RO)M06-2x/def2TZVP] = -655.756563332
```

iii) (RO)ωB97xD / def2tzvp

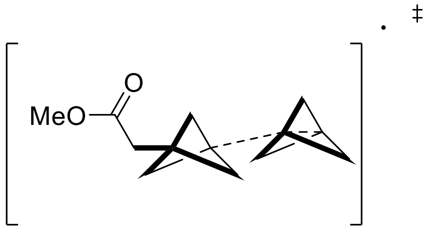

C	-0.155763000	-0.521021000	1.078746000	H	0.036361000	-1.364809000	1.739959000
C	-0.284290000	0.439139000	-0.861537000	H	-0.481305000	0.365885000	1.619514000
C	0.333134000	-1.634455000	-0.716694000	C	-0.909725000	-0.837960000	-0.239997000

H	-0.207345000	0.462288000	-1.947912000
H	-0.613395000	1.390539000	-0.445881000
H	0.452904000	-1.748416000	-1.793665000
H	0.558630000	-2.550596000	-0.171713000
C	-2.354097000	-1.297219000	-0.367099000
C	0.819816000	-0.312772000	-0.094828000
C	3.604785000	1.657502000	0.293478000
C	4.677727000	0.583053000	0.141377000
C	3.989983000	-0.390231000	1.094389000
C	4.044483000	-0.024725000	-1.106825000
H	3.502597000	2.370270000	-0.516786000
H	3.457207000	2.070512000	1.284730000
H	4.217009000	-1.443097000	0.972127000
H	3.857772000	-0.067203000	2.120546000
H	4.273871000	-1.062304000	-1.320818000
H	3.961234000	0.614337000	-1.978457000
C	3.124126000	0.254806000	0.048429000
H	-2.480648000	-2.235244000	0.172744000
H	-2.591909000	-1.447394000	-1.420222000
C	-3.282301000	-0.279295000	0.238607000
O	-3.589895000	-0.234862000	1.398878000
O	-3.695102000	0.615904000	-0.669113000
C	-4.521310000	1.664403000	-0.171154000
H	-3.990843000	2.243506000	0.584903000
H	-4.760228000	2.287420000	-1.028933000
H	-5.431339000	1.258618000	0.270389000

Zero-point correction= 0.271246 (Hartree/Particle)
Thermal correction to Energy= 0.286012
Thermal correction to Enthalpy= 0.286956
Thermal correction to Gibbs Free Energy= 0.224872
Sum of electronic and zero-point Energies= -655.547191
Sum of electronic and thermal Energies= -655.532425
Sum of electronic and thermal Enthalpies= -655.531481
Sum of electronic and thermal Free Energies= -655.593565

E[(RO)wB97xD/def2TZVP] = -655.818436833

[BS]

i) (RO)B3LYP / def2tzvp

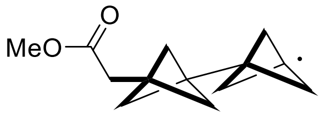

C	-0.603504000	1.654603000	-0.000021000
C	-0.170167000	-0.154611000	1.074570000
C	-0.170167000	-0.154654000	-1.074541000
H	-0.746468000	2.249991000	-0.903869000
H	-0.746471000	2.250025000	0.903804000
C	0.598445000	0.667617000	-0.000002000
H	0.091362000	-1.208573000	1.153208000
H	-0.284726000	0.313992000	2.054176000
H	0.091364000	-1.208619000	-1.153138000
H	-0.284728000	0.313910000	-2.054165000
C	2.049819000	1.067715000	-0.000014000
C	-1.229468000	0.229236000	0.000006000
C	-3.729355000	0.281980000	1.092741000
C	-4.443381000	-0.537268000	0.000004000
C	-3.729313000	0.281825000	-1.092819000
C	-3.289636000	-1.558610000	0.000099000
H	-3.613279000	-0.188225000	2.067510000
H	-3.978975000	1.339276000	1.161484000
H	-3.613198000	-0.188517000	-2.067518000
H	-3.978931000	1.339112000	-1.161722000
H	-3.145310000	-2.143444000	-0.906368000
H	-3.145347000	-2.143318000	0.906653000
C	-2.687482000	-0.117537000	0.000009000
H	2.274992000	1.690891000	0.871321000
H	2.274970000	1.690905000	-0.871345000
C	3.027288000	-0.088017000	-0.000045000
O	2.745181000	-1.258635000	-0.000068000
O	4.297343000	0.367759000	-0.000021000
C	5.326738000	-0.633820000	0.000079000
H	6.265947000	-0.087439000	0.000326000
H	5.249669000	-1.261972000	0.886986000
H	5.250023000	-1.261752000	-0.887017000

Zero-point correction= 0.271414 (Hartree/Particle)
Thermal correction to Energy= 0.285622
Thermal correction to Enthalpy= 0.286566
Thermal correction to Gibbs Free Energy= 0.227498
Sum of electronic and zero-point Energies= -655.816823
Sum of electronic and thermal Energies= -655.802615
Sum of electronic and thermal Enthalpies= -655.801671
Sum of electronic and thermal Free Energies= -655.860740

E[(RO)B3LYP/def2TZVP] = -656.088237183

ii) (RO)M06-2x / def2tzvp

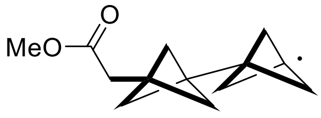

C	0.616854000	1.675401000	0.000101000
C	0.153187000	-0.122481000	-1.071170000
C	0.153251000	-0.122554000	1.071279000
H	0.770889000	2.261775000	0.907214000
H	0.770839000	2.261837000	-0.906981000
C	-0.591385000	0.710184000	0.000104000
H	-0.128148000	-1.171860000	-1.139916000
H	0.278833000	0.347602000	-2.047902000
H	-0.128081000	-1.171936000	1.139975000
H	0.278958000	0.347466000	2.048034000
C	-2.038554000	1.111101000	0.000124000
C	1.205614000	0.245691000	0.000034000

C	3.685084000	0.254462000	-1.090632000
C	4.374372000	-0.576361000	-0.000159000
C	3.685160000	0.254207000	1.090556000
C	3.208653000	-1.573422000	-0.000238000
H	3.554558000	-0.217555000	-2.061797000
H	3.953153000	1.306934000	-1.151374000
H	3.554701000	-0.218034000	2.061620000
H	3.953235000	1.306664000	1.151524000
H	3.050148000	-2.147686000	0.909671000
H	3.050087000	-2.147467000	-0.910274000
C	2.652635000	-0.127218000	-0.000046000
H	-2.273473000	1.724750000	-0.874027000
H	-2.273463000	1.724807000	0.874238000
C	-2.980302000	-0.067005000	0.000169000
O	-2.659204000	-1.223461000	0.000406000
O	-4.258572000	0.338227000	-0.000208000
C	-5.230524000	-0.704961000	-0.000255000
H	-6.197478000	-0.210777000	-0.001249000
H	-5.117768000	-1.329020000	-0.885993000
H	-5.119042000	-1.327887000	0.886445000

```
Zero-point correction=                           0.274379 (Hartree/Particle)
 Thermal correction to Energy=                    0.287592
 Thermal correction to Enthalpy=                  0.288536
 Thermal correction to Gibbs Free Energy=         0.232642
 Sum of electronic and zero-point Energies=           -655.545620
 Sum of electronic and thermal Energies=              -655.532406
 Sum of electronic and thermal Enthalpies=            -655.531462
 Sum of electronic and thermal Free Energies=         -655.587357

E[(RO)M06-2x/def2TZVP] = -655.819998096
```

iii) (RO)ωB97xD / def2tzvp

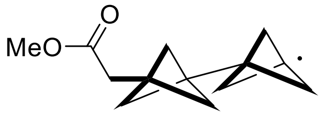

C	0.608204000	1.662390000	0.000240000
C	0.162307000	-0.136583000	-1.070174000
C	0.162224000	-0.136983000	1.069945000
H	0.757574000	2.254545000	0.905172000
H	0.757653000	2.254889000	-0.904454000
C	-0.597948000	0.688878000	0.000009000
H	-0.107885000	-1.188779000	-1.147344000
H	0.282670000	0.331945000	-2.049168000
H	-0.107982000	-1.189203000	1.146713000
H	0.282517000	0.331194000	2.049116000
C	-2.043983000	1.086193000	0.000028000
C	1.218143000	0.237633000	-0.000003000
C	3.702161000	0.267218000	-1.088219000
C	4.413084000	-0.557709000	-0.000019000
C	3.701960000	0.266372000	1.088684000
C	3.245449000	-1.561255000	-0.000521000
H	3.576820000	-0.202003000	-2.062639000
H	3.957623000	1.323364000	-1.157909000
H	3.576438000	-0.203586000	2.062725000
H	3.957411000	1.322463000	1.159234000
H	3.091007000	-2.143030000	0.906524000
H	3.091177000	-2.142313000	-0.908054000
C	2.662458000	-0.120363000	-0.000012000
H	-2.272002000	1.704538000	-0.872823000
H	-2.271983000	1.704490000	0.872918000
C	-3.001050000	-0.080154000	0.000031000
O	-2.699284000	-1.242190000	0.000070000
O	-4.270335000	0.349670000	-0.000070000
C	-5.270682000	-0.664434000	-0.000002000
H	-6.223235000	-0.141340000	-0.000496000
H	-5.182133000	-1.292476000	-0.886512000
H	-5.182679000	-1.291759000	0.887074000

```
Zero-point correction=                           0.274507 (Hartree/Particle)
 Thermal correction to Energy=                    0.286840
 Thermal correction to Enthalpy=                  0.287784
 Thermal correction to Gibbs Free Energy=         0.234565
 Sum of electronic and zero-point Energies=           -655.620456
 Sum of electronic and thermal Energies=              -655.608123
 Sum of electronic and thermal Enthalpies=            -655.607179
 Sum of electronic and thermal Free Energies=         -655.660398

E[(RO)wB97xD/def2TZVP] = -655.894962524
```

[Iodoacetate]

i) (RO)B3LYP / def2tzvp

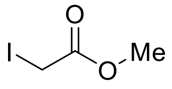

C	0.261788000	0.873644000	0.850178000
H	0.027020000	1.929333000	0.793573000
H	0.286141000	0.512773000	1.872432000
C	1.539972000	0.587545000	0.110722000
O	2.022562000	1.317088000	-0.716244000
O	2.081360000	-0.577116000	0.502471000
C	3.291703000	-0.964454000	-0.170615000
H	3.578179000	-1.914596000	0.270840000
H	3.110244000	-1.076006000	-1.238915000
H	4.068953000	-0.217349000	-0.015322000
I	-1.404956000	-0.153478000	-0.088946000

```
Zero-point correction=                           0.080216 (Hartree/Particle)
 Thermal correction to Energy=                    0.087324
 Thermal correction to Enthalpy=                  0.088269
 Thermal correction to Gibbs Free Energy=         0.046202
 Sum of electronic and zero-point Energies=           -565.617726
 Sum of electronic and thermal Energies=              -565.610617
 Sum of electronic and thermal Enthalpies=            -565.609673
 Sum of electronic and thermal Free Energies=         -565.651740

E[(RO)B3LYP/def2TZVP] = -565.697941736
```

ii) (RO)M06-2x / def2tzvp

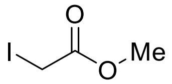

C	0.236084000	0.902007000	0.837221000
H	0.008632000	1.957034000	0.747974000
H	0.264180000	0.570470000	1.869259000
C	1.512790000	0.589780000	0.109713000
O	2.000535000	1.292712000	-0.729372000
O	2.038196000	-0.567904000	0.517691000
C	3.229944000	-0.965973000	-0.159871000
H	3.524338000	-1.910052000	0.287721000
H	3.031816000	-1.088509000	-1.223865000
H	4.007819000	-0.215708000	-0.026847000
I	-1.377727000	-0.155973000	-0.088362000

```
Zero-point correction=                           0.081521 (Hartree/Particle)
 Thermal correction to Energy=                    0.088456
 Thermal correction to Enthalpy=                  0.089400
 Thermal correction to Gibbs Free Energy=         0.047799
 Sum of electronic and zero-point Energies=           -565.329021
 Sum of electronic and thermal Energies=              -565.322086
 Sum of electronic and thermal Enthalpies=            -565.321141
 Sum of electronic and thermal Free Energies=         -565.362743

E[(RO)M06-2x/def2TZVP] = -565.410541279
```

iii) (RO)ωB97xD / def2tzvp

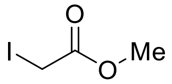

C	0.243274000	0.865308000	0.847854000
H	0.019407000	1.924658000	0.797810000
H	0.279234000	0.508343000	1.872059000
C	1.524623000	0.583672000	0.112011000
O	2.001523000	1.309743000	-0.715030000
O	2.065876000	-0.571064000	0.503466000
C	3.263565000	-0.955221000	-0.169496000
H	3.565183000	-1.896484000	0.281075000
H	3.074768000	-1.085412000	-1.234909000
H	4.037524000	-0.200195000	-0.034177000
I	-1.390643000	-0.153262000	-0.089275000

```
Zero-point correction=                           0.081345 (Hartree/Particle)
 Thermal correction to Energy=                    0.088305
 Thermal correction to Enthalpy=                  0.089249
 Thermal correction to Gibbs Free Energy=         0.047648
 Sum of electronic and zero-point Energies=           -565.508705
 Sum of electronic and thermal Energies=              -565.501745
 Sum of electronic and thermal Enthalpies=            -565.500801
 Sum of electronic and thermal Free Energies=         -565.542402

E[(RO)wB97xD/def2TZVP] = -565.590049775
```

[BI‡]

i) (RO)B3LYP / def2tzvp

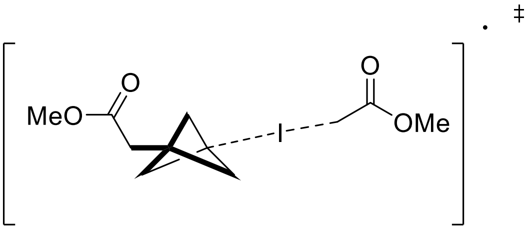

C	1.350227000	1.670812000	-0.227085000
C	1.793833000	-0.059341000	1.039972000
C	1.934343000	-0.232927000	-1.138820000
H	1.243819000	2.172862000	-1.186365000
H	1.125456000	2.315651000	0.619737000
C	2.573874000	0.712462000	-0.071570000
H	2.090601000	-1.090102000	1.205511000
H	1.593136000	0.487064000	1.959180000
H	2.238971000	-1.274004000	-1.100904000
H	1.859268000	0.160718000	-2.150452000
C	4.017702000	1.176289000	-0.016073000
C	0.859067000	0.219824000	-0.143544000
I	-1.620738000	-0.475715000	-0.227394000
C	-3.793672000	-1.247033000	-0.296694000
H	-3.740857000	-2.187384000	0.235987000
H	-3.985185000	-1.351005000	-1.357516000
C	-4.673786000	-0.271212000	0.388466000
O	-5.103474000	0.690940000	-0.459860000
O	-4.978666000	-0.309850000	1.556760000
C	-5.930453000	1.707407000	0.120987000
H	-6.174484000	2.386547000	-0.691734000
H	-6.837207000	1.272123000	0.540637000
H	-5.393291000	2.233276000	0.910214000
H	4.265398000	1.740740000	-0.919090000
H	4.157503000	1.867088000	0.819874000
C	5.024701000	0.053872000	0.128370000
O	4.770360000	-1.120000000	0.205778000
O	6.275355000	0.550613000	0.160160000
C	7.334621000	-0.413197000	0.295749000
H	7.324919000	-1.109655000	-0.541731000
H	8.254507000	0.164543000	0.302316000
H	7.225217000	-0.970931000	1.224979000

```
Zero-point correction=                           0.254579 (Hartree/Particle)
 Thermal correction to Energy=                    0.273152
 Thermal correction to Enthalpy=                  0.274097
 Thermal correction to Gibbs Free Energy=         0.200411
 Sum of electronic and zero-point Energies=          -1027.400773
 Sum of electronic and thermal Energies=             -1027.382199
 Sum of electronic and thermal Enthalpies=           -1027.381255
 Sum of electronic and thermal Free Energies=        -1027.454941

E[(RO)B3LYP/def2TZVP] = -1027.65535121
```

ii) (RO)M06-2x / def2tzvp

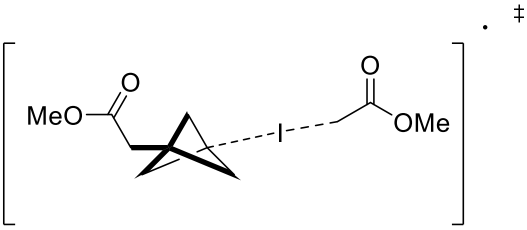

C	1.201112000	1.488620000	-0.673013000
C	0.778119000	0.123476000	-0.136718000
H	1.050705000	1.662303000	-1.735703000
H	0.970748000	2.349606000	-0.050291000
C	1.743138000	0.244368000	1.040475000
H	2.089696000	-0.680225000	1.491077000
H	1.541505000	1.041537000	1.752109000
C	1.837963000	-0.578551000	-0.980890000
H	2.188411000	-1.543803000	-0.629904000
H	1.719180000	-0.510307000	-2.059687000
C	2.451541000	0.662003000	-0.276950000
C	3.877833000	1.148295000	-0.408406000
H	4.101540000	1.392289000	-1.449619000
H	4.022633000	2.066428000	0.165674000
C	4.888128000	0.131507000	0.063889000
O	4.634302000	-0.958504000	0.495563000
O	6.133156000	0.604627000	-0.061429000
C	7.170354000	-0.281919000	0.359260000
H	8.102009000	0.247997000	0.187084000
H	7.054731000	-0.524093000	1.414752000
H	7.138894000	-1.202710000	-0.221359000
I	-1.561752000	-0.574282000	0.020625000
C	-3.735871000	-1.288839000	0.103147000
H	-3.844076000	-1.664883000	1.112748000
H	-3.795621000	-2.044295000	-0.669319000
C	-4.580384000	-0.112713000	-0.202056000
O	-4.973061000	0.192739000	-1.297381000
O	-4.848018000	0.607331000	0.901616000
C	-5.597335000	1.796484000	0.677664000
H	-5.727694000	2.255613000	1.653297000
H	-5.055127000	2.464284000	0.008608000
H	-6.563293000	1.561255000	0.232049000

```
Zero-point correction=                           0.258004 (Hartree/Particle)
 Thermal correction to Energy=                    0.276176
 Thermal correction to Enthalpy=                  0.277120
 Thermal correction to Gibbs Free Energy=         0.205009
 Sum of electronic and zero-point Energies=          -1026.916350
```

```
 Sum of electronic and thermal Energies=             -1026.898178
 Sum of electronic and thermal Enthalpies=           -1026.897234
 Sum of electronic and thermal Free Energies=        -1026.969344

E[(RO)M06-2x/def2TZVP] = -1027.17435372
```

iii) (RO)ωB97xD / def2tzvp

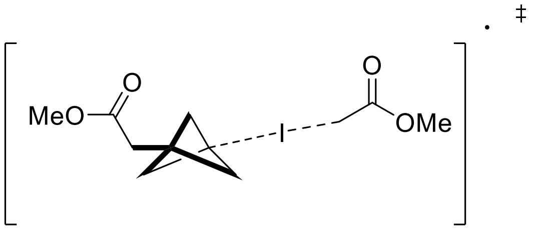

C	-1.693912000	-2.047769000	0.359729000
C	-0.871985000	-0.801777000	0.011811000
H	-1.671296000	-2.865149000	-0.358510000
H	-1.655206000	-2.387689000	1.392988000
C	-1.774046000	0.050606000	0.913332000
H	-1.822612000	1.115160000	0.693127000
H	-1.740945000	-0.158926000	1.980501000
C	-1.795038000	-0.521807000	-1.185210000
H	-1.851249000	0.505101000	-1.538633000
H	-1.781463000	-1.248145000	-1.994966000
C	-2.670817000	-0.880265000	0.048355000
C	-4.185293000	-0.960783000	0.080155000
H	-4.520694000	-1.692498000	-0.654220000
H	-4.509633000	-1.265930000	1.074720000
C	-4.784092000	0.374793000	-0.280139000
O	-4.968071000	0.759960000	-1.402345000
O	-5.046879000	1.106072000	0.808700000
C	-5.565727000	2.412578000	0.568510000
H	-5.691397000	2.866505000	1.547573000
H	-4.872388000	2.996001000	-0.036951000
H	-6.522802000	2.352740000	0.050737000
I	1.524924000	-0.678817000	-0.074857000
C	3.791511000	-0.849946000	0.282429000
H	4.142558000	-1.382127000	-0.594338000
H	3.877780000	-1.411421000	1.203989000
C	4.335846000	0.520611000	0.418777000
O	4.493847000	1.100968000	1.461543000
O	4.623081000	1.057597000	-0.777990000
C	5.100472000	2.397284000	-0.755698000
H	5.274739000	2.665428000	-1.794478000
H	4.359657000	3.062103000	-0.310748000
H	6.026620000	2.468213000	-0.184919000

```
Zero-point correction=                           0.258120 (Hartree/Particle)
 Thermal correction to Energy=                    0.276280
 Thermal correction to Enthalpy=                  0.277224
 Thermal correction to Gibbs Free Energy=         0.204406
 Sum of electronic and zero-point Energies=          -1027.142026
 Sum of electronic and thermal Energies=             -1027.123867
 Sum of electronic and thermal Enthalpies=           -1027.122923
 Sum of electronic and thermal Free Energies=        -1027.195741

E[(RO)wB97xD/def2TZVP] = -1027.40014630
```

[BI]

i) (RO)B3LYP / def2tzvp

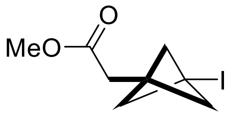

C	0.258404000	1.693043000	-0.414691000
C	0.787795000	0.293287000	-0.037303000
H	0.493893000	2.502205000	0.275539000
H	0.369446000	1.990139000	-1.457029000
C	-0.328285000	-0.329222000	-0.903724000
H	-0.620334000	-1.348682000	-0.655830000
H	-0.255522000	-0.167030000	-1.978499000
C	-0.180718000	0.283694000	1.165201000
H	-0.472235000	-0.689049000	1.553793000
H	0.024158000	1.001832000	1.957979000
C	-0.979078000	0.813070000	-0.063795000
C	-2.423917000	1.269698000	-0.102076000
H	-2.556371000	2.090656000	0.607425000
H	-2.674214000	1.633748000	-1.098437000
C	-3.381025000	0.168324000	0.300473000
O	-3.401627000	-0.360991000	1.382359000
O	-4.212506000	-0.162454000	-0.706163000
C	-5.159884000	-1.206257000	-0.420685000
H	-5.734901000	-1.335673000	-1.333245000
H	-4.642721000	-2.128331000	-0.158349000
H	-5.808893000	-0.915416000	0.404417000
I	2.853415000	-0.308692000	-0.012599000

```
Zero-point correction=                           0.177214 (Hartree/Particle)
 Thermal correction to Energy=                    0.187789
 Thermal correction to Enthalpy=                  0.188733
 Thermal correction to Gibbs Free Energy=         0.138625
 Sum of electronic and zero-point Energies=           -759.659621
 Sum of electronic and thermal Energies=              -759.649045
 Sum of electronic and thermal Enthalpies=            -759.648101
```

```
 Sum of electronic and thermal Free Energies=         -759.698209

E[(RO)B3LYP/def2TZVP] = -759.836834643
```

ii) (RO)M06-2x / def2tzvp

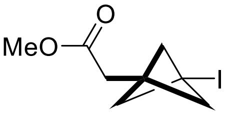

C	0.262984000	1.674192000	-0.666596000	C	-2.433309000	1.364058000	-0.309087000
C	0.728906000	0.328282000	-0.093297000	H	-2.562868000	2.266049000	0.289047000
H	0.523660000	2.558646000	-0.087793000	H	-2.692450000	1.567066000	-1.347621000
H	0.392294000	1.812565000	-1.738823000	C	-3.329199000	0.292666000	0.255779000
C	-0.392711000	-0.368610000	-0.875975000	O	-3.532310000	0.122049000	1.426154000
H	-0.726960000	-1.328867000	-0.486004000	O	-3.831420000	-0.496440000	-0.702992000
H	-0.304933000	-0.357242000	-1.961009000	C	-4.627339000	-1.586058000	-0.237894000
C	-0.239675000	0.518181000	1.082177000	H	-4.955247000	-2.116402000	-1.126676000
H	-0.570093000	-0.384815000	1.591124000	H	-4.035299000	-2.238165000	0.403109000
H	-0.013948000	1.333653000	1.766832000	H	-5.481020000	-1.215543000	0.327722000
C	-0.997258000	0.896280000	-0.214502000	I	2.745327000	-0.332373000	0.055494000

```
Zero-point correction=                           0.179593 (Hartree/Particle)
 Thermal correction to Energy=                    0.190858
 Thermal correction to Enthalpy=                  0.191802
 Thermal correction to Gibbs Free Energy=         0.139223
 Sum of electronic and zero-point Energies=           -759.295925
 Sum of electronic and thermal Energies=              -759.284659
 Sum of electronic and thermal Enthalpies=            -759.283715
 Sum of electronic and thermal Free Energies=         -759.336294

E[(RO)M06-2x/def2TZVP] = -759.475517295
```

iii) (RO)ωB97xD / def2tzvp

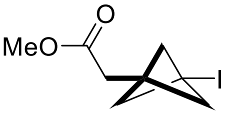

C	0.258705000	1.639596000	-0.712429000	C	-2.434492000	1.334626000	-0.363660000
C	0.748749000	0.314215000	-0.095604000	H	-2.561251000	2.269033000	0.183027000
H	0.508578000	2.549130000	-0.166721000	H	-2.677949000	1.494560000	-1.414194000
H	0.390965000	1.752280000	-1.788228000	C	-3.365657000	0.312702000	0.237637000
C	-0.377710000	-0.410480000	-0.857794000	O	-3.651758000	0.250089000	1.402049000
H	-0.706051000	-1.363060000	-0.443456000	O	-3.802879000	-0.557306000	-0.680797000
H	-0.289713000	-0.439743000	-1.943413000	C	-4.634212000	-1.608077000	-0.192734000
C	-0.238228000	0.537855000	1.067630000	H	-4.896921000	-2.205580000	-1.061469000
H	-0.561995000	-0.349155000	1.609768000	H	-4.096294000	-2.212535000	0.537564000
H	-0.023990000	1.373166000	1.733147000	H	-5.529922000	-1.201126000	0.275664000
C	-1.003550000	0.869708000	-0.241433000	I	2.761510000	-0.323587000	0.069031000

```
Zero-point correction=                           0.179656 (Hartree/Particle)
 Thermal correction to Energy=                    0.190925
 Thermal correction to Enthalpy=                  0.191869
 Thermal correction to Gibbs Free Energy=         0.139120
 Sum of electronic and zero-point Energies=           -759.500345
 Sum of electronic and thermal Energies=              -759.489077
 Sum of electronic and thermal Enthalpies=            -759.488132
 Sum of electronic and thermal Free Energies=         -759.540882

E[(RO)wB97xD/def2TZVP] = -759.680001681
```

[bromoacetate]

i) (RO)B3LYP / def2tzvp

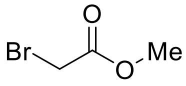

C	-0.239416000	0.764828000	0.810731000
H	-0.525659000	1.808834000	0.773000000
H	-0.232650000	0.381608000	1.825712000
C	1.091832000	0.574950000	0.125093000
O	1.586366000	1.378992000	-0.620791000
O	1.653878000	-0.593484000	0.466144000
C	2.910011000	-0.895356000	-0.166388000
H	3.201690000	-1.868737000	0.217048000
H	2.788982000	-0.928532000	-1.248347000
H	3.654007000	-0.141770000	0.088503000
Br	-1.639511000	-0.234343000	-0.143867000

```
Zero-point correction=                           0.080665 (Hartree/Particle)
 Thermal correction to Energy=                    0.087709
 Thermal correction to Enthalpy=                  0.088653
 Thermal correction to Gibbs Free Energy=         0.047291
 Sum of electronic and zero-point Energies=          -2841.996891
 Sum of electronic and thermal Energies=             -2841.989847
 Sum of electronic and thermal Enthalpies=           -2841.988903
 Sum of electronic and thermal Free Energies=        -2842.030265

E[(RO)B3LYP/def2TZVP] = -2842.07755604
```

ii) (RO)M06-2x / def2tzvp

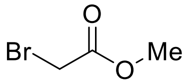

C	-0.255920000	0.790743000	0.794292000
H	-0.535782000	1.834867000	0.723042000
H	-0.247921000	0.438914000	1.820555000
C	1.077248000	0.576948000	0.124515000
O	1.583830000	1.359879000	-0.627313000
O	1.619139000	-0.588849000	0.477403000
C	2.860269000	-0.899337000	-0.156593000
H	3.157032000	-1.868804000	0.230792000
H	2.727673000	-0.939262000	-1.236786000
H	3.605544000	-0.142208000	0.082996000
Br	-1.611997000	-0.237196000	-0.142703000

```
 Zero-point correction=                           0.081842 (Hartree/Particle)
 Thermal correction to Energy=                    0.088768
 Thermal correction to Enthalpy=                  0.089713
 Thermal correction to Gibbs Free Energy=         0.048615
 Sum of electronic and zero-point Energies=          -2841.890676
 Sum of electronic and thermal Energies=             -2841.883750
 Sum of electronic and thermal Enthalpies=           -2841.882806
 Sum of electronic and thermal Free Energies=        -2841.923903

E[(RO)M06-2x/def2TZVP] = -2841.97251788
```

iii) (RO)ωB97xD / def2tzvp

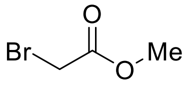

C	-0.250718000	0.747946000	0.813236000
H	-0.529636000	1.795237000	0.790321000
H	-0.235596000	0.358148000	1.826312000
C	1.081917000	0.568107000	0.128654000
O	1.565761000	1.371398000	-0.618324000
O	1.649314000	-0.588637000	0.467019000
C	2.893003000	-0.880140000	-0.169274000
H	3.212481000	-1.837144000	0.233292000
H	2.758625000	-0.945539000	-1.248688000
H	3.627409000	-0.106690000	0.053707000
Br	-1.625688000	-0.232616000	-0.145148000

```
Zero-point correction=                           0.081656 (Hartree/Particle)
 Thermal correction to Energy=                    0.088626
 Thermal correction to Enthalpy=                  0.089570
 Thermal correction to Gibbs Free Energy=         0.048353
 Sum of electronic and zero-point Energies=          -2841.938664
 Sum of electronic and thermal Energies=             -2841.931694
 Sum of electronic and thermal Enthalpies=           -2841.930750
 Sum of electronic and thermal Free Energies=        -2841.971967

E[(RO)wB97xD/def2TZVP] = -2842.02031976
```

[BBr‡]

i) (RO)B3LYP / def2tzvp

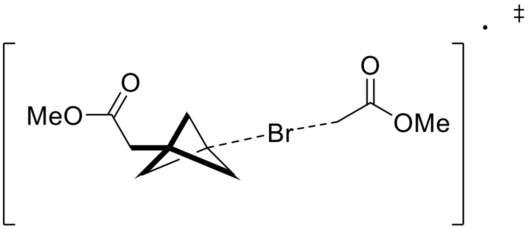

C	1.155437000	-1.321506000	1.262016000	C	-4.634513000	0.076627000	0.067035000
C	1.637147000	-1.011150000	-0.850287000	O	-4.708076000	1.351876000	-0.374985000
C	1.283267000	0.709677000	0.456264000	O	-5.110627000	-0.293783000	1.113905000
H	0.909108000	-0.978794000	2.264697000	C	-5.362009000	2.277293000	0.502914000
H	1.202157000	-2.405394000	1.180511000	H	-5.324452000	3.238170000	-0.003517000
C	2.253664000	-0.515179000	0.497726000	H	-6.395054000	1.977228000	0.677825000
H	1.828523000	-0.392071000	-1.721089000	H	-4.842421000	2.328838000	1.459610000
H	1.710718000	-2.078038000	-1.051659000	H	3.836800000	-0.155308000	1.911100000
H	1.453483000	1.428957000	-0.338637000	H	4.122986000	-1.518824000	0.858775000
H	1.042474000	1.168613000	1.413017000	C	4.590421000	0.363518000	-0.007289000
C	3.720877000	-0.502054000	0.880722000	O	5.865545000	0.341068000	0.424655000
C	0.515283000	-0.565798000	0.094052000	C	6.803912000	1.122940000	-0.334726000
Br	-1.806011000	-0.641650000	-0.404318000	H	7.763676000	0.980957000	0.153944000
C	-3.893902000	-0.785418000	-0.882903000	H	6.843464000	0.774821000	-1.366138000
H	-4.104940000	-1.836034000	-0.737587000	H	6.520165000	2.174609000	-0.323859000
H	-3.925707000	-0.457018000	-1.913694000	O	4.222143000	0.990006000	-0.966739000

```
Zero-point correction=                           0.254820 (Hartree/Particle)
 Thermal correction to Energy=                    0.273290
 Thermal correction to Enthalpy=                  0.274234
 Thermal correction to Gibbs Free Energy=         0.201810
 Sum of electronic and zero-point Energies=          -3303.773305
 Sum of electronic and thermal Energies=             -3303.754835
 Sum of electronic and thermal Enthalpies=           -3303.753891
 Sum of electronic and thermal Free Energies=        -3303.826314

E[(RO)B3LYP/def2TZVP] = -3304.02812337
```

ii) (RO)M06-2x / def2tzvp

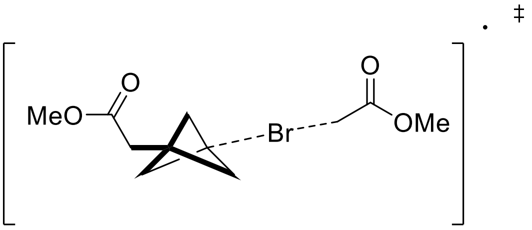

C	1.148735000	-1.615564000	1.055418000	C	-4.491952000	0.180305000	0.104597000
C	1.574326000	-0.903575000	-0.965497000	O	-4.484713000	1.420263000	-0.411573000
C	1.203019000	0.528256000	0.641333000	O	-4.946027000	-0.081685000	1.186972000
H	0.914923000	-1.465497000	2.106519000	C	-5.018272000	2.429455000	0.439126000
H	1.224564000	-2.661178000	0.766834000	H	-4.937864000	3.360603000	-0.114069000
C	2.193341000	-0.650402000	0.436096000	H	-6.058889000	2.213935000	0.679378000
H	1.731020000	-0.122139000	-1.701977000	H	-4.447535000	2.482533000	1.365704000
H	1.672403000	-1.913063000	-1.357803000	H	3.809083000	-0.468656000	1.845523000
H	1.341933000	1.382378000	-0.013839000	H	4.110975000	-1.621209000	0.561544000
H	0.970514000	0.786555000	1.671870000	C	4.441630000	0.401934000	0.018008000
C	3.666234000	-0.646730000	0.777131000	O	5.734648000	0.379881000	0.362687000
C	0.470289000	-0.677871000	0.062515000	C	6.561745000	1.334329000	-0.302865000
Br	-1.780739000	-0.711039000	-0.386870000	H	7.558493000	1.199366000	0.105602000
C	-3.849240000	-0.795112000	-0.806864000	H	6.558190000	1.152805000	-1.376748000
H	-4.126037000	-1.815053000	-0.575521000	H	6.201291000	2.344331000	-0.113219000
H	-3.910422000	-0.533526000	-1.855616000	O	3.985356000	1.163296000	-0.789053000

```
Zero-point correction=                           0.258512 (Hartree/Particle)
 Thermal correction to Energy=                    0.276525
 Thermal correction to Enthalpy=                  0.277469
 Thermal correction to Gibbs Free Energy=         0.206543
 Sum of electronic and zero-point Energies=          -3303.469478
```

```
 Sum of electronic and thermal Energies=              -3303.451466
 Sum of electronic and thermal Enthalpies=            -3303.450522
 Sum of electronic and thermal Free Energies=         -3303.521448

E[(RO)M06-2x/def2TZVP] = -3303.72799004
```

iii) (RO)ωB97xD / def2tzvp

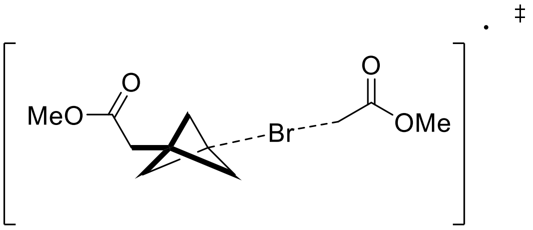

C	1.303849000	-2.047574000	0.277721000
C	1.409547000	-0.422028000	-1.163850000
C	1.240462000	0.016708000	0.959837000
H	1.221615000	-2.454003000	1.284167000
H	1.362945000	-2.818530000	-0.488533000
C	2.235510000	-0.814669000	0.096940000
H	1.437035000	0.627445000	-1.442674000
H	1.474408000	-1.098973000	-2.014075000
H	1.256682000	1.091381000	0.803230000
H	1.152717000	-0.266290000	2.007332000
C	3.736565000	-0.845245000	0.222469000
C	0.445691000	-0.821764000	-0.043451000
Br	-1.796151000	-0.836058000	-0.194512000
C	-3.920484000	-0.918220000	-0.303254000
H	-4.176723000	-1.780278000	0.299020000
H	-4.114018000	-1.037096000	-1.362361000
C	-4.430319000	0.344491000	0.279146000
O	-4.494664000	1.321348000	-0.638666000
O	-4.734691000	0.496773000	1.433765000
C	-4.906226000	2.593263000	-0.151961000
H	-4.912930000	3.254320000	-1.014732000
H	-5.900487000	2.533519000	0.291107000
H	-4.206754000	2.958332000	0.600597000
H	4.025574000	-1.220574000	1.207058000
H	4.161121000	-1.540408000	-0.506310000
C	4.389727000	0.501019000	0.021647000
O	5.714215000	0.408357000	0.183554000
C	6.445988000	1.622159000	0.024916000
H	7.488852000	1.363034000	0.185301000
H	6.303491000	2.028822000	-0.976098000
H	6.121328000	2.361930000	0.756367000
O	3.823714000	1.524842000	-0.246459000

```
Zero-point correction=                             0.258409 (Hartree/Particle)
 Thermal correction to Energy=                      0.276352
 Thermal correction to Enthalpy=                    0.277296
 Thermal correction to Gibbs Free Energy=           0.207130
 Sum of electronic and zero-point Energies=            -3303.564163
 Sum of electronic and thermal Energies=               -3303.546220
 Sum of electronic and thermal Enthalpies=             -3303.545276
 Sum of electronic and thermal Free Energies=          -3303.615442

E[(RO)wB97xD/def2TZVP] = -3303.82257154
```

[BBr]

i) (RO)B3LYP / def2tzvp

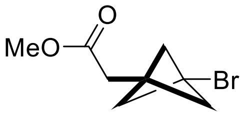

C	0.848733000	1.553473000	-0.577444000
C	1.309689000	0.178588000	-0.056658000
H	1.121575000	2.417268000	0.027658000
H	0.973254000	1.736144000	-1.644261000
C	0.170850000	-0.479481000	-0.859512000
H	-0.166354000	-1.453874000	-0.508917000
H	0.251308000	-0.432629000	-1.944854000
C	0.345441000	0.334867000	1.136005000
H	0.010204000	-0.579792000	1.619598000
H	0.583225000	1.120175000	1.852132000
C	-0.429538000	0.771896000	-0.144666000
C	-1.852116000	1.284290000	-0.232840000
H	-1.948623000	2.178488000	0.387218000
H	-2.088689000	1.548339000	-1.263620000
C	-2.848138000	0.267510000	0.281713000
O	-2.954748000	-0.064507000	1.434699000
O	-3.598586000	-0.243451000	-0.713331000
C	-4.563459000	-1.236799000	-0.325600000
H	-5.067636000	-1.527387000	-1.242971000
H	-4.066686000	-2.093820000	0.127583000
H	-5.273448000	-0.819460000	0.387364000
Br	3.148850000	-0.447881000	0.031604000

```
Zero-point correction=                           0.177700 (Hartree/Particle)
 Thermal correction to Energy=                    0.189007
 Thermal correction to Enthalpy=                  0.189952
 Thermal correction to Gibbs Free Energy=         0.137344
 Sum of electronic and zero-point Energies=          -3036.041813
 Sum of electronic and thermal Energies=             -3036.030505
 Sum of electronic and thermal Enthalpies=           -3036.029561
 Sum of electronic and thermal Free Energies=        -3036.082169

E[(RO)B3LYP/def2TZVP] = -3036.21951290
```

ii) (RO)M06-2x / def2tzvp

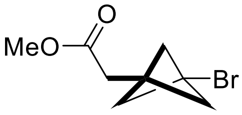

C	-0.255920000	0.790743000	0.794292000
H	-0.535782000	1.834867000	0.723042000
H	-0.247921000	0.438914000	1.820555000
C	1.077248000	0.576948000	0.124515000
O	1.583830000	1.359879000	-0.627313000
O	1.619139000	-0.588849000	0.477403000
C	2.860269000	-0.899337000	-0.156593000
H	3.157032000	-1.868804000	0.230792000
H	2.727673000	-0.939262000	-1.236786000
H	3.605544000	-0.142208000	0.082996000
Br	-1.611997000	-0.237196000	-0.142703000

```
Zero-point correction=                           0.081842 (Hartree/Particle)
 Thermal correction to Energy=                    0.088768
 Thermal correction to Enthalpy=                  0.089713
 Thermal correction to Gibbs Free Energy=         0.048615
 Sum of electronic and zero-point Energies=          -2841.890676
 Sum of electronic and thermal Energies=             -2841.883750
 Sum of electronic and thermal Enthalpies=           -2841.882806
 Sum of electronic and thermal Free Energies=        -2841.923903

E[(RO)M06-2x/def2TZVP] = -2841.97251788
```

iii) (RO)ωB97xD / def2tzvp

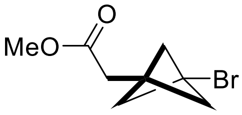

C	-0.250718000	0.747946000	0.813236000
H	-0.529636000	1.795237000	0.790321000
H	-0.235596000	0.358148000	1.826312000
C	1.081917000	0.568107000	0.128654000
O	1.565761000	1.371398000	-0.618324000
O	1.649314000	-0.588637000	0.467019000
C	2.893003000	-0.880140000	-0.169274000
H	3.212481000	-1.837144000	0.233292000
H	2.758625000	-0.945539000	-1.248688000
H	3.627409000	-0.106690000	0.053707000
Br	-1.625688000	-0.232616000	-0.145148000

```
Zero-point correction=                           0.081656 (Hartree/Particle)
 Thermal correction to Energy=                    0.088626
 Thermal correction to Enthalpy=                  0.089570
 Thermal correction to Gibbs Free Energy=         0.048353
 Sum of electronic and zero-point Energies=          -2841.938664
 Sum of electronic and thermal Energies=             -2841.931694
 Sum of electronic and thermal Enthalpies=           -2841.930750
 Sum of electronic and thermal Free Energies=        -2841.971967

E[(RO)wB97xD/def2TZVP] = -2842.02031976
```

11. X-ray Crystallography

Low temperature[40] single crystal X-ray diffraction data were collected with a (Rigaku) Oxford Diffraction SuperNova A diffractometer at 150 or 200 K. Data were reduced using the instrument manufacturer software CrysAlisPro. All structures were solved *ab initio* using SuperFlip[41] and the structures were refined using CRYSTALS.[42,43]

Structure **5m** contained solvent accessible voids comprising of weak, diffuse electron density. The discrete Fourier transforms of the void regions were treated as contributions to the A and B parts of the calculated structure factors using PLATON/SQUEEZE[44,45] integrated within the CRYSTALS software. This enabled a comparison of models, one of which contained the disordered solvent, the other without.

Further details about the refinements, including disorder modelling and restraints, are documented in the CIF. The crystallographic data have been deposited with the CCDC as entries CCDC 1825056–1825060.

Table S3 Summary of X-ray crystallographic data

Compound Number	**5m**	**5r**	**5s**	**6b**	**17**
Moiety Formula	$C_8H_{12}NOI$, 0.5 ($CHCl_3$)	$C_{12}H_{12}NO_2I$	$C_{13}H_{12}F_3I$	$C_8H_{11}NO_2Br$	$C_{21}H_{30}N_2O$
CCDC	1825056	1825057	1825058	1825059	1825060
Space Group	C 2/c	P 2_1/c	P -1	P -1	P 2_1/n
a [Å]	25.6568(11)	12.8692(2)	5.6020(3)	7.0213(3)	14.7270(3)
b [Å]	9.9841(3)	8.45340(10)	8.9978(5)	11.7894(5)	5.77560(10)
c [Å]	9.9880(4)	11.4743(2)	13.3874(9)	12.2156(5)	21.9219(5)
α [°]	90	90	95.953(5)	72.797(4)	90
β [°]	95.639(4)	99.0055(15)	98.695(5)	86.293(4)	92.4149(18)
γ [°]	90	90	98.785(5)	89.879(3)	90
V [$Å^3$]	2546.14(17)	1232.88(3)	653.64(7)	963.76(7)	1862.96(7)
Z	8	4	2	4 (Z' = 2)	4
T [K]	150	150	200	150	150
Total Reflections	12571	26750	5134	16923	19286
R_{int}	0.0490	0.0654	0.0320	0.0503	0.0229
Reflections, Restraints, Parameters (I>-3.0/ σ (I))	2659, 347, 173	2575, 0, 145	2661, 555, 211	3998, 948, 311	3885, 462, 263
Min. and Max. Residual Density, [$eÅ^{-3}$]	-1.44, 1.34	-0.99, 0.86	-0.94, 0.76	-0.68, 0.52	-0.22, 0.22
R_1 (I>2σ(I)) wR_2	0.0399 0.1071	0.0274 0.0756	0.0358 0.0947	0.0328 0.0824	0.0377 0.0948

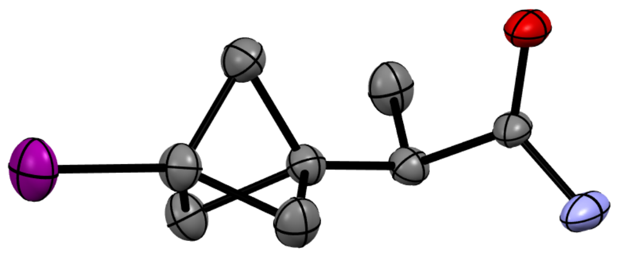

Figure S2: Solid state structure of **5m**. Displacement ellipsoid plots are drawn at 50% probability. Hydrogen atoms and disordered solvent molecules are omitted for clarity.

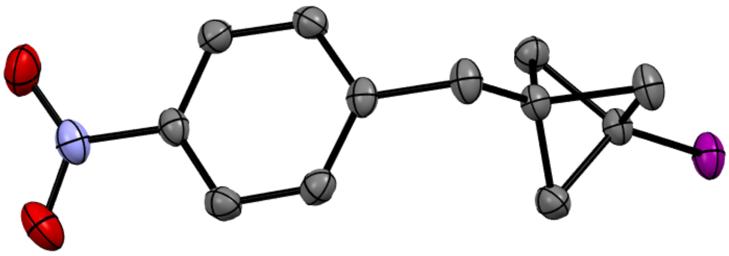

Figure S3: Solid state structure of **5r**. Displacement ellipsoid plots are drawn at 50% probability. Hydrogen atoms are omitted for clarity.

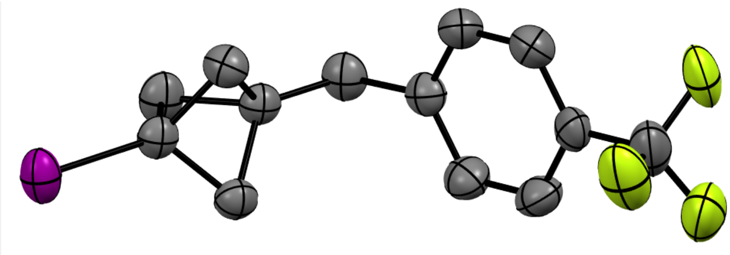

Figure S4: Solid state structure of **5s**. Displacement ellipsoid plots are drawn at 50% probability. Hydrogen atoms and disordered components are omitted for clarity.

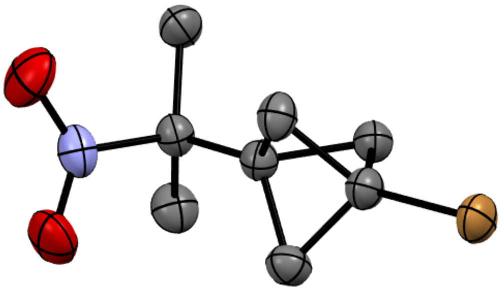

Figure S5: Solid state structure of **6b**. Displacement ellipsoid plots are drawn at 50% probability. Hydrogen atoms and disordered components are omitted for clarity.

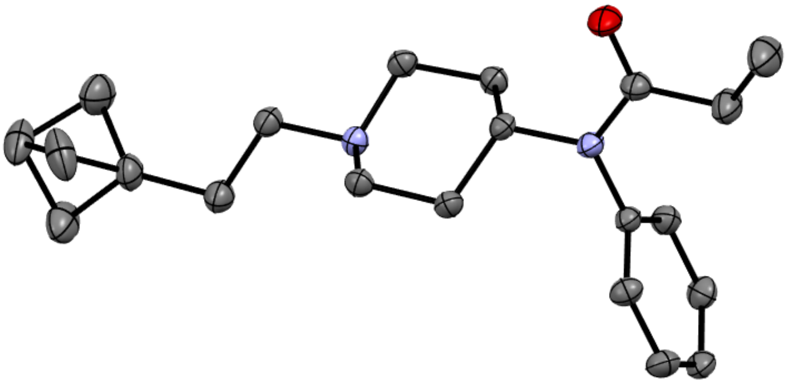

Figure S6: Solid state structure of **17**. Displacement ellipsoid plots are drawn at 50% probability. Hydrogen atoms and disordered components are omitted for clarity.

12. References

(1) Zhou, X. M.; Wang, Z. Q.; Chang, J. Y.; Chen, H. X.; Cheng, Y. C.; Lee, K. H. *J. Med. Chem.* **1991**, *34*, 3346.

(2) Yin, G.; Gao, M.; She, N.; Hu, S.; Wu, A.; Pan, Y. *Synthesis* **2007**, 3113.

(3) Gianatassio, R.; Lopchuk, J. M.; Wang, J.; Pan, C.-M.; Malins, L. R.; Prieto, L.; Brandt, T. A.; Collins, M. R.; Gallego, G. M.; Sach, N. W.; Spangler, J. E.; Zhu, H.; Zhu, J.; Baran, P. S. *Science* **2016**, *351*, 241.

(4) Messner, M.; Kozhushkov, S. I.; de Meijere, A. *Eur. J. Org. Chem.* **2000**, 1137.

(5) Messner, M.; Kozhushkov, S. I.; de Meijere, A. *Eur. J. Org. Chem.* **2000**, 1137.

(6) Koseki, Y.; Yamada, H.; Usuki, T. *Tetrahedron: Asymm.* **2011**, *22*, 580.

(7) Lei, L.; Tanishima, M.; Goto, A.; Kaji, H.; Yamaguchi, Y.; Komatsu, H.; Jitsukawa, T.; Miyamoto, M. *Macromolecules* **2014**, *47*, 6610.

(8) Liu, Y.; Xu, Y.; Jung, S. H.; Chae, J. *Synlett* **2012**, 2692.

(9) Hunter, C.; Jackson, R. F. W.; Rami, H. K. *J. Chem. Soc., Perkin Trans. 1* **2000**, 219.

(10) Prebil, R.; Stavber, S. *Tetrahedron Lett.* **2014**, *55*, 5643.

(11) Aydin, O.; Kilic, H.; Bayindir, S.; Erdogan, E.; Saracoglu, N. *J. Heterocyc. Chem.* **2015**, *52*, 1540.

(12) Jereb, M.; Stavber, S.; Zupan, M. *Synthesis* **2003**, 853.

(13) Pospíšil, J.; Robiette, R.; Sato, H.; Debrus, K. *Org. Biomol. Chem.* **2012**, *10*, 1225.

(14) Danner, P.; Morkunas, M.; Maier, M. E. *Org. Lett.* **2013**, *15*, 2474.

(15) Ikubo, M.; Inoue, A.; Nakamura, S.; Jung, S.; Sayama, M.; Otani, Y.; Uwamizu, A.; Suzuki, K.; Kishi, T.; Shuto, A.; Ishiguro, J.; Okudaira, M.; Kano, K.; Makide, K.; Aoki, J.; Ohwada, T. *J. Med. Chem.* **2015**, *58*, 4204.

(16) Ding, R.; He, Y.; Wang, X.; Xu, J.; Chen, Y.; Feng, M.; Qi, C. *Molecules* **2011***, 16*, 5665.

(17) Sakai, N.; Matsushita, Y.; Konakahara, T.; Ogiwara, Y.; Hirano, K. *Eur. J. Org. Chem.* **2015**, 1591.

(18) Fuson, R. C. *J. Am. Chem. Soc.* **1926**, *48*, 830.

(19) Guisán Ceinos, M.; Soler Yanes, R.; Collado Sanz, D.; Phapale, V. B.; Buñuel, E.; Cárdenas, D. J. *Chem. Eur. J.* **2013**, *19*, 8405.

(20) Wolfe, S.; Ro, S.; Kim, C.-K.; Shi, Z. *Can. J. Chem.* **2001**, *79*, 1238.

(21) Curran, D. P.; Bosch, E.; Kaplan, J.; Newcomb, M. *J. Org. Chem.* **1989**, *54*, 1826.

(22) Izumisawa, Y.; Togo, H. *Green Sust.Chem.* **2011**, *1*, 54.

(23) Paul, B.; Bhuyan, B.; Purkayastha, D. D.; Dhar, S. S.; Patel, B. K. *Tetrahedron Lett.* **2015**, *56*, 5646.

(24) Suryakiran, N.; Reddy, T. S.; Ashalatha, K.; Lakshman, M.; Venkateswarlu, Y. *Tetrahedron Lett.* **2006**, *47*, 3853.

(25) Suryakiran, N.; Prabhakar, P.; Srikanth Reddy, T.; Chinni Mahesh, K.; Rajesh, K.; Venkateswarlu, Y. *Tetrahedron Lett.* **2007**, *48*, 877.

(26) Lu, Q.; Zhang, J.; Zhao, G.; Qi, Y.; Wang, H.; Lei, A. *J. Am. Chem. Soc.* **2013**, *135*, 11481.

(27) Haugland, M. M.; El-Sagheer, A. H.; Porter, R. J.; Peña, J.; Brown, T.; Anderson, E. A.; Lovett, J. E. *J. Am. Chem. Soc.* **2016**, *138*, 9069.

(28) Seamon, K. J.; Hansen, E. C.; Kadina, A. P.; Kashemirov, B. A.; McKenna, C. E.; Bumpus, N. N.; Stivers, J. T. *J. Am. Chem. Soc.* **2014**, *136*, 9822.

(29) Sun, Q.; Sun, J.; Gong, S.-S.; Wang, C.-J.; Pu, S.-Z.; Feng, F.-D. *RSC Adv.* **2014**, *4*, 36036.

(30) Wegert, A.; Kühnert, S.; Koenigs, R. M.; Nolte, B.; Linz, K.; Harlfinger, S.; Kögel, B.-Y.; Ratcliffe, P.; Theil, F.; Gröger, O.; Braun, B. World Intellectual Property Organization January 21, 2016, pp C07D209/96AI; A61K31/403AI; A61P29/00AI.

(31) Pu, Y. J.; Vaid, R. K.; Boini, S. K.; Towsley, R. W.; Doecke, C. W.; Mitchell, D. *Org. Proc. Res. Dev.* **2009**, *13*, 310.

(32) Brine, G. A.; Boldt, K. G.; Huang, P. T.; Sawyer, D. K.; Carroll, F. I. *J. Heterocyc. Chem.* **1989**, *26*, 677.

(33) Gupta, P. K.; Yadav, S. K.; Bhutia, Y. D.; Singh, P.; Rao, P.; Gujar, N. L.; Ganesan, K.; Bhattacharya, R. *Med. Chem. Res.* **2012**, *22*, 3888.

(34) Sudo, M.; Iwata, Y.; Arano, Y.; Jinno, M.; Ohmi, M.; Noguchi, H. World Intellectual Property Organization September 2, 2010, pp C07D401/14AI; A61K31/454AI; A61K31/497AI; A61P1/04AI; A61P1/06AI; A61P43/00AI; C07D401/04AI.

(35) Gaussian 09, Revision D.01, Frisch, M. J.; Trucks, G. W.; Schlegel, H. B.; Scuseria, G. E.; Robb, M. A.; Cheeseman, J. R.; Scalmani, G.; Barone, V.; Mennucci, B.; Petersson, G. A.; Nakatsuji, H.; Caricato, M.; Li, X.; Hratchian, H. P.; Izmaylov, A. F.; Bloino, J.; Zheng, G.; Sonnenberg, J. L.; Hada, M.; Ehara, M.; Toyota, K.; Fukuda, R.; Hasegawa, J.; Ishida, M.; Nakajima, T.; Honda, Y.; Kitao, O.; Nakai, H.; Vreven, T.; Montgomery, J. A., Jr.; Peralta, J. E.; Ogliaro, F.; Bearpark, M.; Heyd, J. J.; Brothers, E.; Kudin, K. N.; Staroverov, V. N.; Kobayashi, R.; Normand, J.; Raghavachari, K.; Rendell, A.; Burant, J. C.; Iyengar, S. S.; Tomasi, J.; Cossi, M.; Rega, N.; Millam, J. M.; Klene, M.; Knox, J. E.; Cross, J. B.; Bakken, V.; Adamo, C.; Jaramillo, J.; Gomperts, R.; Stratmann, R. E.; Yazyev, O.; Austin, A. J.; Cammi, R.; Pomelli, C.; Ochterski, J. W.; Martin, R. L.; Morokuma, K.; Zakrzewski, V. G.; Voth, G. A.; Salvador, P.; Dannenberg, J. J.; Dapprich, S.; Daniels, A. D.; Farkas, Ö.; Foresman, J. B.; Ortiz, J. V.; Cioslowski, J.; Fox, D. J. Gaussian, Inc., Wallingford CT, 2009.

(36) A. D. Becke, *Phys. Rev. A* **1988**, *38*, 3098; A. D. Becke, *J. Phys. Chem.* **1993**, *98*, 5648; C. Lee, W. Yang, R. G. Parr, *Phys. Rev. B* **1988**, *37*, 785.

(37) Y. Zhao, D. G. Truhlar, *Theor. Chem. Acc.* **2008**, *120*, 215.

(38) J.-D. Chai, M. Head-Gordon, *J. Chem. Phys.* **2009**, *131*, 174105.

(39) F. Weigend, R. Ahlrichs, *Phys. Chem. Chem. Phys.* **2005**, *7*, 3297.

(40) Cosier, J.; Glazer, A. M.; IUCr. *J. Appl. Crystallogr.* **1986**, *19*, 105.

(41) Palatinus, L.; Chapuis, G. *J. Appl. Crystallogr.* **2007**, *40*, 786.

(42) Betteridge, P. W.; Carruthers, J. R.; Cooper, R. I.; Prout, K.; Watkin, D. J. *J. Appl. Crystallogr.* **2003**, *36*, 1487.

(43) Cooper, R. I.; Thompson, A. L.; Watkin, D. J. *J. Appl. Crystallogr.* **2010**, *43*, 1100.

(44) Spek, A. L. *J. Appl. Crystallogr.* **2003**, *36*, 7.

(45) van der Sluis, P.; Spek, A. L.; IUCr. *Acta Cryst. Sect A Found. Crystallogr.* **1990**, *46*, 194.

12. Copies of NMR spectra

Ethyl 2-(3-iodobicyclo[1.1.1]pentan-1-yl)acetate, 5a

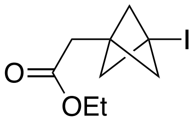

^{1}H NMR (400 MHz, $CDCl_3$)

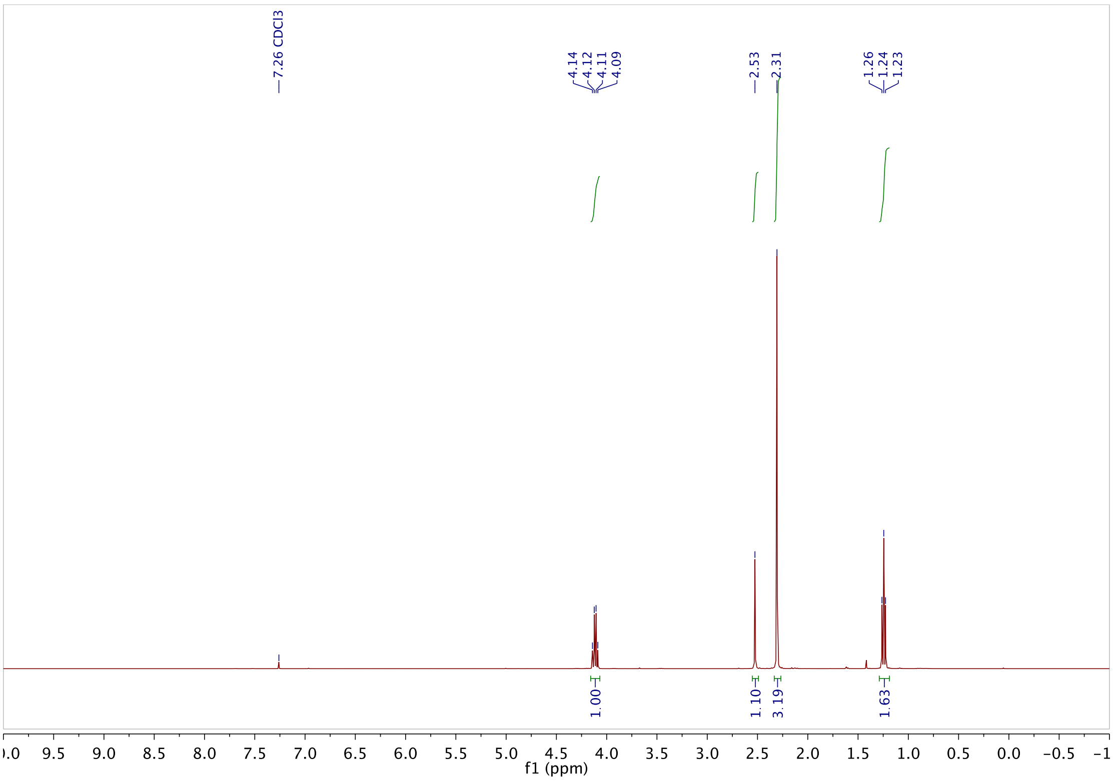

^{13}C NMR (101 MHz, $CDCl_3$)

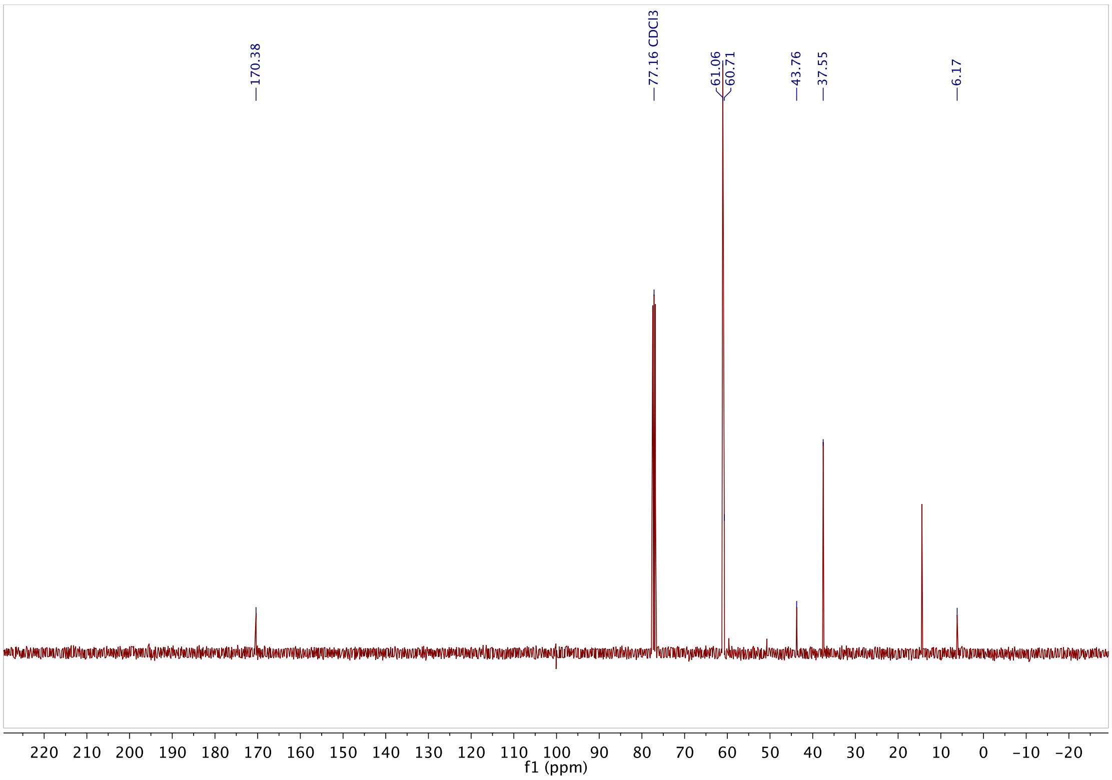

1-Bromo-3-(2-nitropropan-2-yl)bicyclo[1.1.1]pentane, 6b

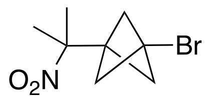

¹H NMR (400 MHz, $CDCl_3$)

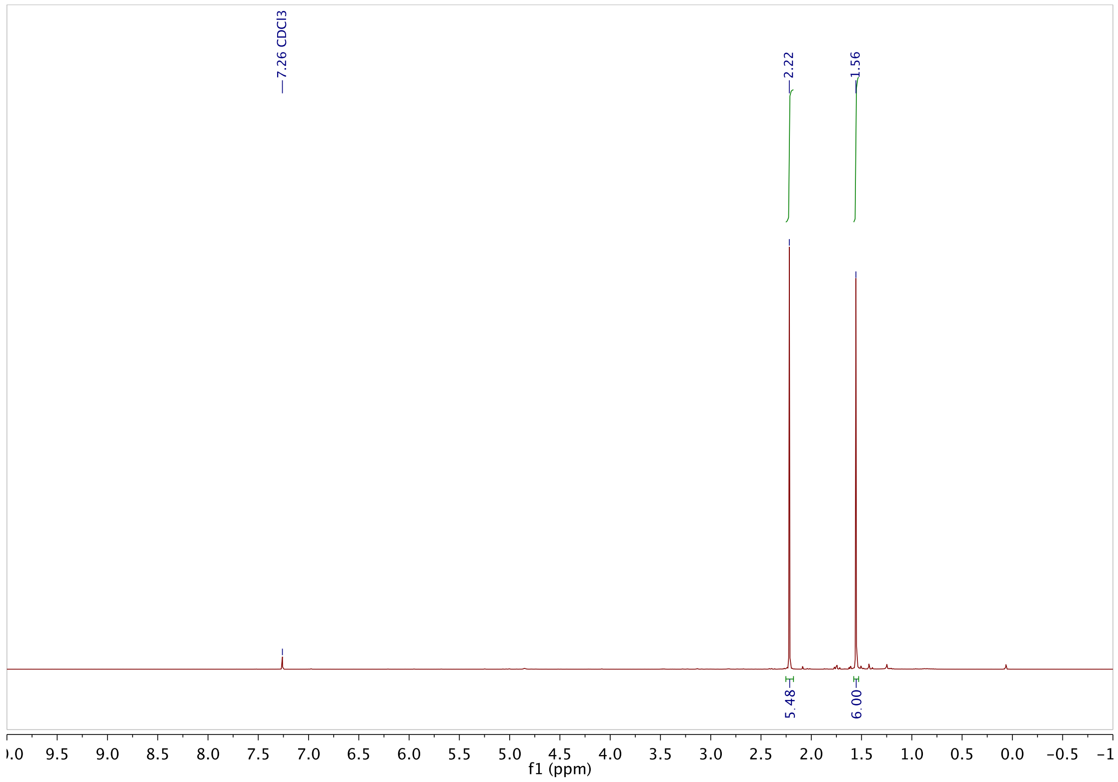

¹³C NMR (101 MHz, $CDCl_3$)

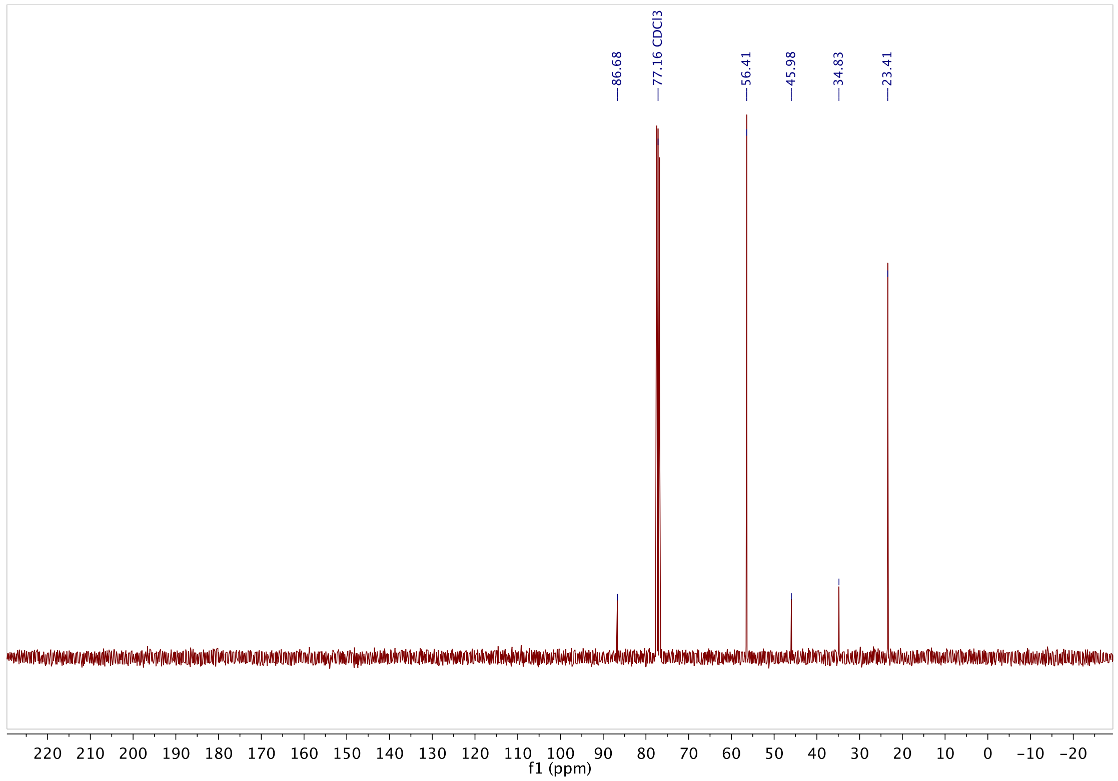

Ethyl 2,2-difluoro-2-(3-iodobicyclo[1.1.1]pentan-1-yl)acetate, 5b

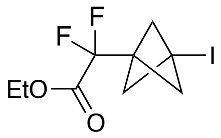

^{1}H NMR (400 MHz, $CDCl_3$)

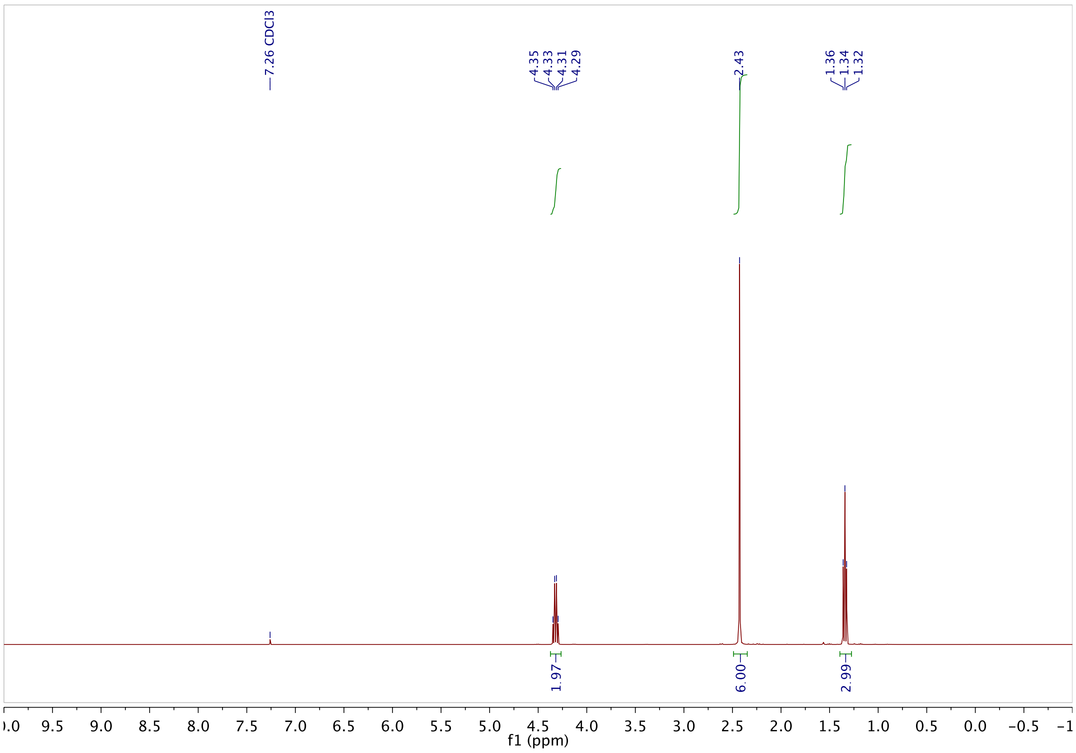

^{13}C NMR (101 MHz, $CDCl_3$)

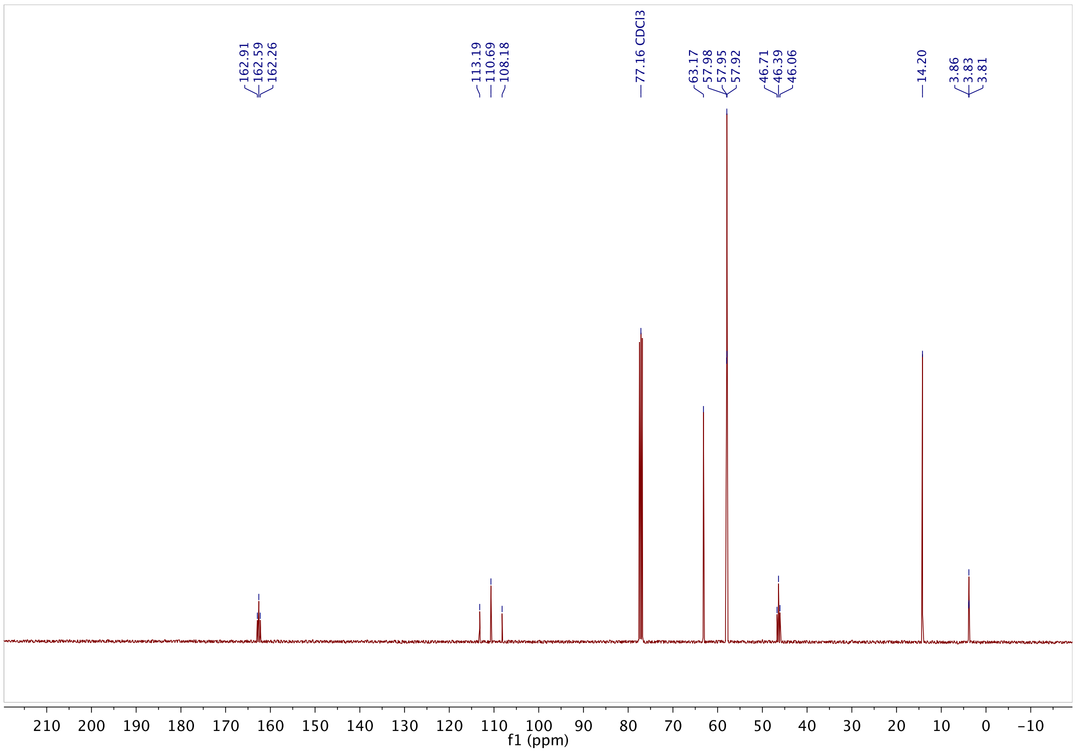

^{19}F NMR (377 MHz, $CDCl_3$)

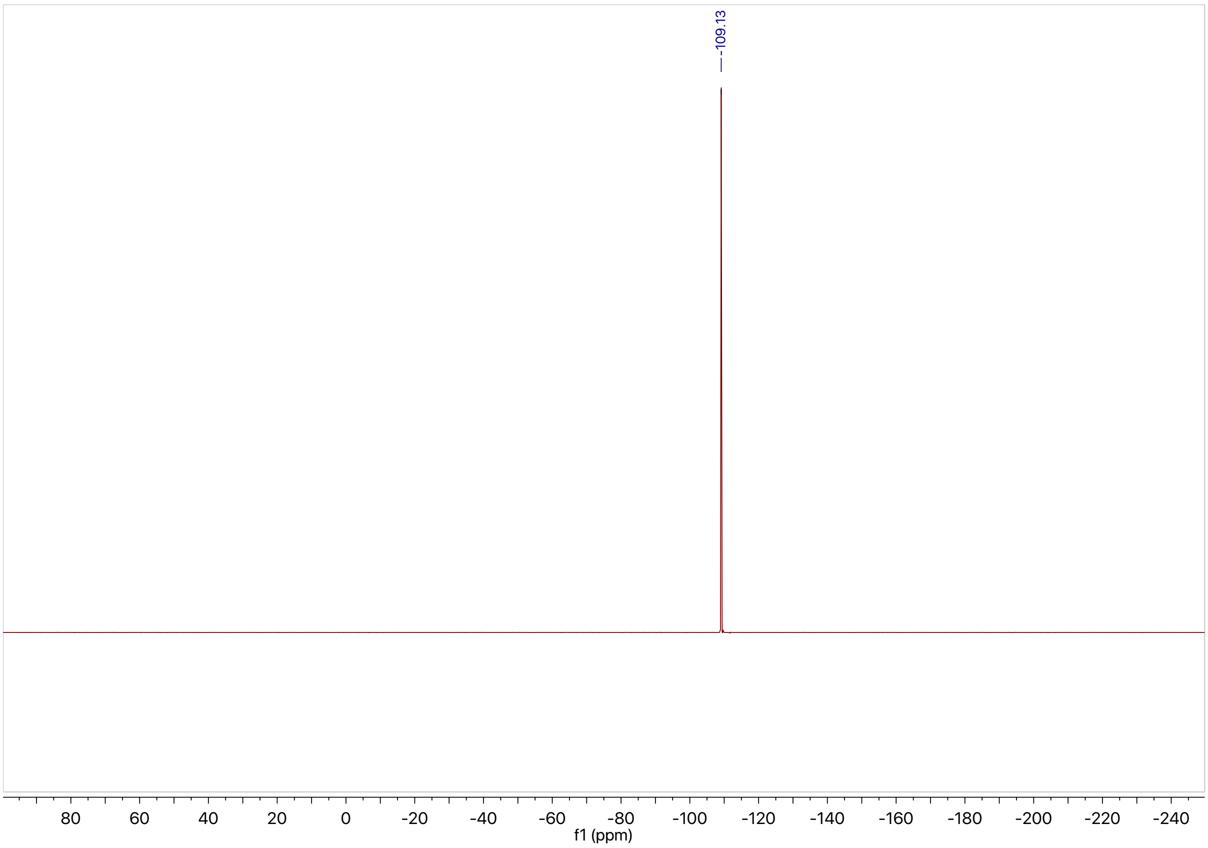

1-Iodo-3-phenethylbicyclo[1.1.1]pentane, 5c

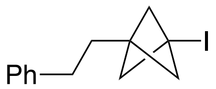

^{1}**H NMR** (400 MHz, C_6D_6)

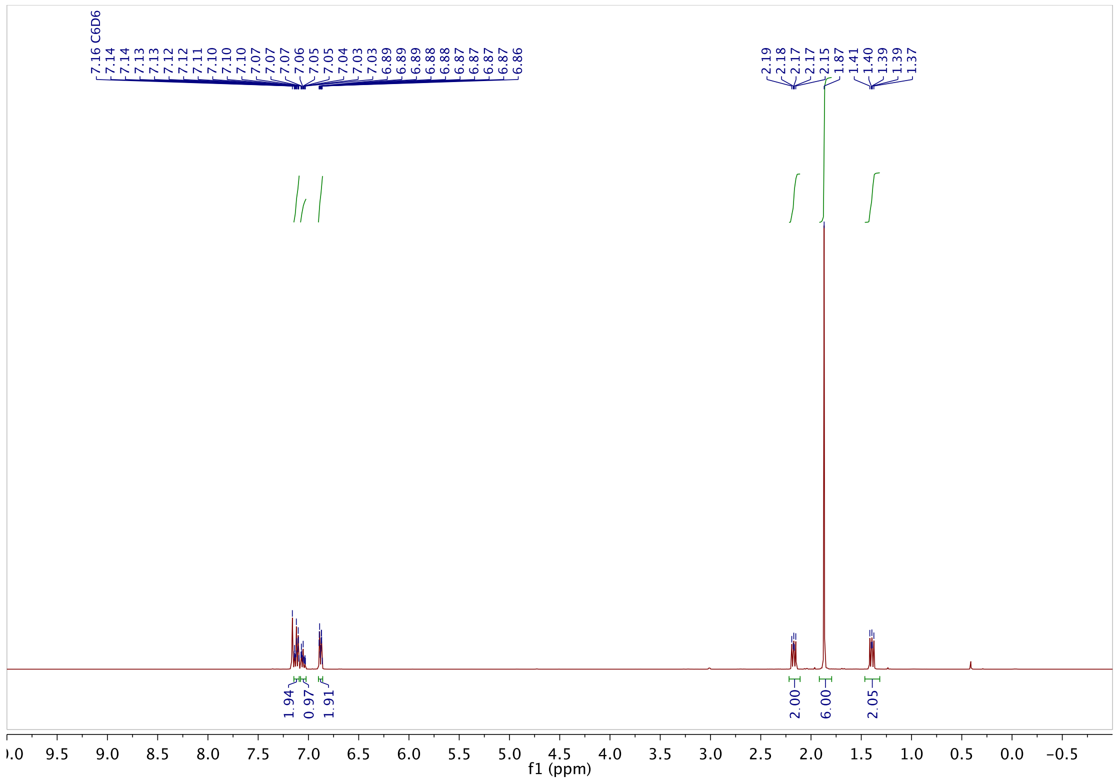

^{13}**C NMR** (101 MHz, C_6D_6)

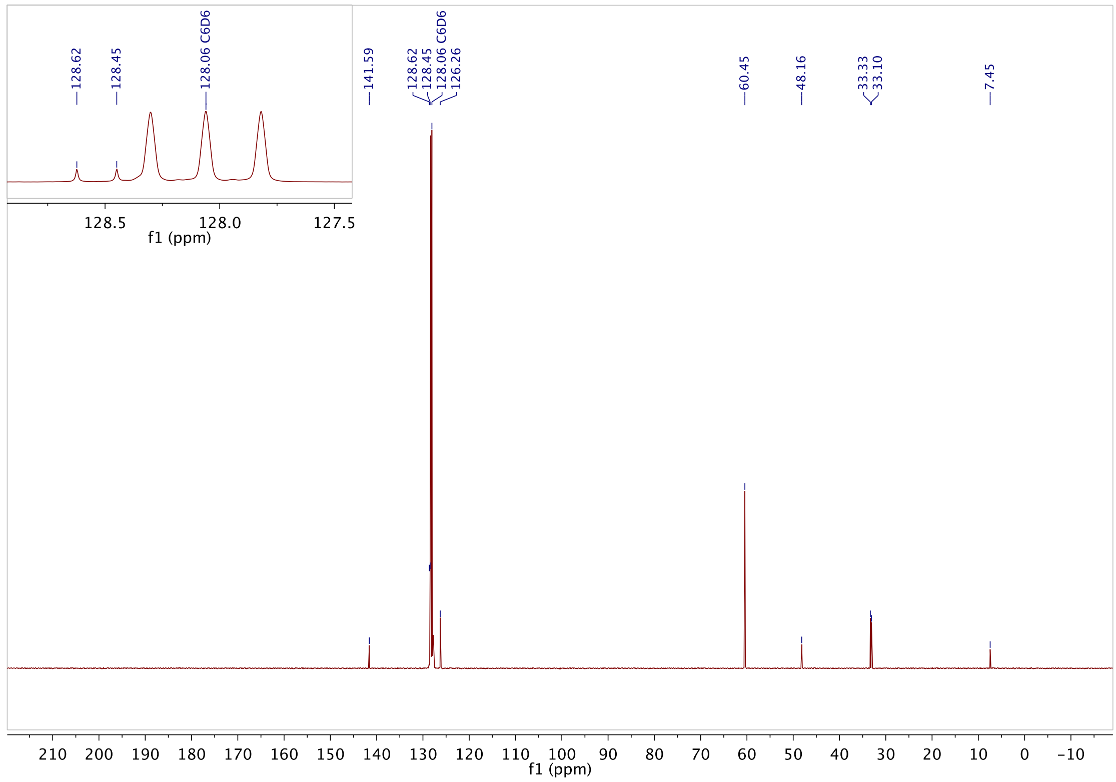

2-(3-Iodobicyclo[1.1.1]pentan-1-yl)ethan-1-ol, 5d

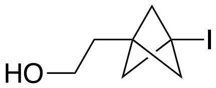

^{1}H NMR (400 MHz, $CDCl_3$)

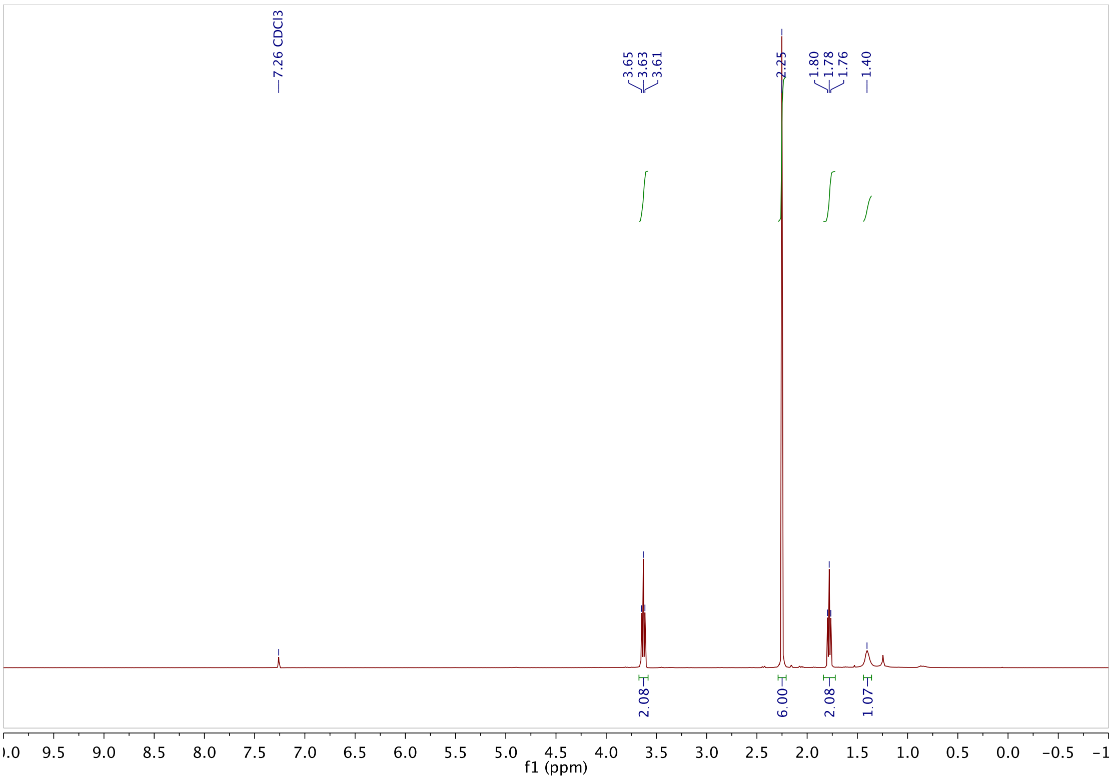

^{13}C NMR (101 MHz, $CDCl_3$)

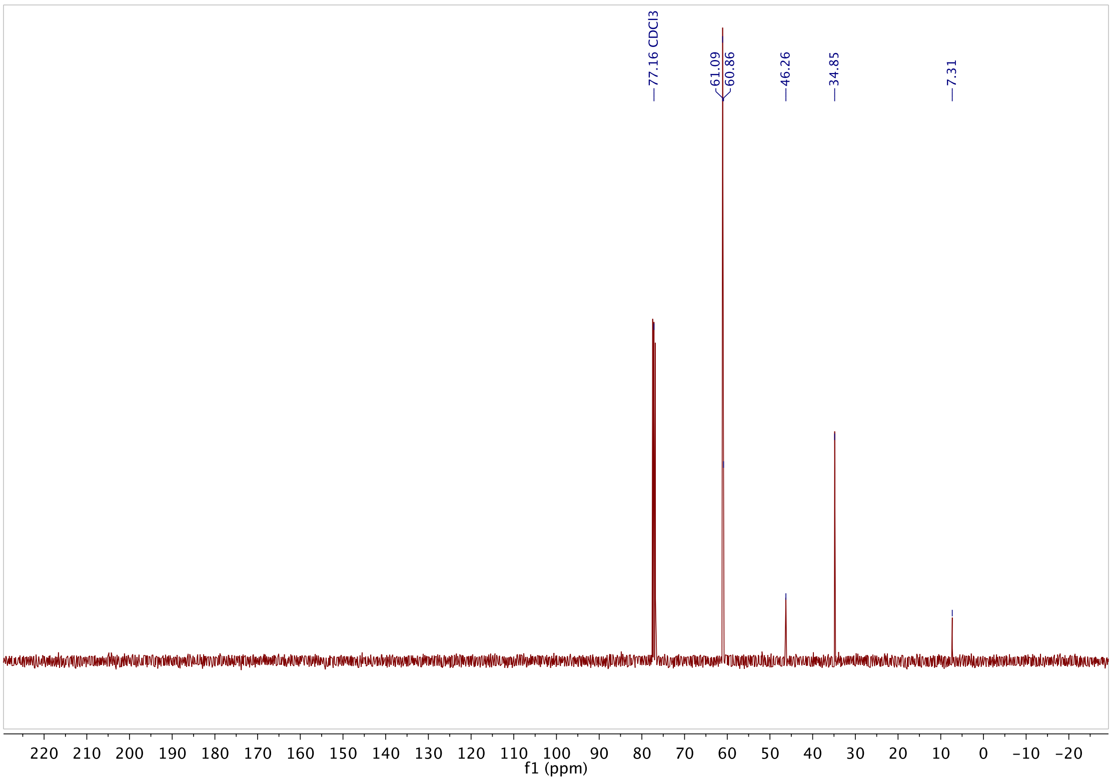

***tert*-Butyl (2-(3-iodobicyclo[1.1.1]pentan-1-yl)ethyl)carbamate, 5e**

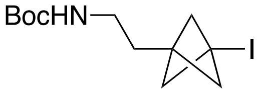

¹H NMR (400 MHz, CDCl₃)

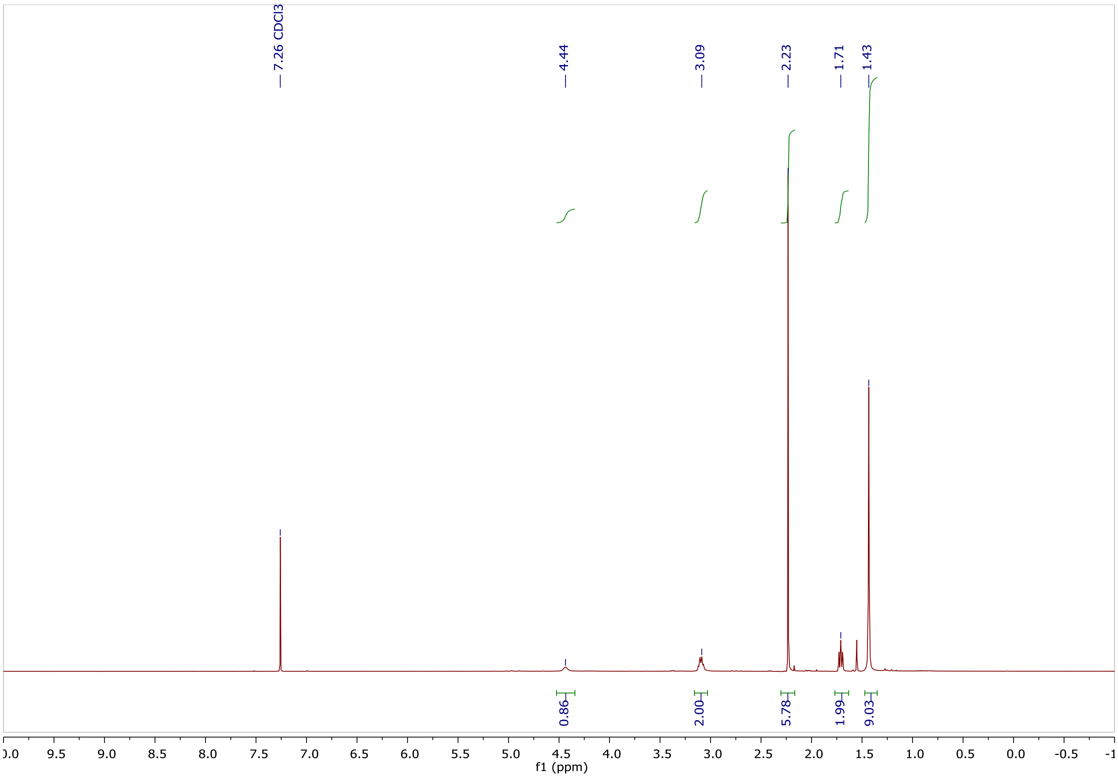

¹³C NMR (101 MHz, CDCl₃)

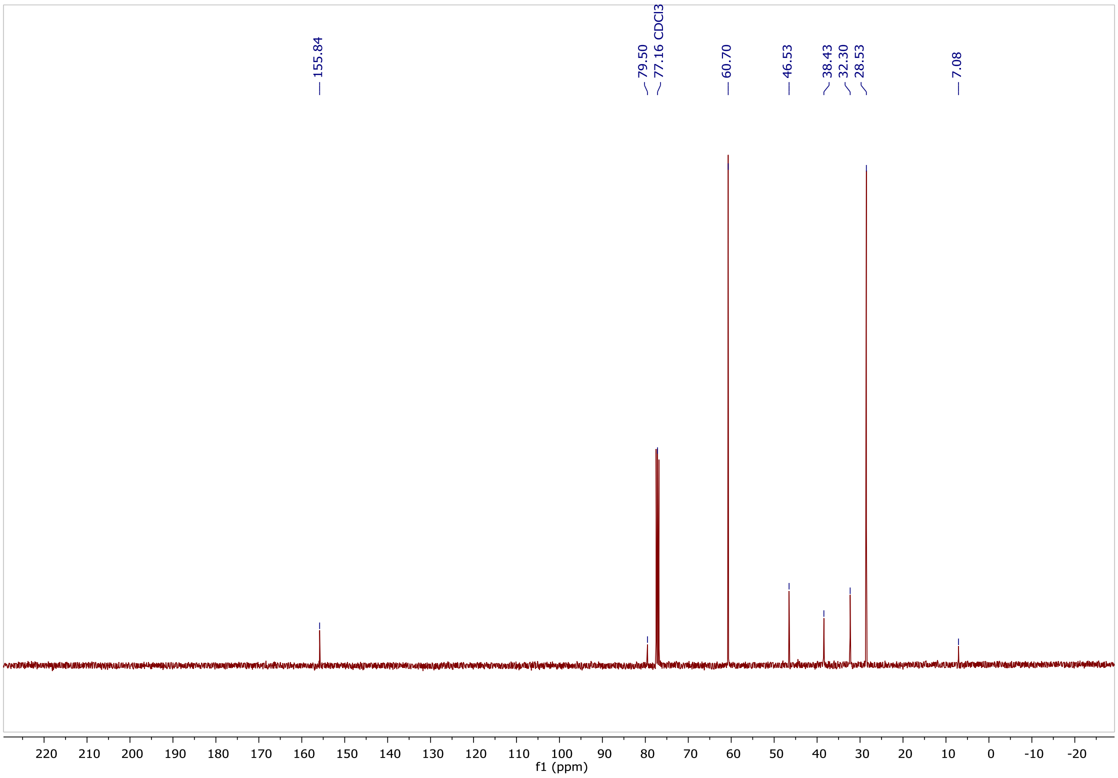

2-(3-Iodobicyclo[1.1.1]pentan-1-yl)-1-phenylethan-1-one, 5f

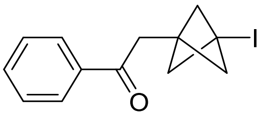

[1]**H NMR** (400 MHz, $CDCl_3$)

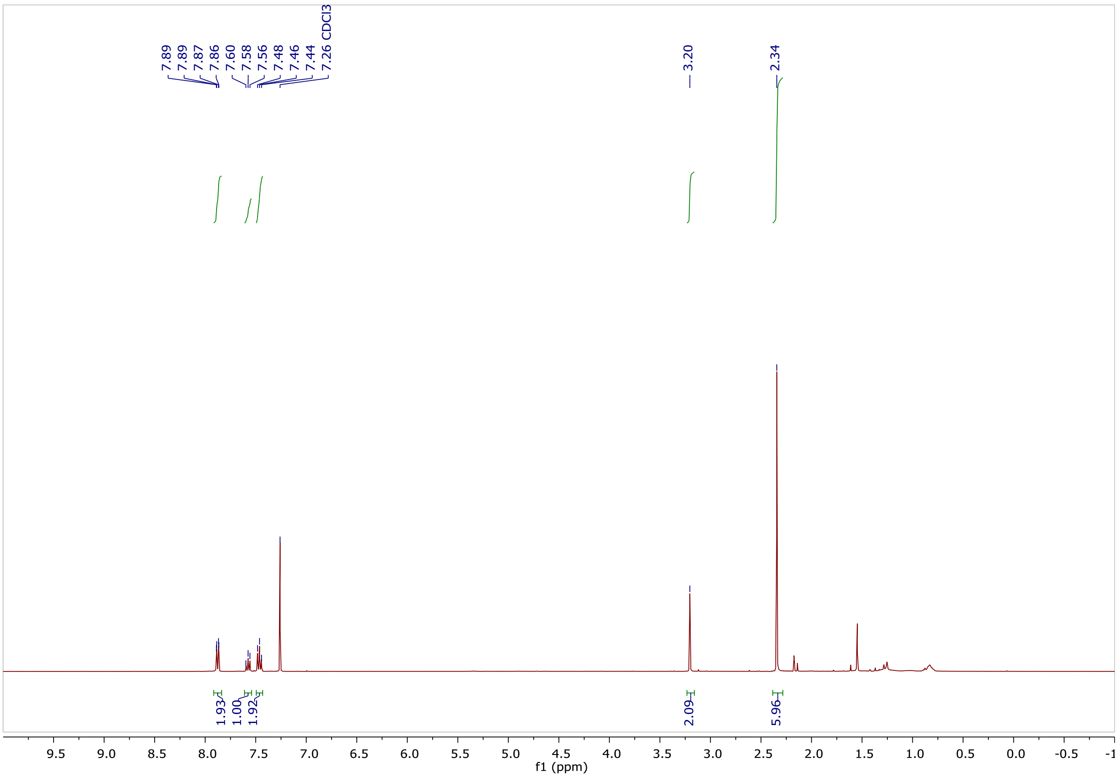

[13]**C NMR** (101 MHz, $CDCl_3$)

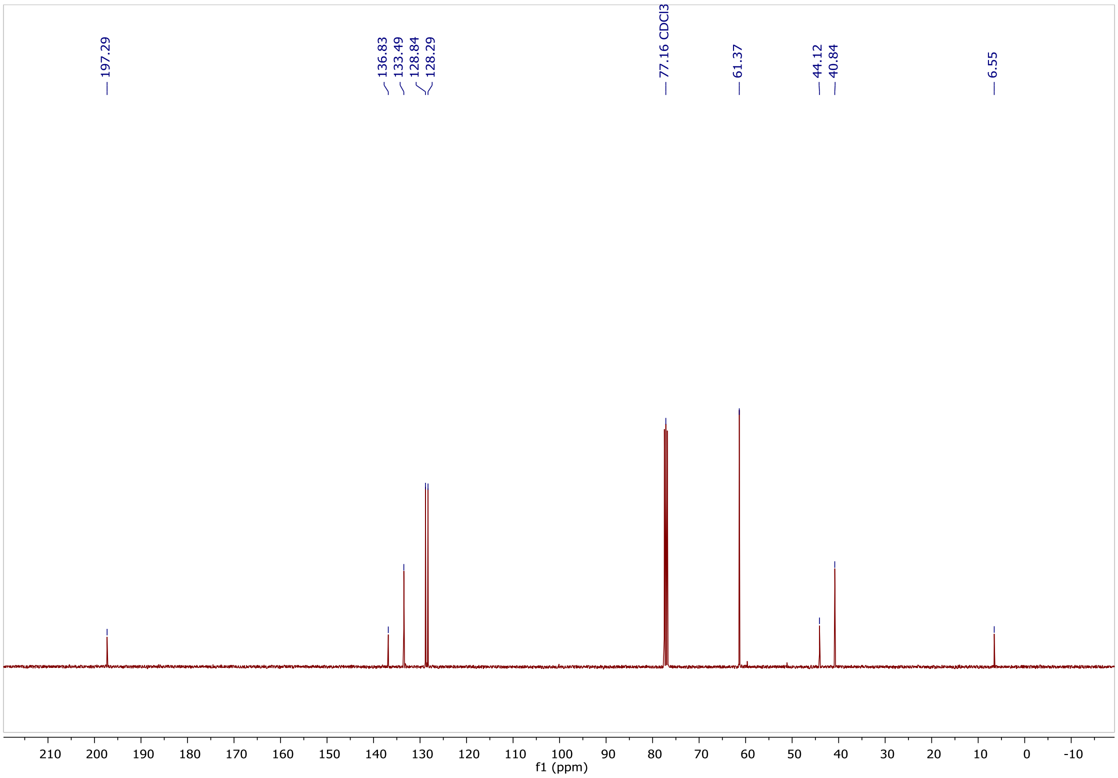

1-(Furan-2-yl)-2-(3-iodobicyclo[1.1.1]pentan-1-yl)ethan-1-one, 5g

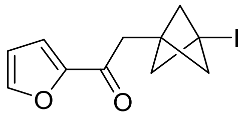

^{1}H NMR (400 MHz, $CDCl_3$)

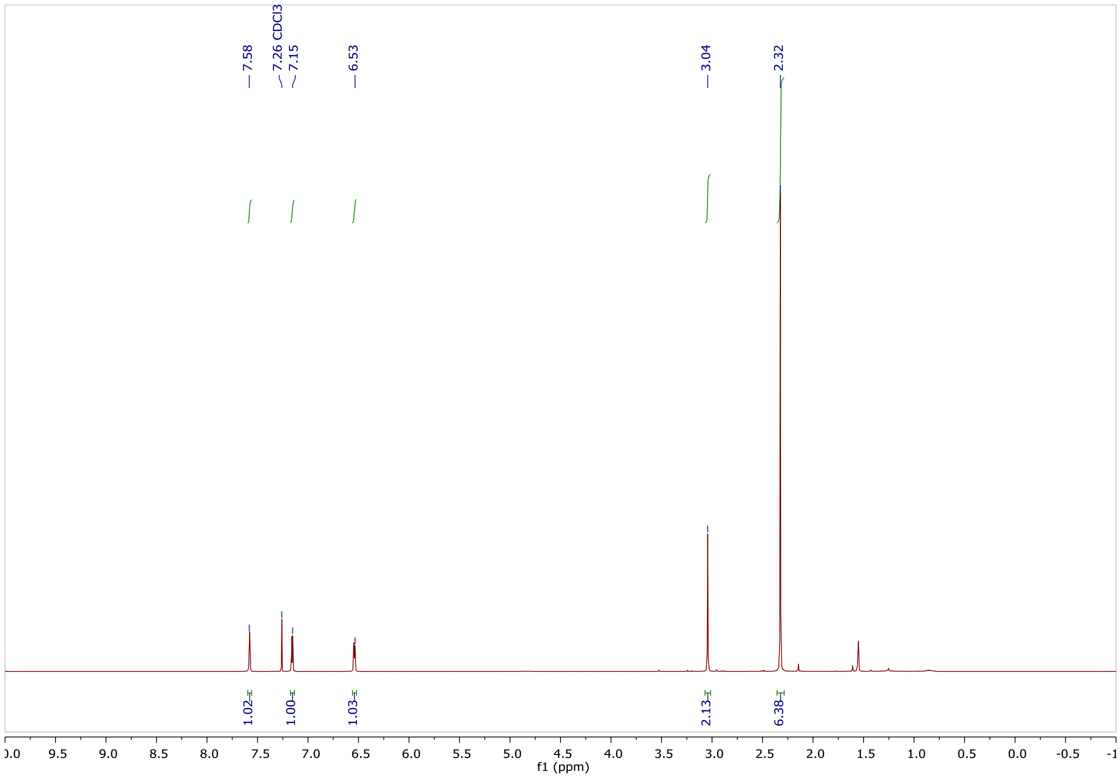

^{13}C NMR (101 MHz, $CDCl_3$)

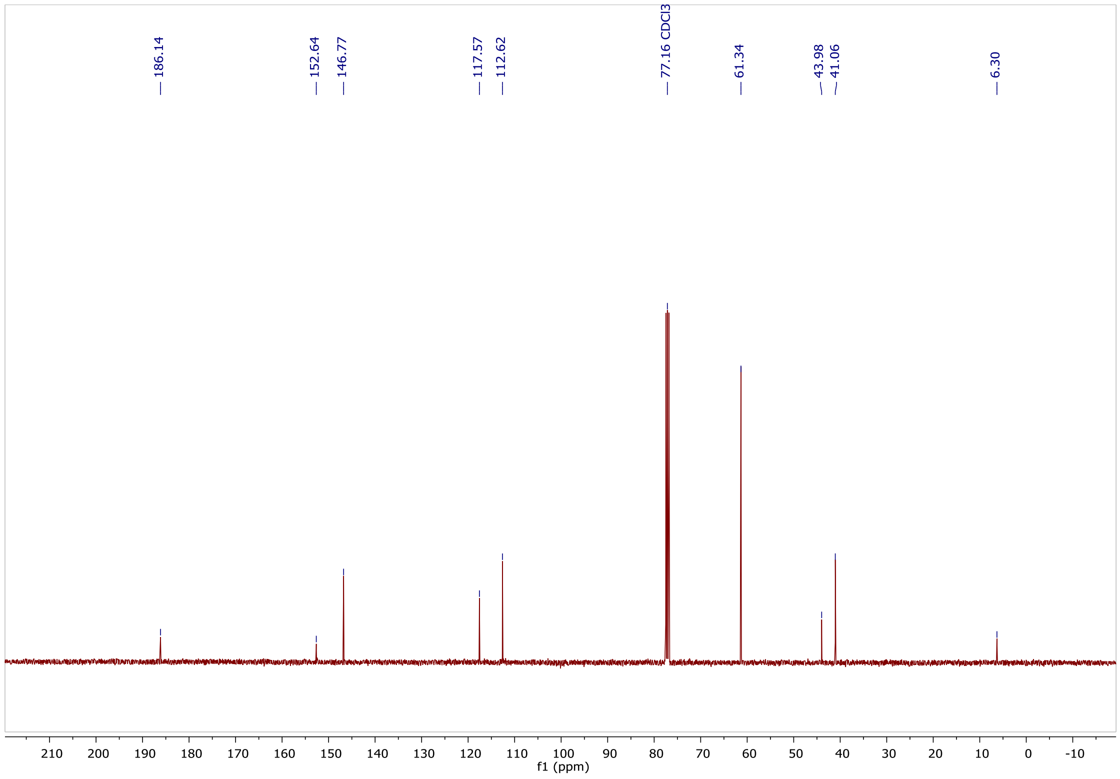

2-(3-Iodobicyclo[1.1.1]pentan-1-yl)-1-(1*H*-pyrrol-2-yl)ethan-1-one, 5h

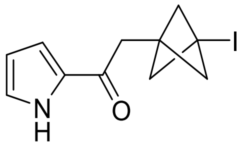

[1]**H NMR** (400 MHz, $CDCl_3$)

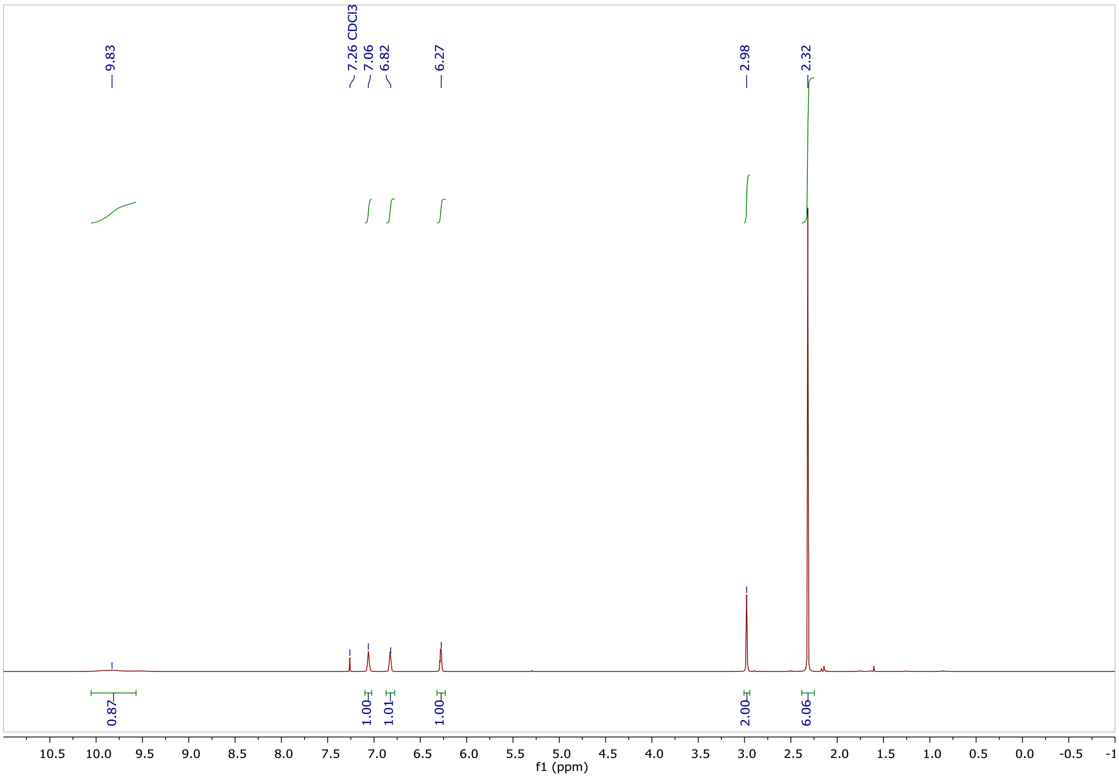

[13]**C NMR** (101 MHz, $CDCl_3$)

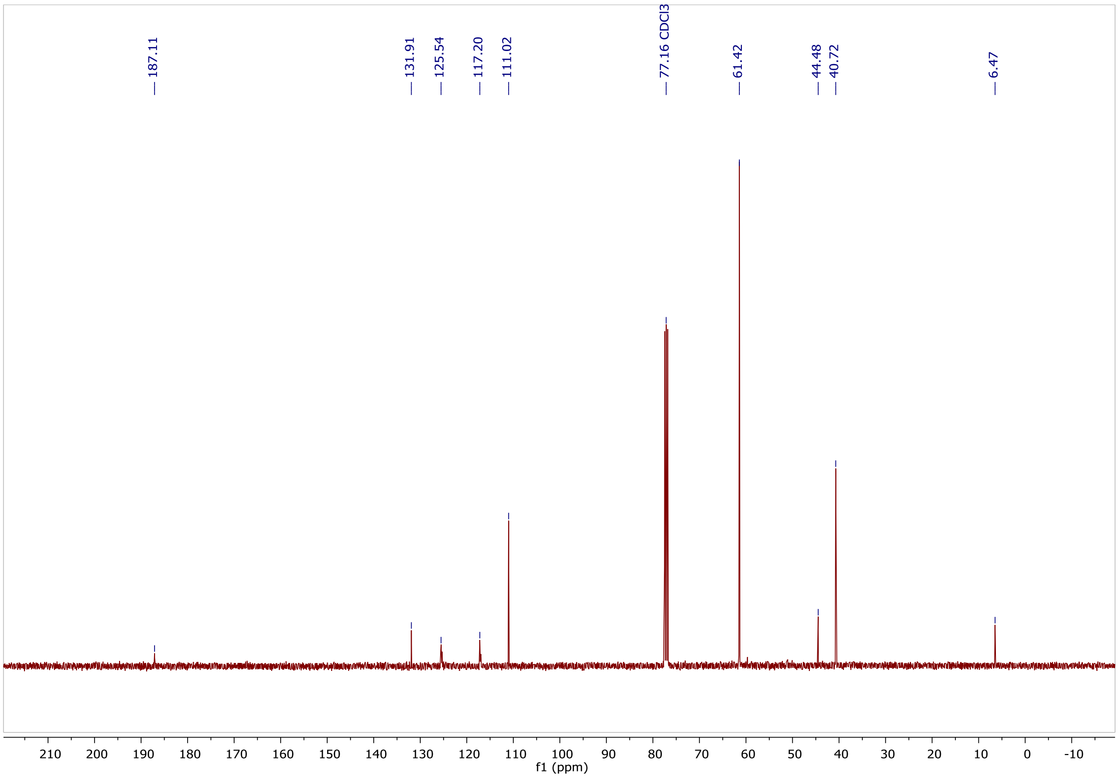

2-(3-Iodobicyclo[1.1.1]pentan-1-yl)-1-(thiophen-2-yl)ethan-1-one, 5i

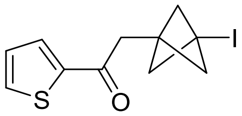

^{1}H NMR (400 MHz, $CDCl_3$)

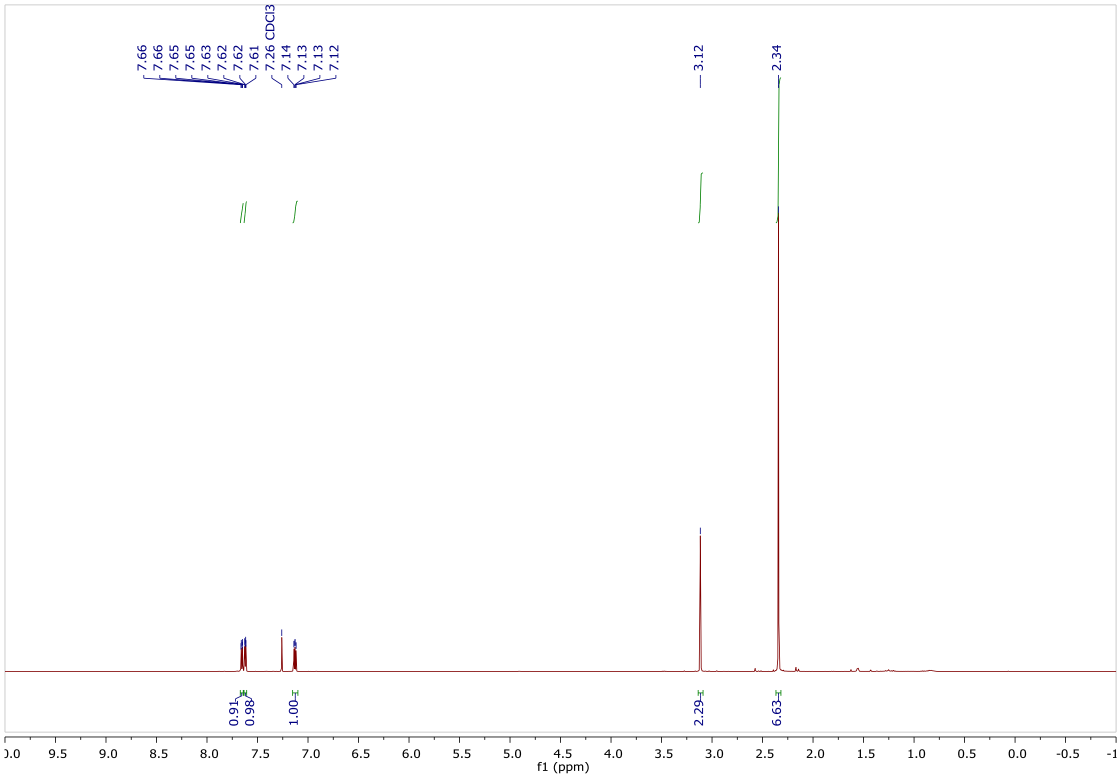

^{13}C NMR (101 MHz, $CDCl_3$)

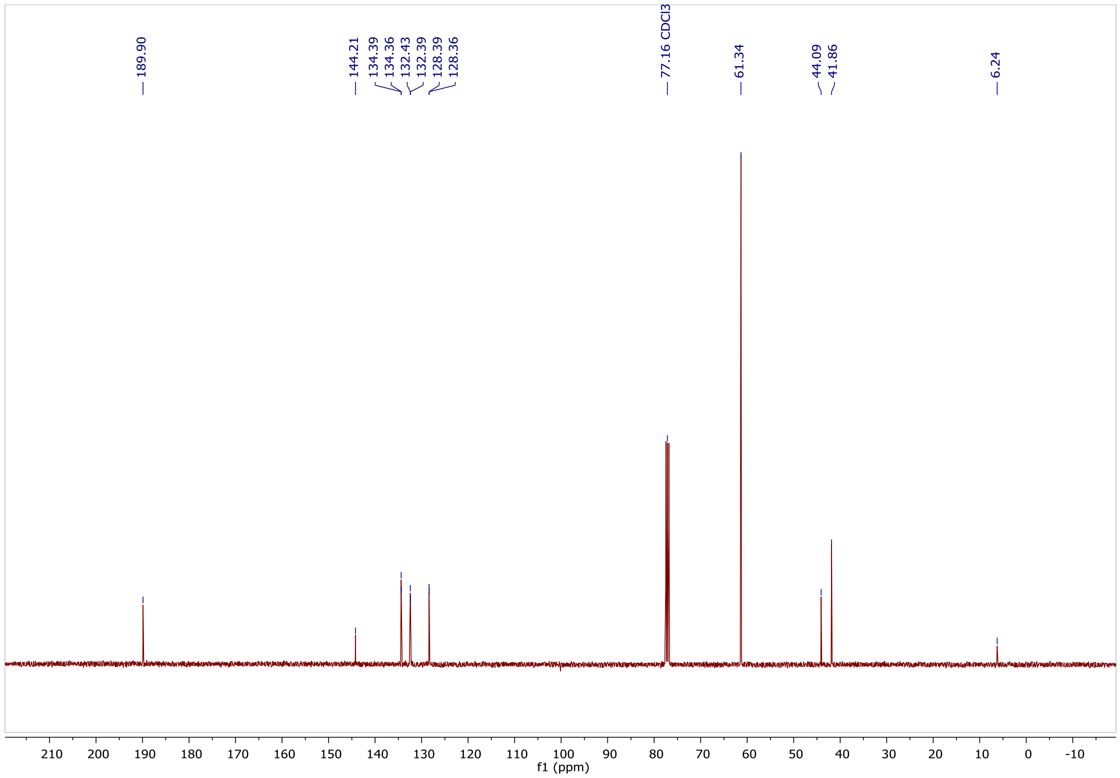

1-(Benzofuran-2-yl)-2-(3-iodobicyclo[1.1.1]pentan-1-yl)ethan-1-one, 5j

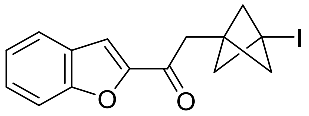

^{1}H NMR (400 MHz, $CDCl_3$)

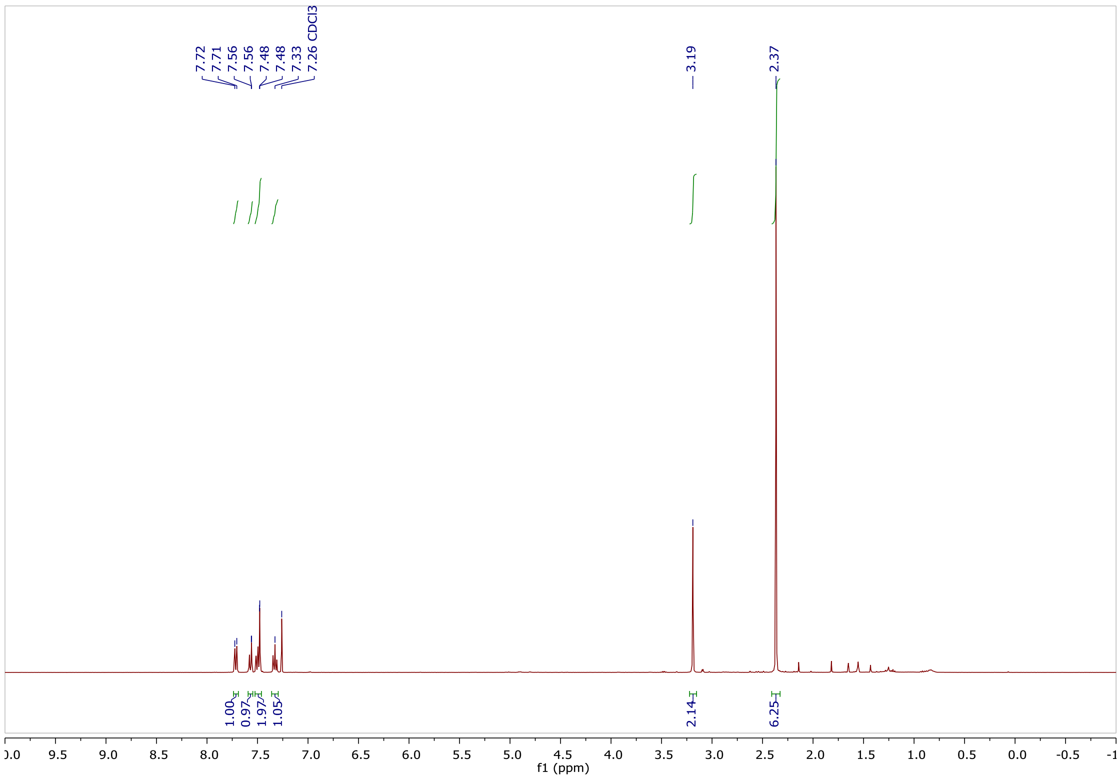

^{13}C NMR (101 MHz, $CDCl_3$)

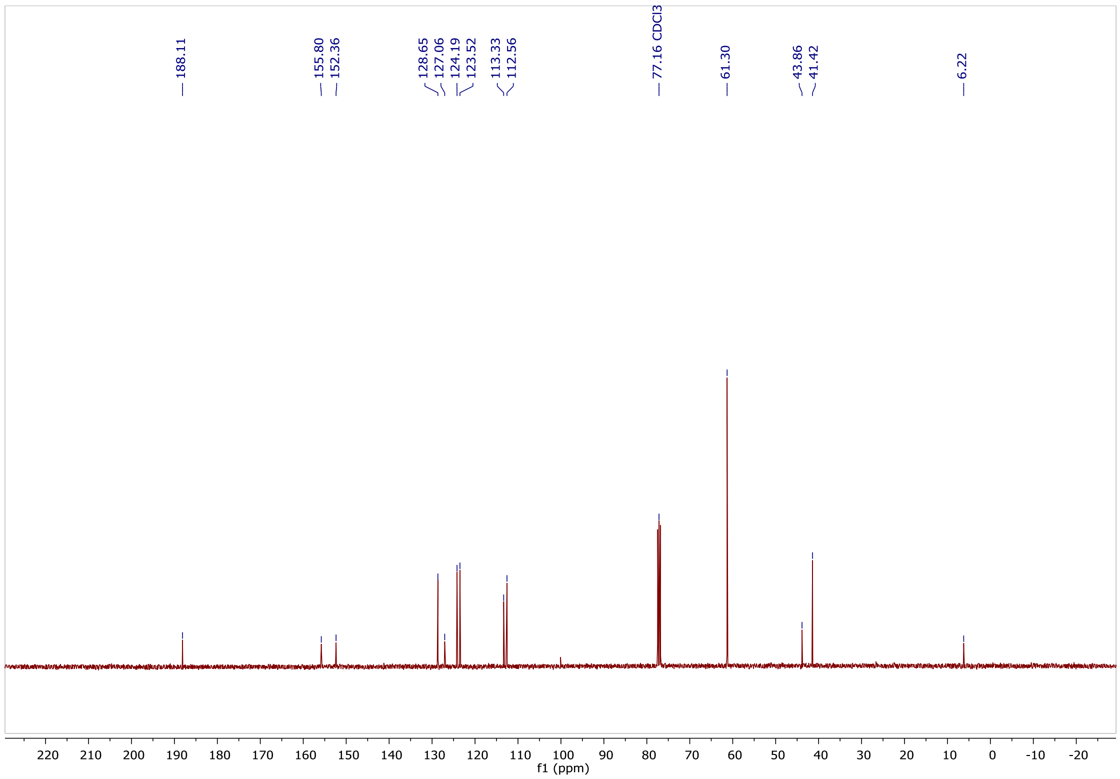

***tert*-Butyl 3-(2-(3-iodobicyclo[1.1.1]pentan-1-yl)acetyl)-1H-indole-1-carboxylate, 5k**

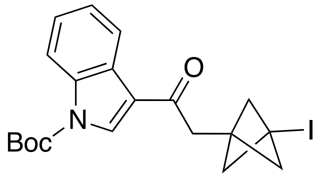

^{1}H NMR (400 MHz, $CDCl_3$)

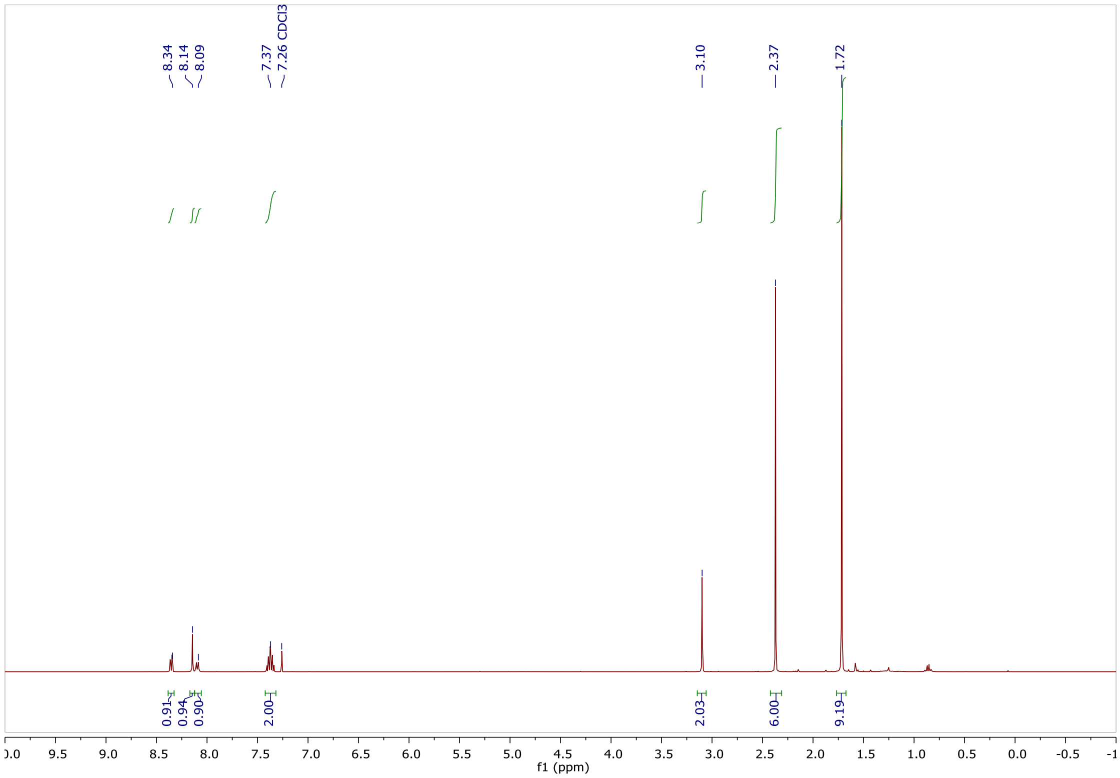

^{13}C NMR (101 MHz, $CDCl_3$)

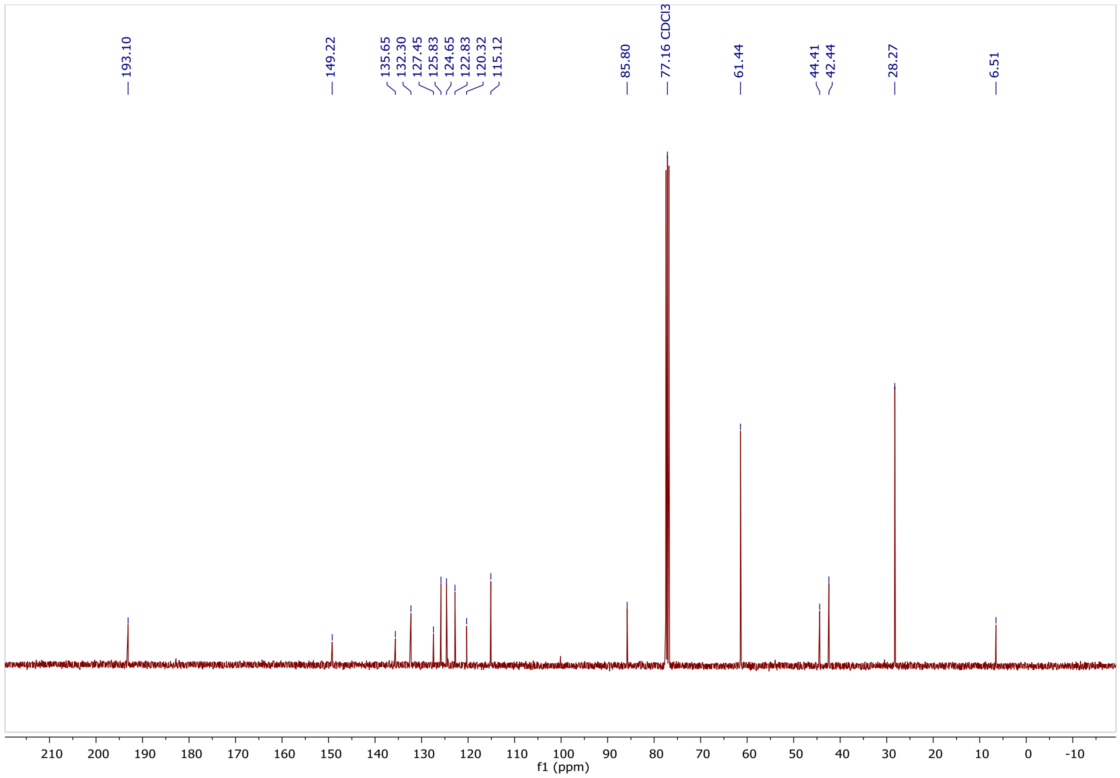

2-(3-Iodobicyclo[1.1.1]pentan-1-yl)acetamide, 5l

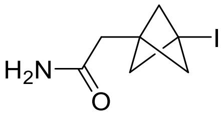

^{1}H NMR (400 MHz, $CDCl_3$)

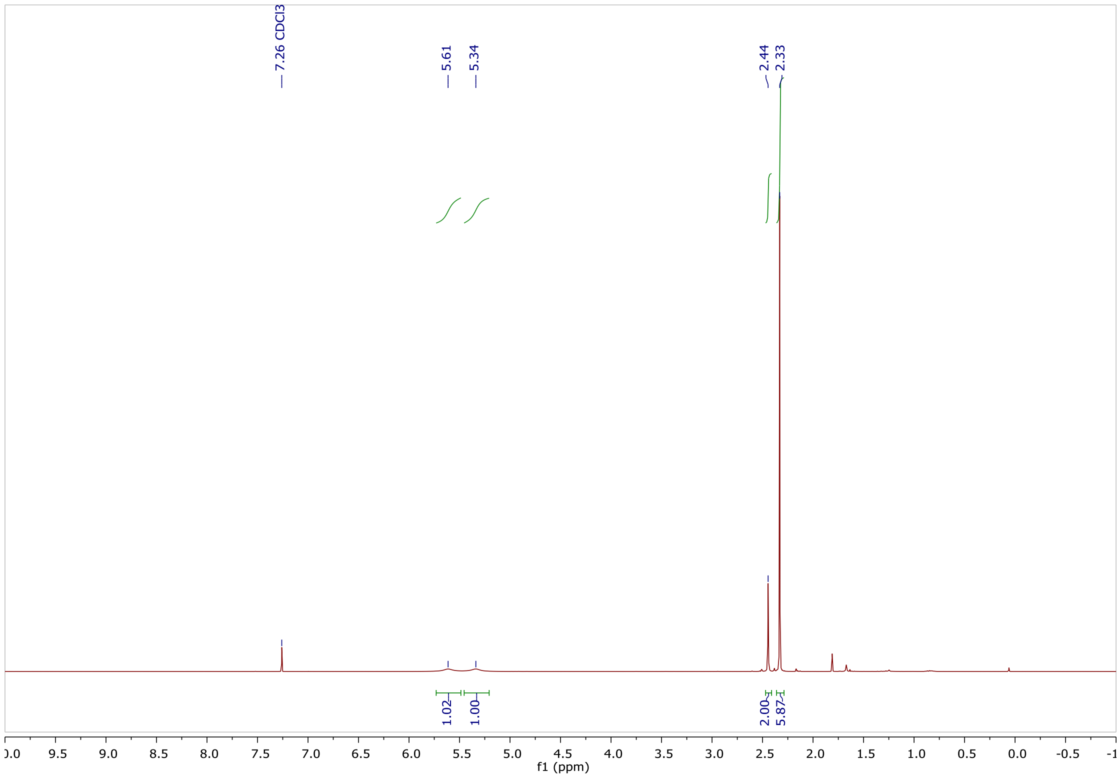

^{13}C NMR (101 MHz, $CDCl_3$)

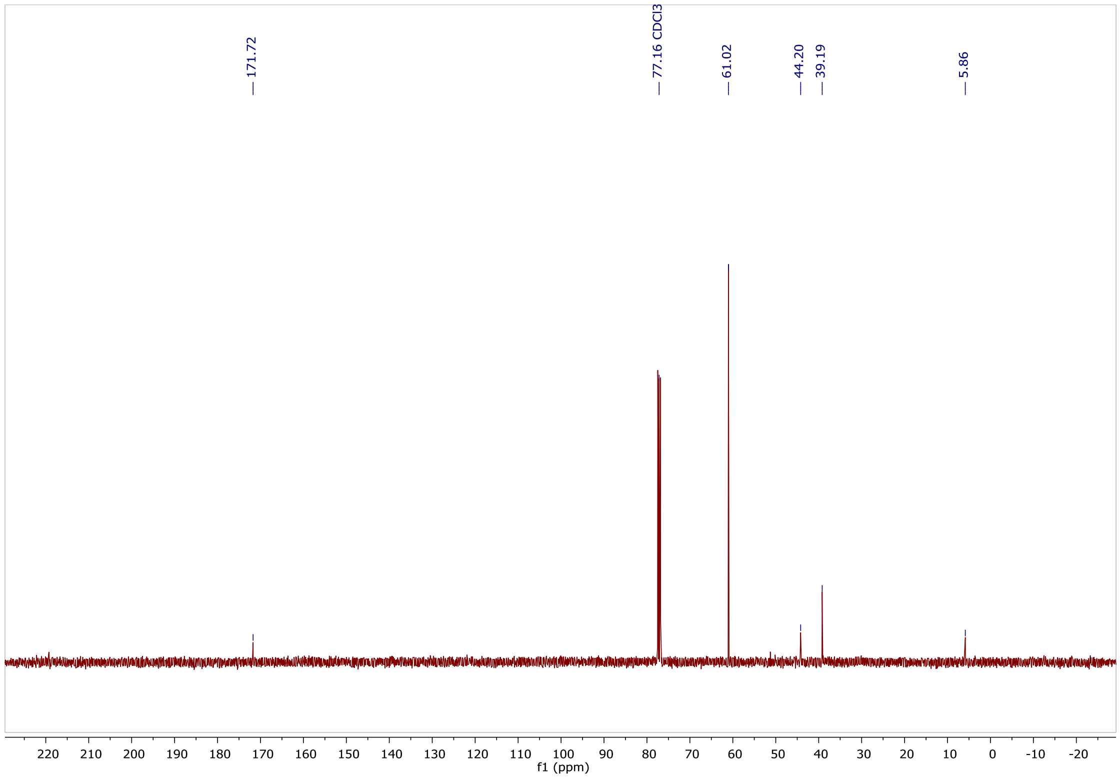

2-(3-Iodobicyclo[1.1.1]pentan-1-yl)propanamide, 5m

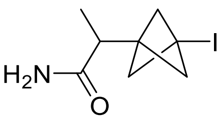

^{1}H NMR (400 MHz, $CDCl_3$)

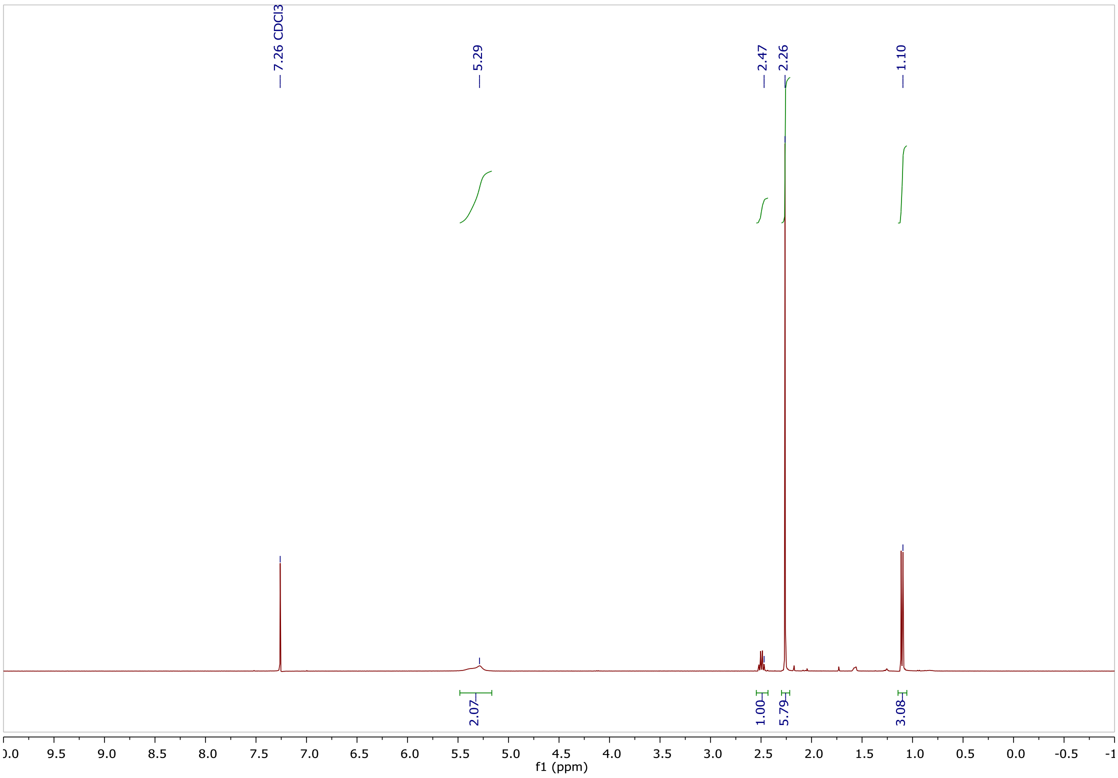

^{13}C NMR (101 MHz, $CDCl_3$)

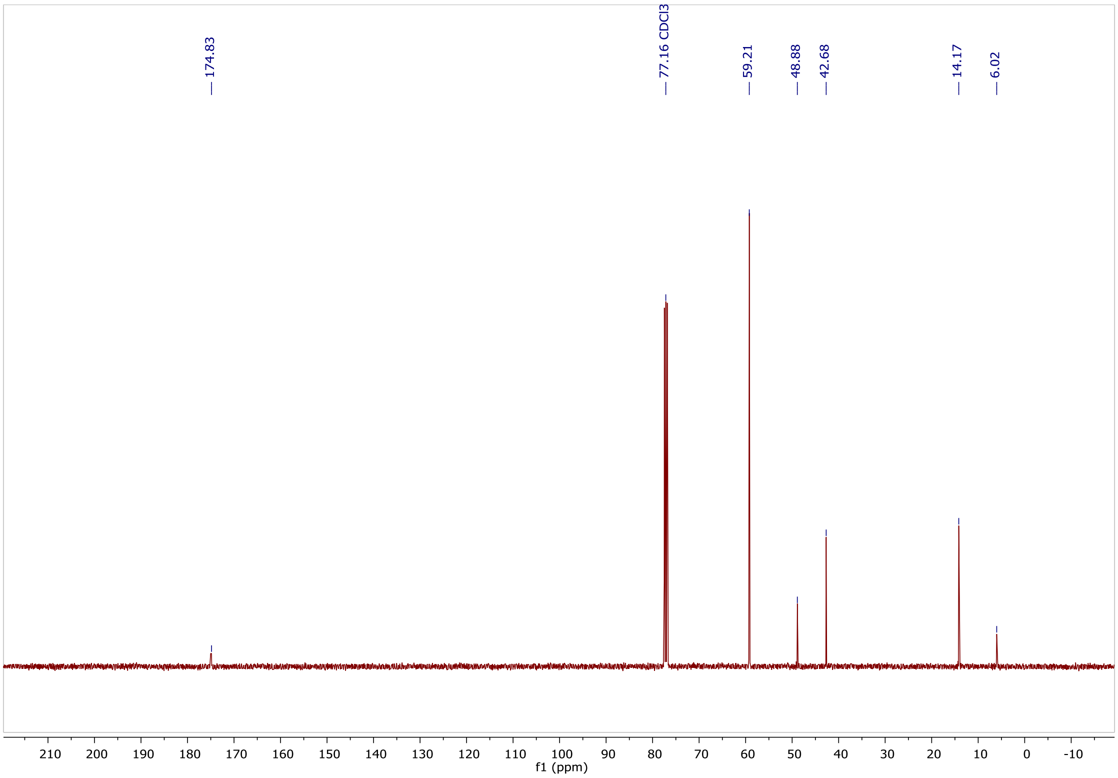

1-Iodo-3-((phenylsulfonyl)methyl)bicyclo[1.1.1]pentane, 5n

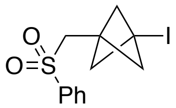

^{1}H NMR (400 MHz, $CDCl_3$)

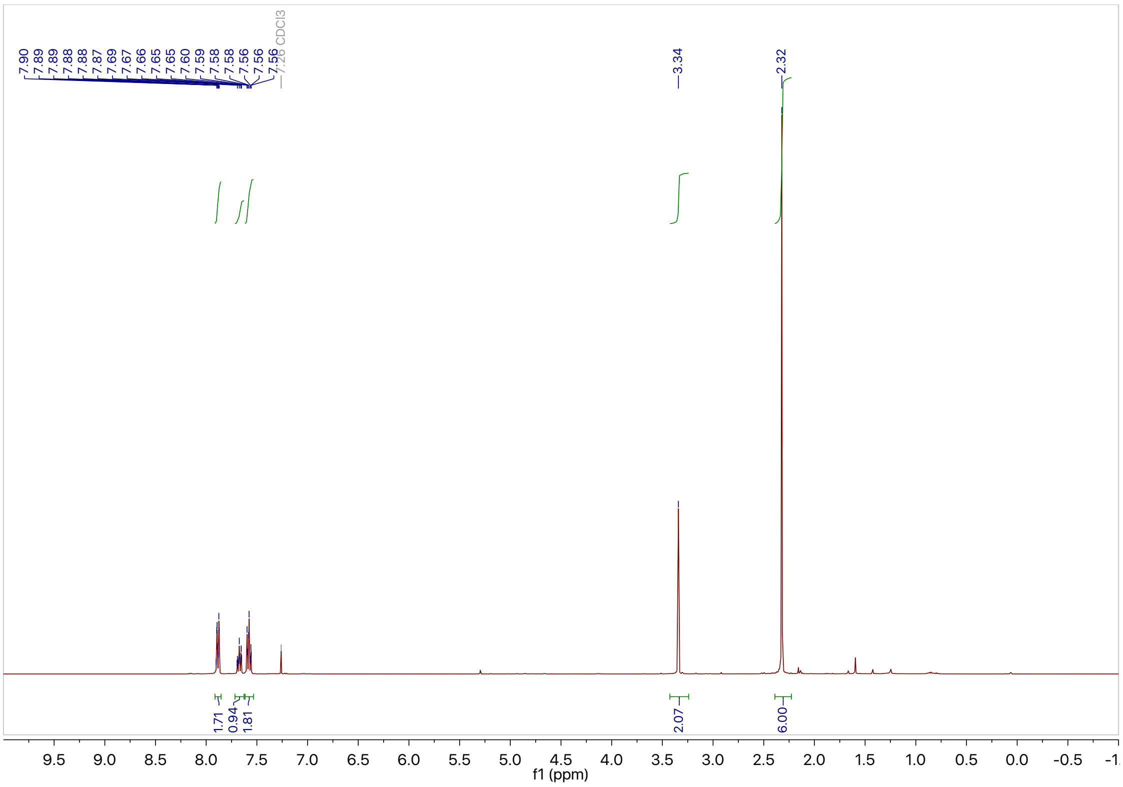

^{13}C NMR (101 MHz, $CDCl_3$)

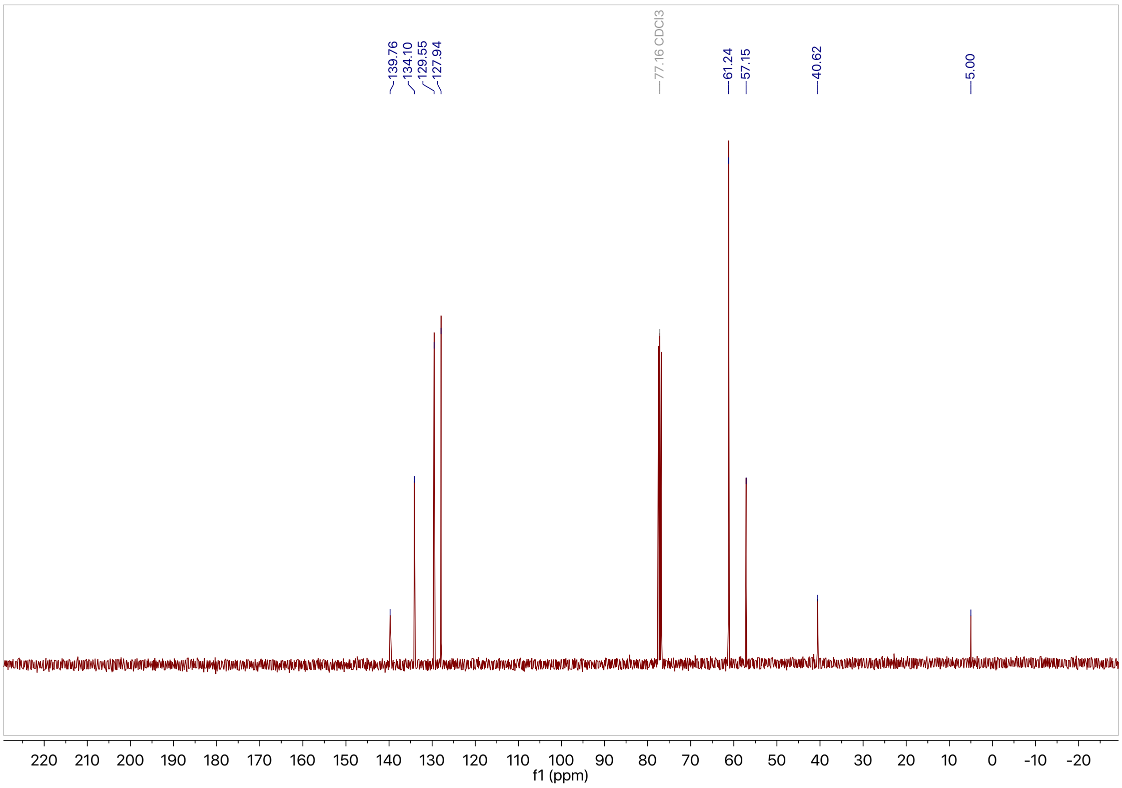

2-(3-Iodobicyclo[1.1.1]pentan-1-yl)-3-phenylpropanal, 5o

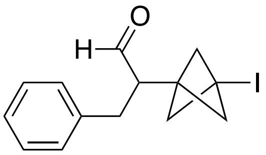

^{1}**H NMR** (400 MHz, $CDCl_3$)

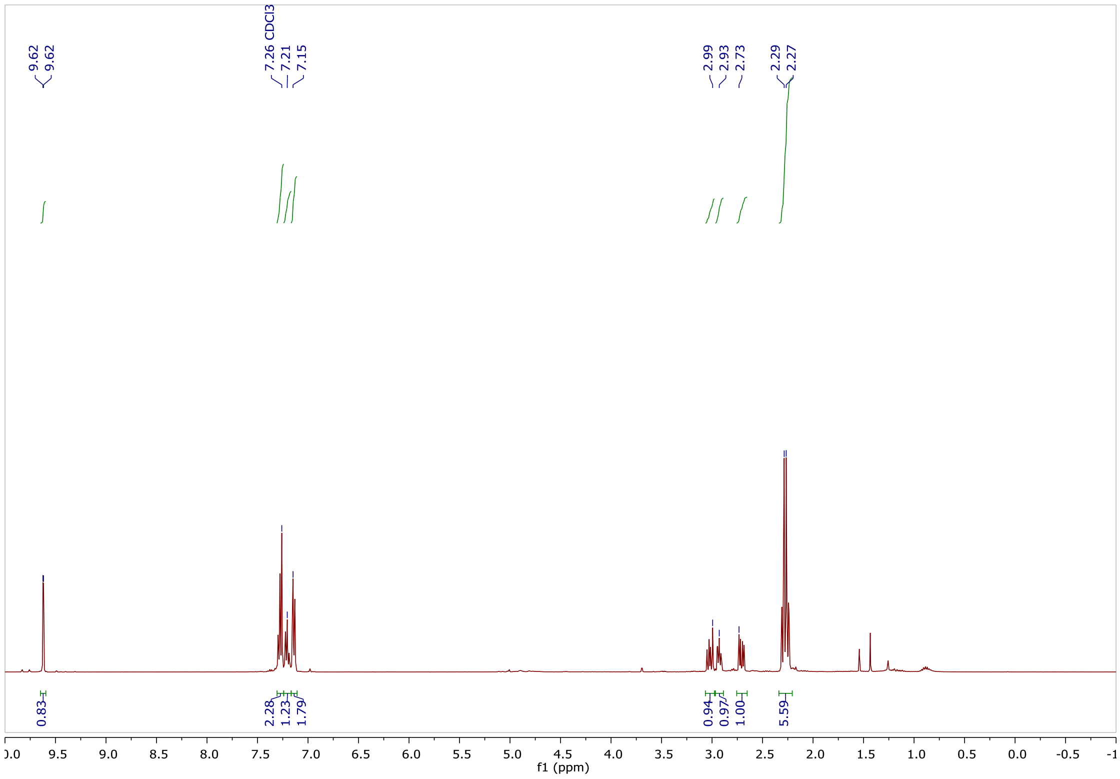

^{13}**C NMR** (101 MHz, $CDCl_3$)

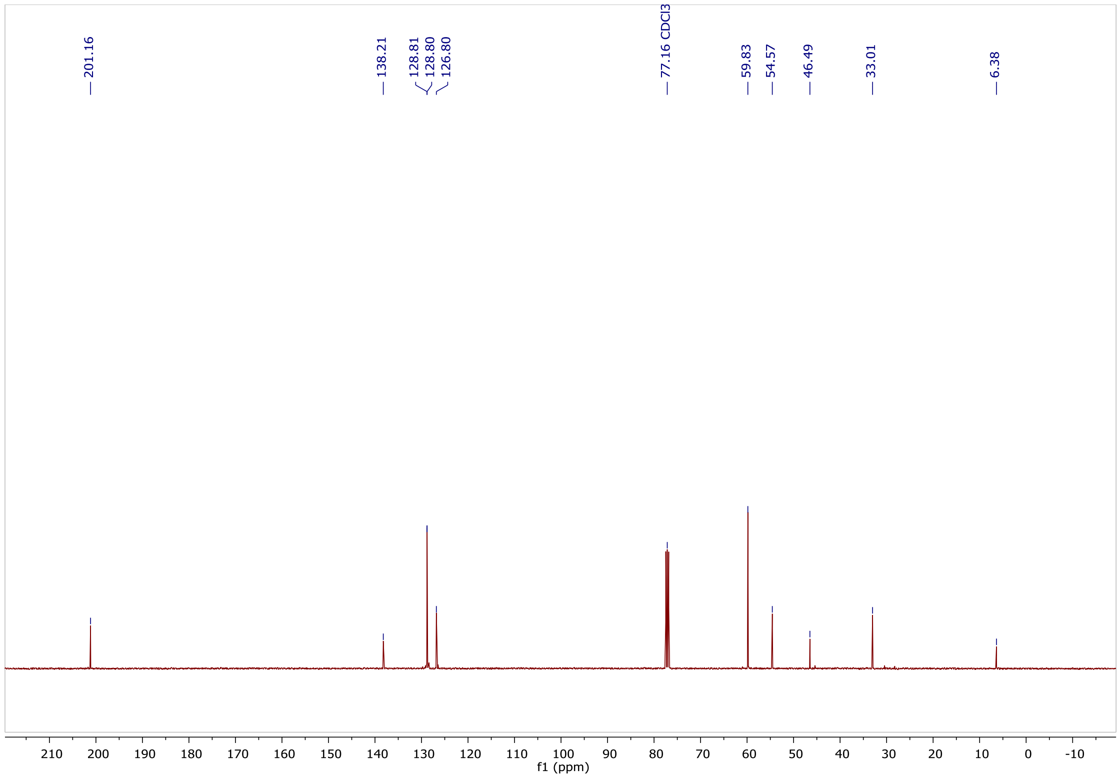

Methyl (*R*)-2-((*tert*-butoxycarbonyl)amino)-3-(3-iodobicyclo[1.1.1]pentan-1-yl)propanoate, 5p

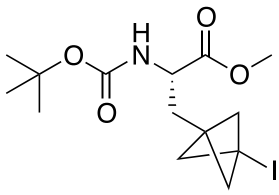

^{1}H NMR (400 MHz, $CDCl_3$)

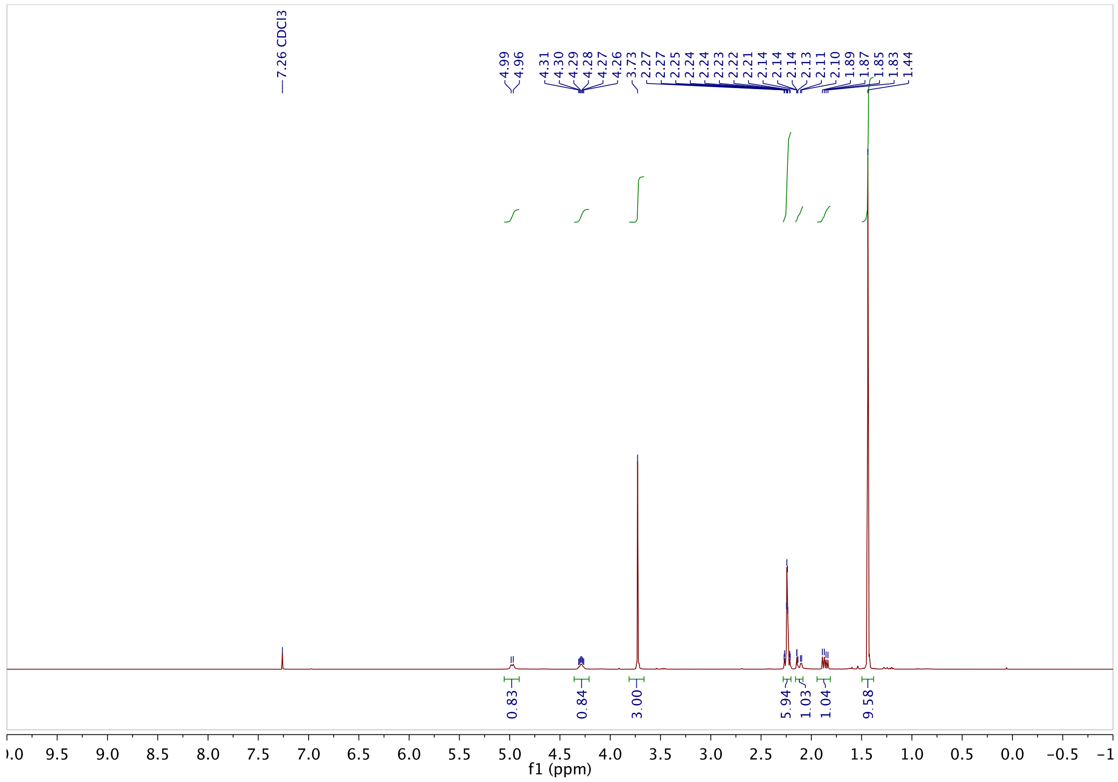

^{13}C NMR (101 MHz, $CDCl_3$)

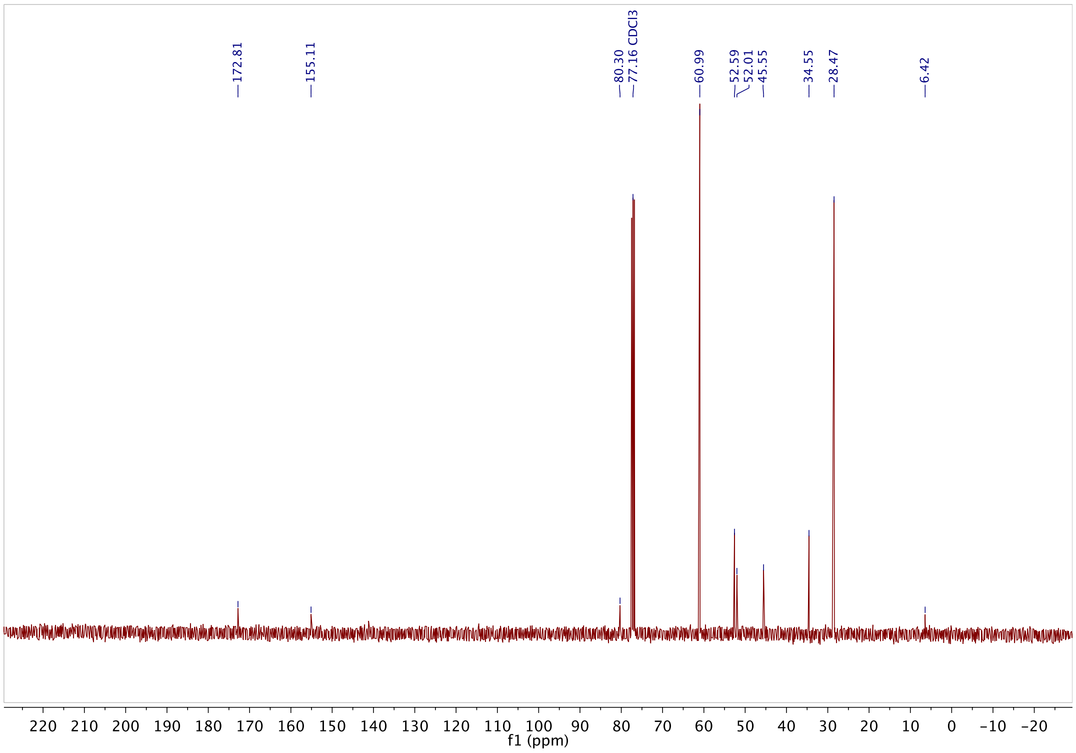

Methyl (*S*)-2-((*tert*-butoxycarbonyl)amino)-3-(3-iodobicyclo[1.1.1]pentan-1-yl)propanoate, 5p

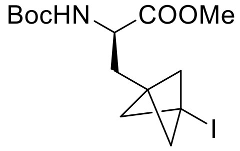

^{1}H NMR (400 MHz, $CDCl_3$)

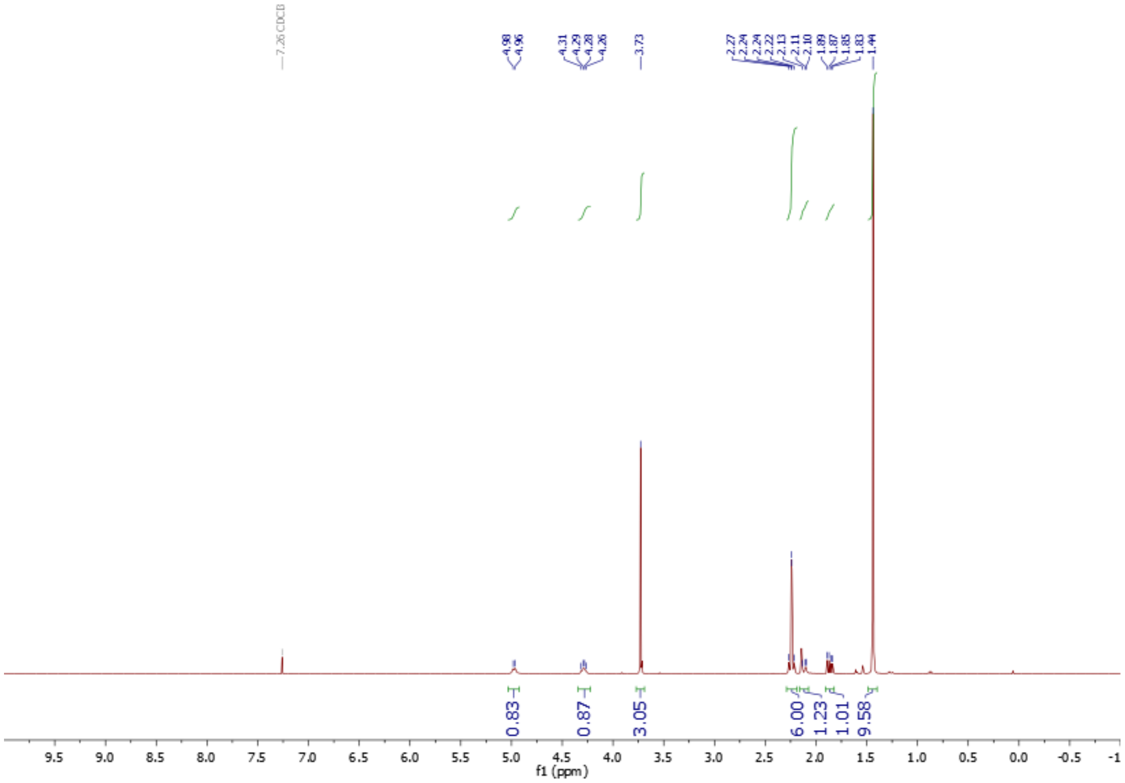

^{13}C NMR (101 MHz, $CDCl_3$)

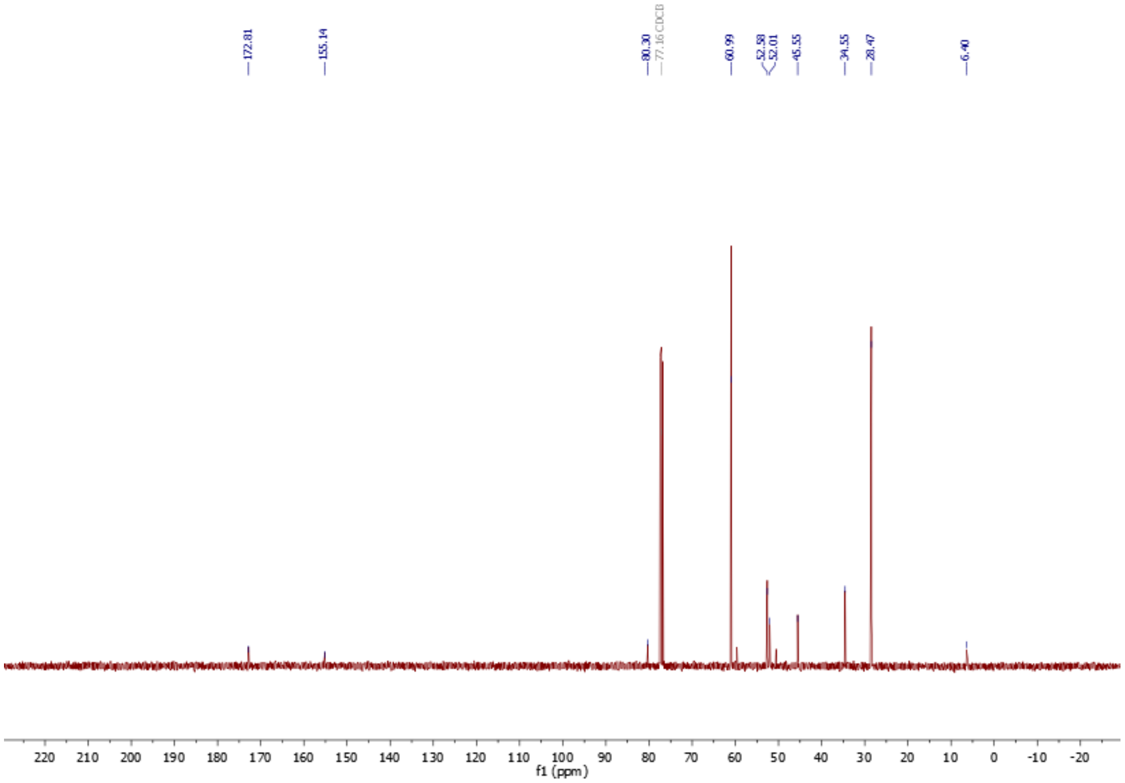

1-(chloromethyl)-3-iodobicyclo[1.1.1]pentane, 5q

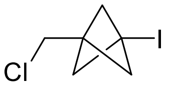

^{1}H NMR (400 MHz, $CDCl_3$)

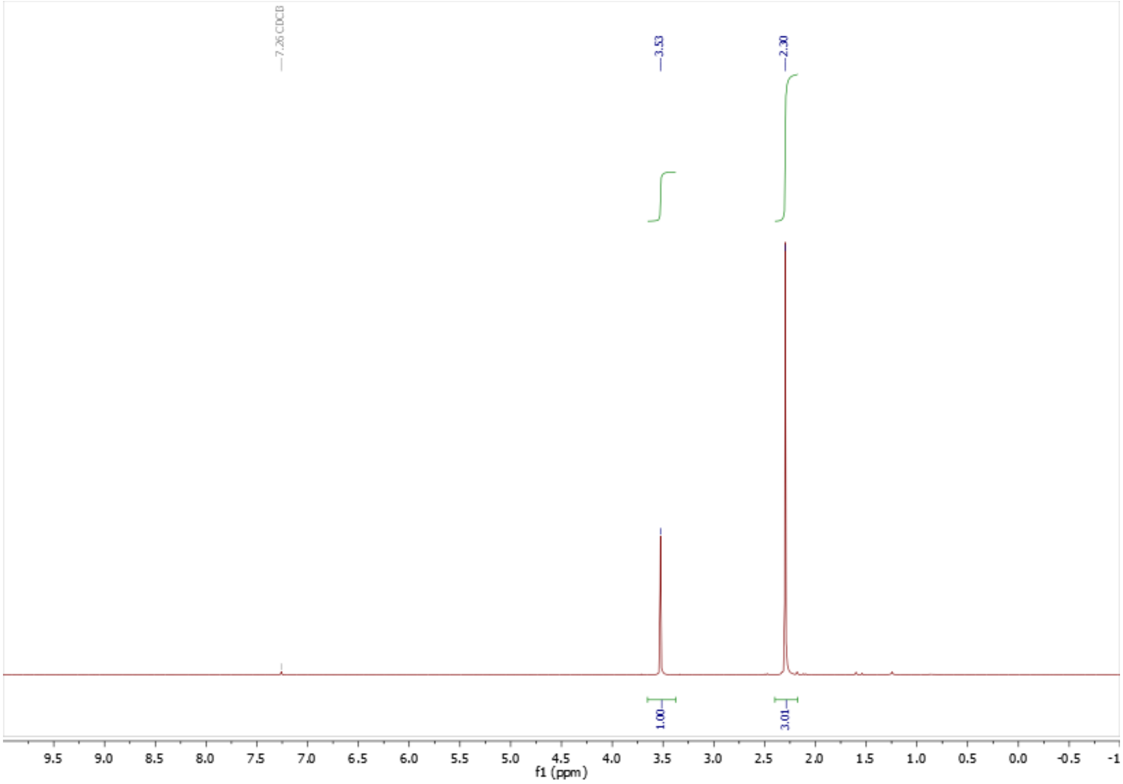

^{13}C NMR (101 MHz, $CDCl_3$)

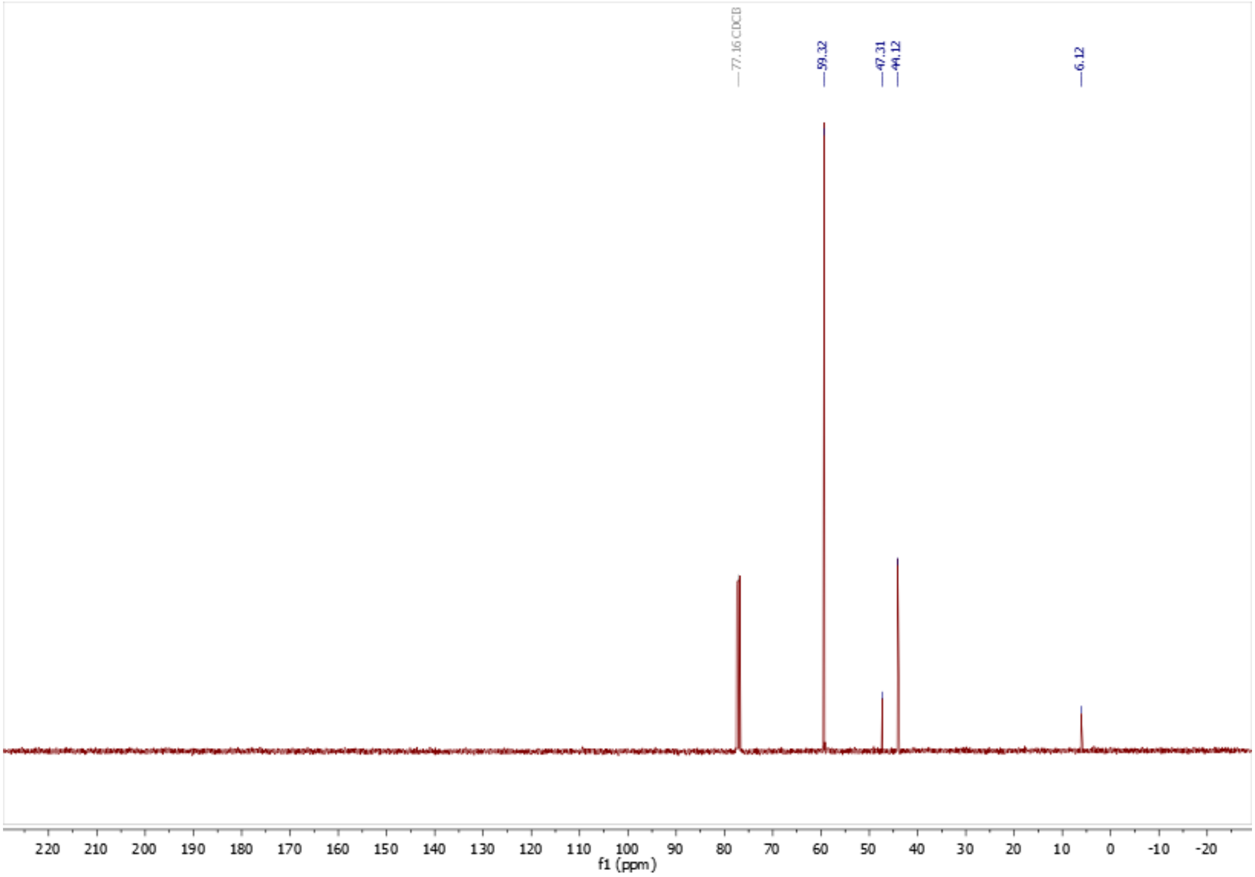

1-Iodo-3-(4-nitrobenzyl)bicyclo[1.1.1]pentane, 5r

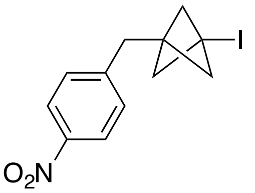

^{1}H NMR (400 MHz, CDCl3)

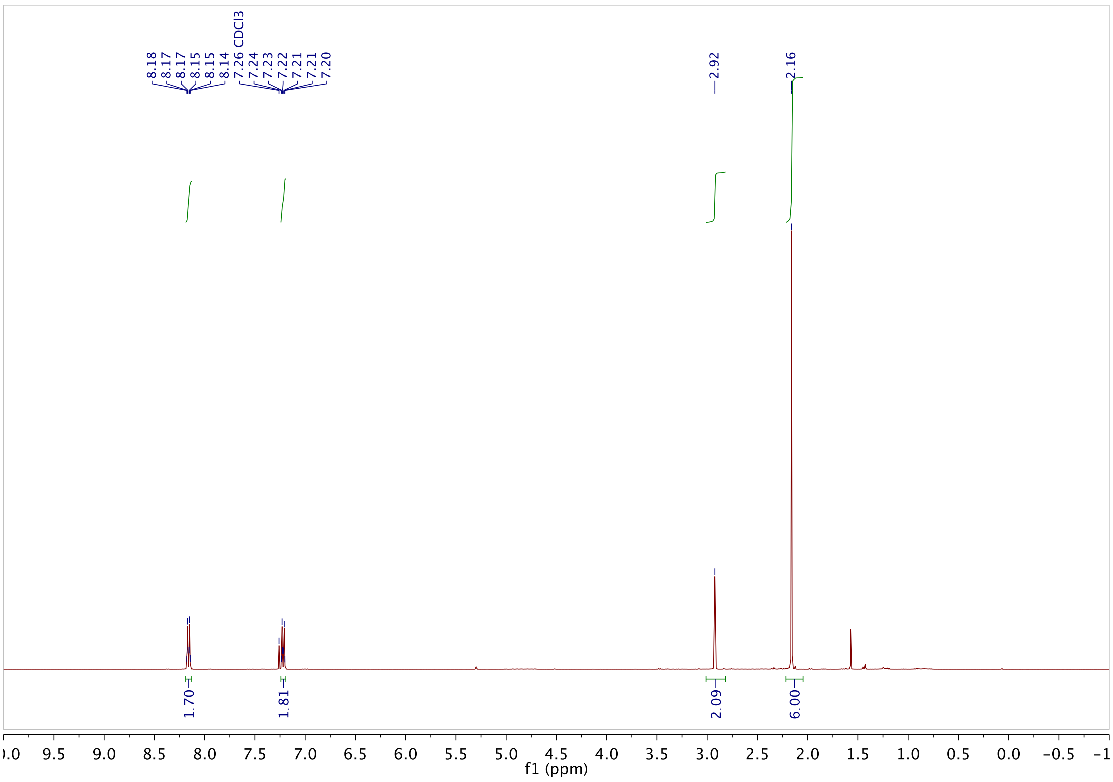

^{13}C NMR (101 MHz, CDCl3)

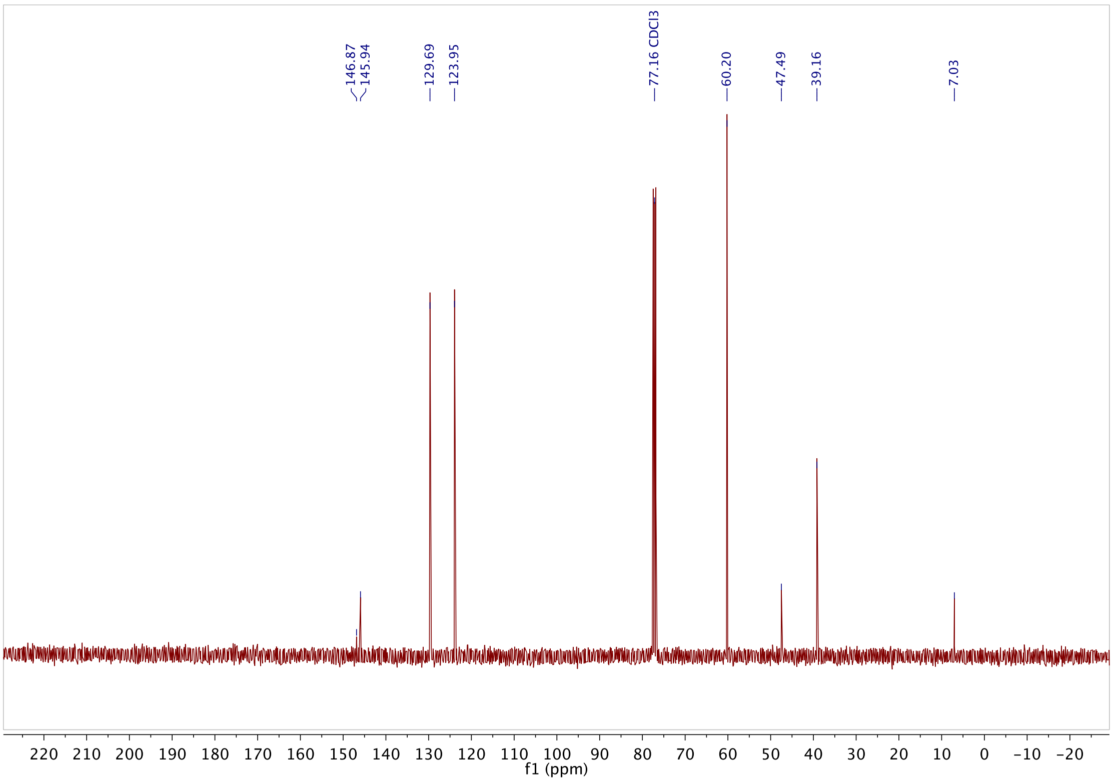

1-Iodo-3-(4-(trifluoromethyl)benzyl)bicyclo[1.1.1]pentane, 5s

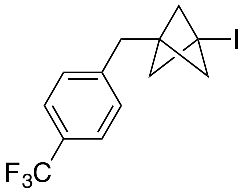

^{1}H NMR (400 MHz, CDCl$_3$)

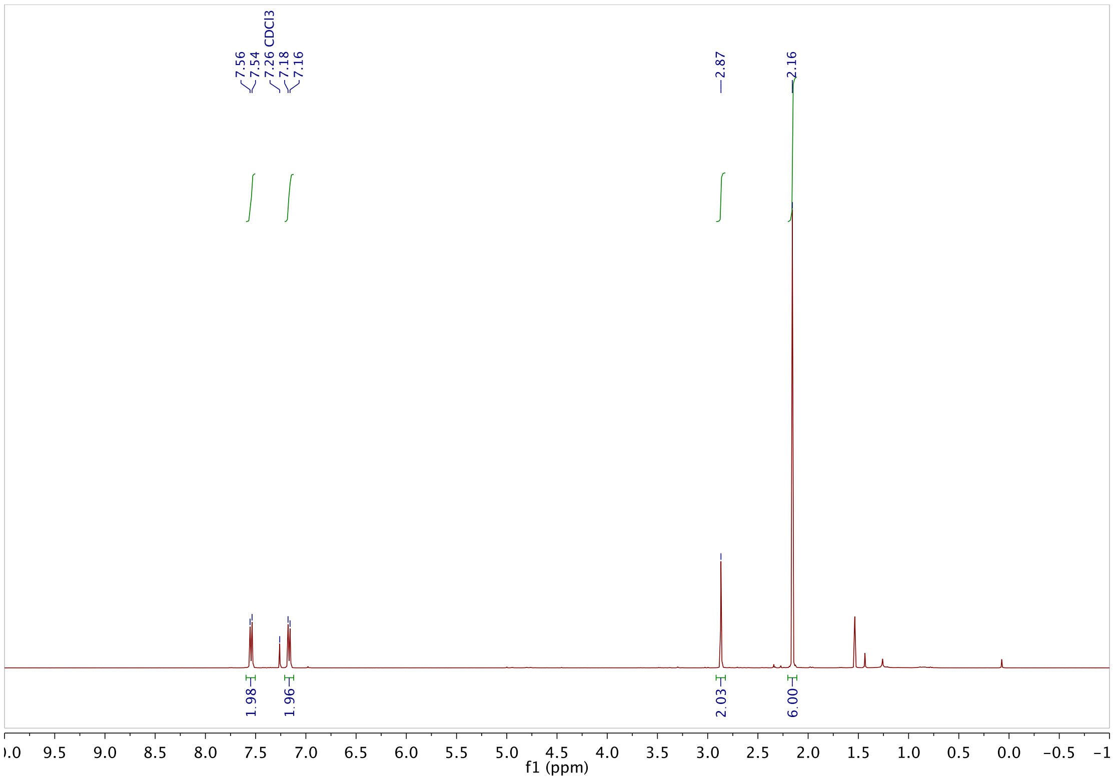

^{13}C NMR (101 MHz, CDCl$_3$)

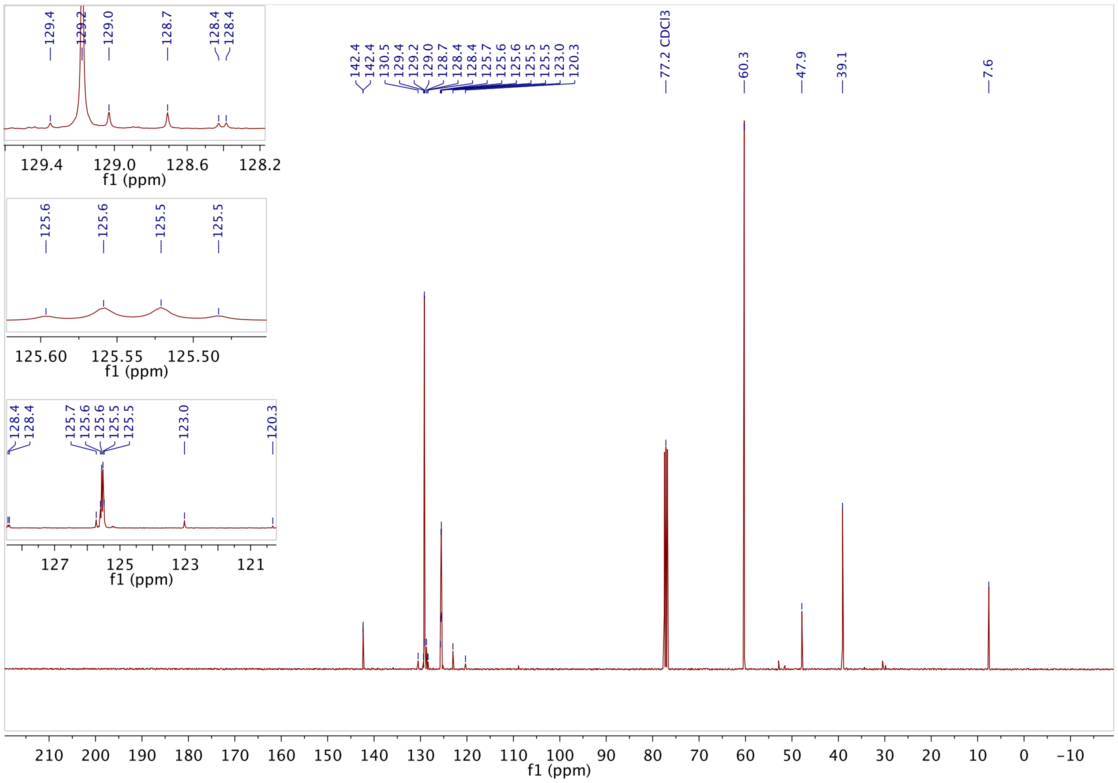

^{19}F NMR (376 MHz, $CDCl_3$)

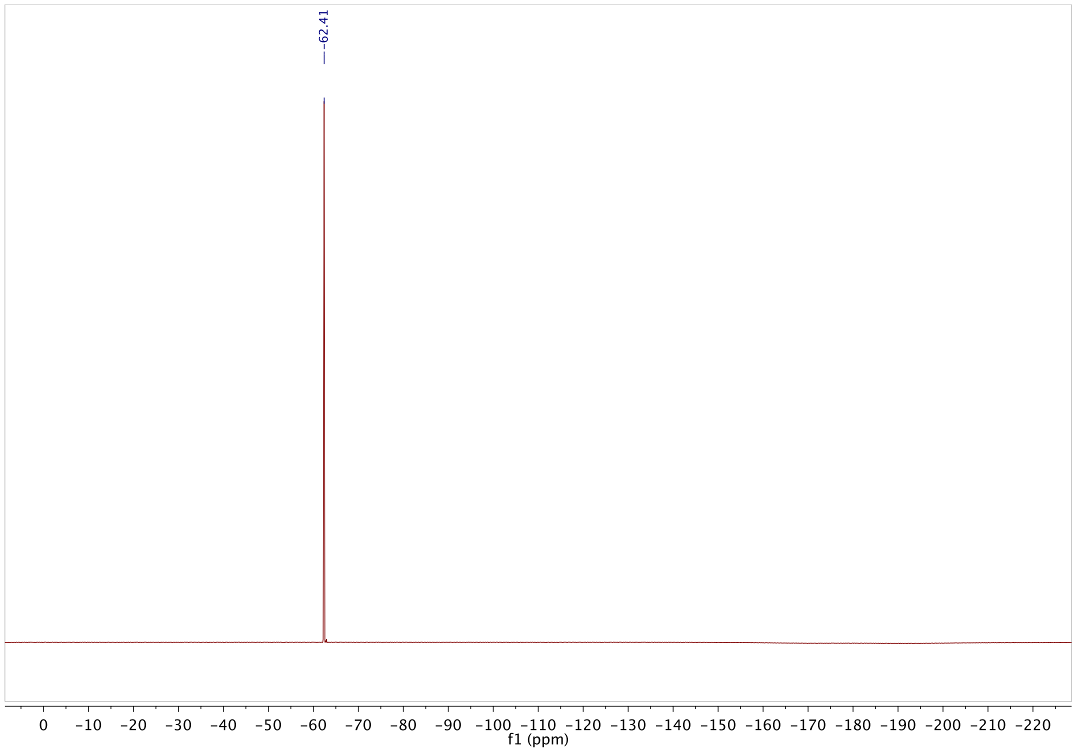

1-(3,5-Bis(trifluoromethyl)benzyl)-3-iodobicyclo[1.1.1]pentane, 5t

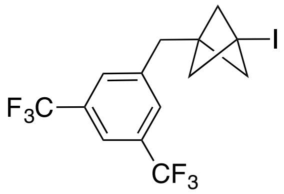

^{1}H NMR (400 MHz, CDCl$_3$)

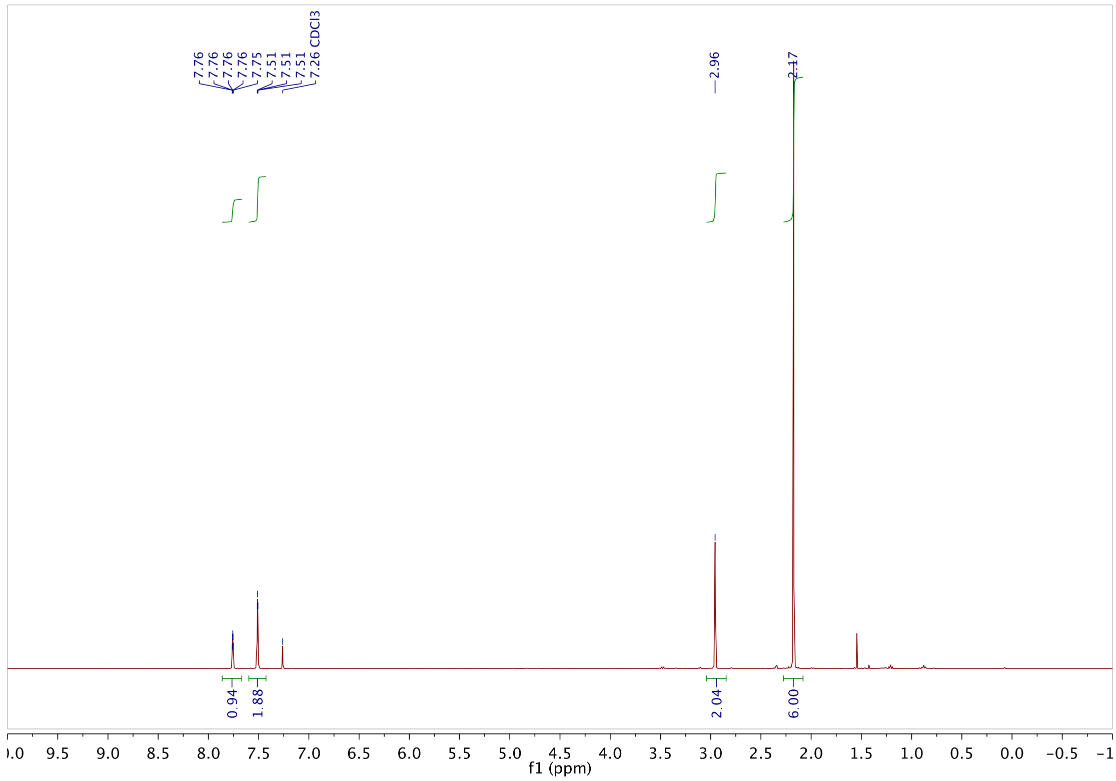

^{13}C NMR (101 MHz, CDCl$_3$)

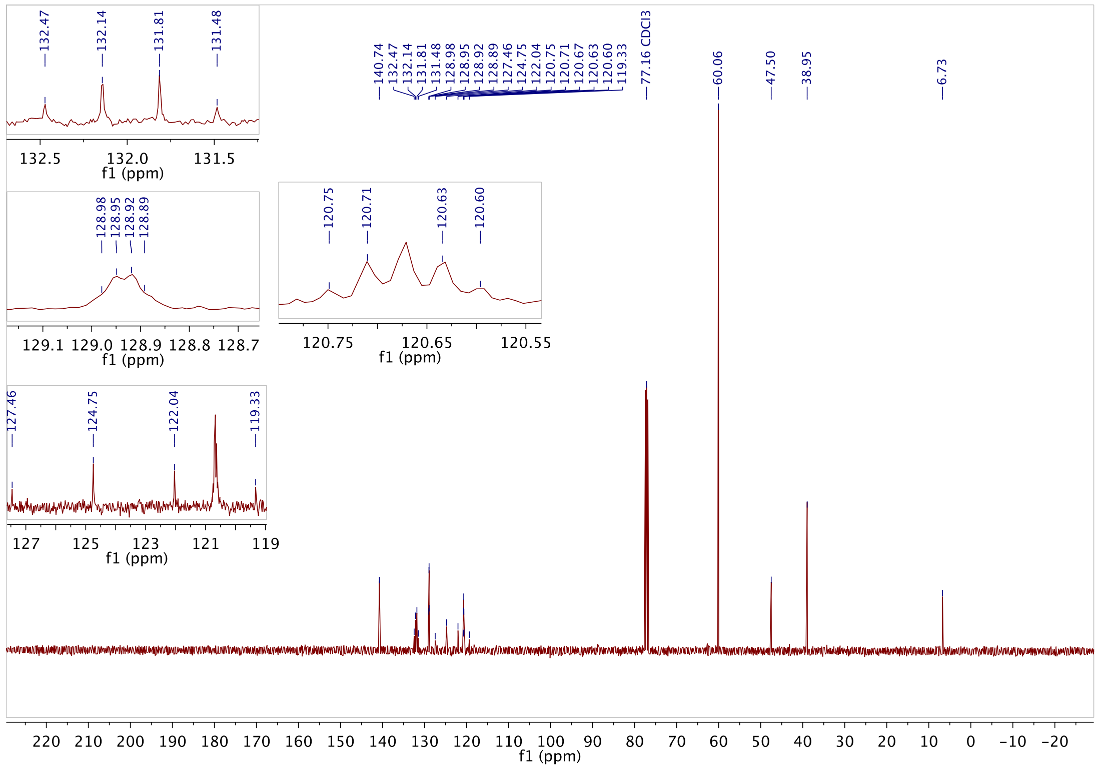

^{19}F NMR (377 MHz, $CDCl_3$)

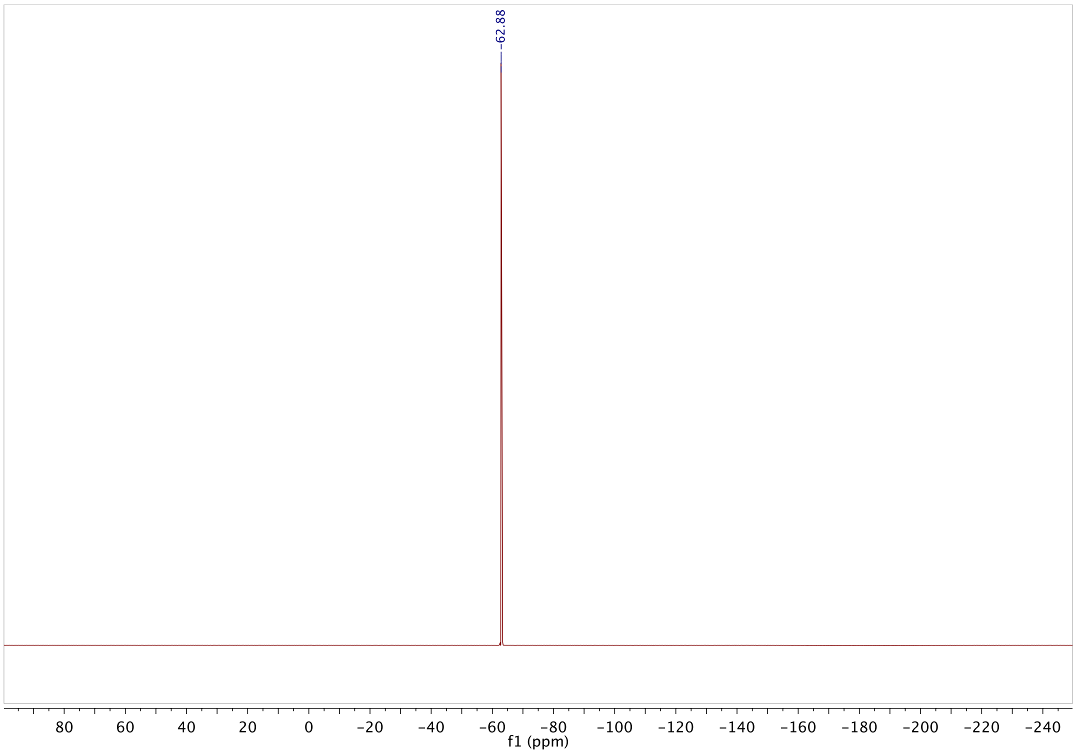

2-((3-Iodobicyclo[1.1.1]pentan-1-yl)methyl)benzonitrile, 5u

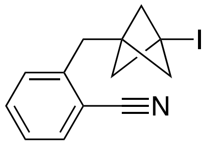

^{1}H NMR (400 MHz, $CDCl_3$)

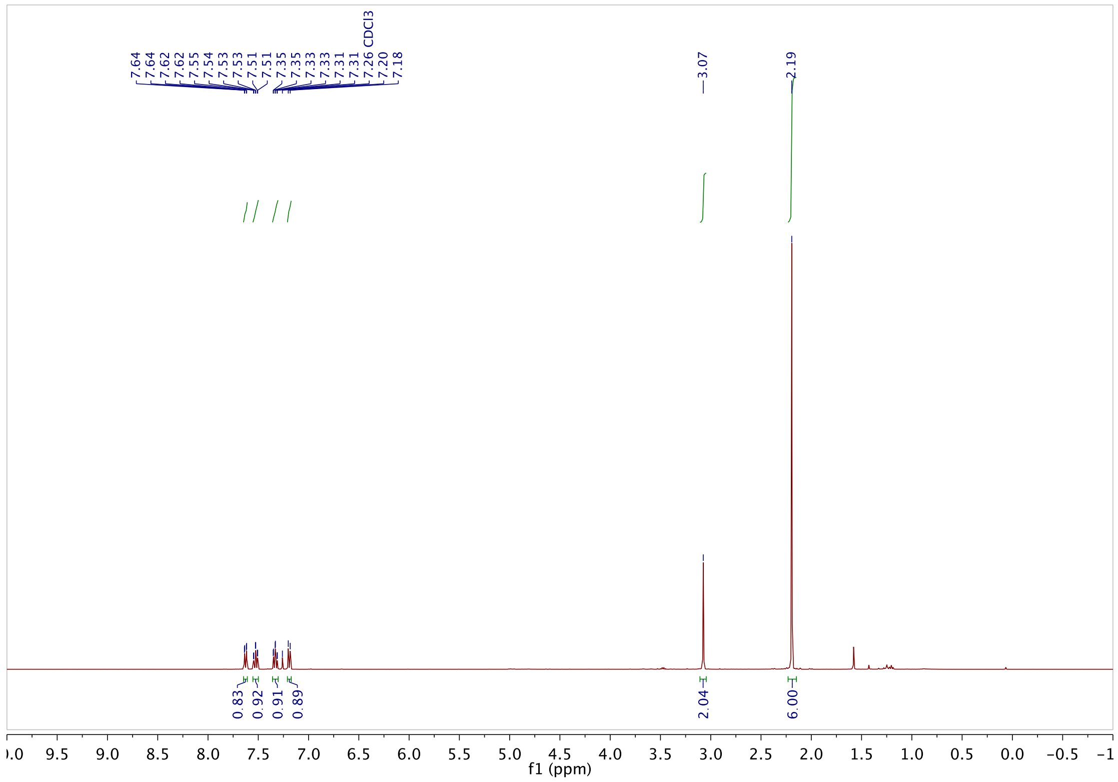

^{13}C NMR (101 MHz, $CDCl_3$)

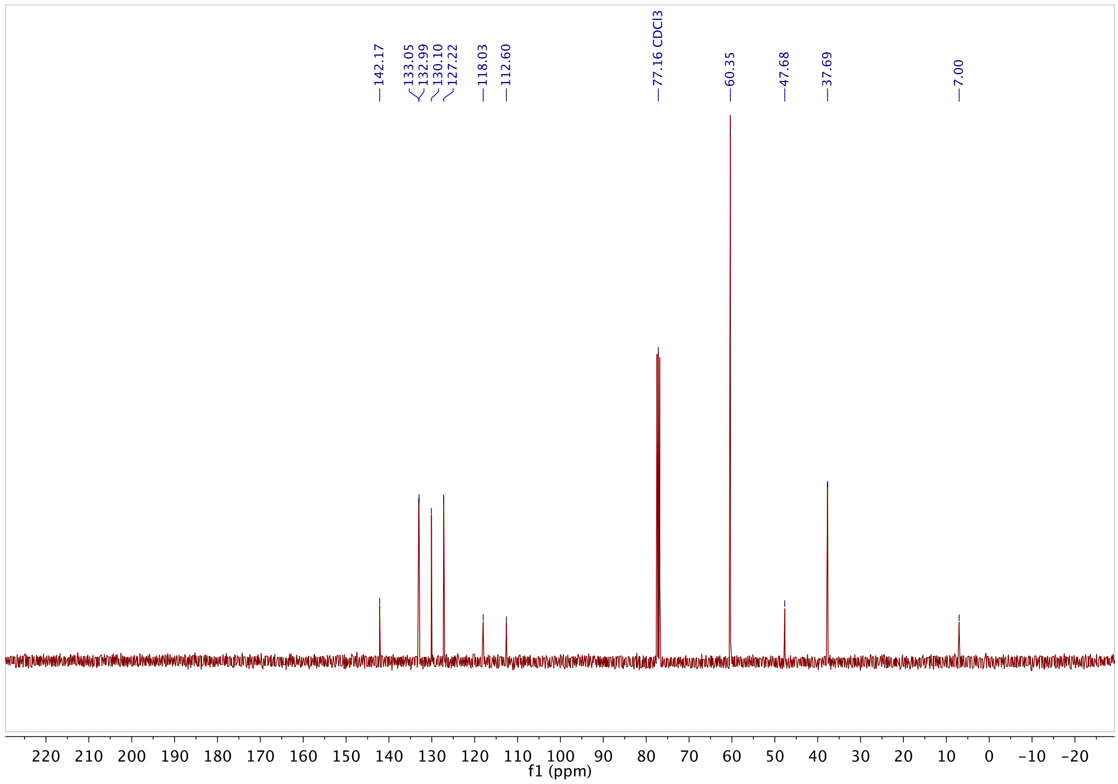

1-(but-3-en-1-yl)-3-iodobicyclo[1.1.1]pentane, 5v

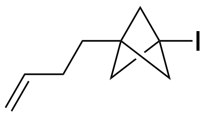

^{1}**H NMR** (400 MHz, $CDCl_3$)

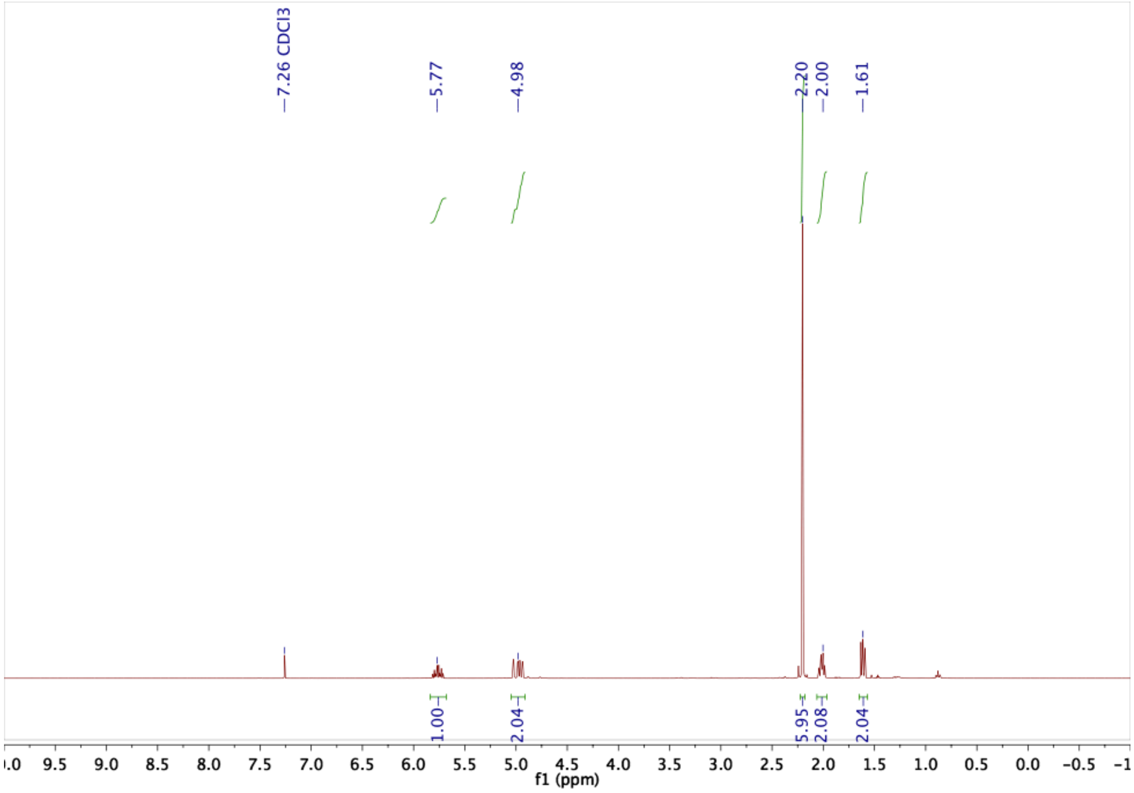

^{13}**C NMR** (101 MHz, $CDCl_3$)

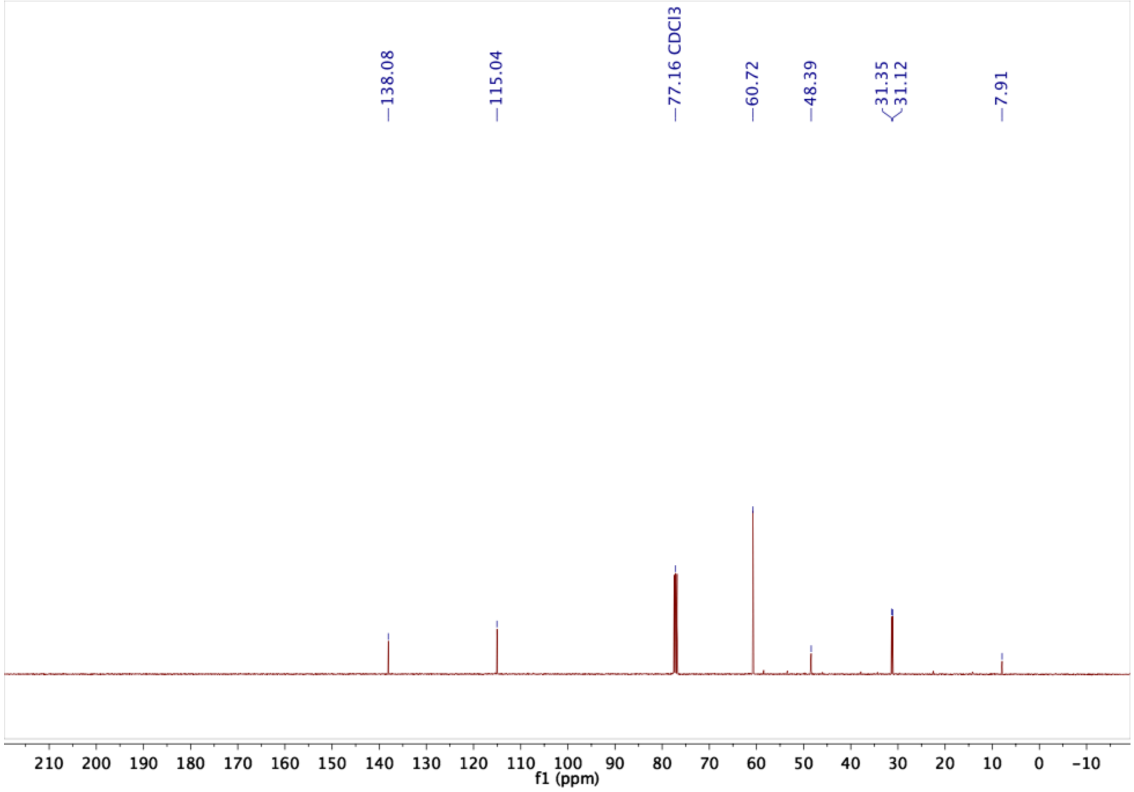

Dimethyl 2-(3-bromobicyclo[1.1.1]pentan-1-yl)malonate, 6c

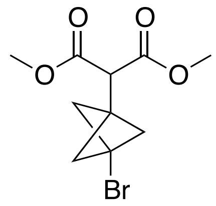

^{1}H NMR (400 MHz, $CDCl_3$)

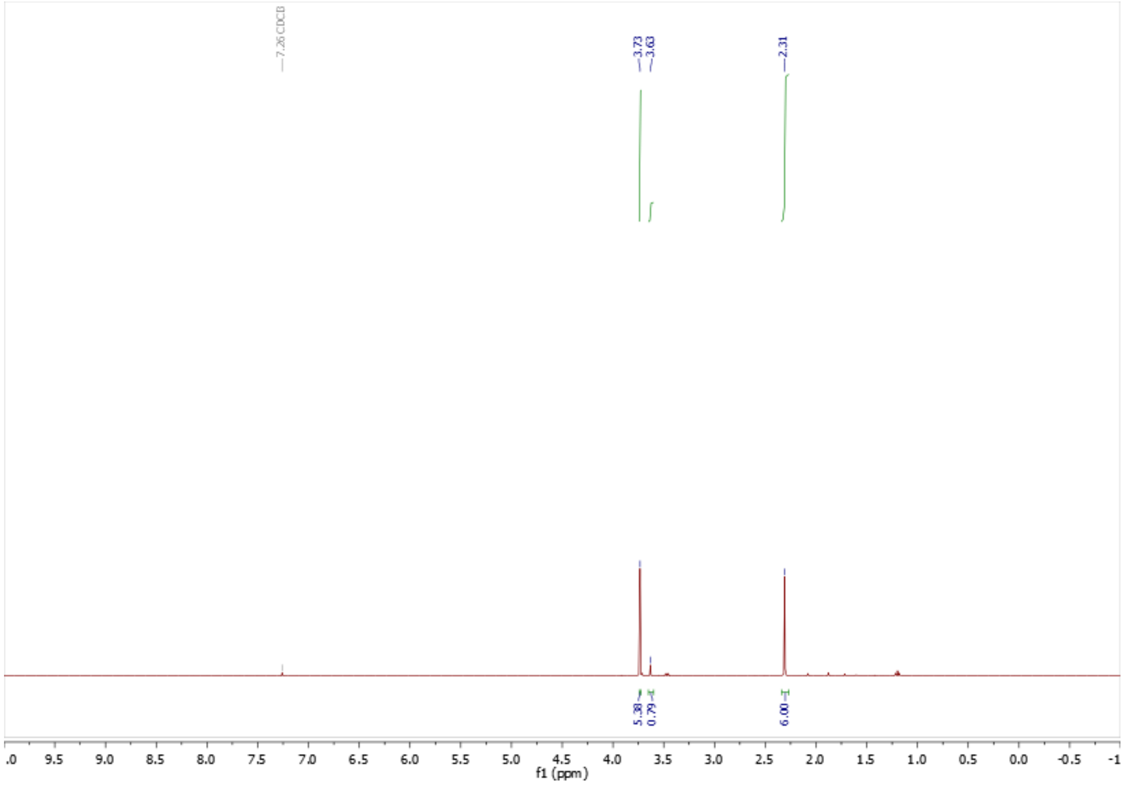

^{13}C NMR (101 MHz, $CDCl_3$)

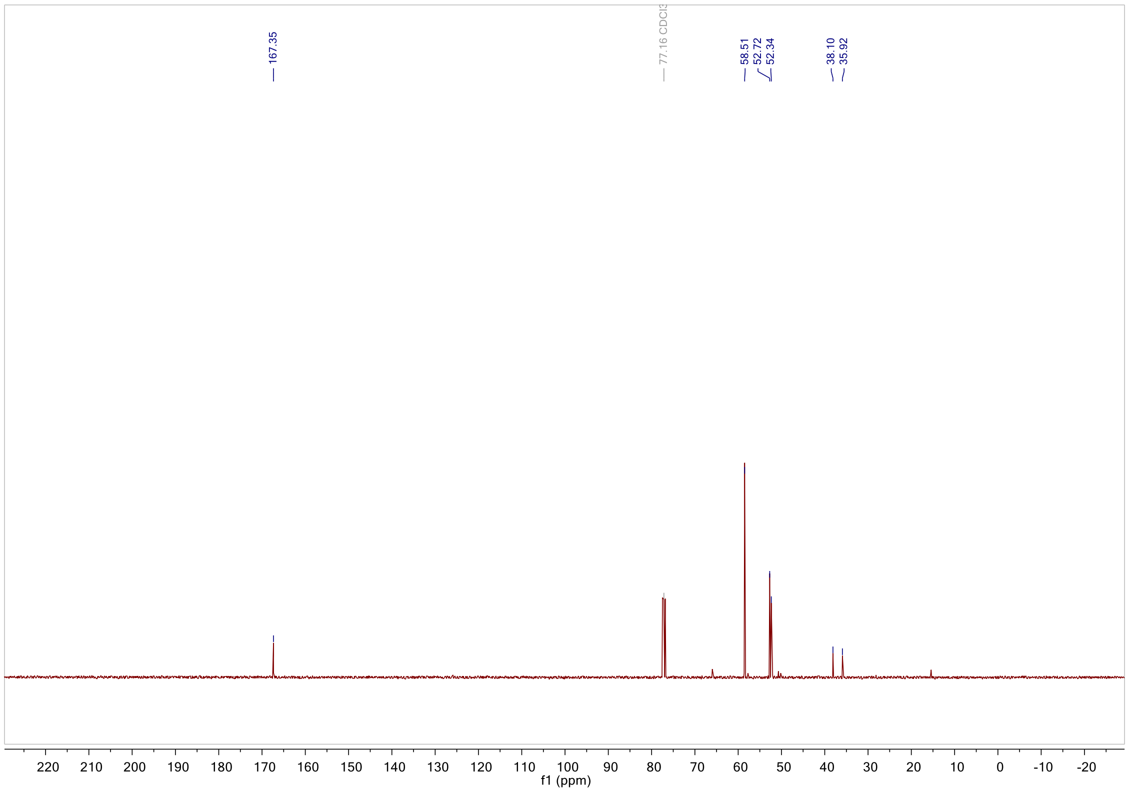

Diethyl 2-(3-bromobicyclo[1.1.1]pentan-1-yl)-2-methylmalonate, 6d

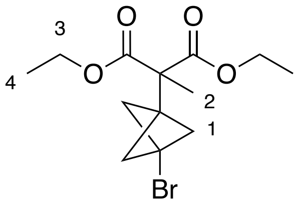

^{1}H NMR (400 MHz, $CDCl_3$)

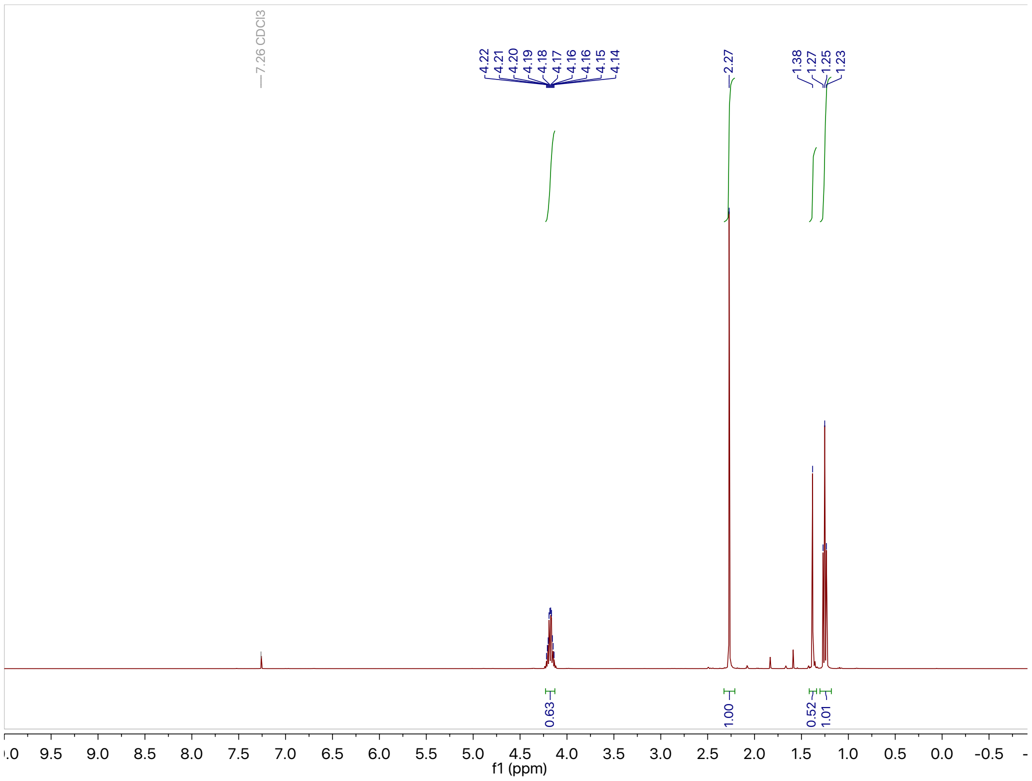

^{13}C NMR (101 MHz, $CDCl_3$)

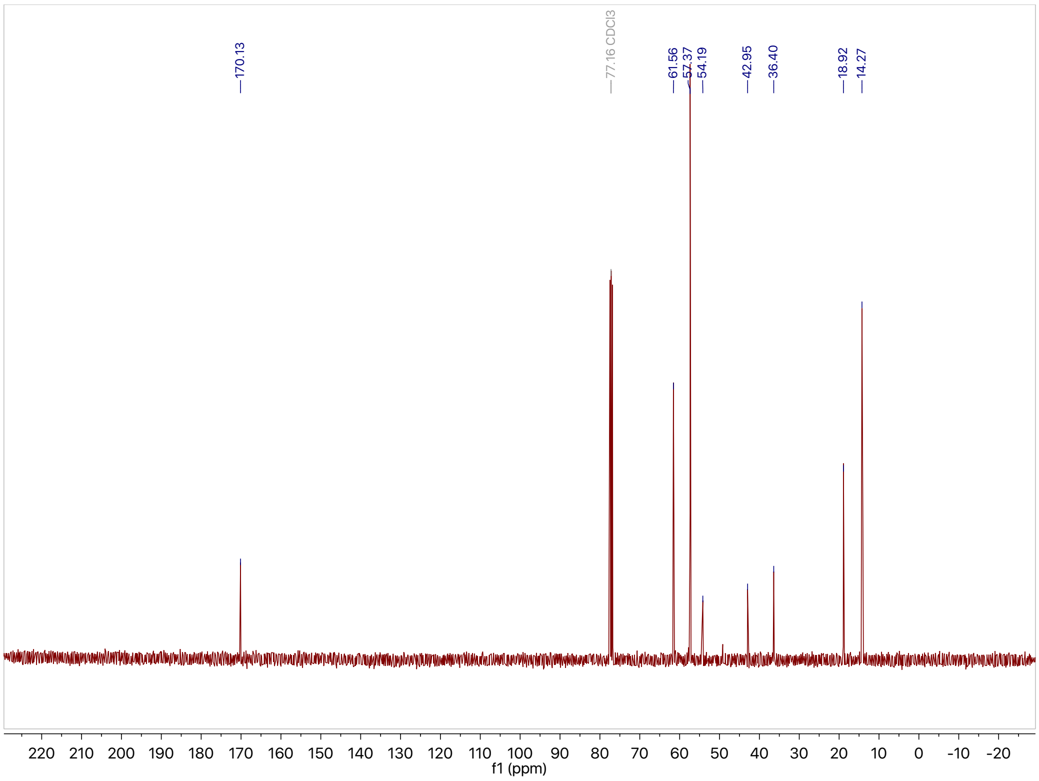

2-(3-Bromobicyclo[1.1.1]pentan-1-yl)-1,3-diphenylpropane-1,3-dione, 6e

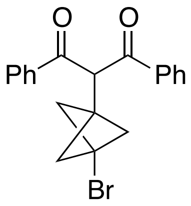

^{1}H NMR (400 MHz, $CDCl_3$)

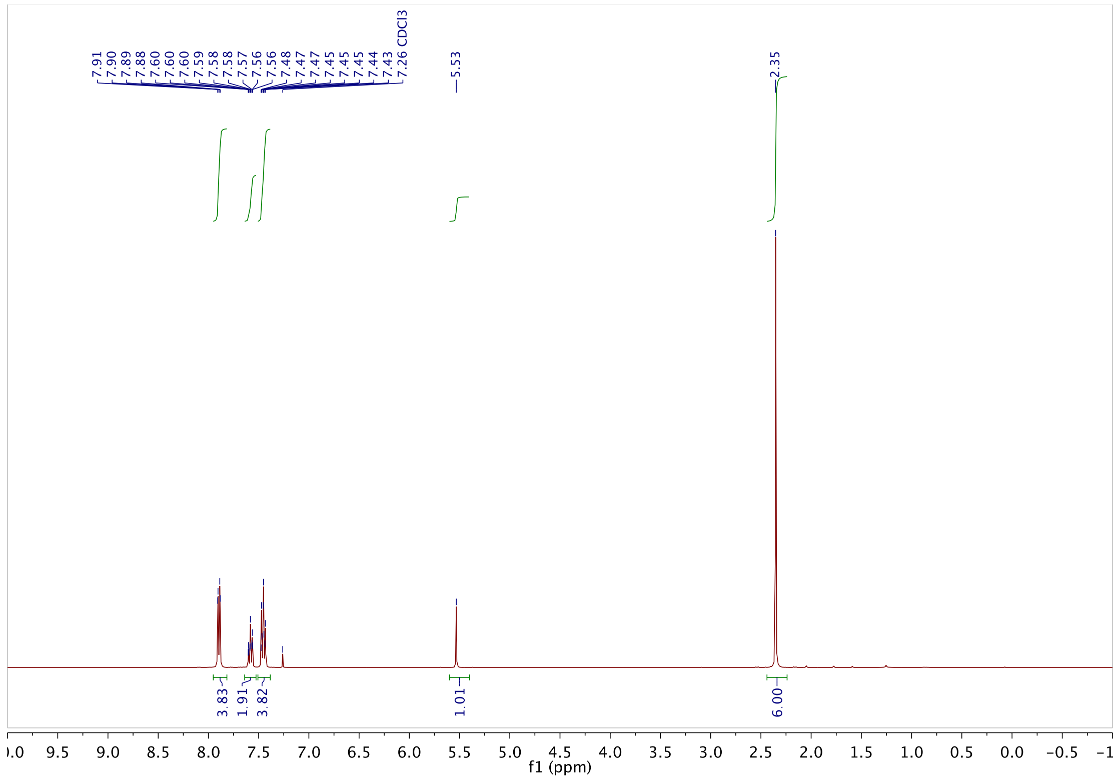

^{13}C NMR (101 MHz, $CDCl_3$)

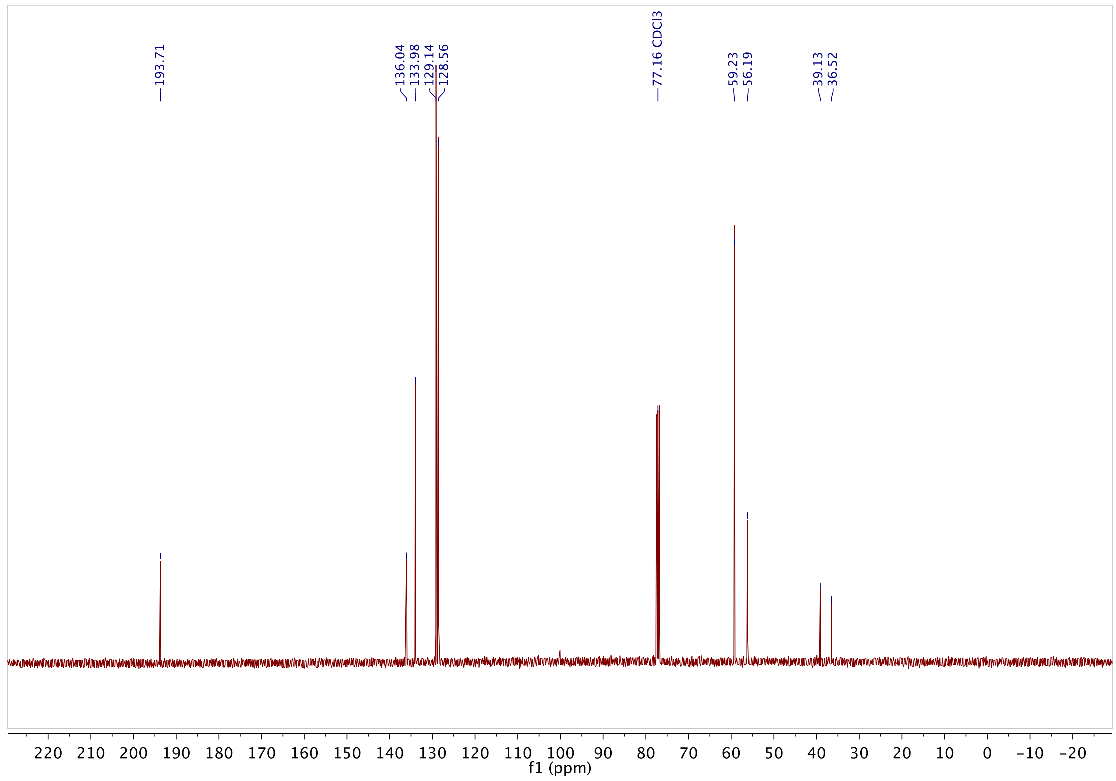

2-(3-Bromobicyclo[1.1.1]pentan-1-yl)-1-phenyl-2-tosylethan-1-one, 6f

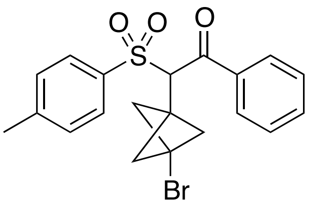

^{1}**H NMR** (400 MHz, $CDCl_3$)

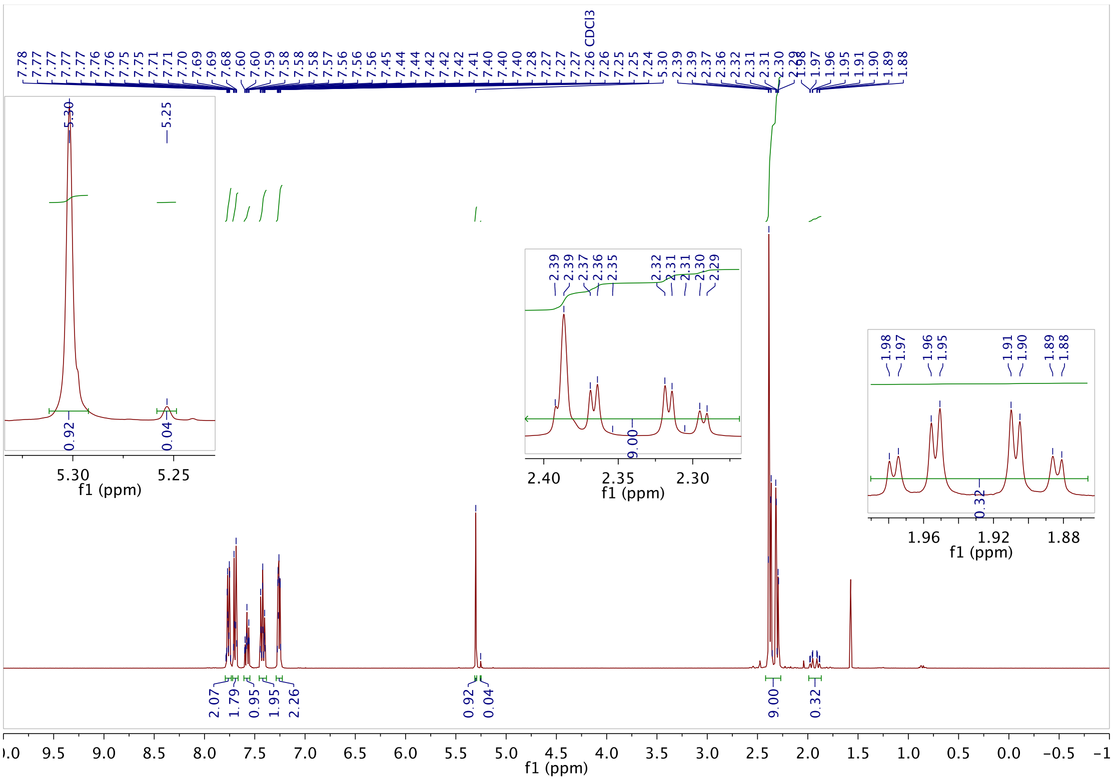

^{13}**C NMR** (101 MHz, $CDCl_3$)

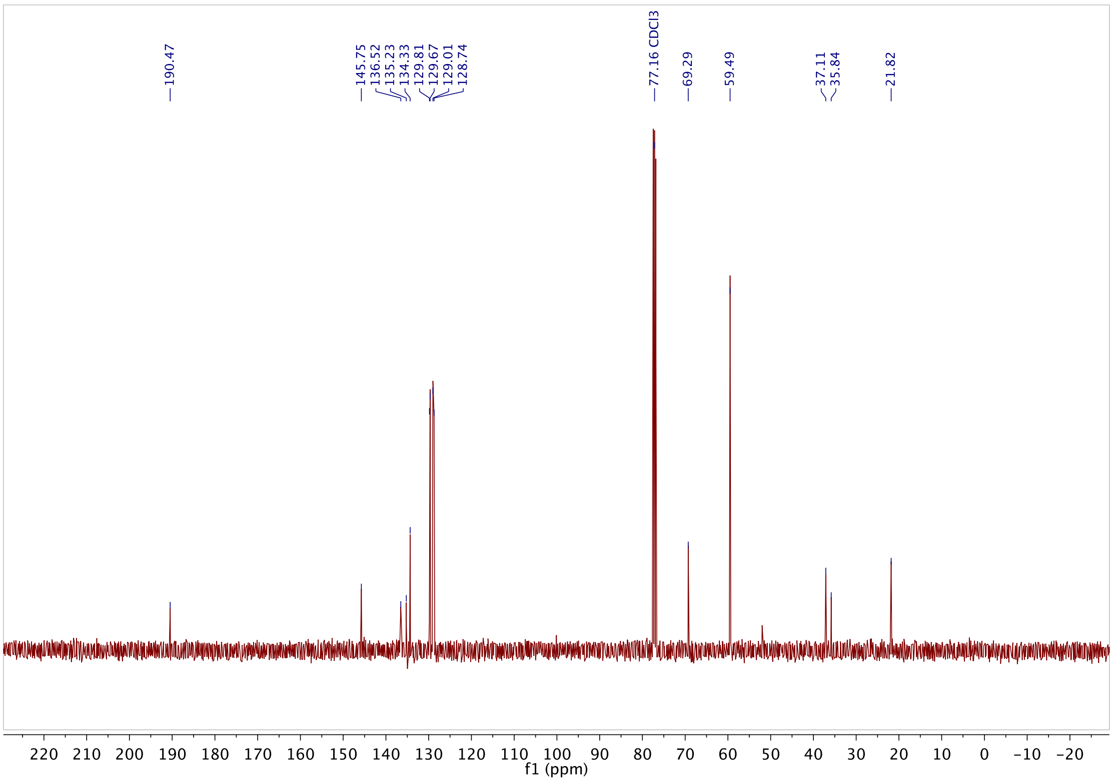

2-(Bicyclo[1.1.1]pentan-1-yl)acetamide, 9l

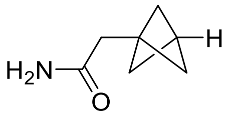

¹H NMR (400 MHz, CDCl₃)

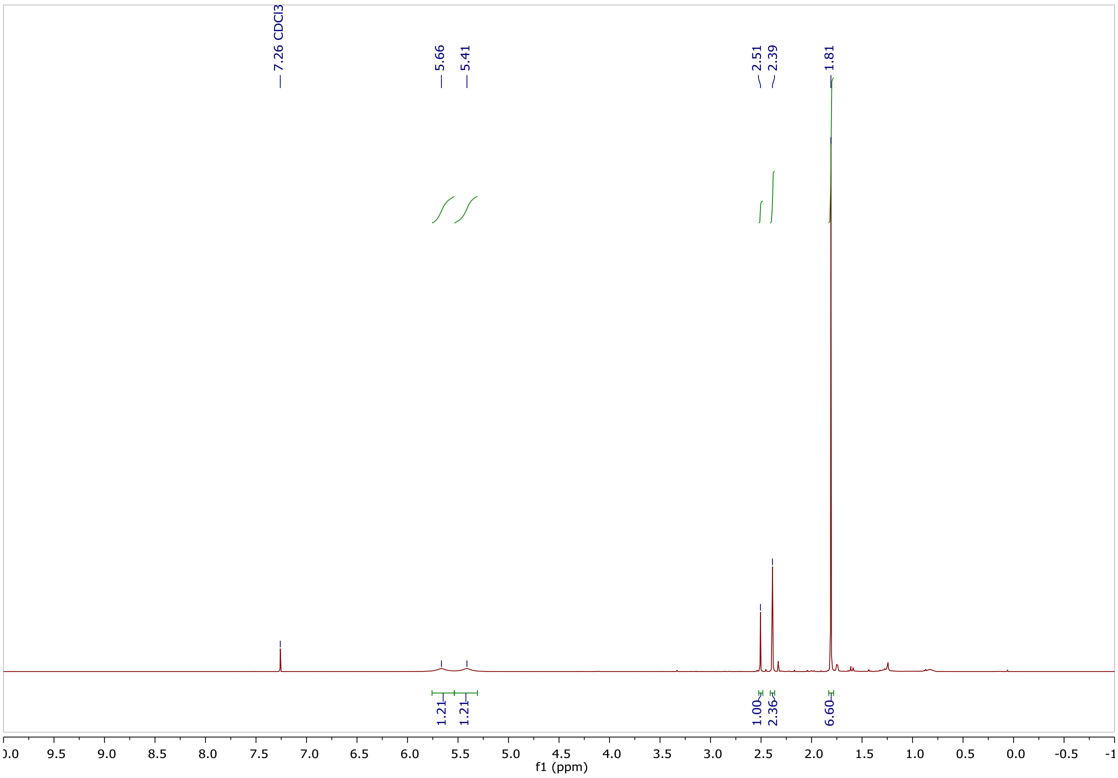

¹³C NMR (101 MHz, CDCl₃)

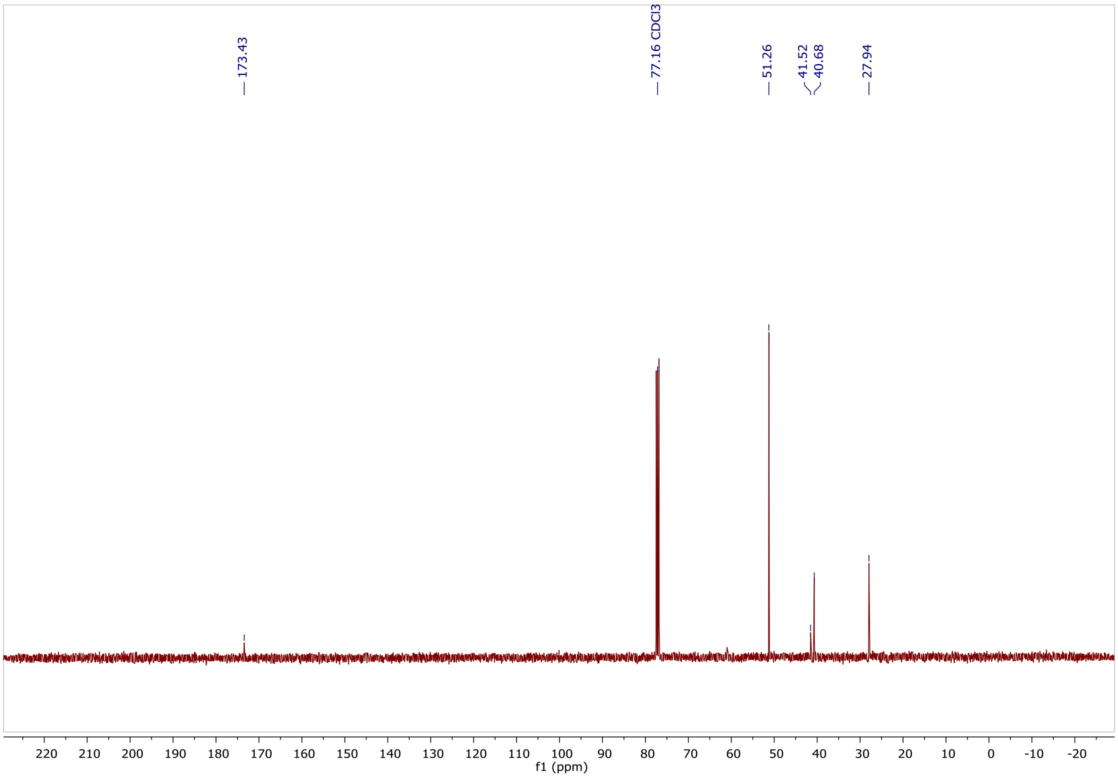

Methyl (*S*)-3-(bicyclo[1.1.1]pentan-1-yl)-2-((tert-butoxycarbonyl)amino)propanoate, 9p

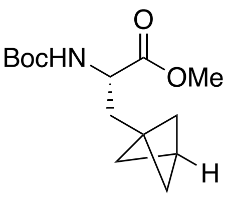

^{1}H NMR (400 MHz, $CDCl_3$)

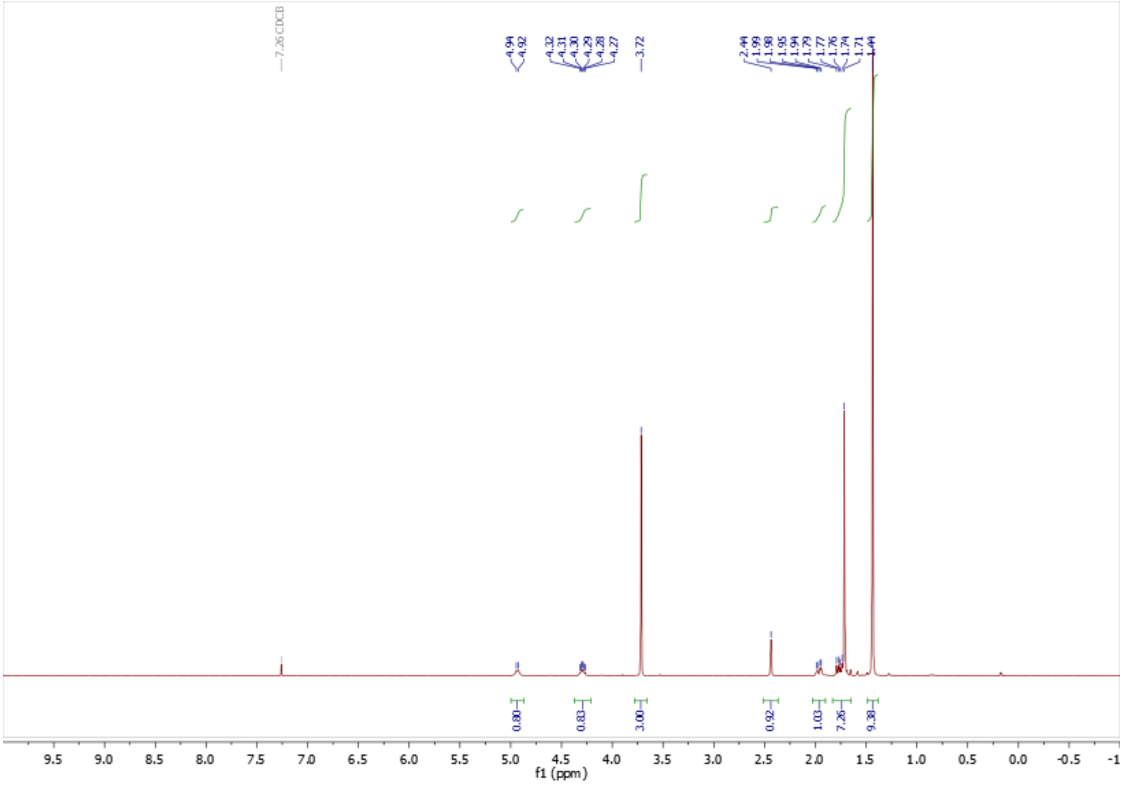

^{13}C NMR (101 MHz, $CDCl_3$)

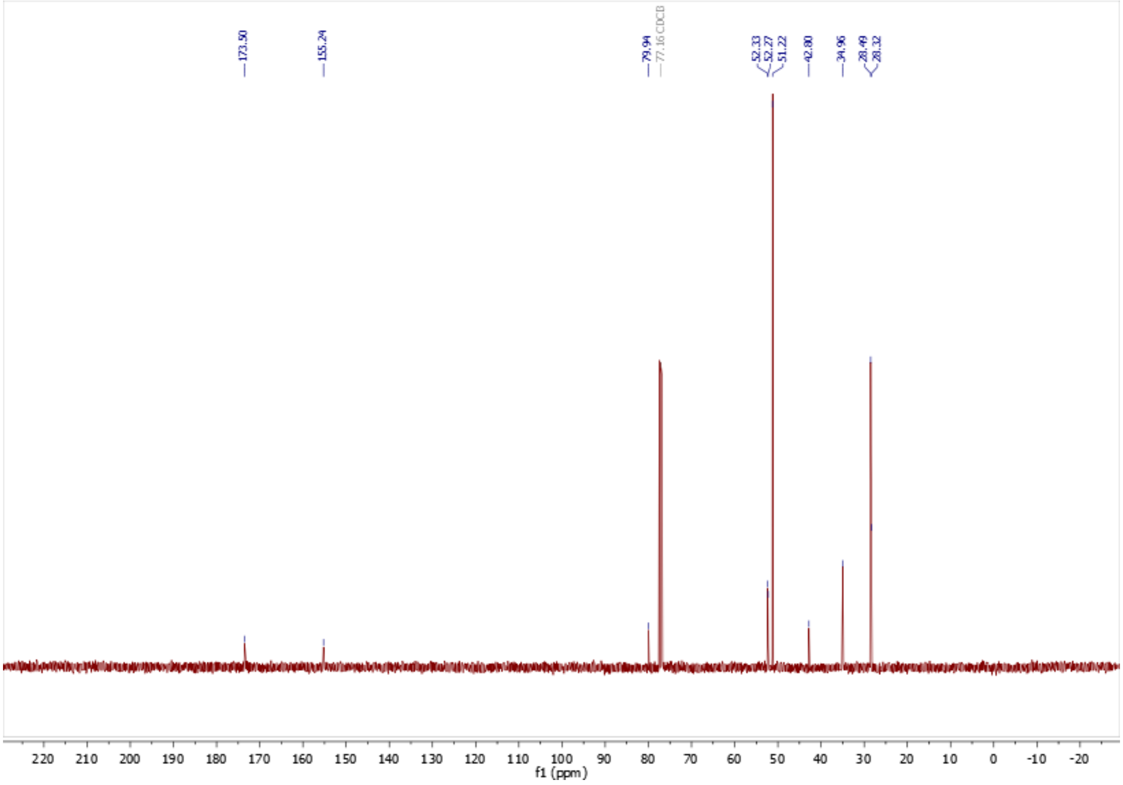

2-(Bicyclo[1.1.1]pentan-1-yl)ethan-1-aminium 2,2,2-trifluoroacetate, 9e

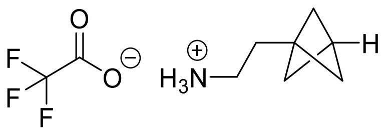

^{1}H NMR (400 MHz, CD_3OD)

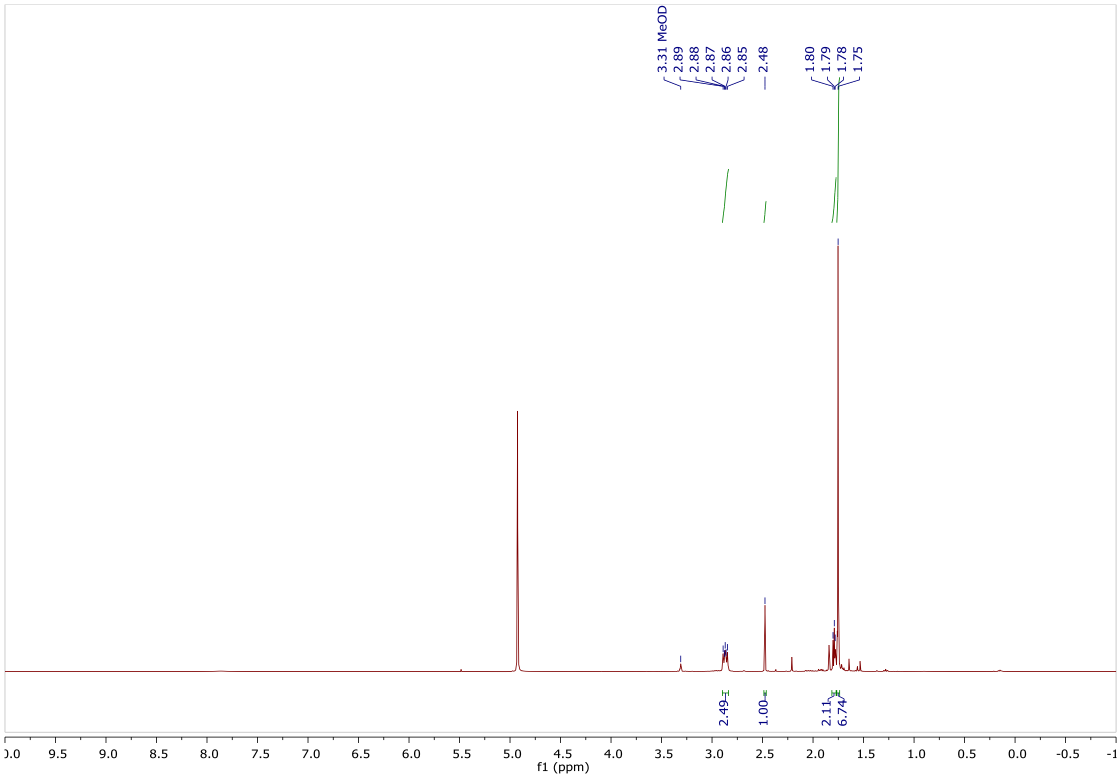

^{13}C NMR (101 MHz, CD_3OD)

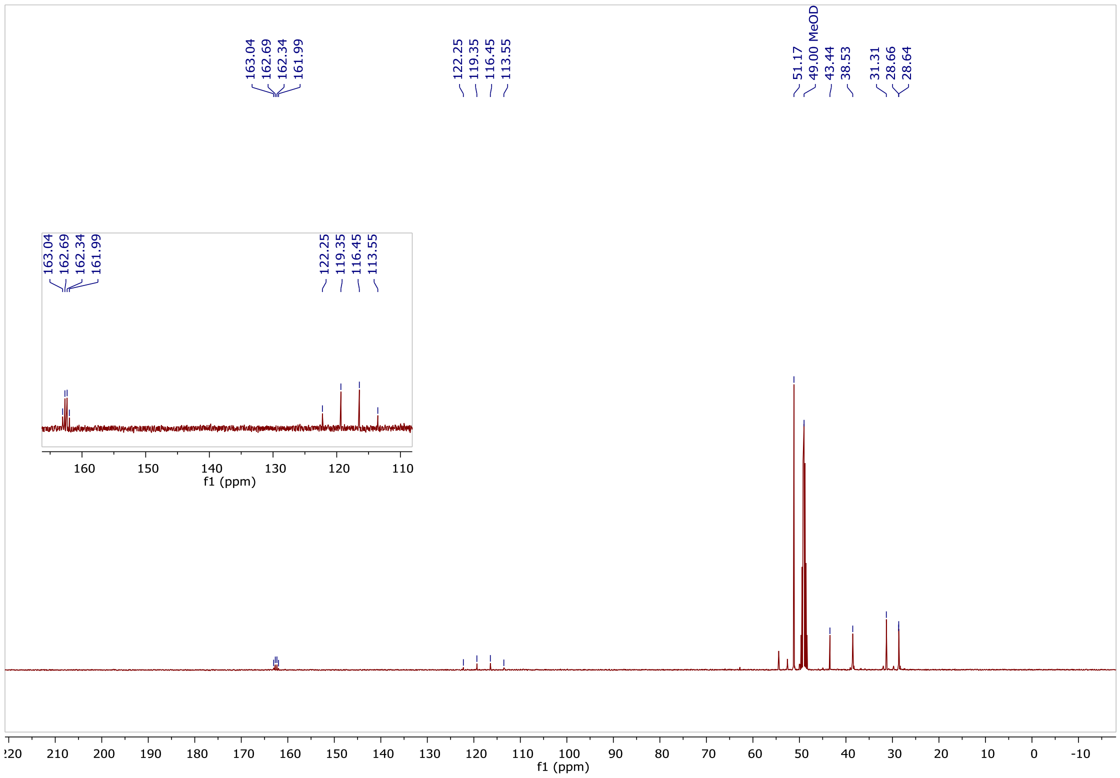

^{19}**F NMR** (376 MHz, CD_3OD)

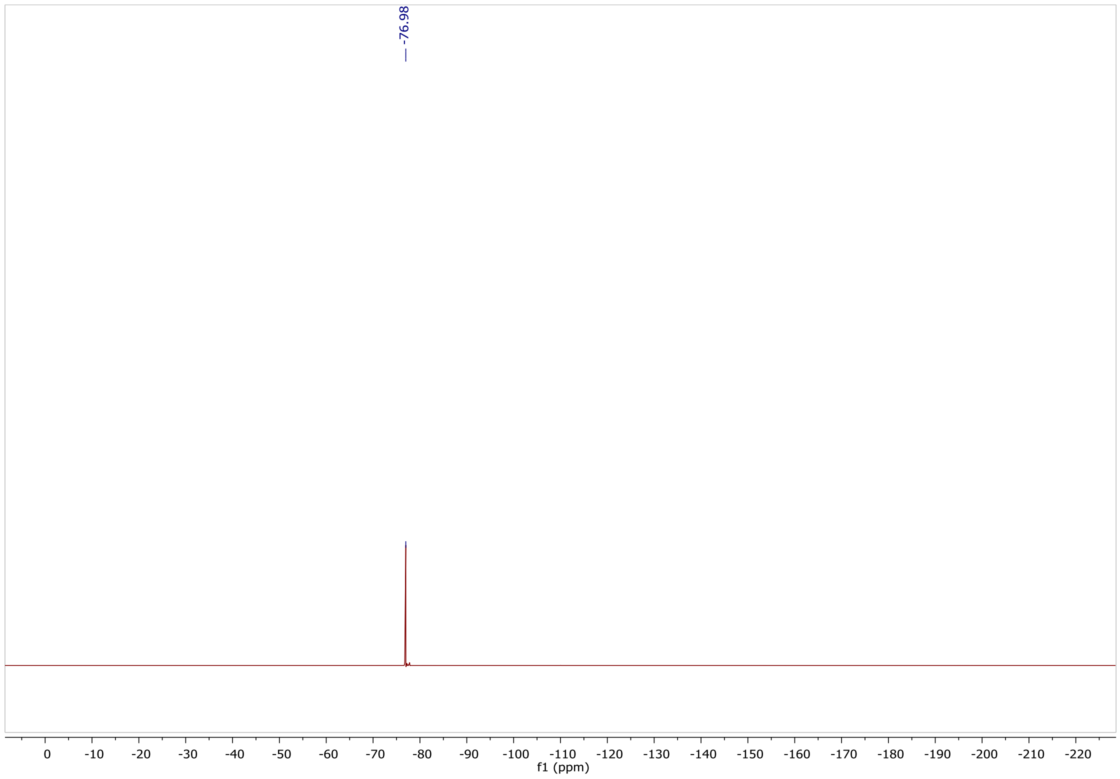

2-(Bicyclo[1.1.1]pentan-1-yl)acetic acid, 9a

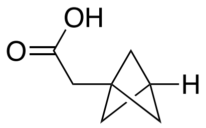

^{1}H NMR (400 MHz, $CDCl_3$)

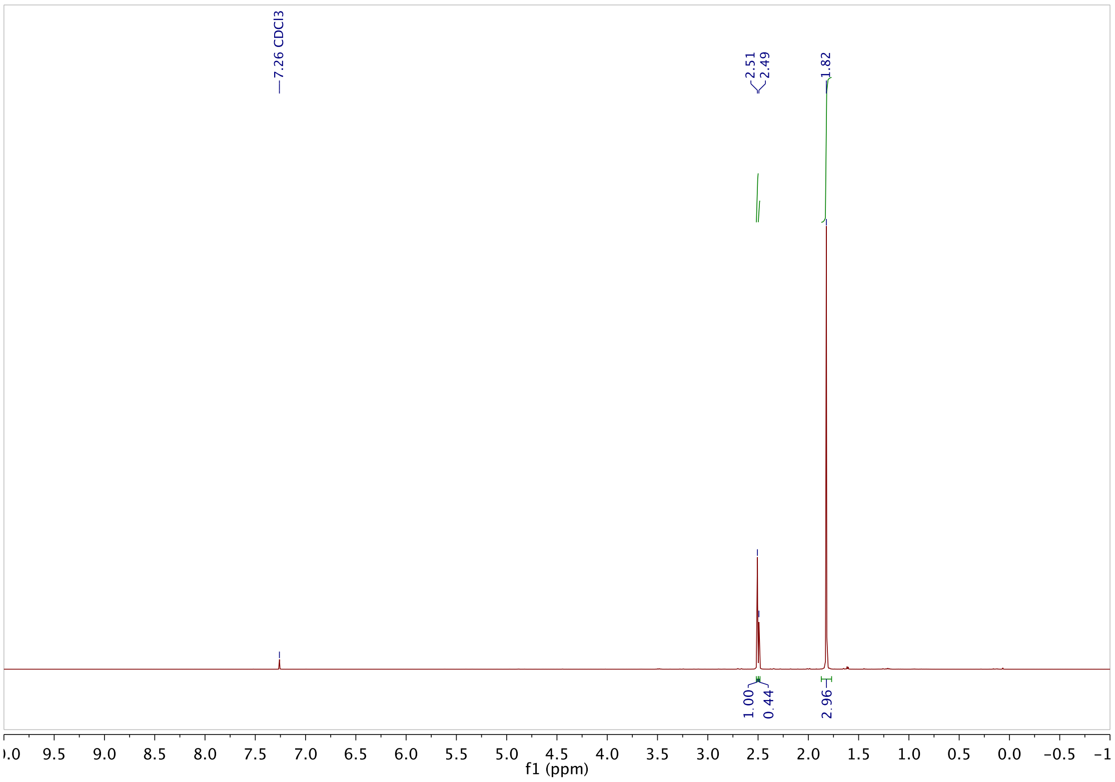

^{13}C NMR (101 MHz, $CDCl_3$)

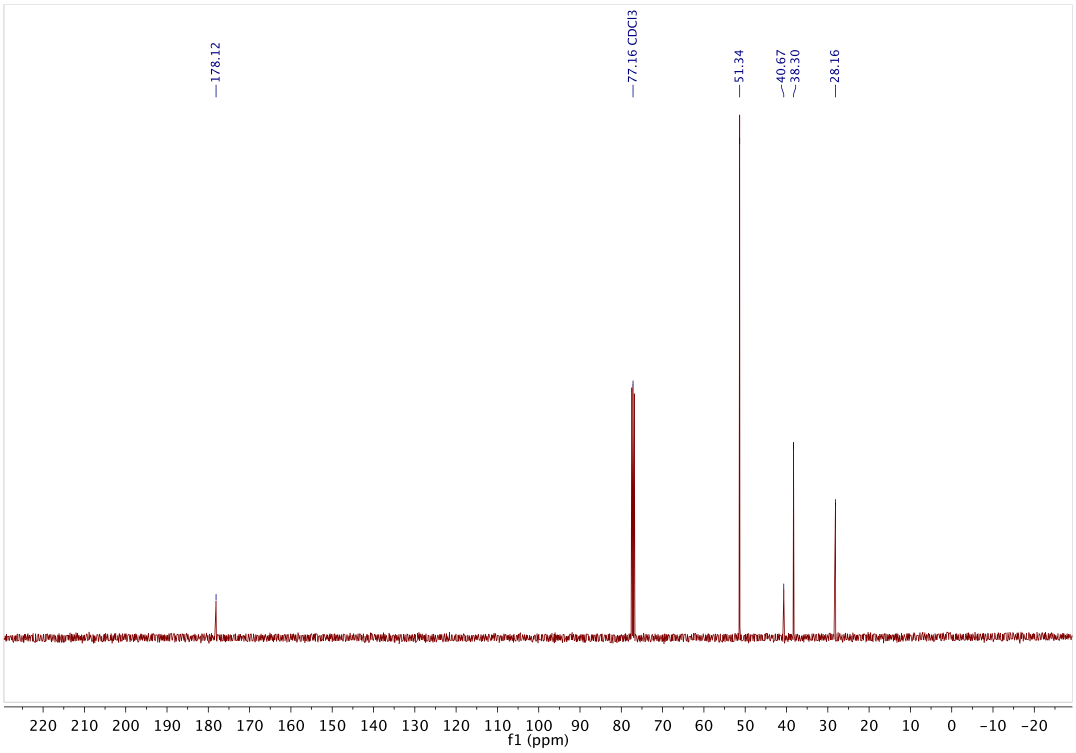

Phenyl(3-(4-(trifluoromethyl)benzyl)bicyclo[1.1.1]pentan-1-yl)methanol, 11

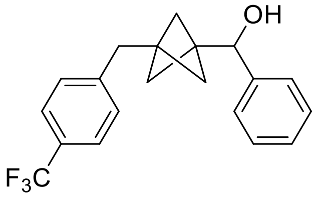

^{1}H NMR (500 MHz, $CDCl_3$)

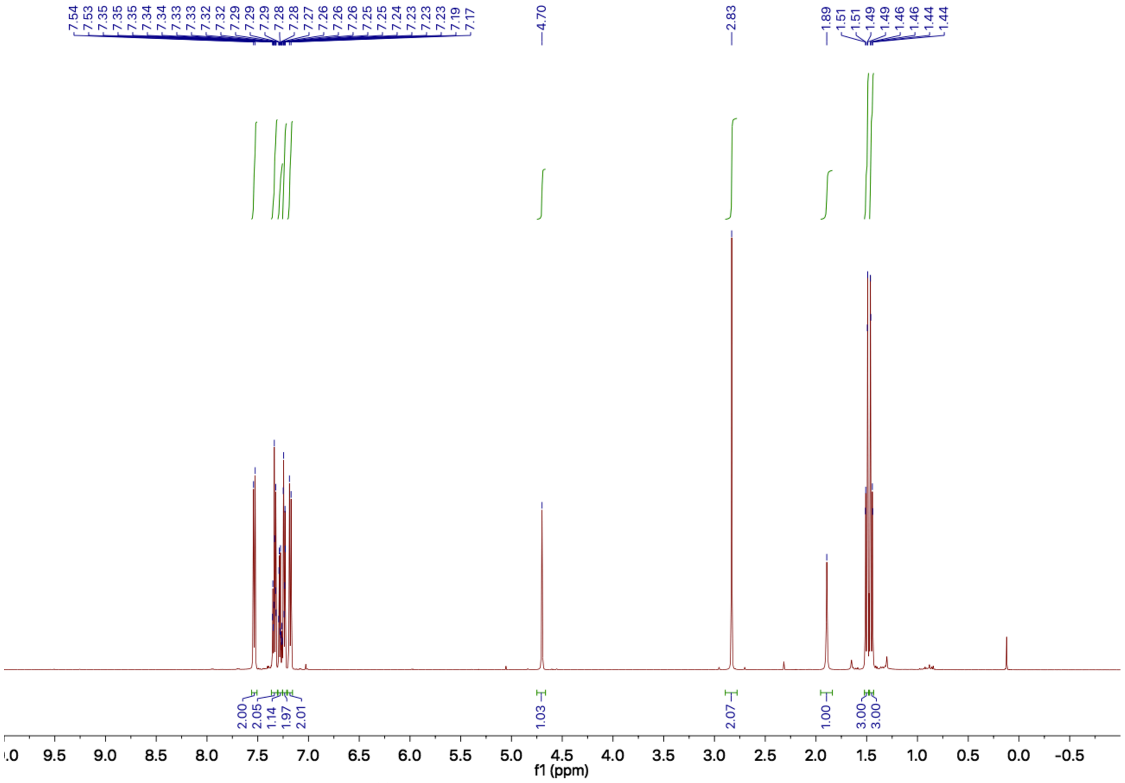

^{13}C NMR ($CDCl_3$, 126 MHz)

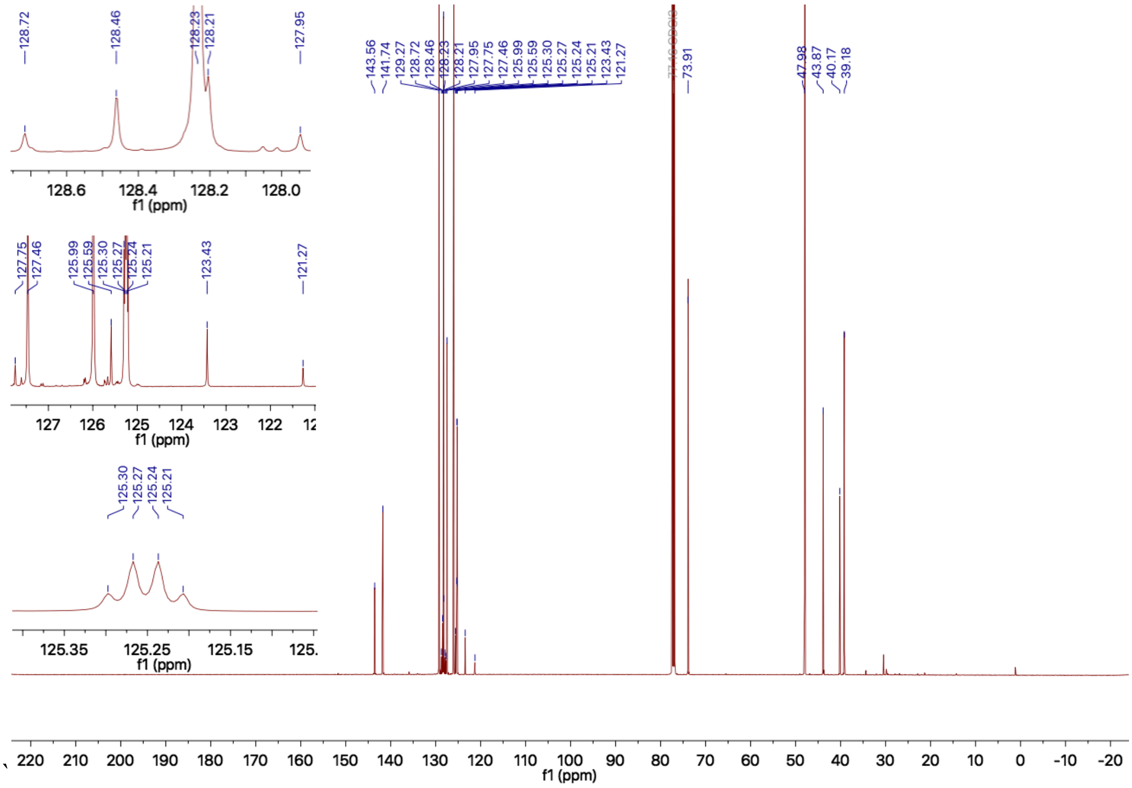

^{19}F NMR ($CDCl_3$, 377 MHz)

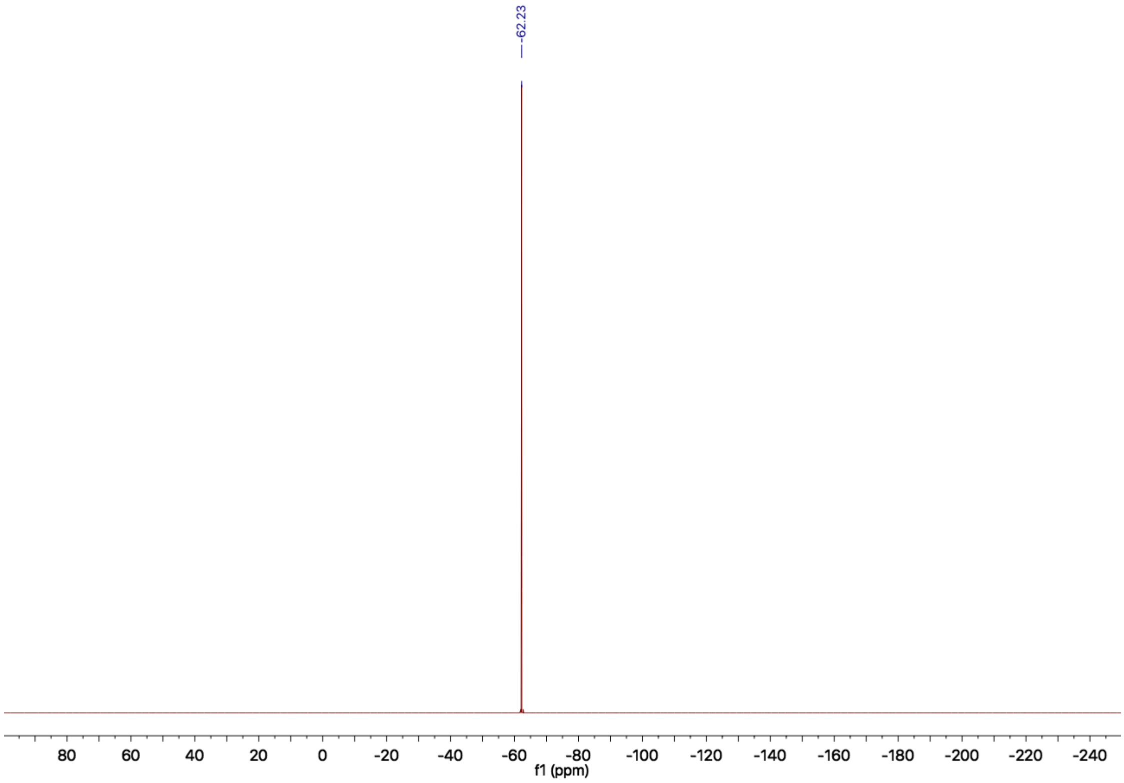

3-(4-(Trifluoromethyl)benzyl)bicyclo[1.1.1]pentane-1-carbaldehyde, 12

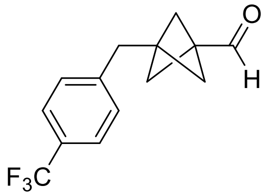

^{1}H NMR (500 MHz, $CDCl_3$)

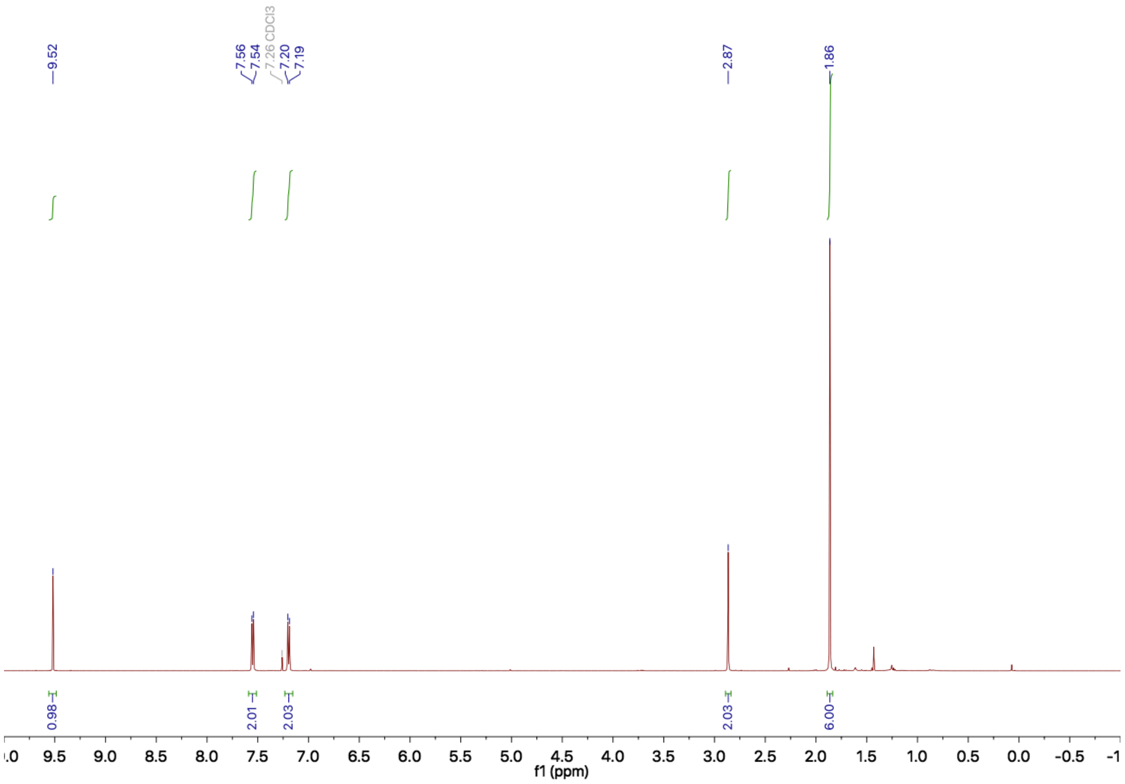

^{13}C NMR ($CDCl_3$, 126 MHz)

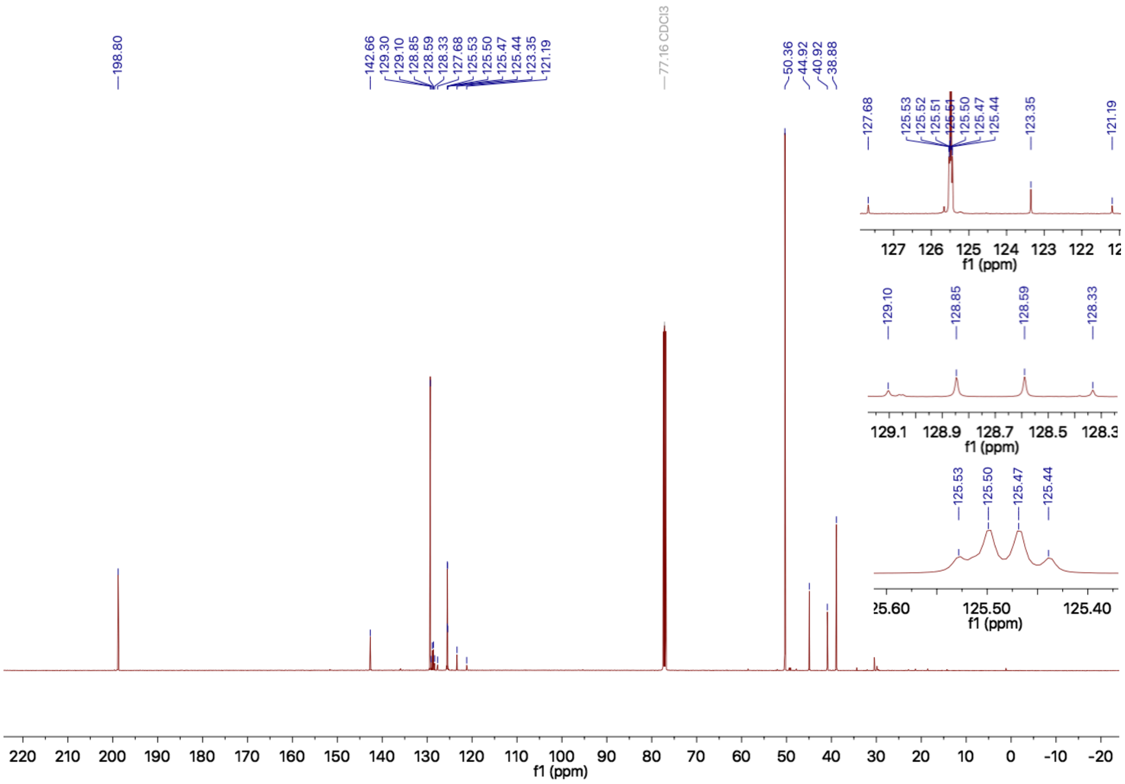

^{19}F NMR ($CDCl_3$, 377 MHz)

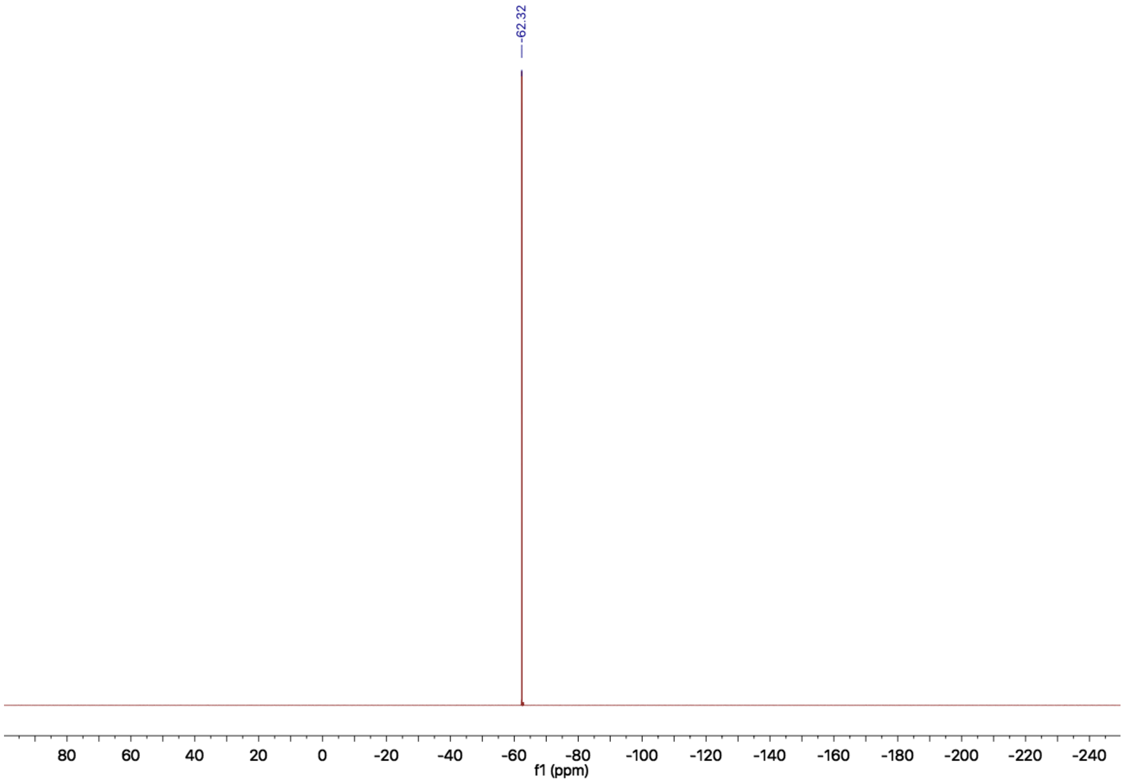

2-(3-(4-(Trifluoromethyl)benzyl)bicyclo[1.1.1]pentan-1-yl)pyridine, 13

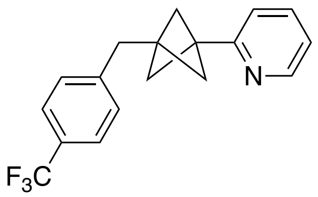

^{1}H NMR ($CDCl_3$, 400 MHz)

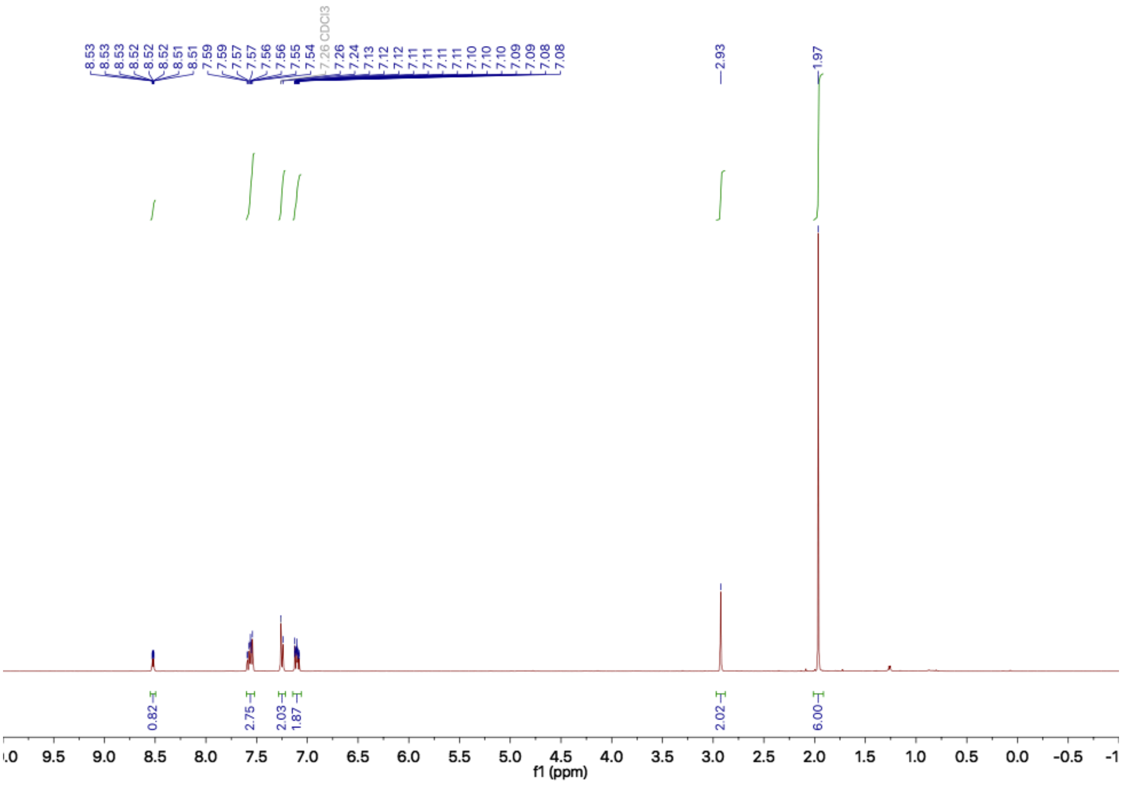

^{13}C NMR ($CDCl_3$, 126 MHz)

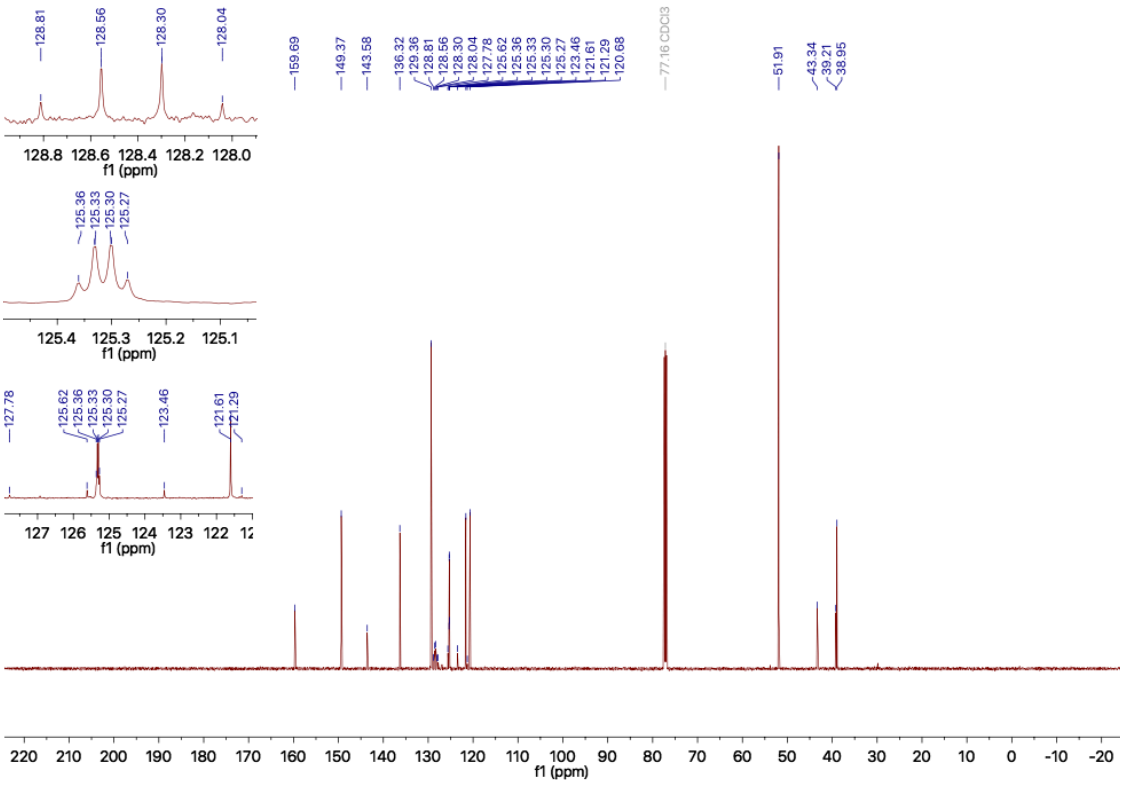

^{19}F NMR ($CDCl_3$, 377 MHz)

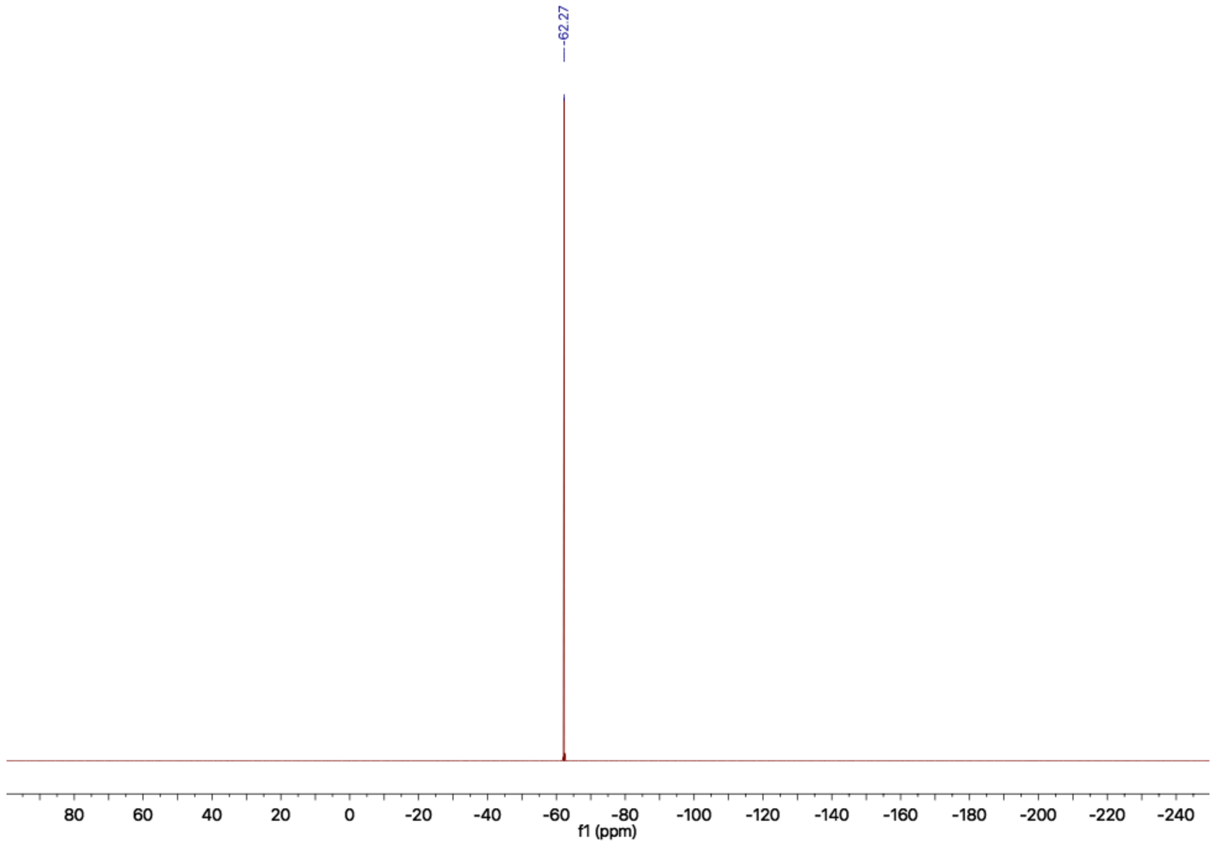

3-(4-(trifluoromethyl)benzyl)bicyclo[1.1.1]pentan-1-ol, 14

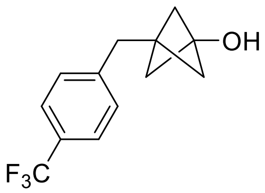

^{1}H NMR ($CDCl_3$, 400 MHz)

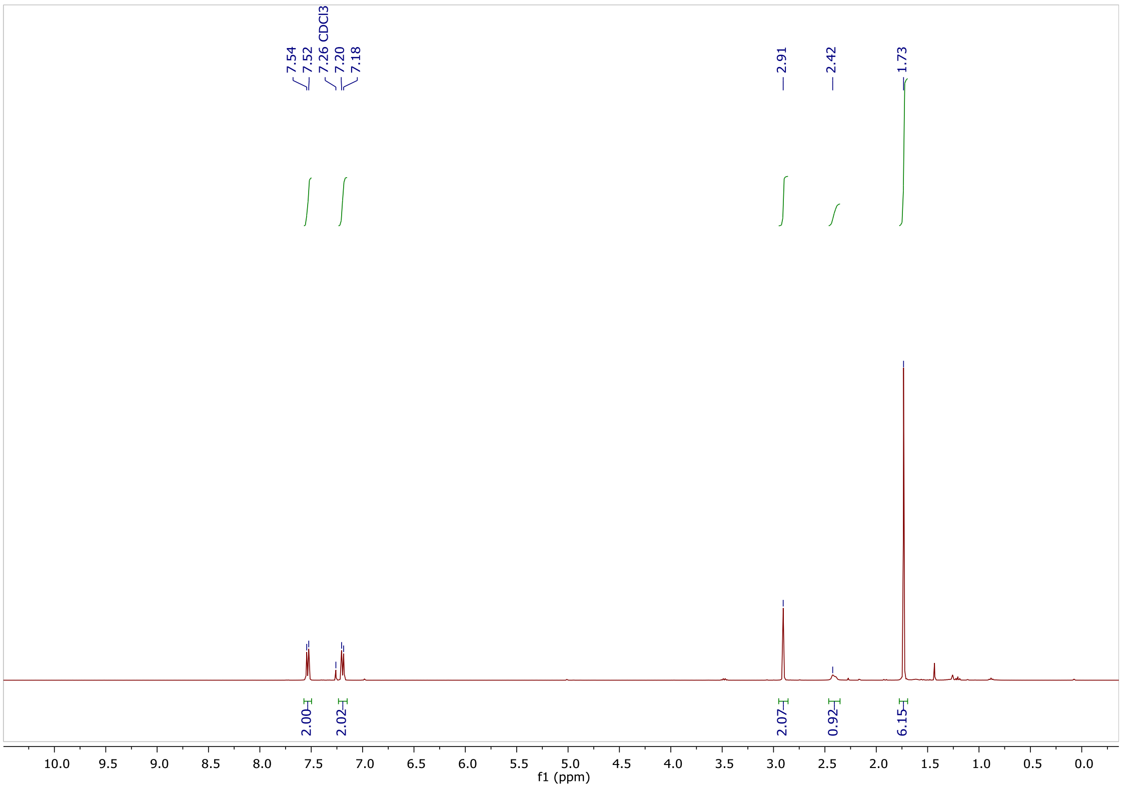

^{13}C NMR ($CDCl_3$, 101 MHz)

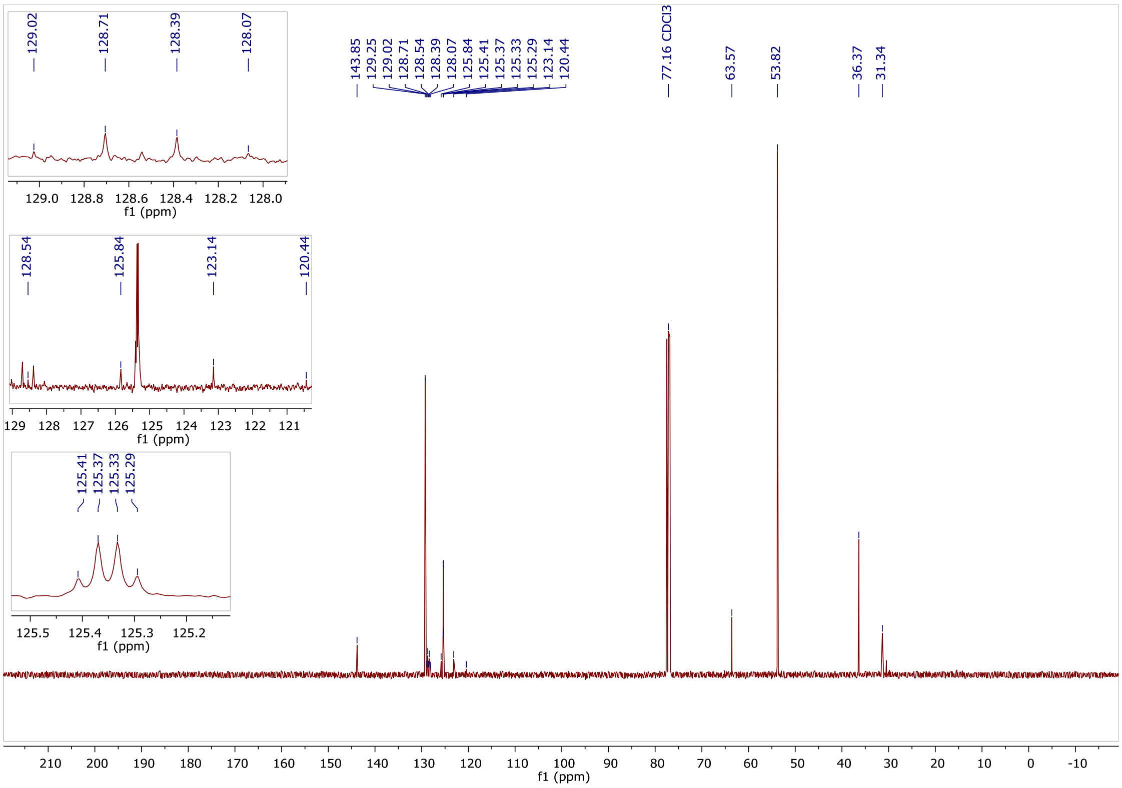

^{19}F NMR (CDCl$_3$, 377 MHz)

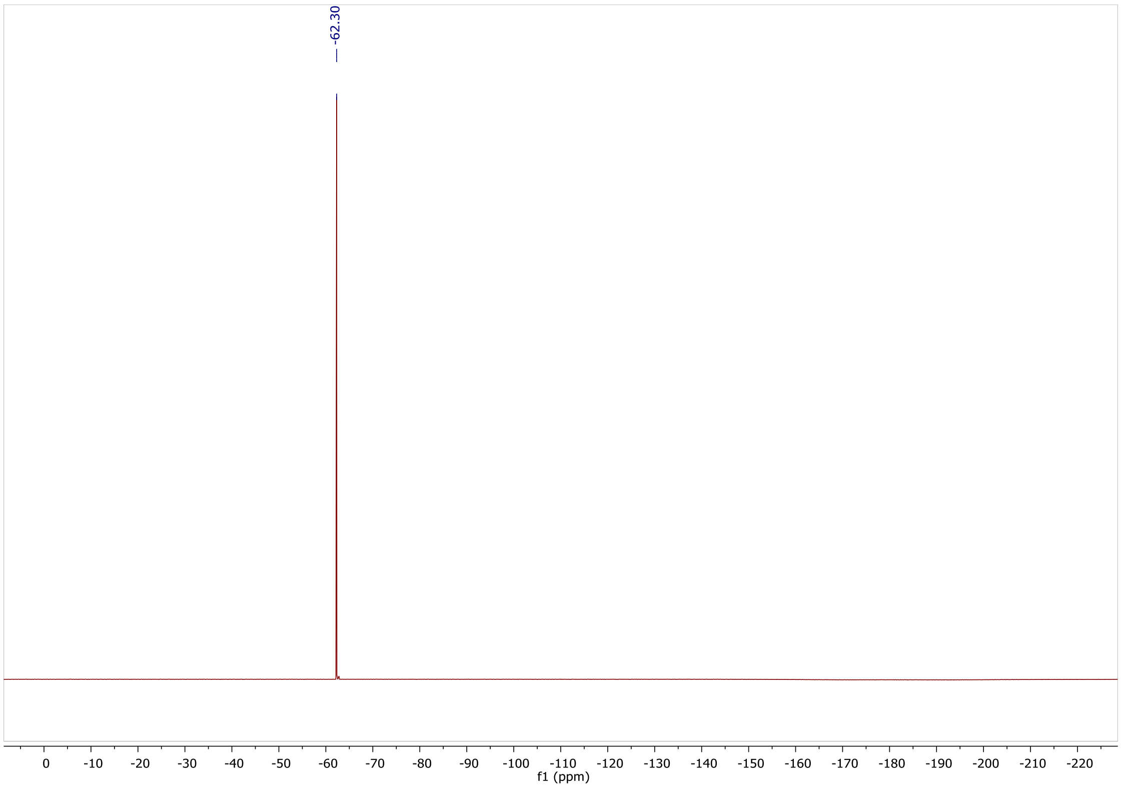

1-((2*R*,3*R*,4*S*,5*R*)-4-((*tert*-butyldimethylsilyl)oxy)-5-(hydroxymethyl)-3-(3-iodobicyclo[1.1.1]pentan-1-yl)tetrahydrofuran-2-yl)pyrimidine-2,4(1*H*,3*H*)-dione, 5w

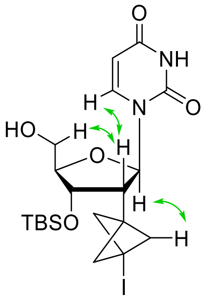

(nOe enhancements indicated in green)

^{1}H NMR (400 MHz, MeOD)

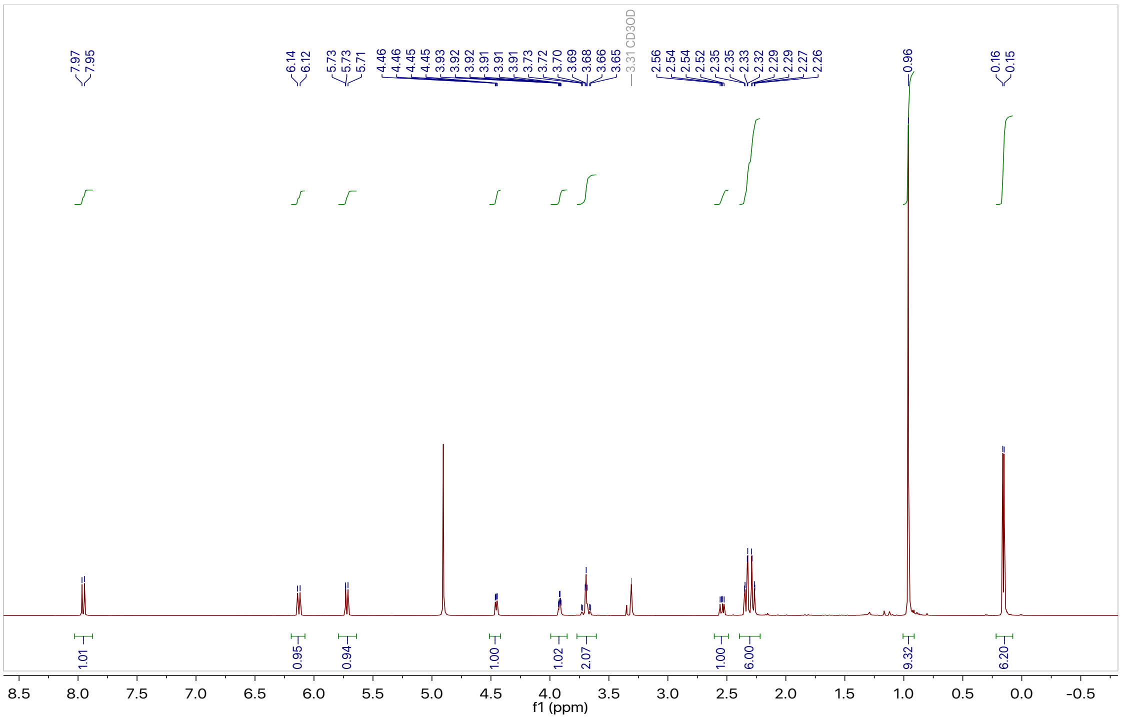

^{13}C NMR (101 MHz, MeOD)

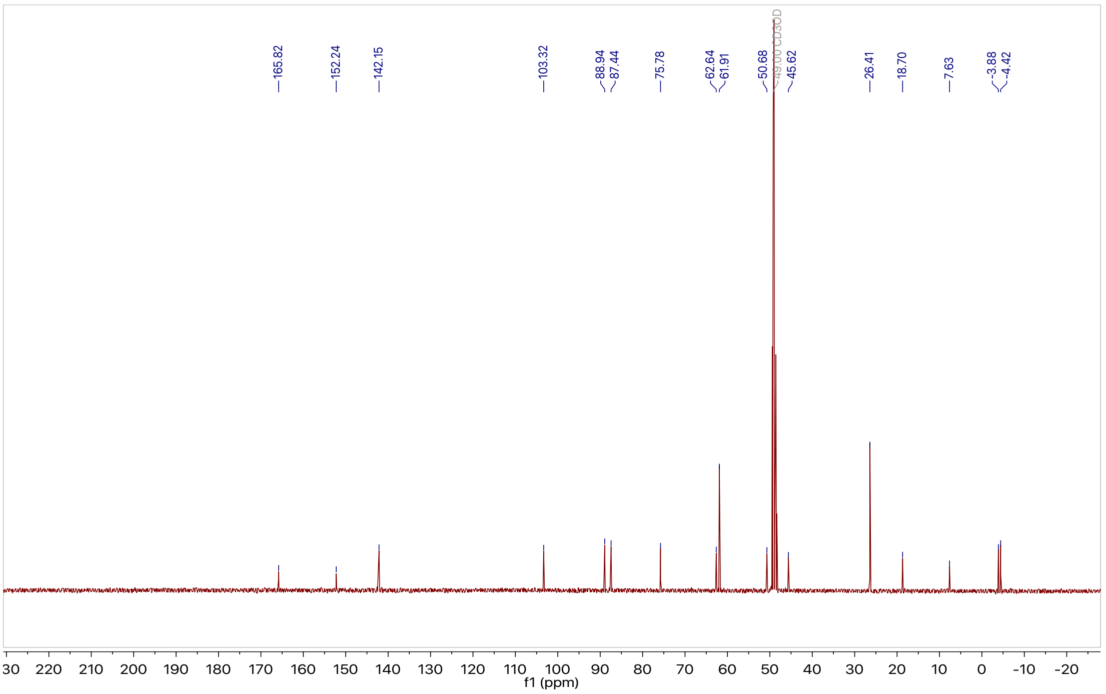

NOESY (400 MHz, CD_3OD)

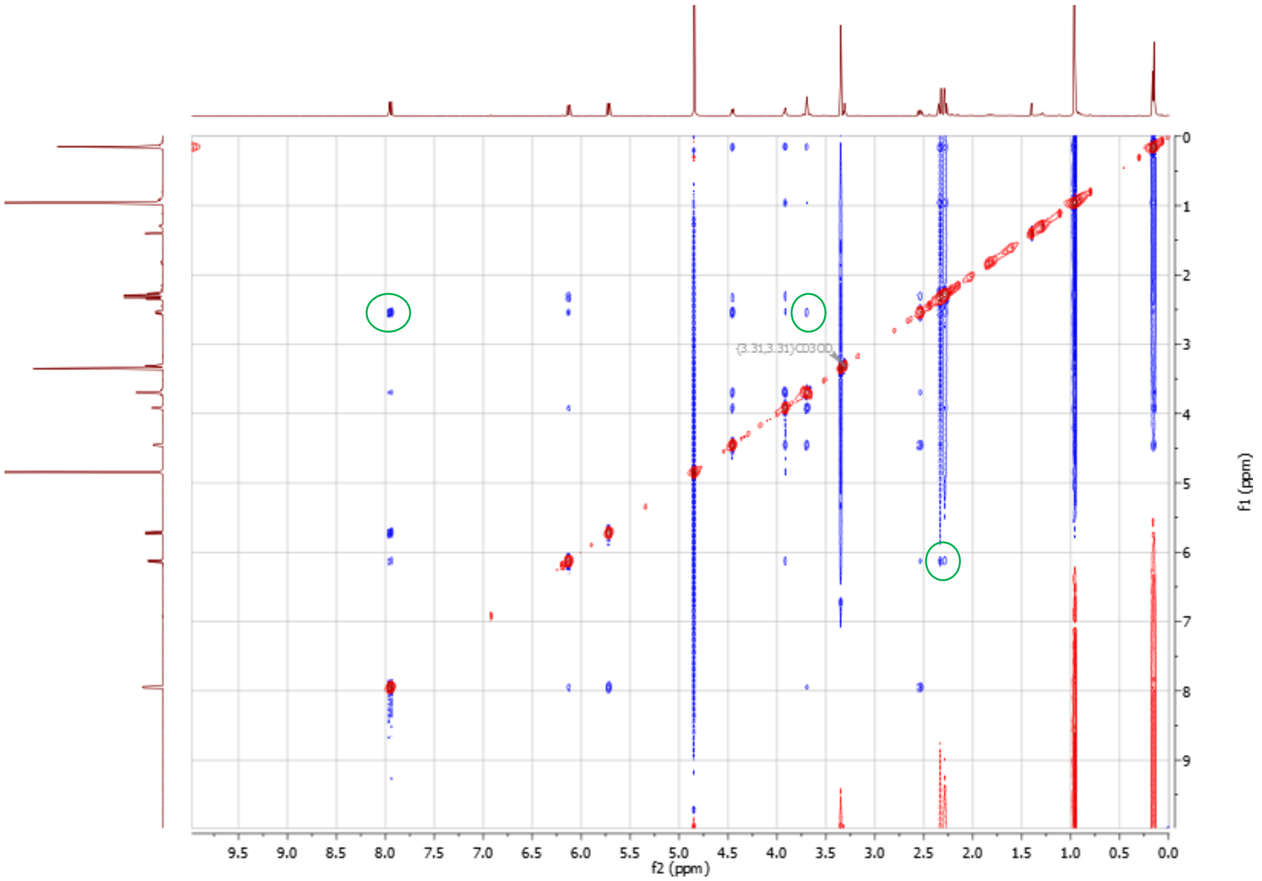

1-((2*R*,3*S*,4*S*,5*R*)-4-((*tert*-butyldimethylsilyl)oxy)-5-(hydroxymethyl)-3-(3-iodobicyclo[1.1.1] pentan-1-yl)tetrahydrofuran-2-yl)pyrimidine-2,4(1*H*,3*H*)-dione, 5w'

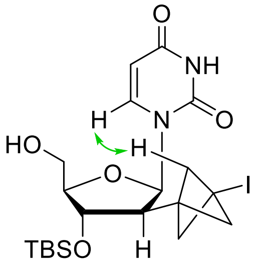

(nOe enhancements indicated in green)

^{1}**H NMR** (400 MHz, CD_3OD)

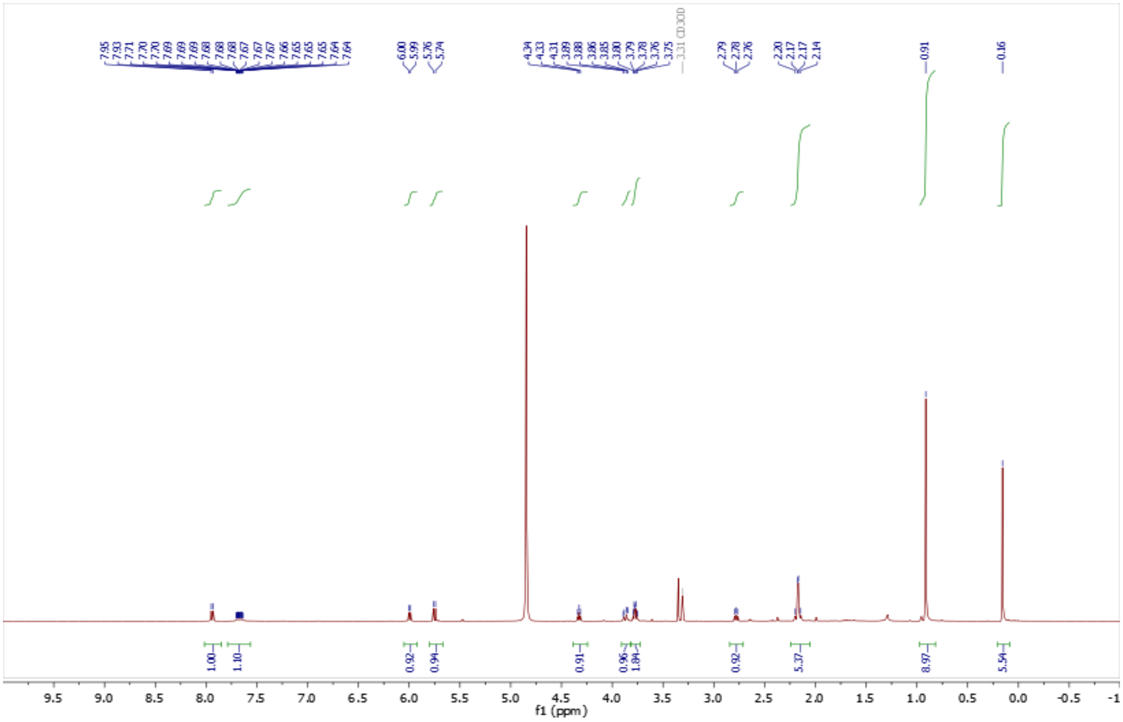

^{13}**C NMR** (101 MHz, CD_3OD)

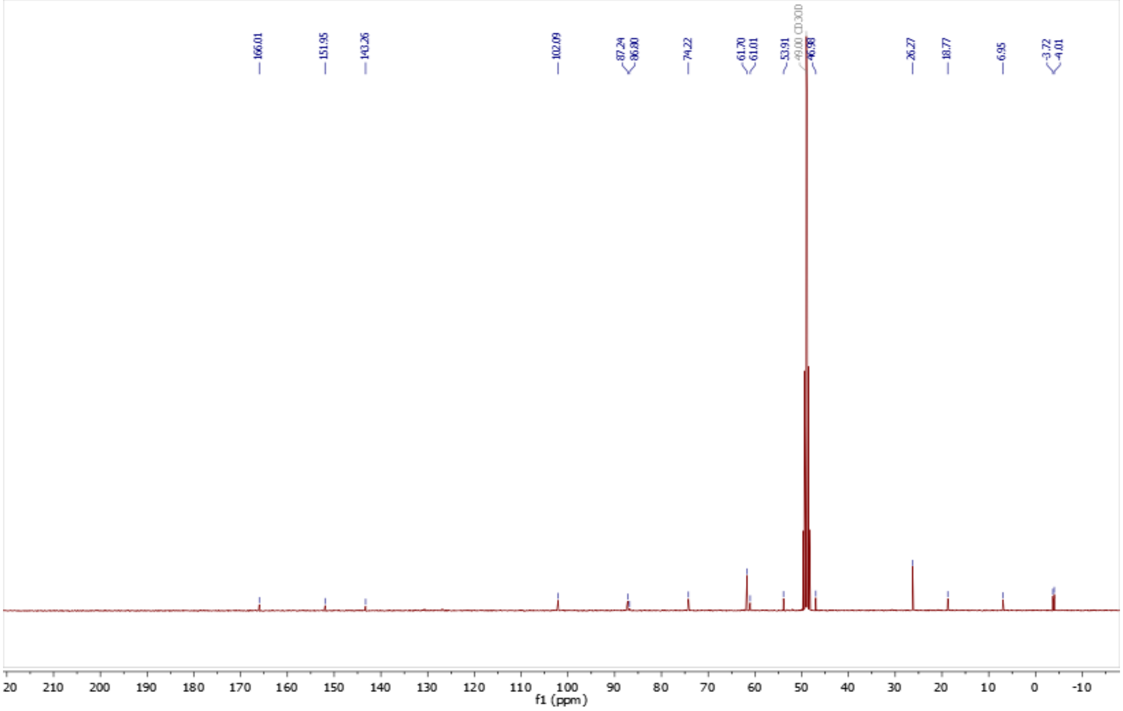

NOESY (400 MHz, CD_3OD)

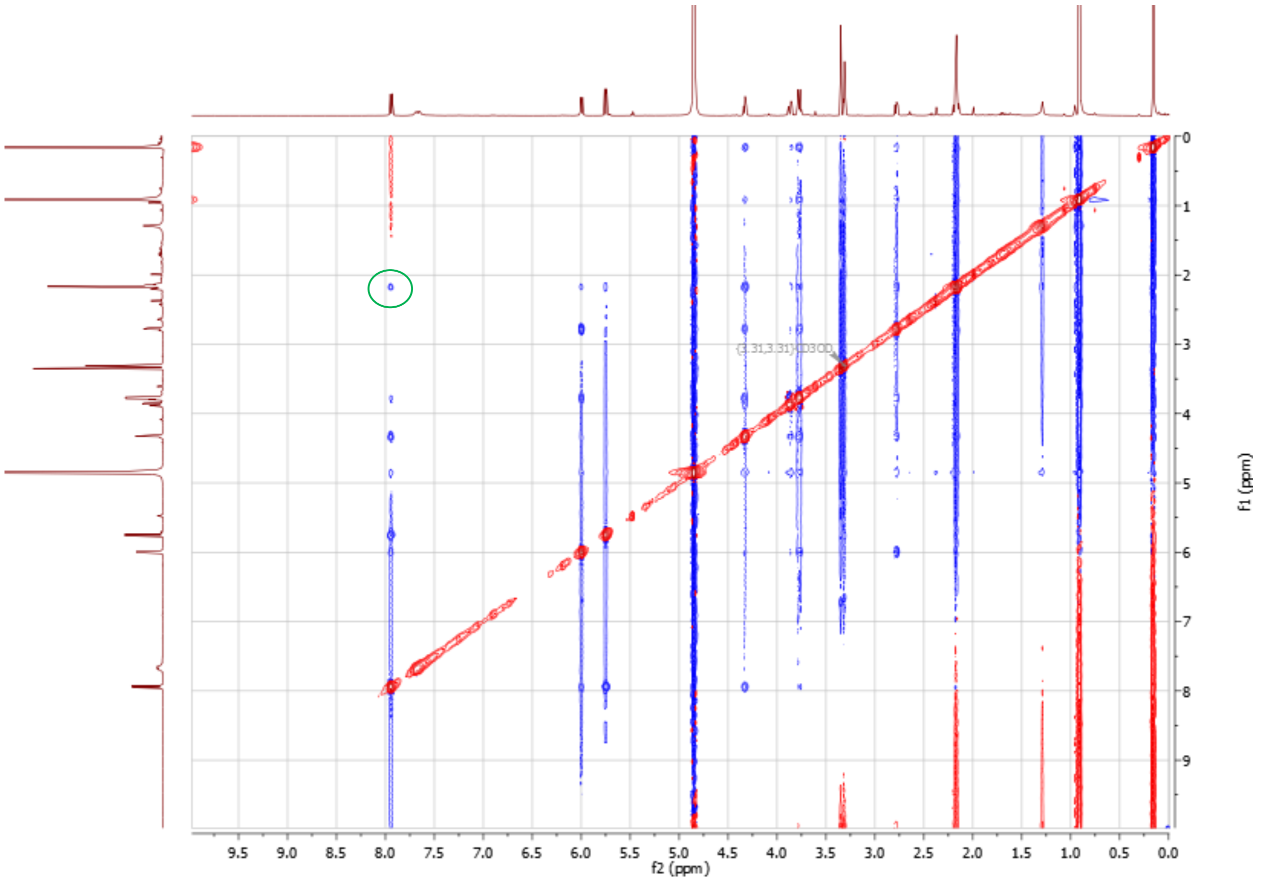

1-((2*R*,3*R*,4*S*,5*R*)-3-(bicyclo[1.1.1]pentan-1-yl)-4-((*tert*-butyldimethylsilyl)oxy)-5-(hydroxymethyl)tetrahydrofuran-2-yl)pyrimidine-2,4(1*H*,3*H*)-dione, 9w

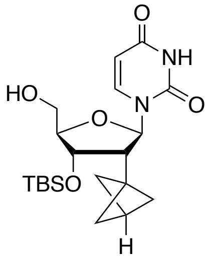

^{1}H NMR (400 MHz, MeOD)

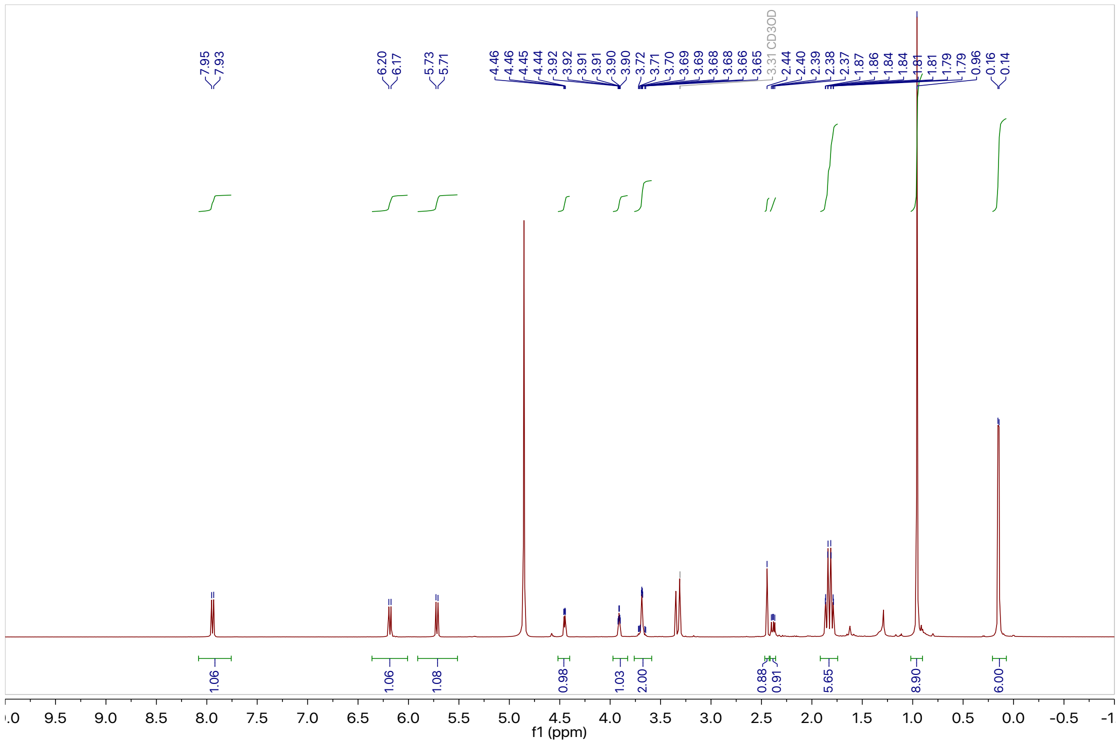

^{13}C NMR (101 MHz, MeOD)

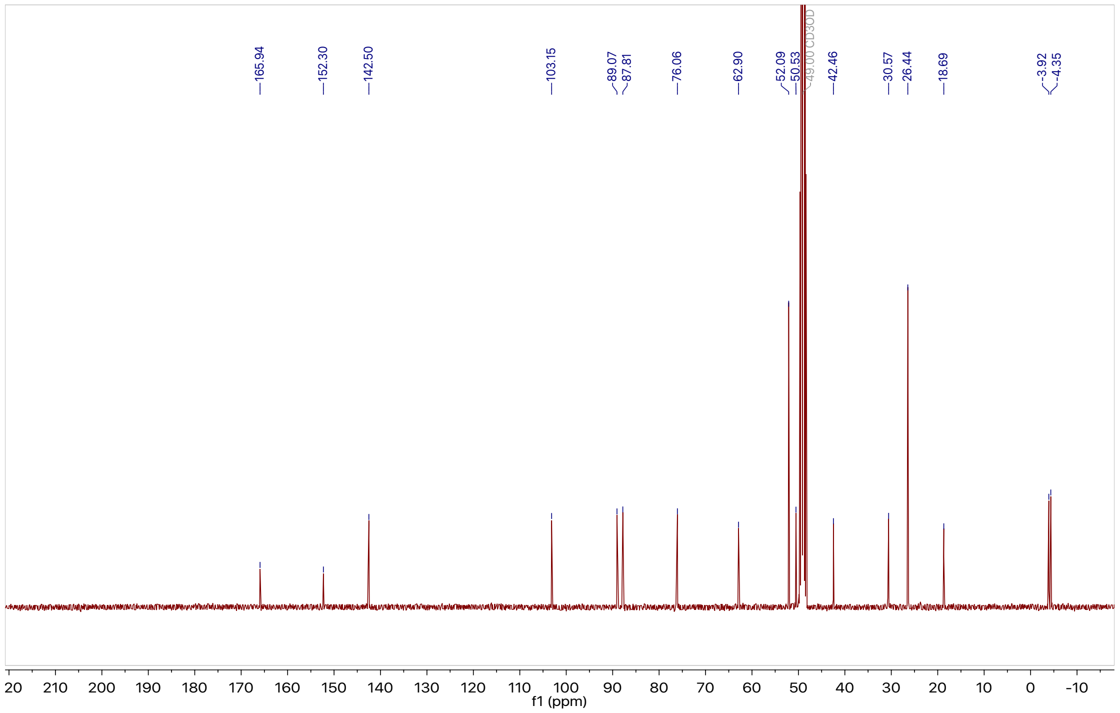

Boc-Asp(O*t*-Bu)-β-(3-iodobicyclo[1.1.1]pentan-1-yl)Ala-OMe, 5x

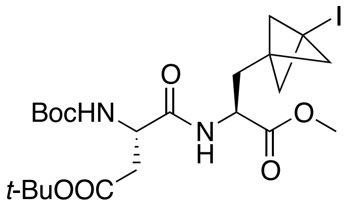

^{1}H NMR (400 MHz, $CDCl_3$)

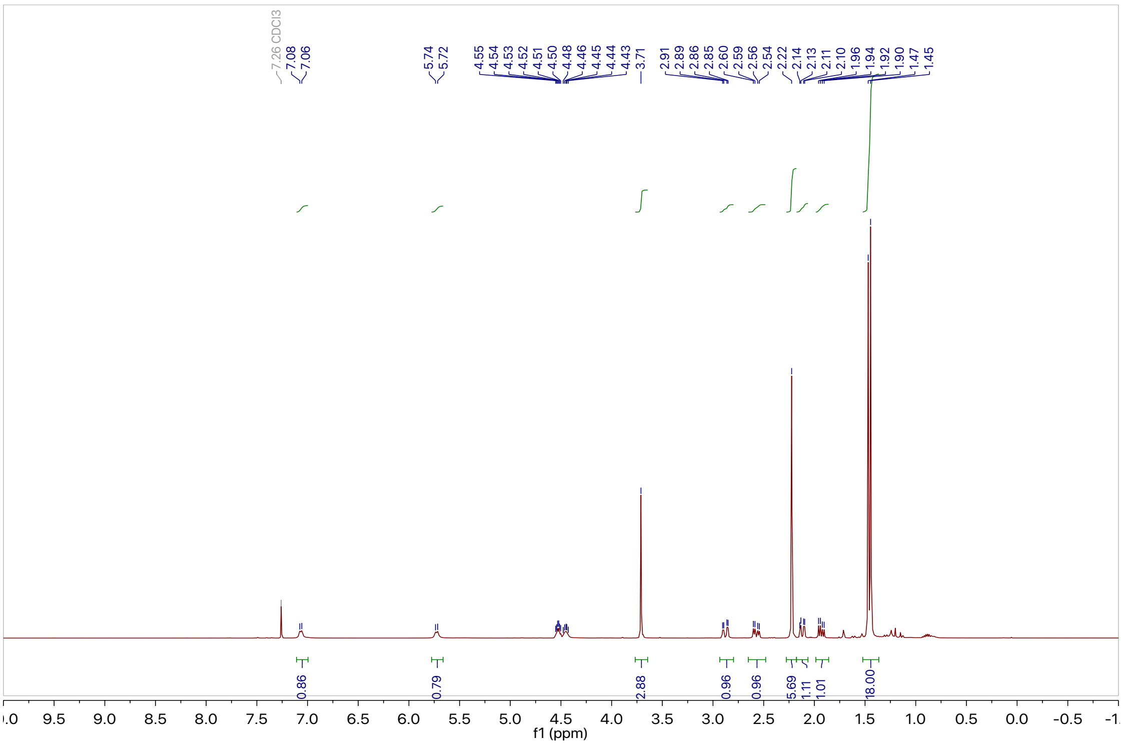

^{13}C NMR (101 MHz, $CDCl_3$)

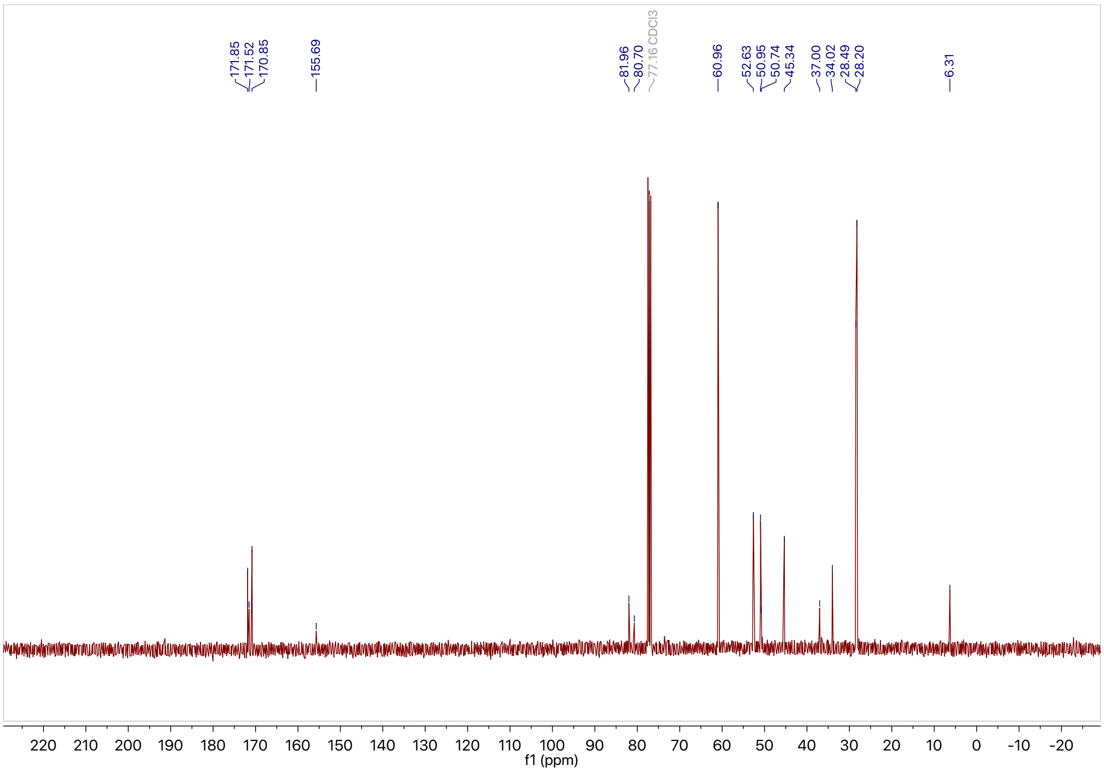

Boc-Asp(O*t*-Bu)-β-(bicyclo[1.1.1]pentan-1-yl)Ala-OMe, 9x

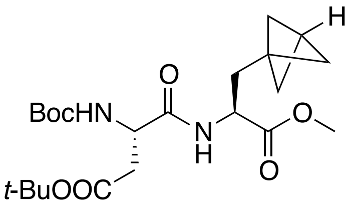

^{1}H NMR (400 MHz, CDCl$_3$)

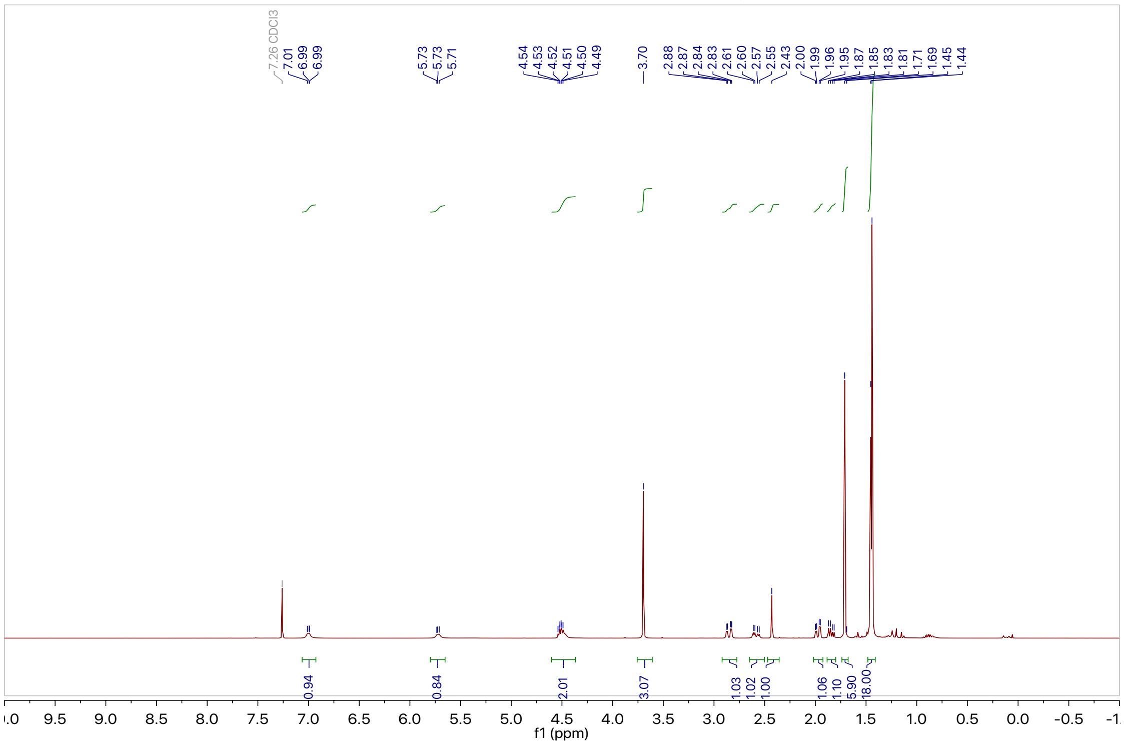

^{13}C NMR (101 MHz, CDCl$_3$)

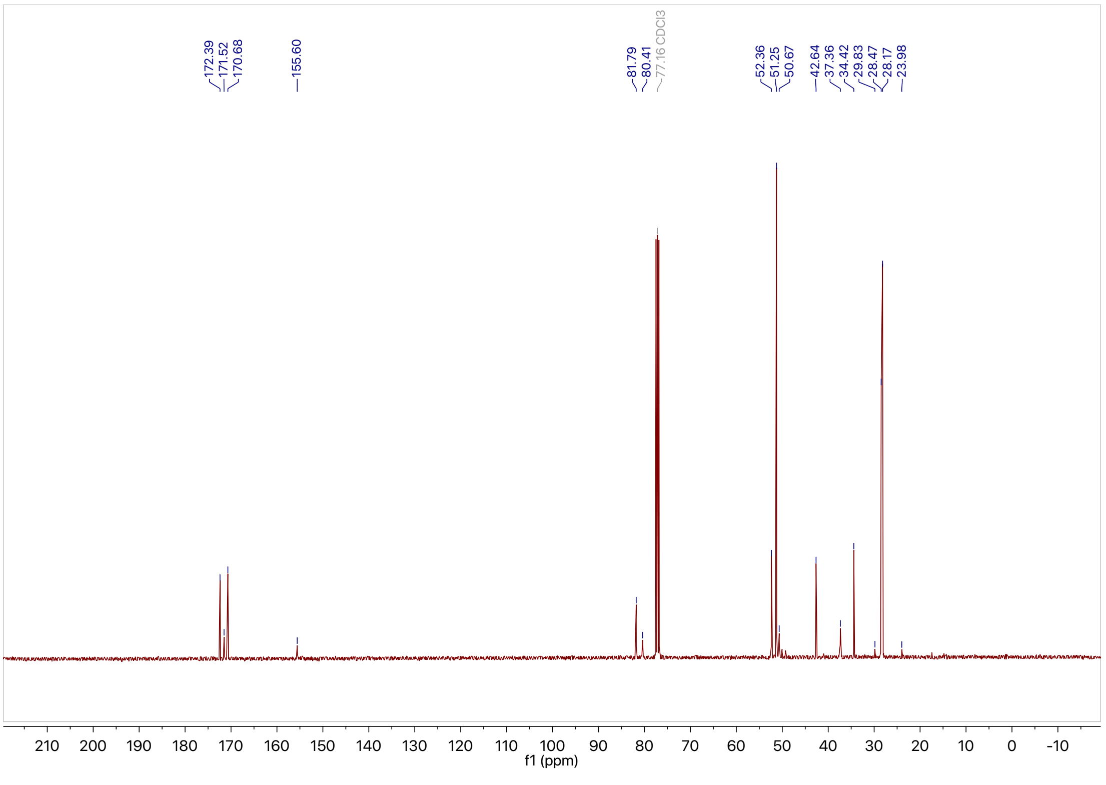

2-(3-iodobicyclo[1.1.1]pentan-1-yl)ethyl 4-methylbenzenesulfonate, 5y

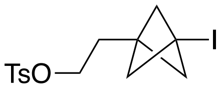

^{1}H NMR (400 MHz, CDCl$_3$)

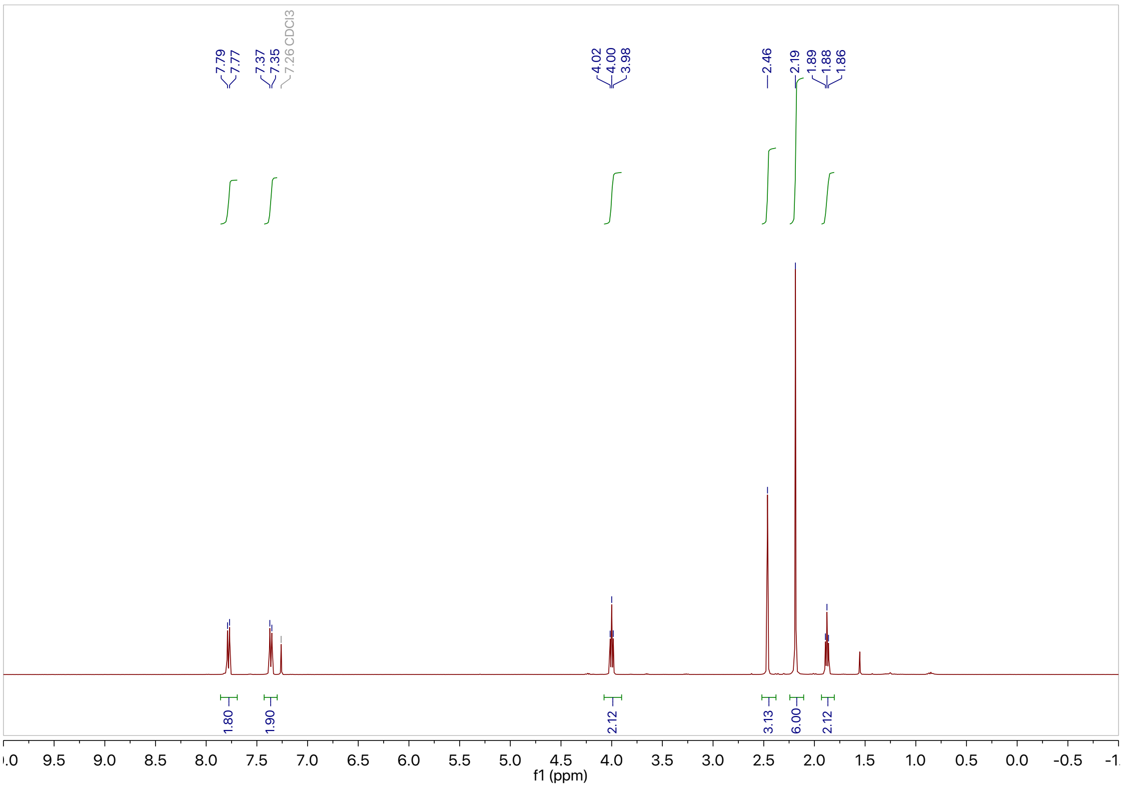

^{13}C NMR (101 MHz, CDCl$_3$)

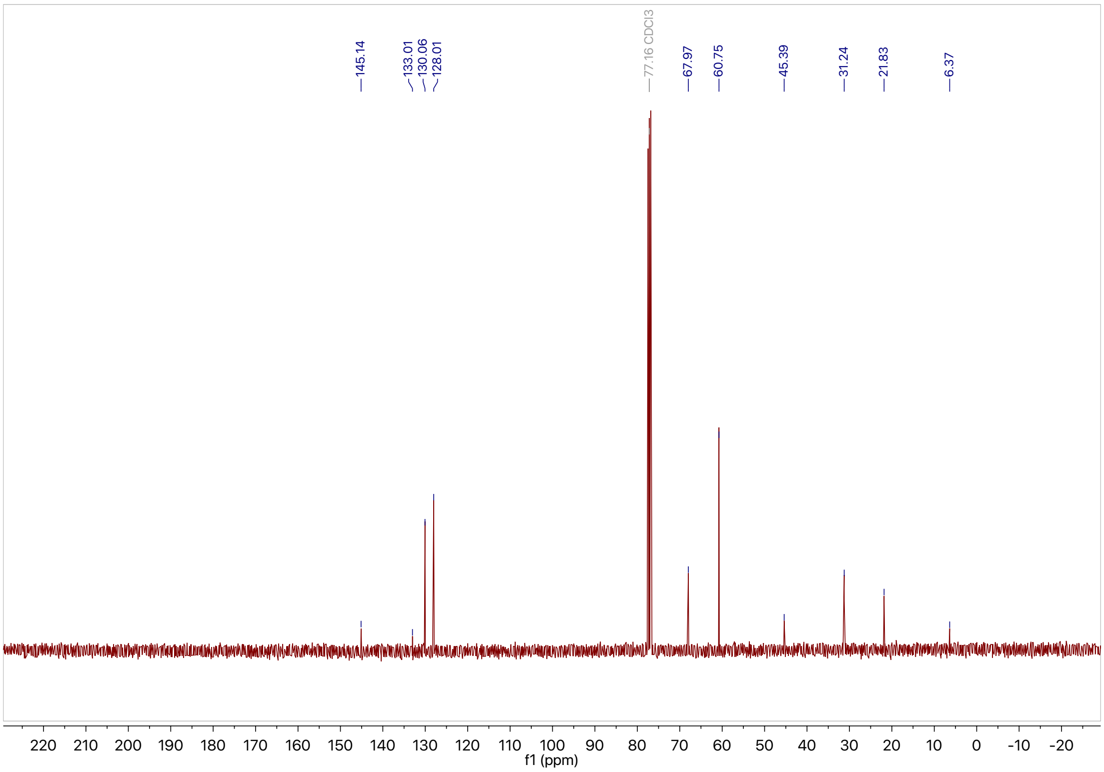

2-(bicyclo[1.1.1]pentan-1-yl)ethyl 4-methylbenzenesulfonate, 9y

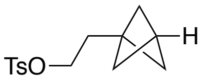

^{1}H NMR (400 MHz, $CDCl_3$)

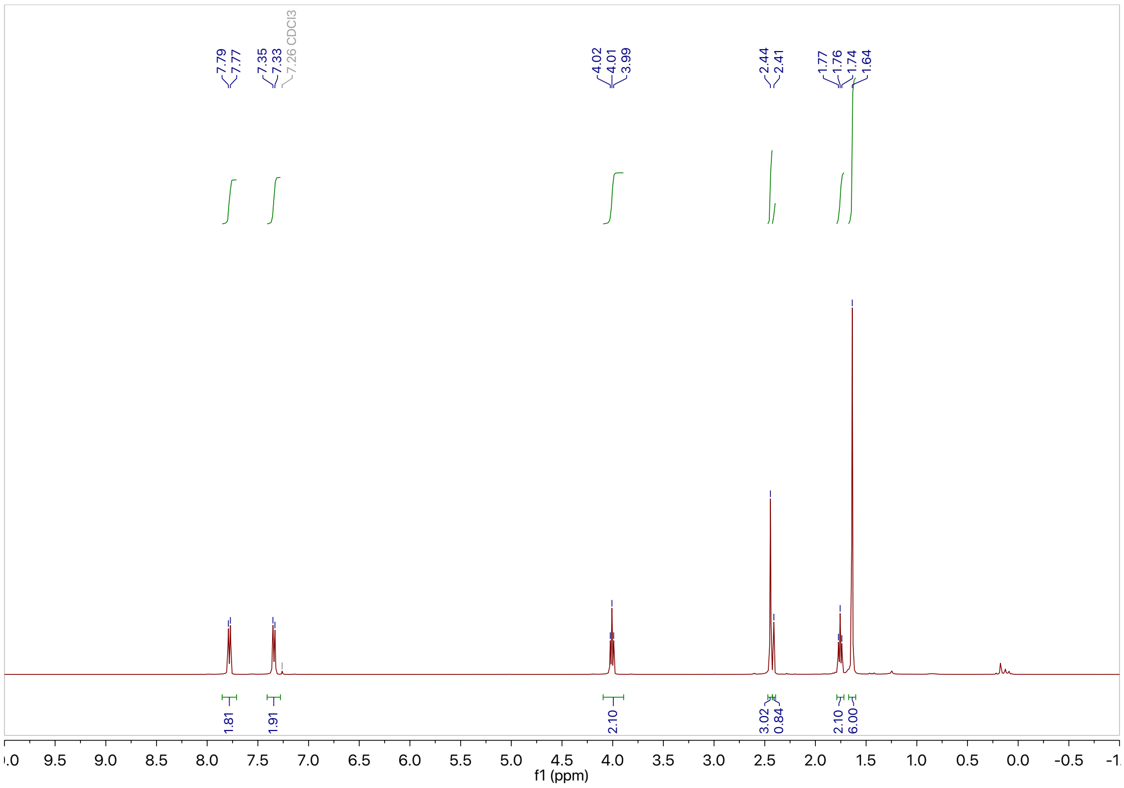

^{13}C NMR (101 MHz, $CDCl_3$)

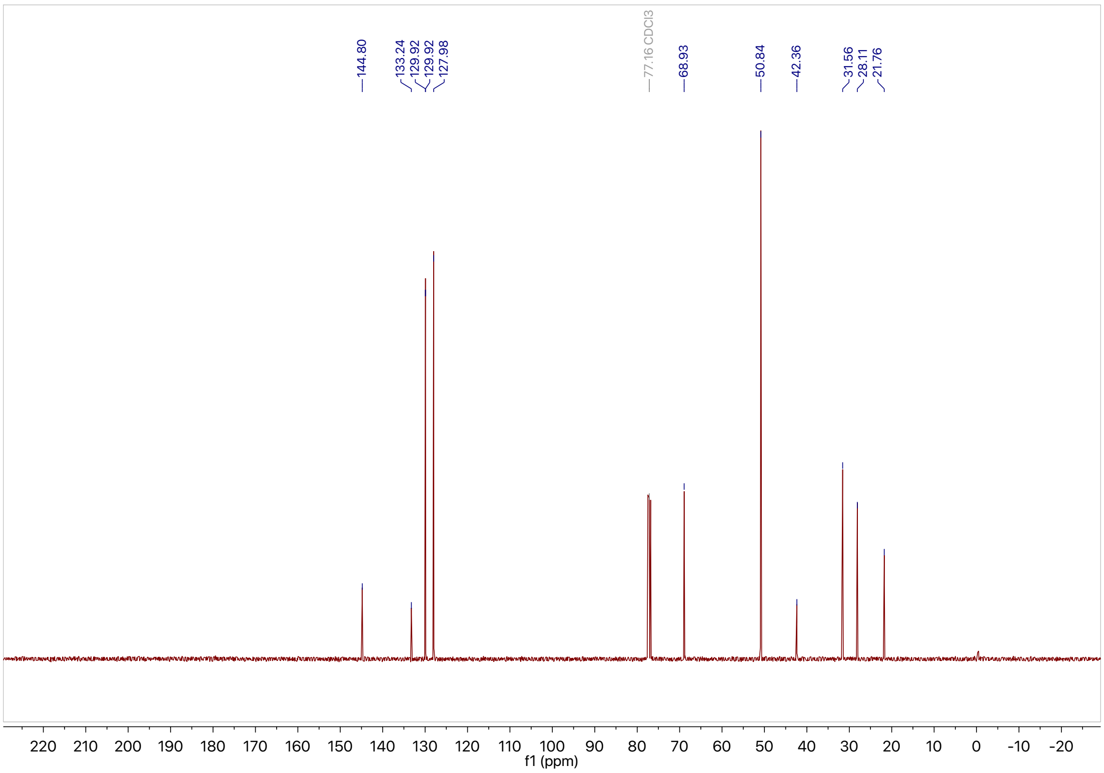

N-(1-(2-(bicyclo[1.1.1]pentan-1-yl)ethyl)piperidin-4-yl)-N-phenylpropionamide, (BCP-fentanyl), 18

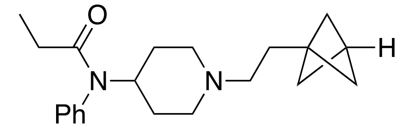

^{1}H NMR (400 MHz, $CDCl_3$)

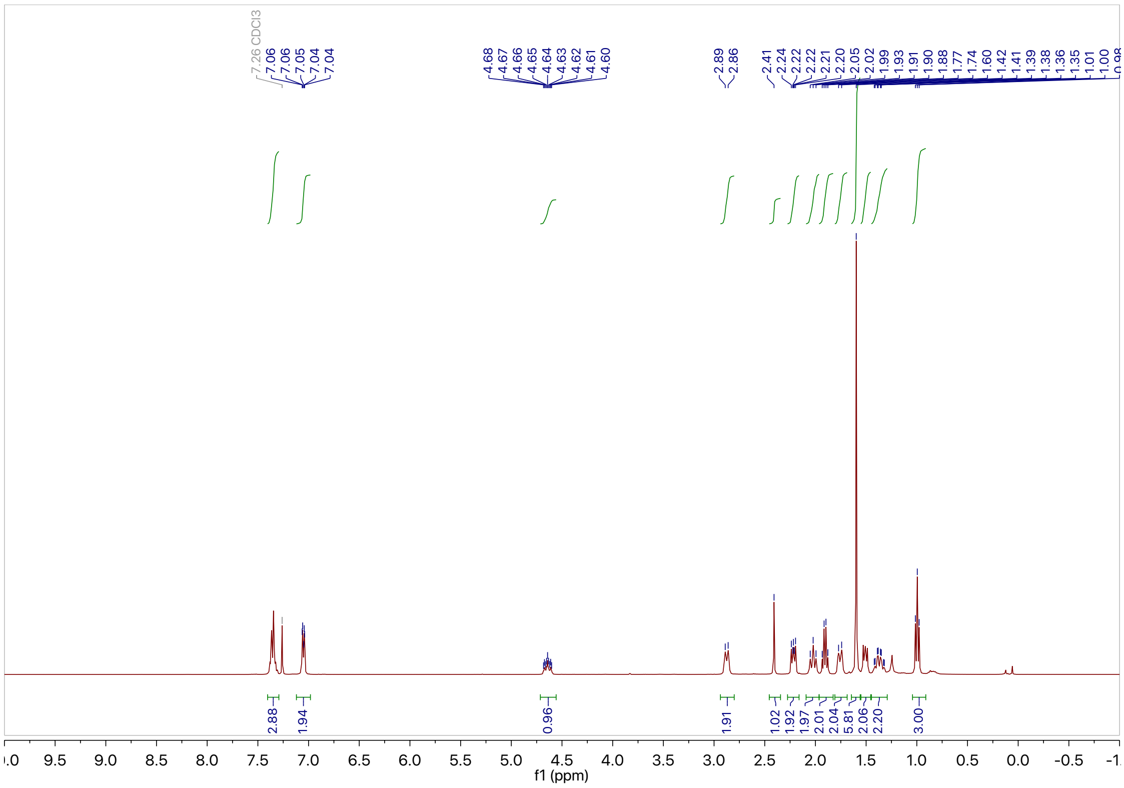

^{13}C NMR (101 Hz, $CDCl_3$)

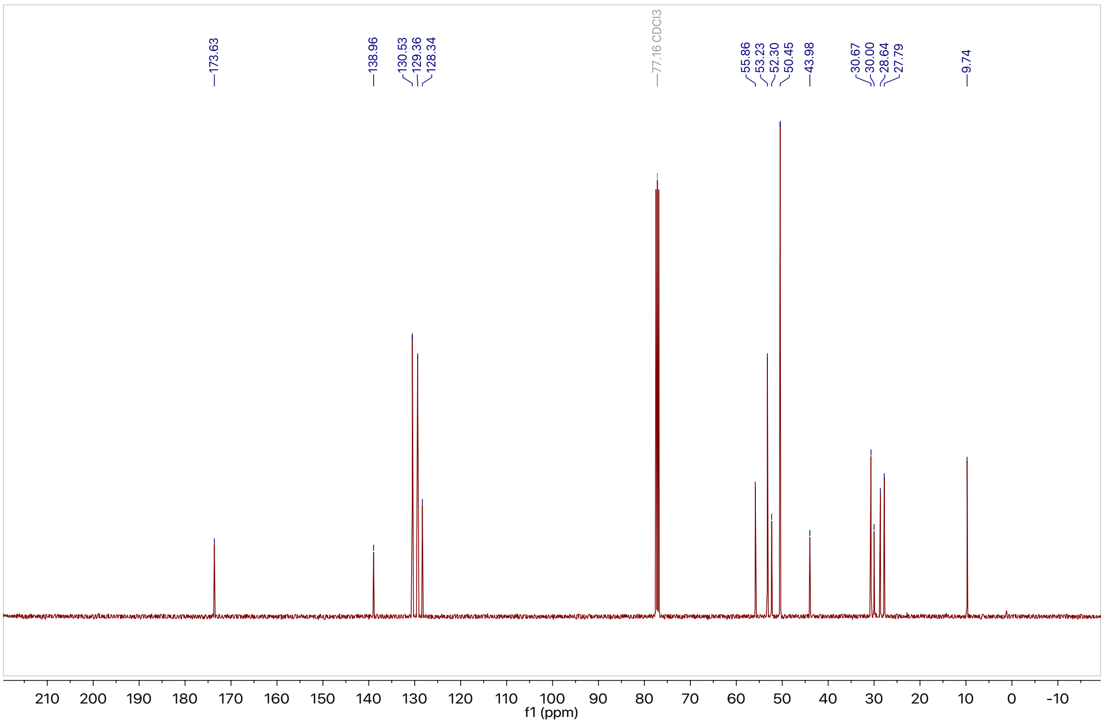

Ethyl iodoacetate, 7a

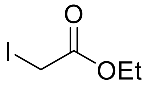

^{1}H NMR (400 MHz, $CDCl_3$)

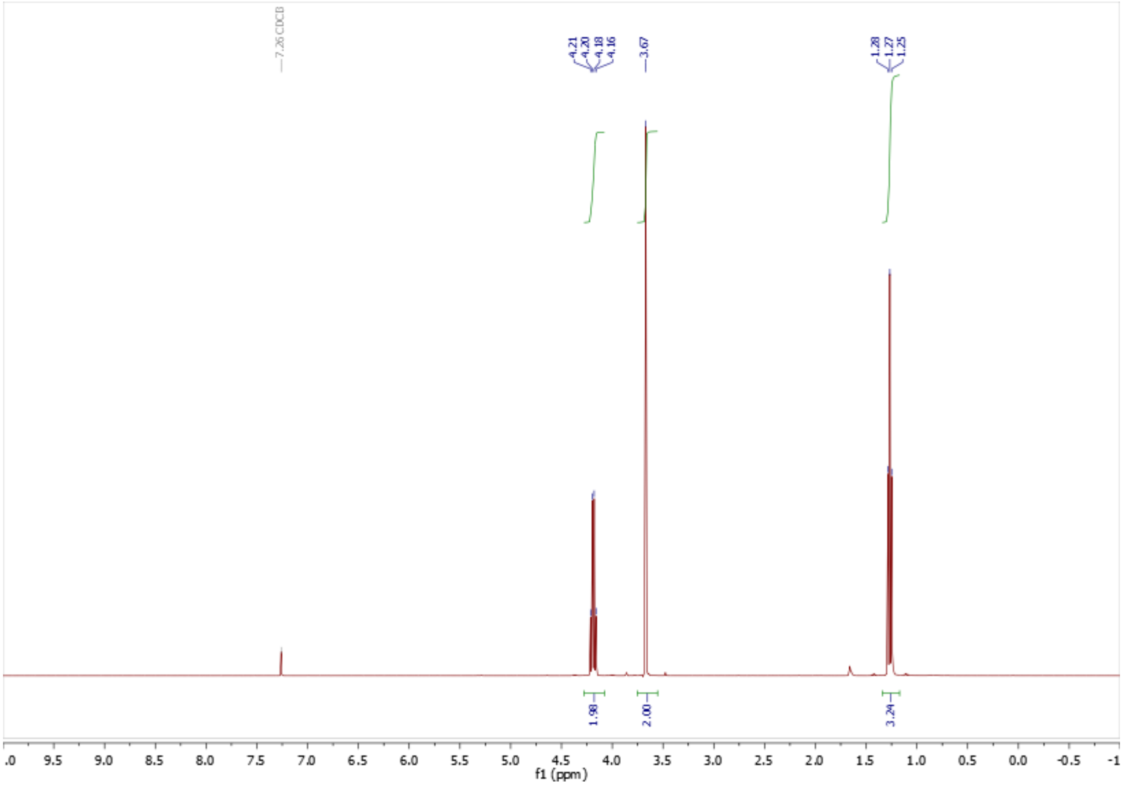

^{13}C NMR (101 MHz, $CDCl_3$)

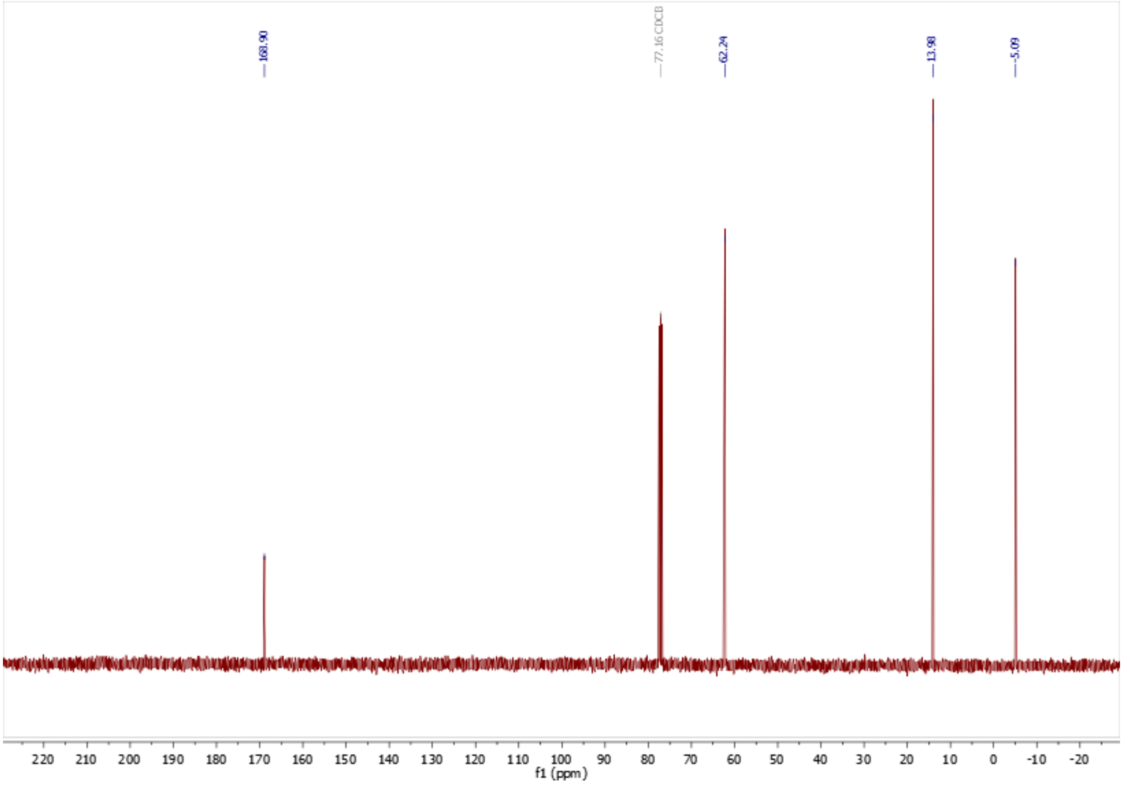

(2-iodoethyl)benzene, **7c**

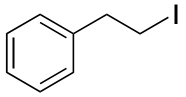

^{1}H NMR (400 MHz, $CDCl_3$)

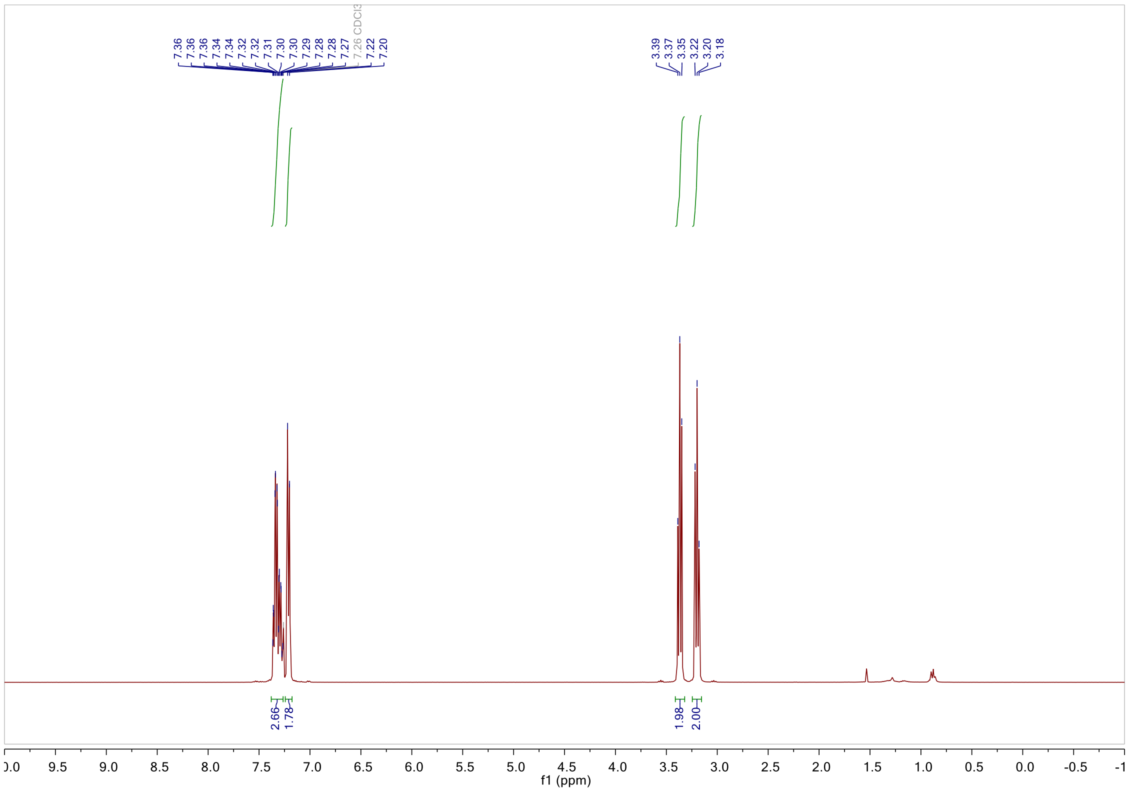

^{13}C NMR (101 MHz, $CDCl_3$)

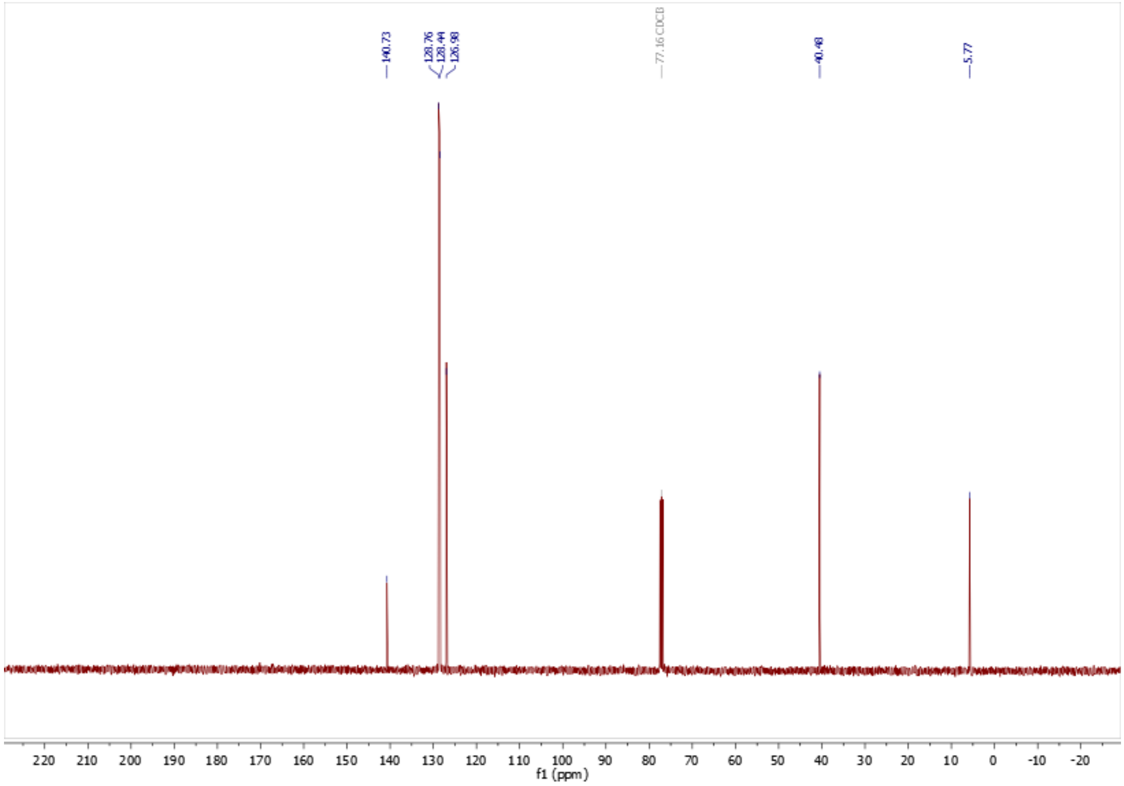

***tert*-Butyl (2-iodoethyl)carbamate, 7e**

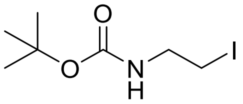

[1]**H NMR** (400 MHz, $CDCl_3$)

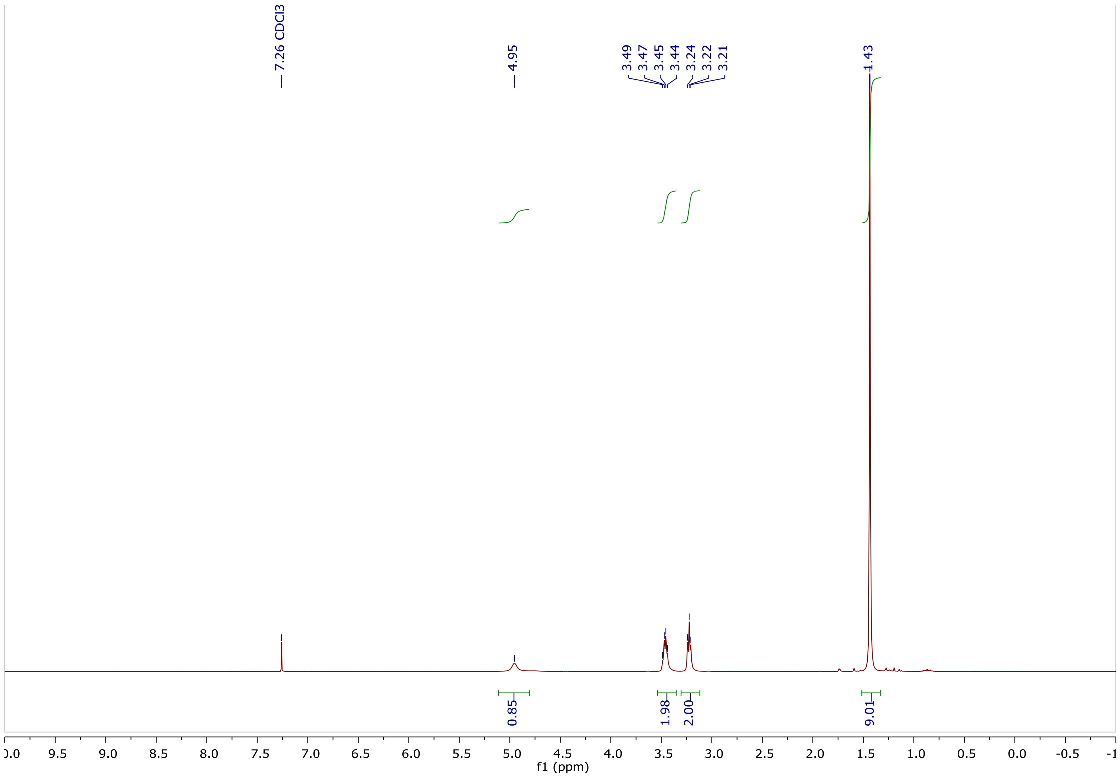

[13]**C NMR** (101 MHz, $CDCl_3$)

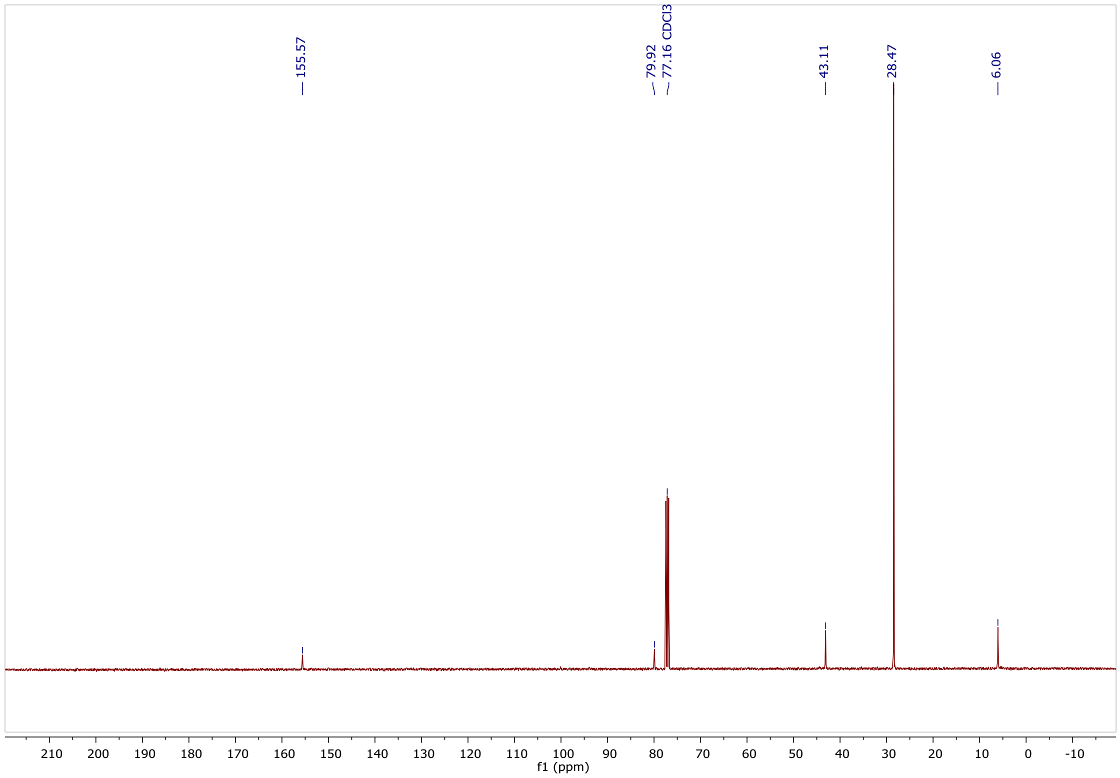

2-Iodo-1-phenylethan-1-one, 7f

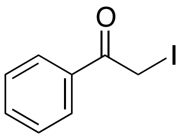

^{1}H NMR (400 MHz, $CDCl_3$)

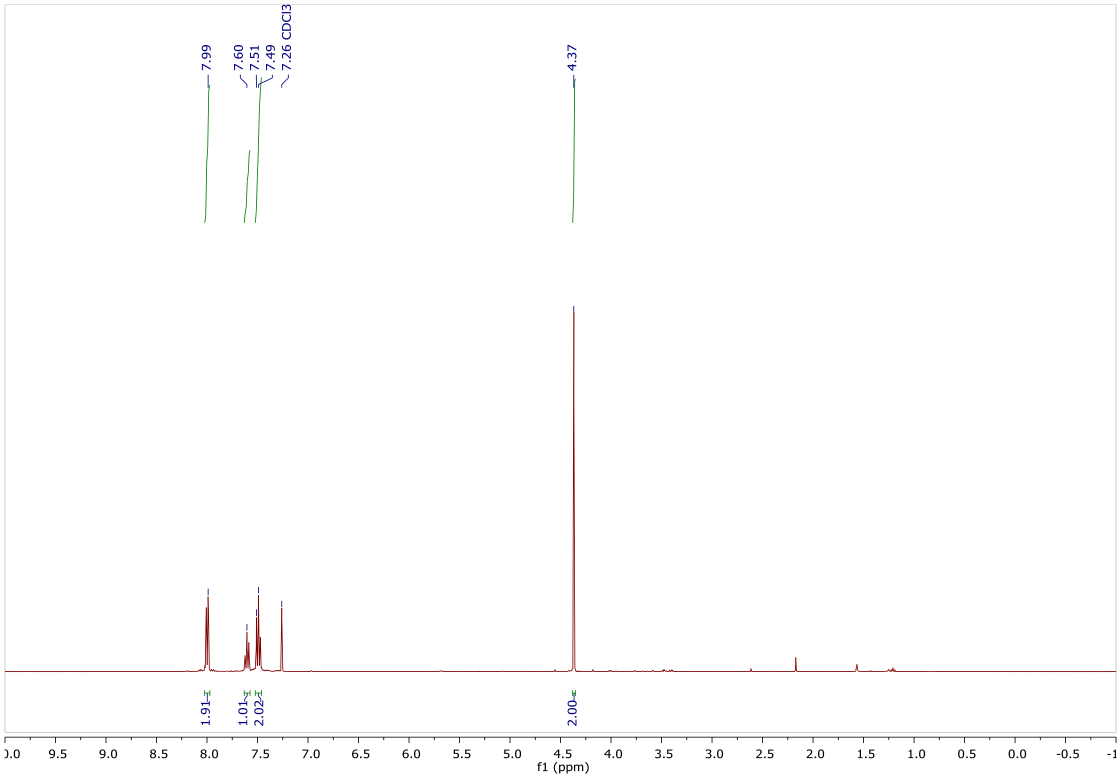

^{13}C NMR (101 MHz, $CDCl_3$)

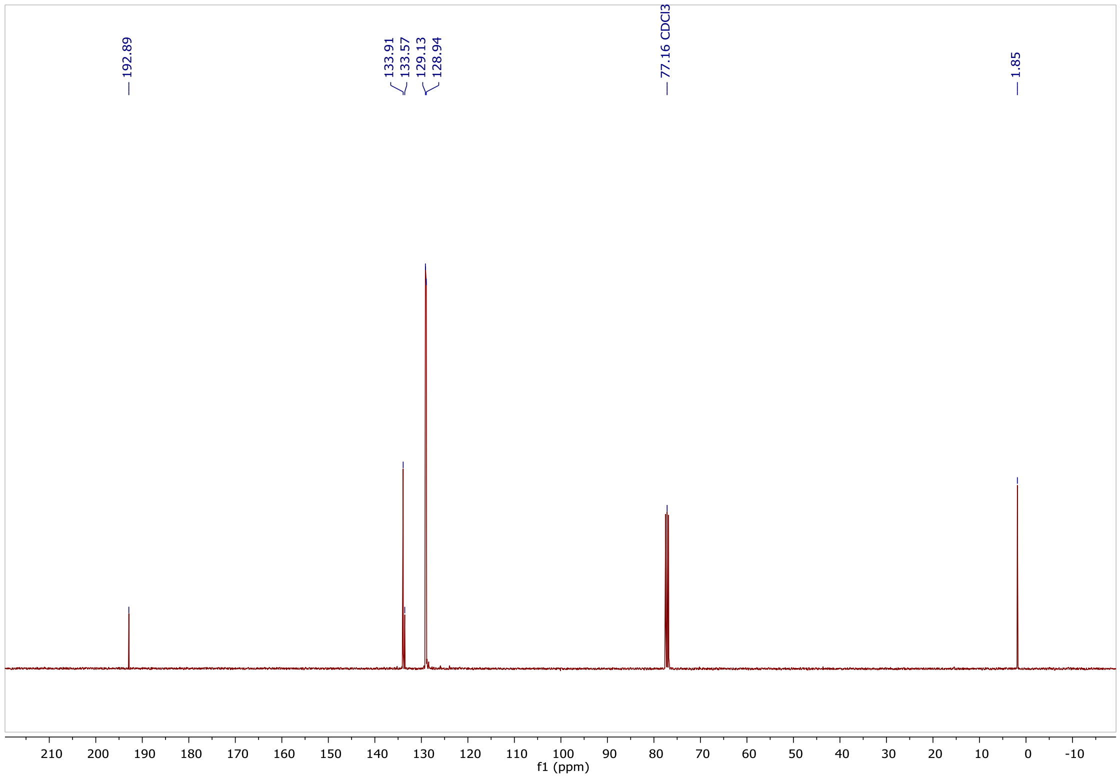

1-(Furan-2-yl)-2-iodoethan-1-one, 7g

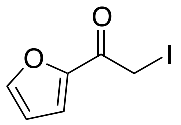

¹H NMR (400 MHz, $CDCl_3$)

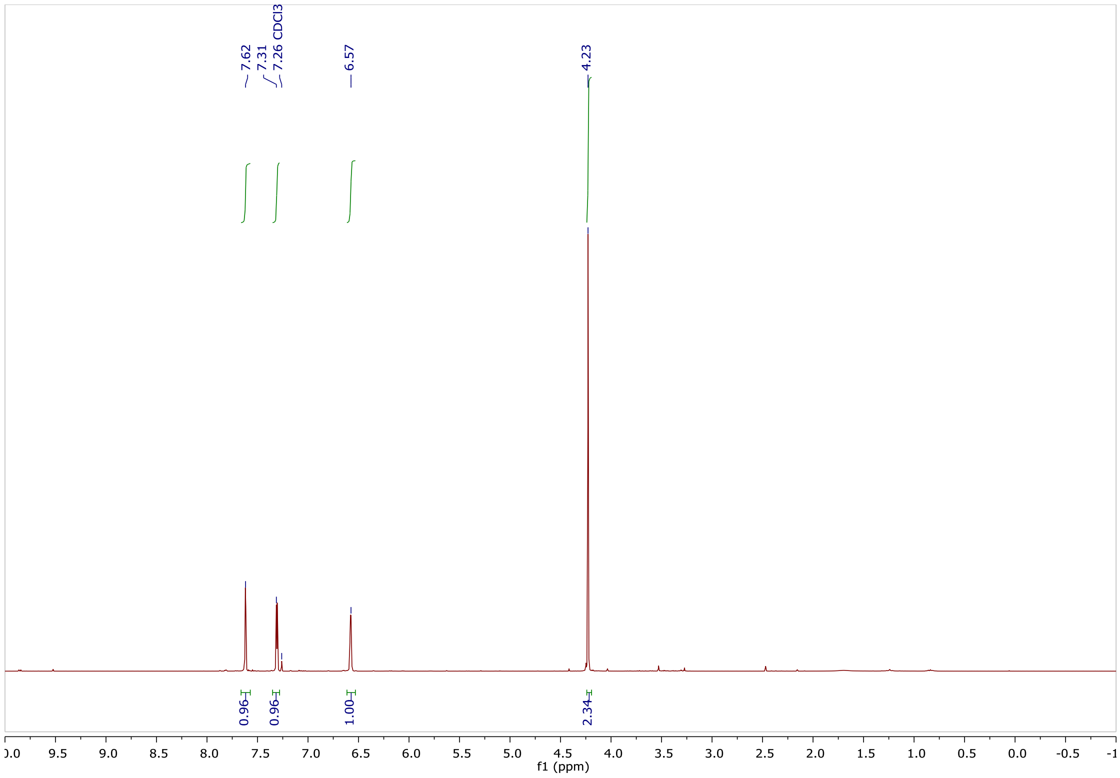

¹³C NMR (101 MHz, $CDCl_3$)

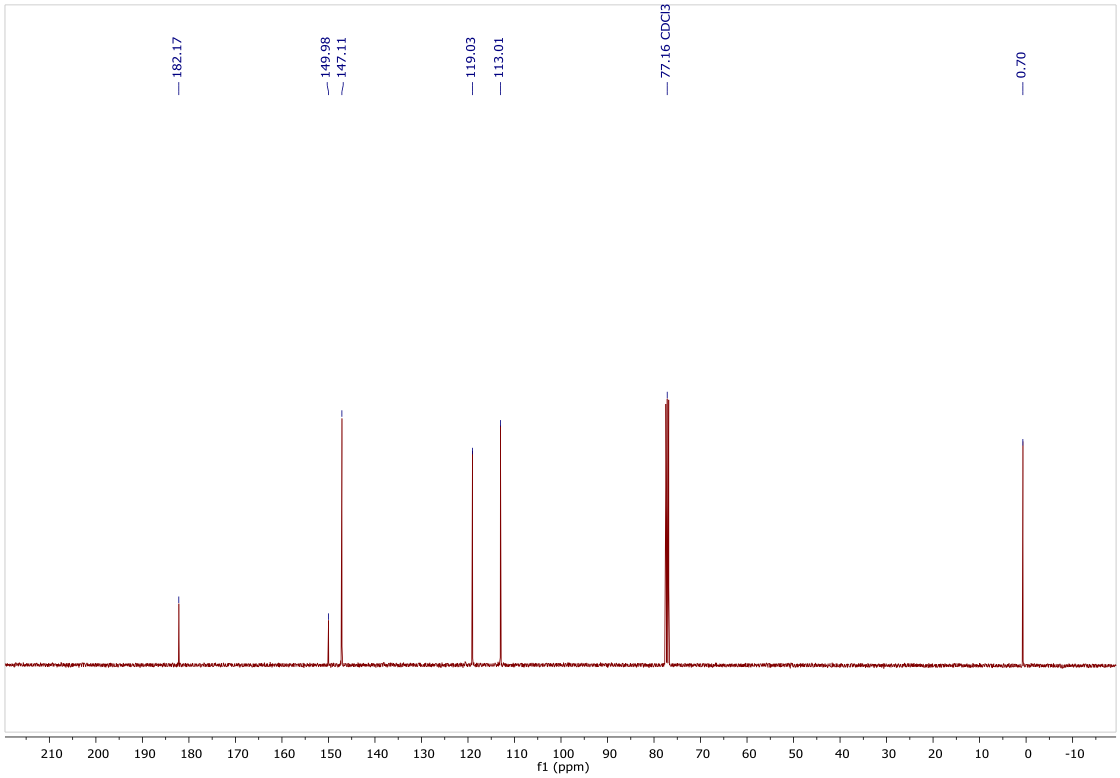

***tert*-Butyl 2-acetyl-1*H*-pyrrole-1-carboxylate, S1**

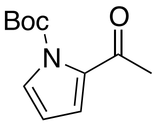

[1]**H NMR** (400 MHz, $CDCl_3$)

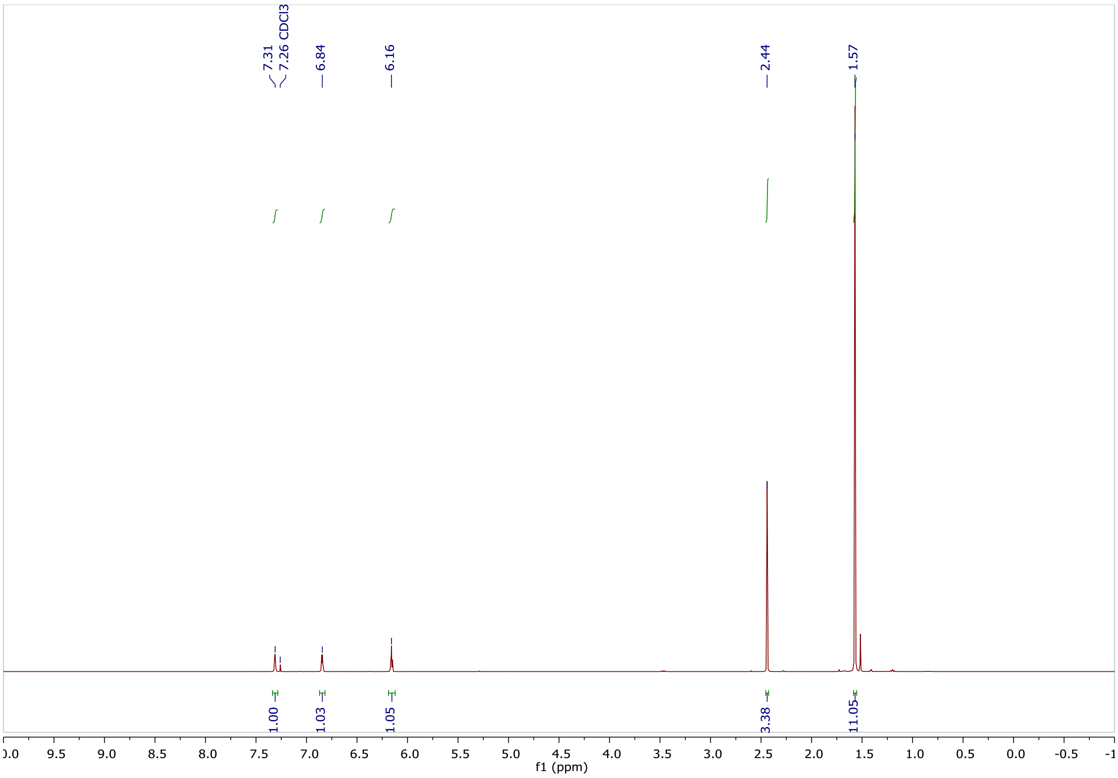

[13]**C NMR** (101 MHz, $CDCl_3$)

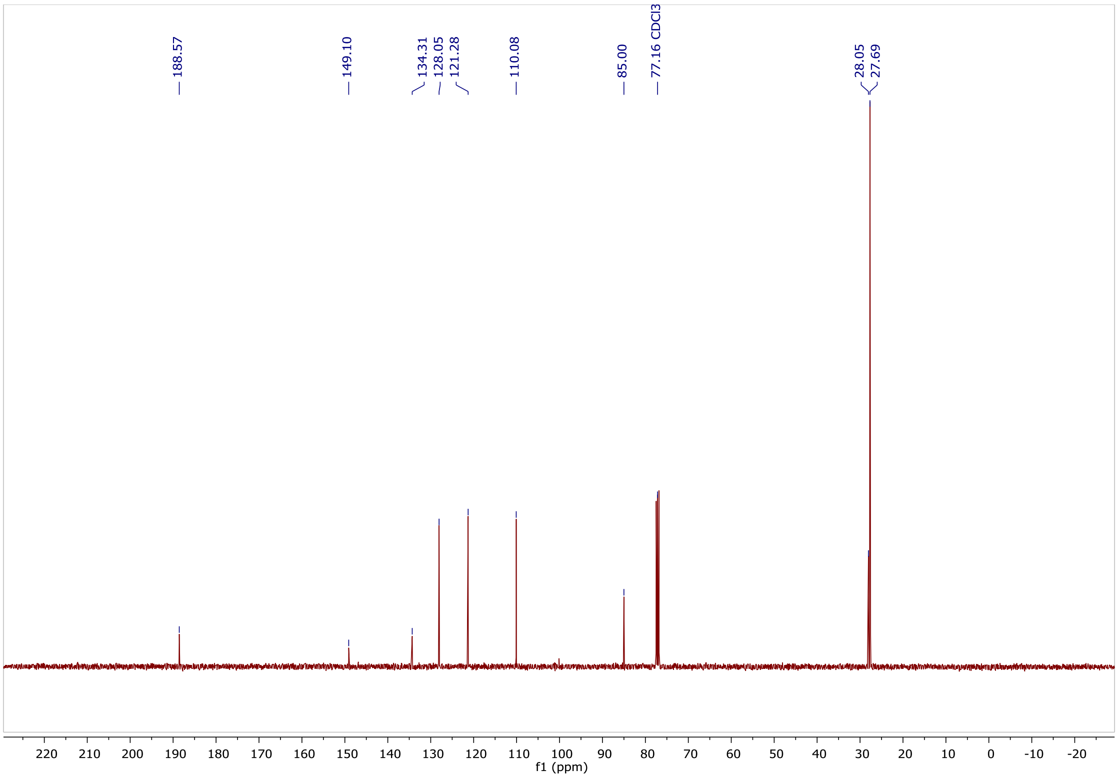

2-Iodo-1-(1*H*-pyrrol-2-yl)ethan-1-one, 7h

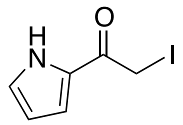

¹H NMR (400 MHz, CDCl₃)

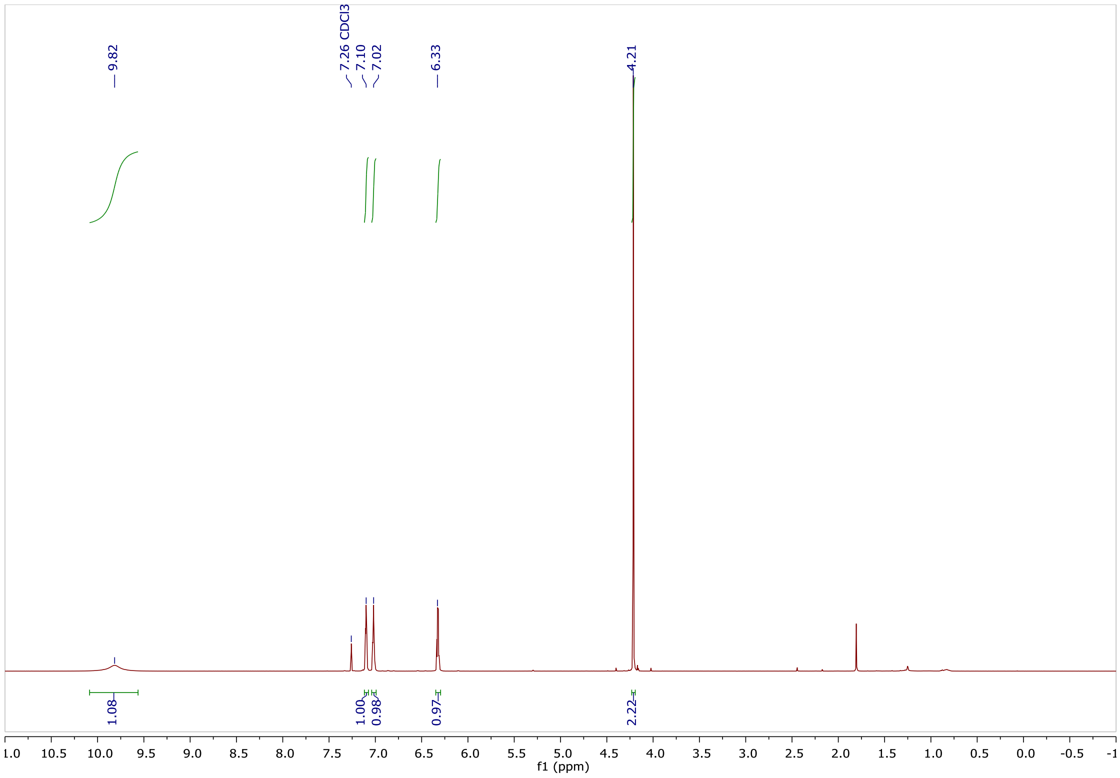

¹³C NMR (101 MHz, CDCl₃)

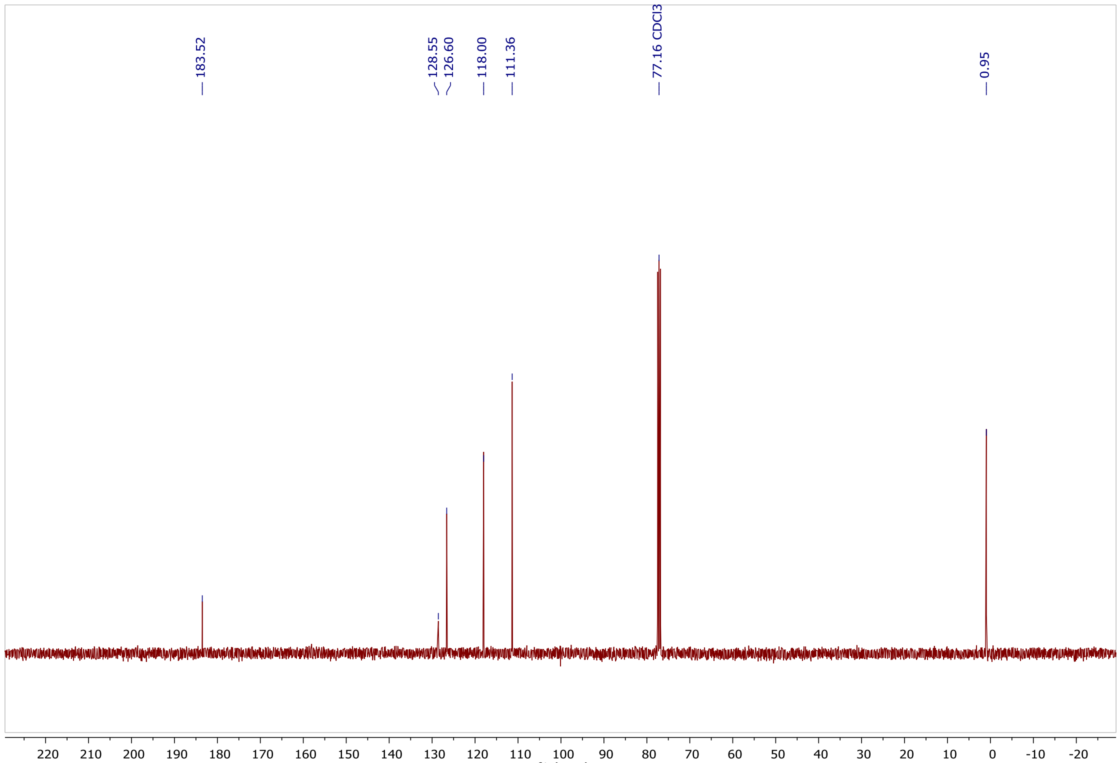

2-Iodo-1-(thiophen-2-yl)ethan-1-one, 7i

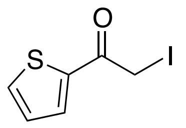

^{1}H NMR (400 MHz, $CDCl_3$)

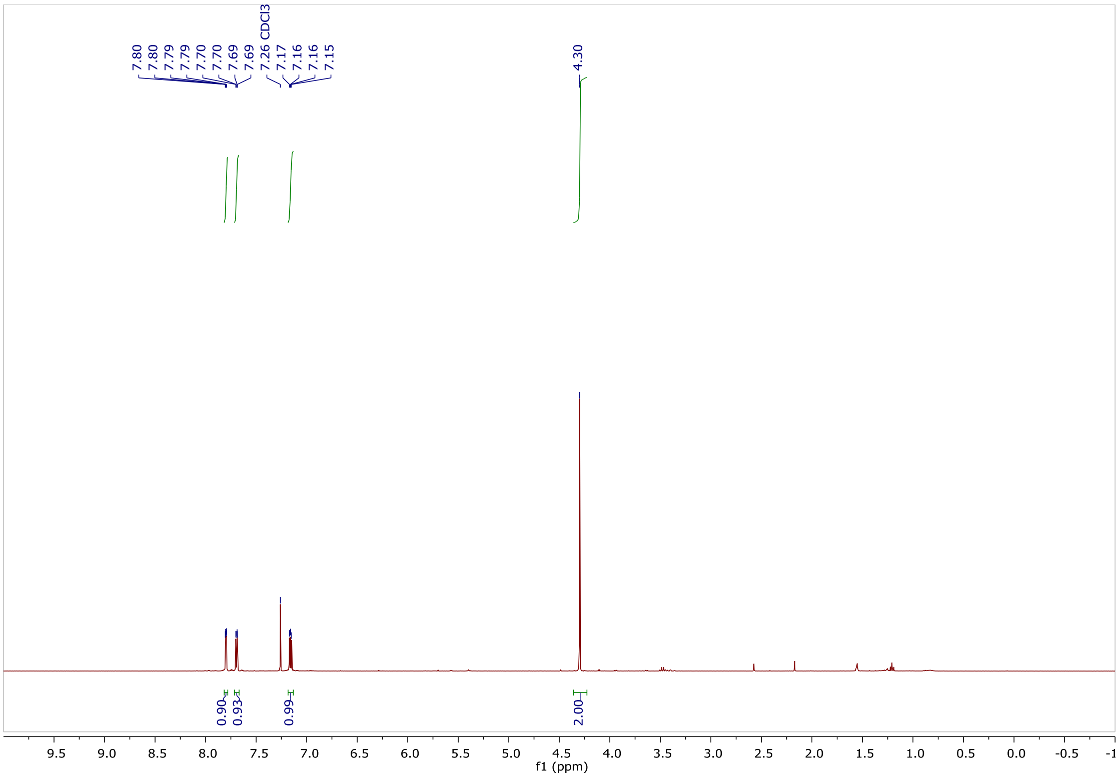

^{13}C NMR (101 MHz, $CDCl_3$)

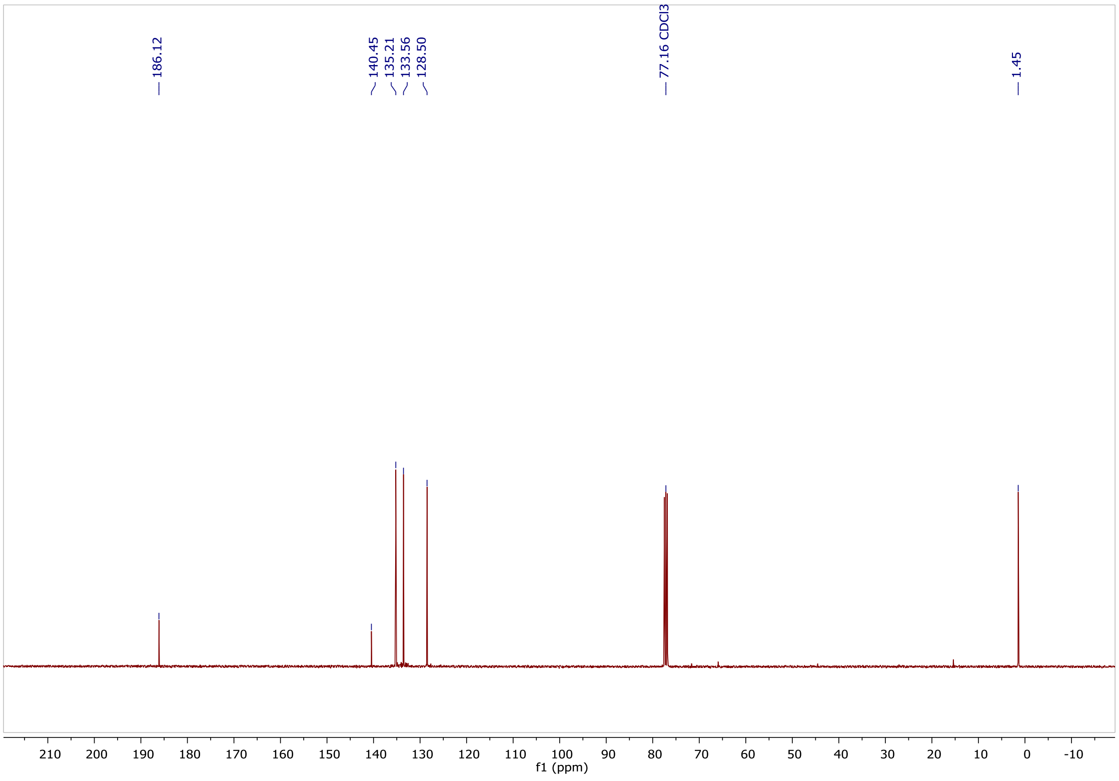

1-(Benzofuran-2-yl)-2-iodoethan-1-one, 7j

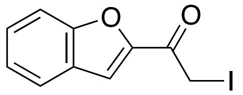

^{1}H NMR (400 MHz, $CDCl_3$)

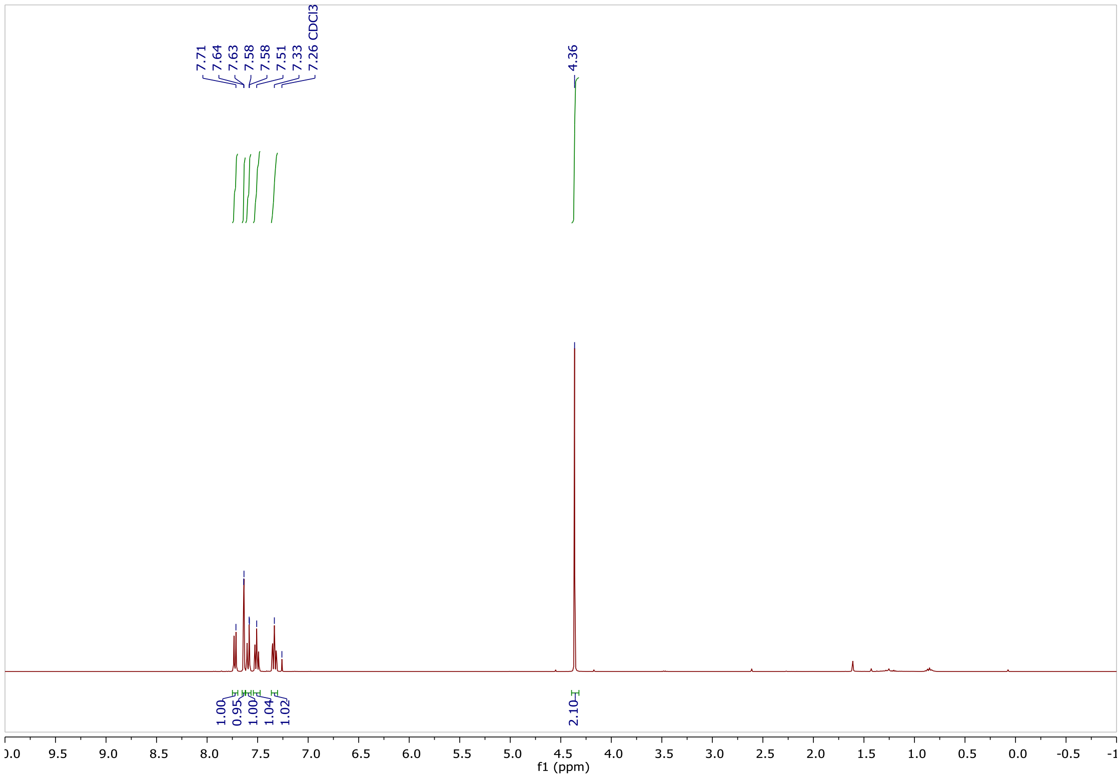

^{13}C NMR (101 MHz, $CDCl_3$)

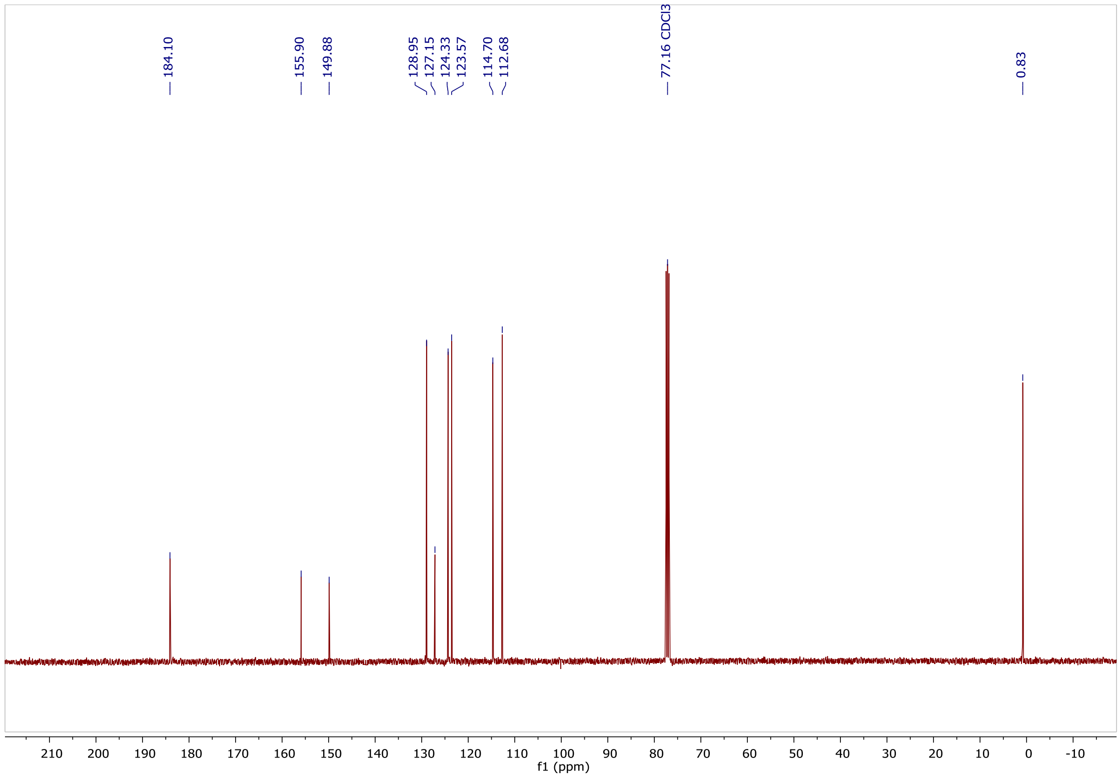

***tert*-Butyl 3-acetyl-1*H*-indole-1-carboxylate, S2**

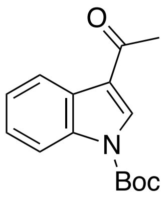

^{1}H NMR (400 MHz, CDCl$_3$)

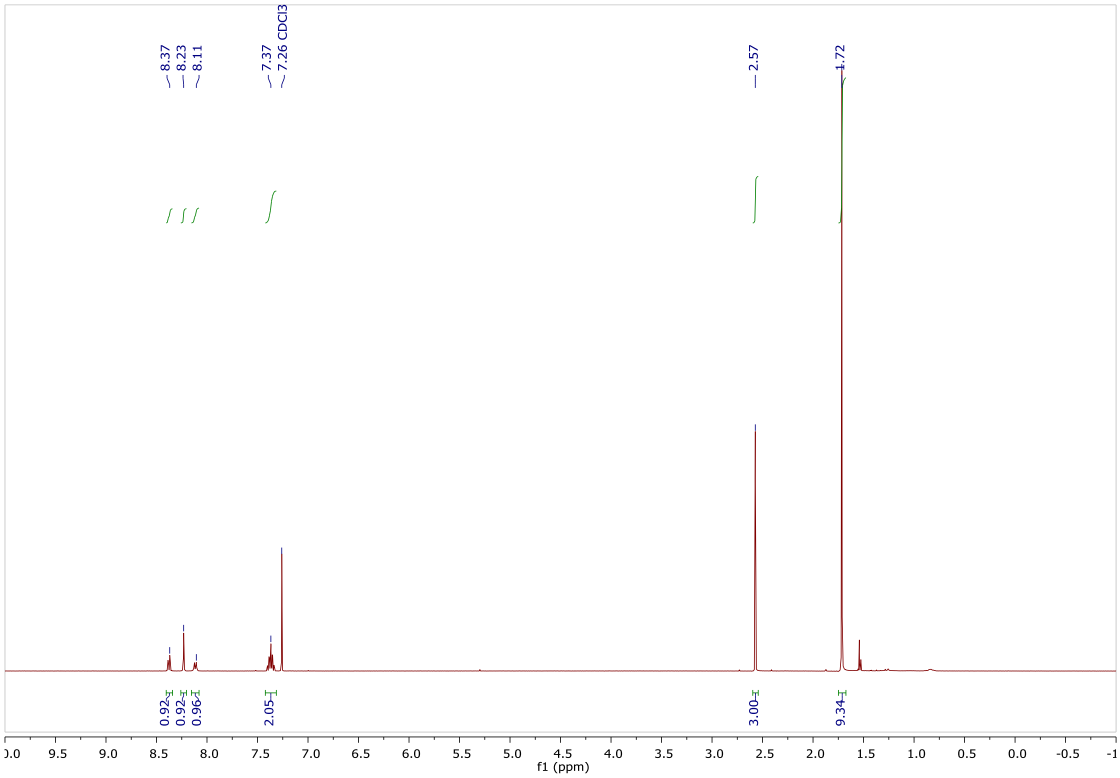

^{13}C NMR (101 MHz, CDCl$_3$)

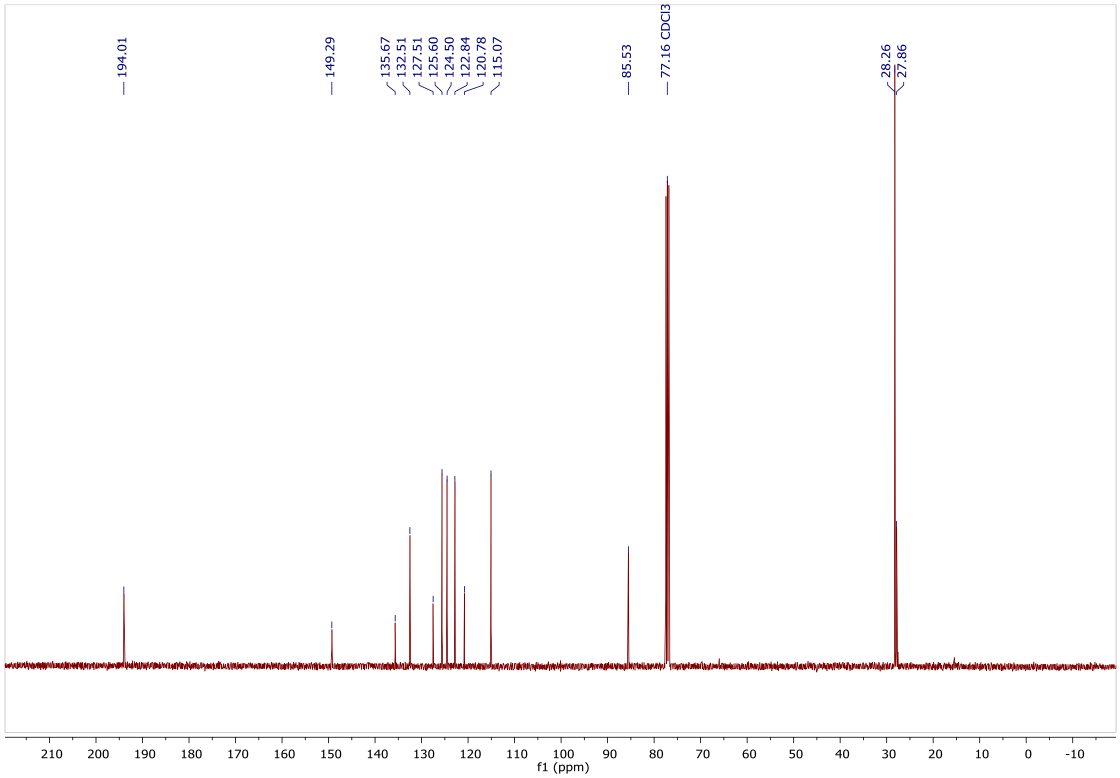

tert-Butyl 3-(2-iodoacetyl)-1_H_-indole-1-carboxylate, 7k

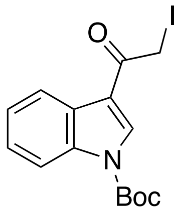

^{1}H NMR (400 MHz, $CDCl_3$)

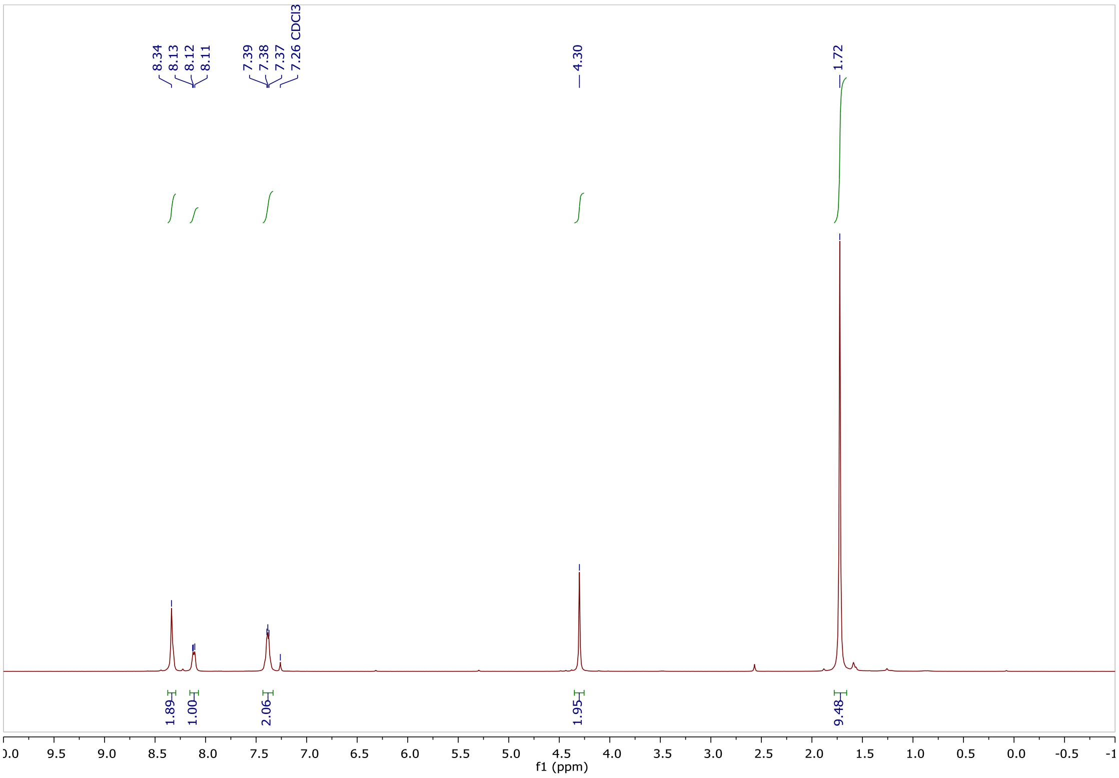

^{13}C NMR (101 MHz, $CDCl_3$)

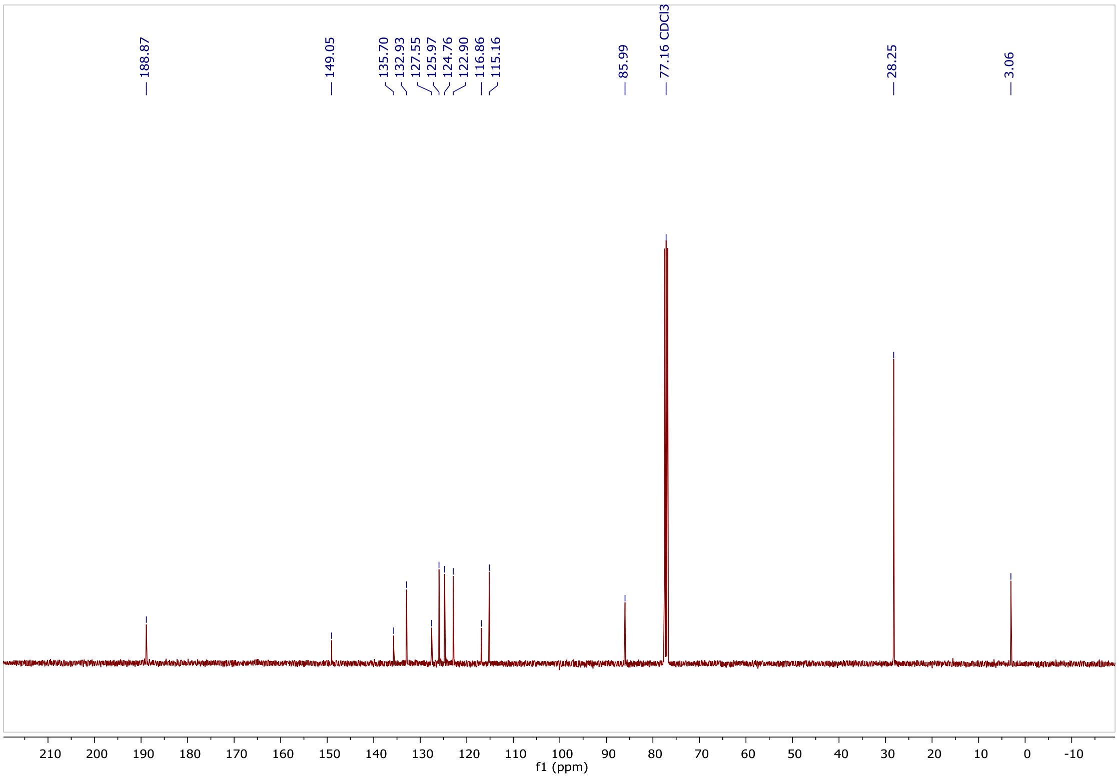

2-Iodopropanamide, 7l

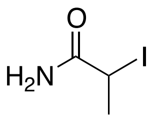

^{1}H NMR (400 MHz, CD_3OD)

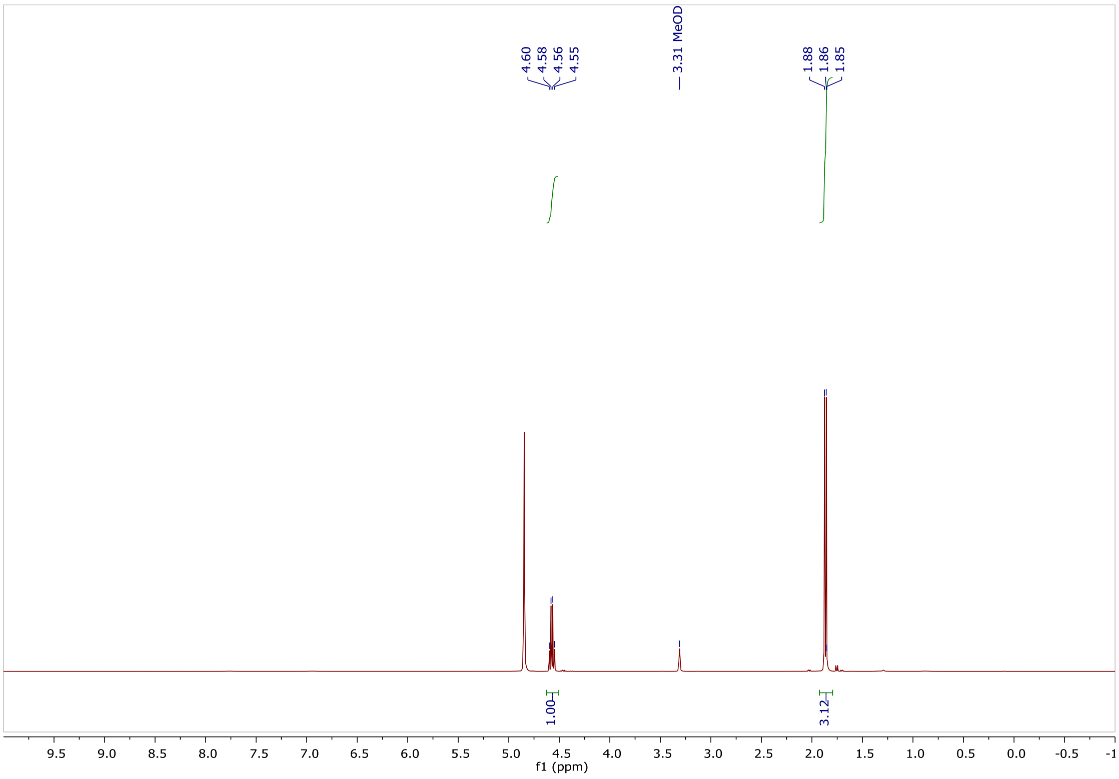

^{13}C NMR (101 MHz, CD_3OD)

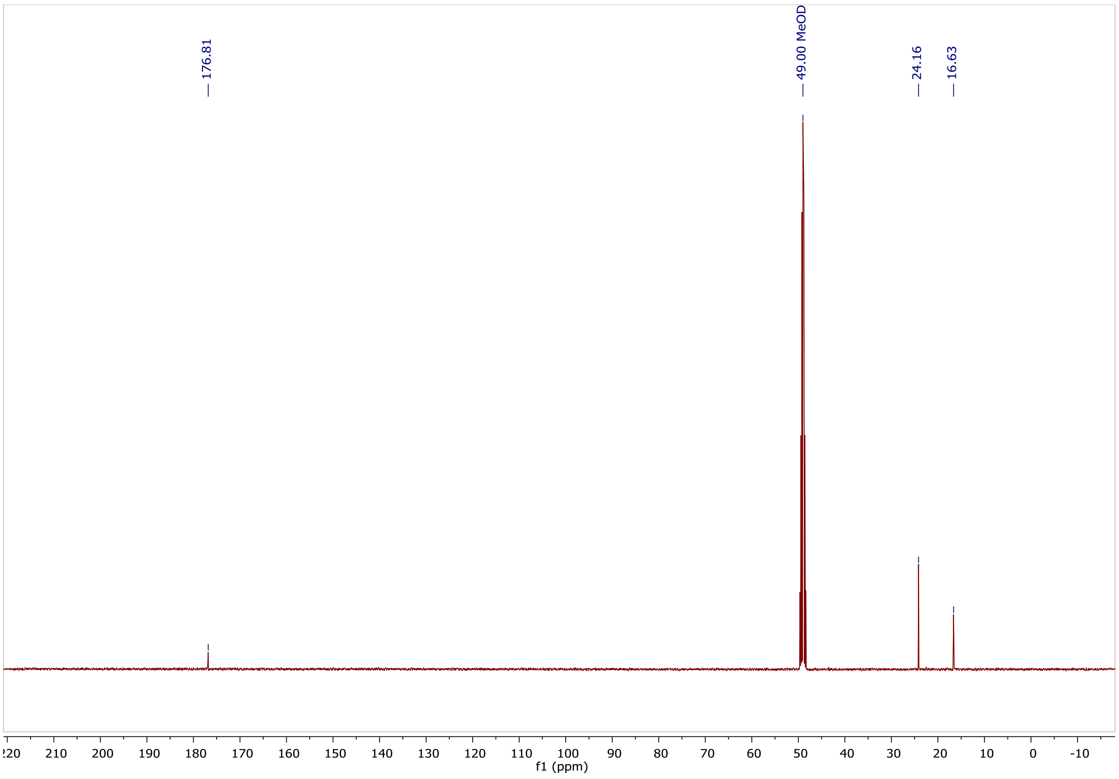

((Iodomethyl)sulfonyl)benzene, 7n

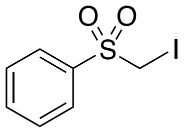

^{1}H NMR (400 MHz, $CDCl_3$)

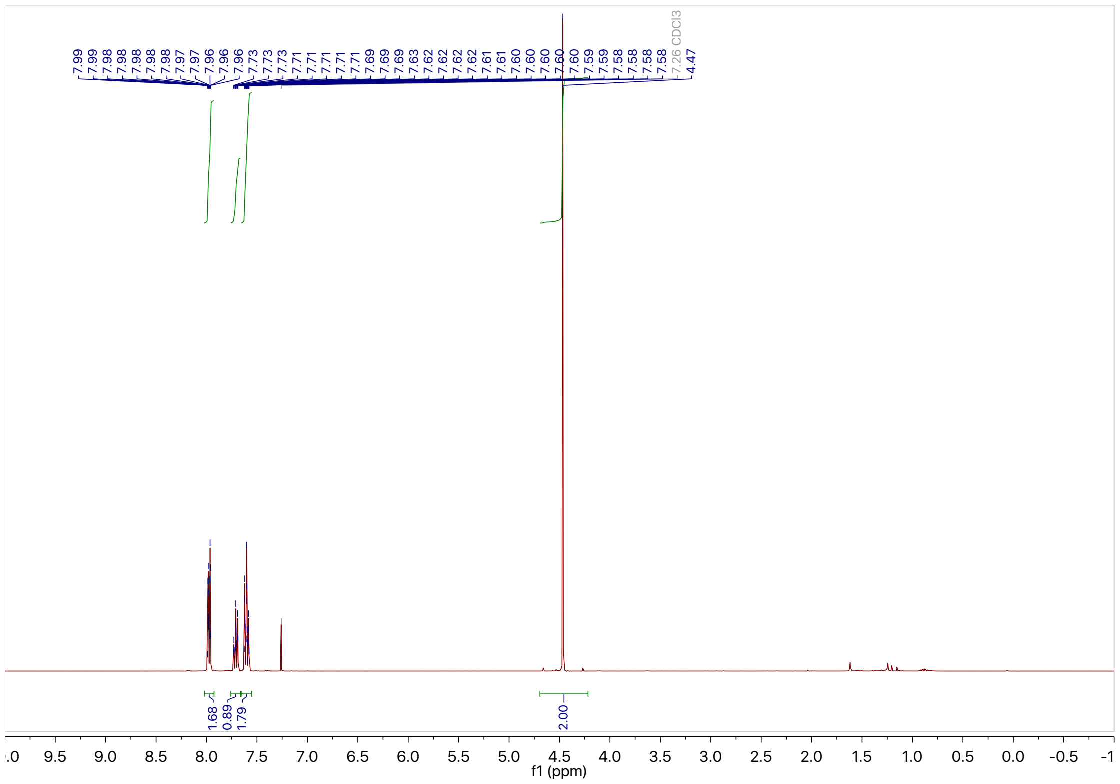

^{13}C NMR (101 MHz, $CDCl_3$)

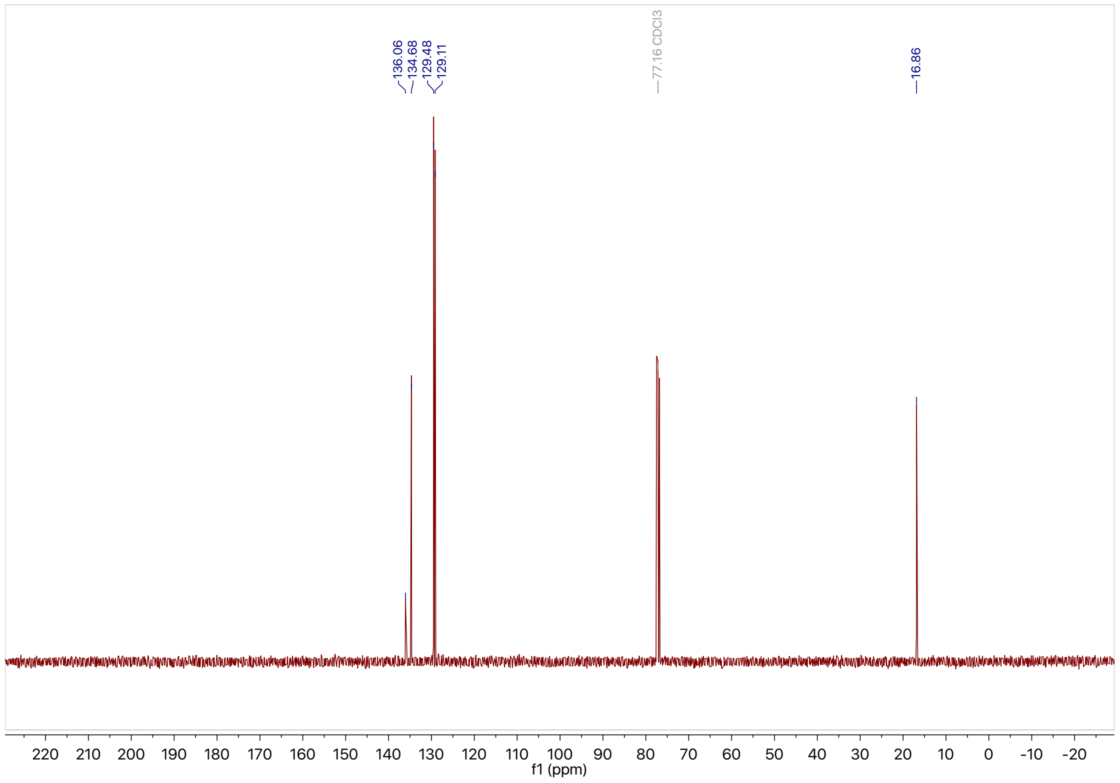

2-Iodo-3-phenylpropanal, 7o

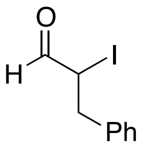

^{1}H NMR (400 MHz, $CDCl_3$)

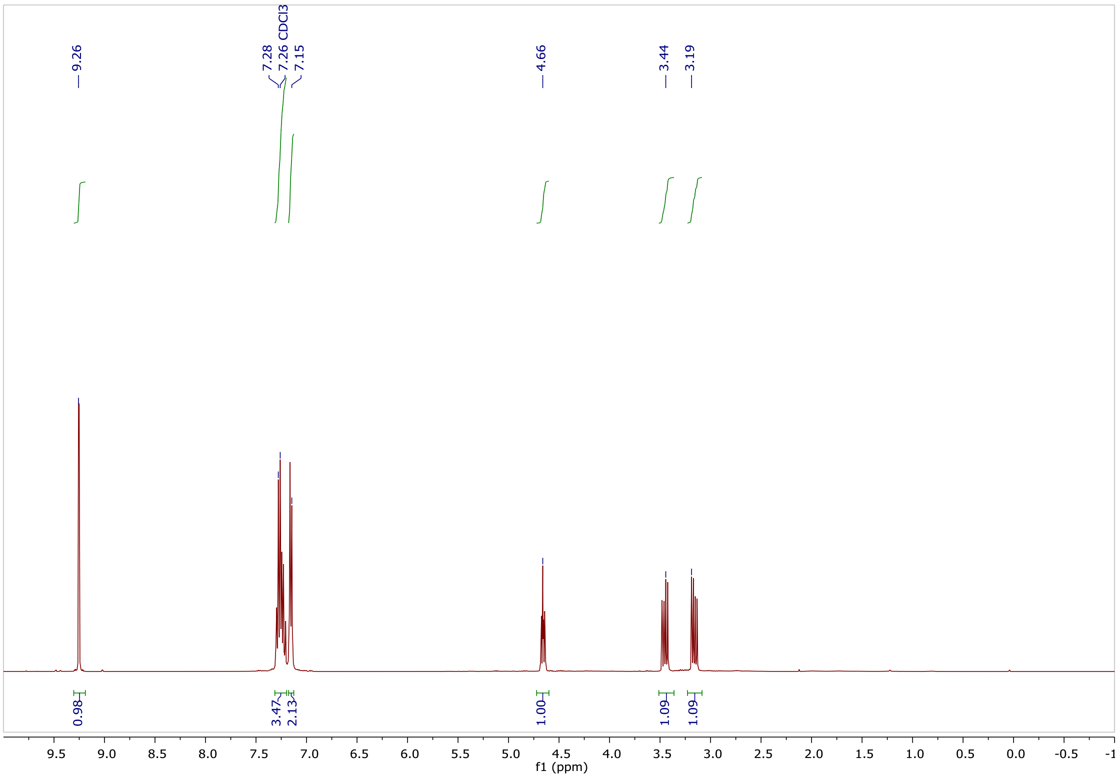

^{13}C NMR (101 MHz, $CDCl_3$)

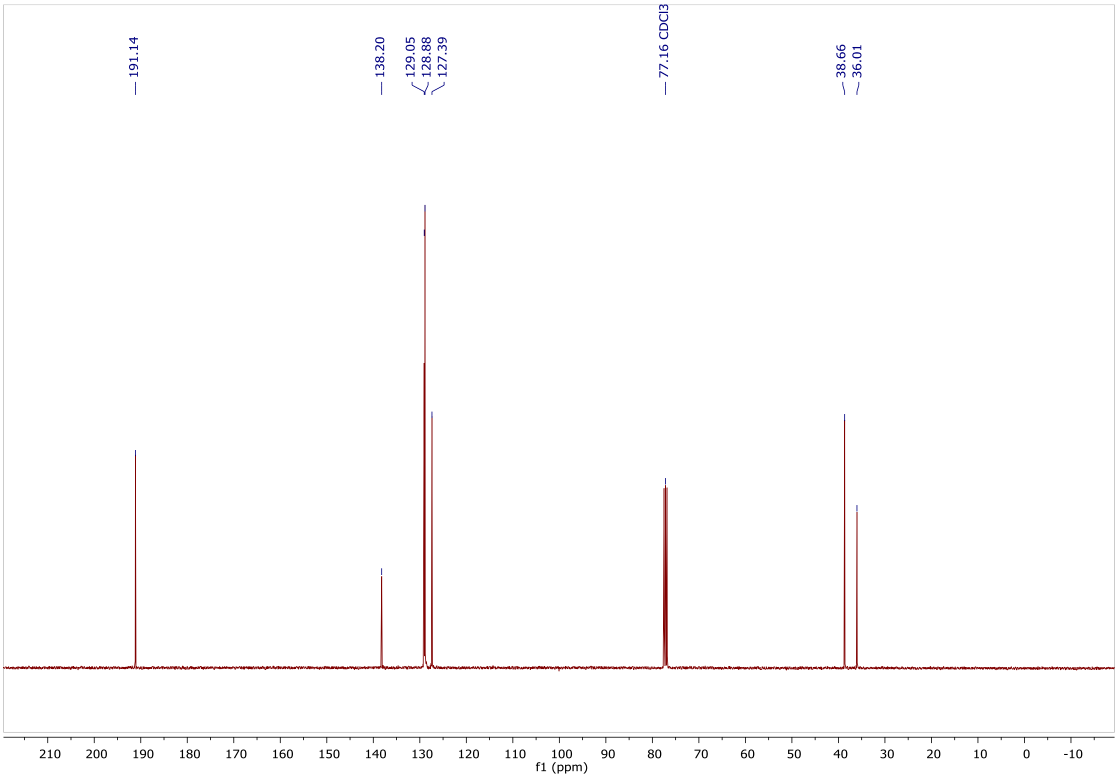

Methyl (*R*)-2-((tert-butoxycarbonyl)amino)-3-iodopropanoate, 7p

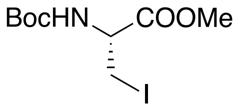

^{1}H NMR (400 MHz, $CDCl_3$)

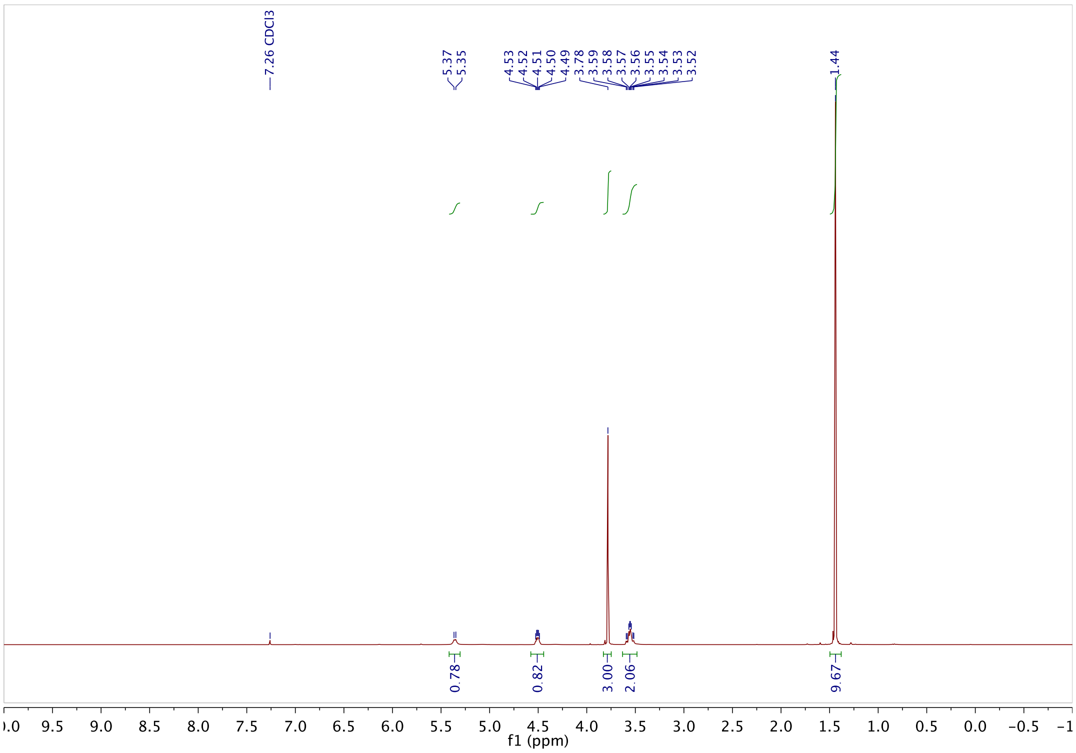

^{13}C NMR (101 MHz, $CDCl_3$)

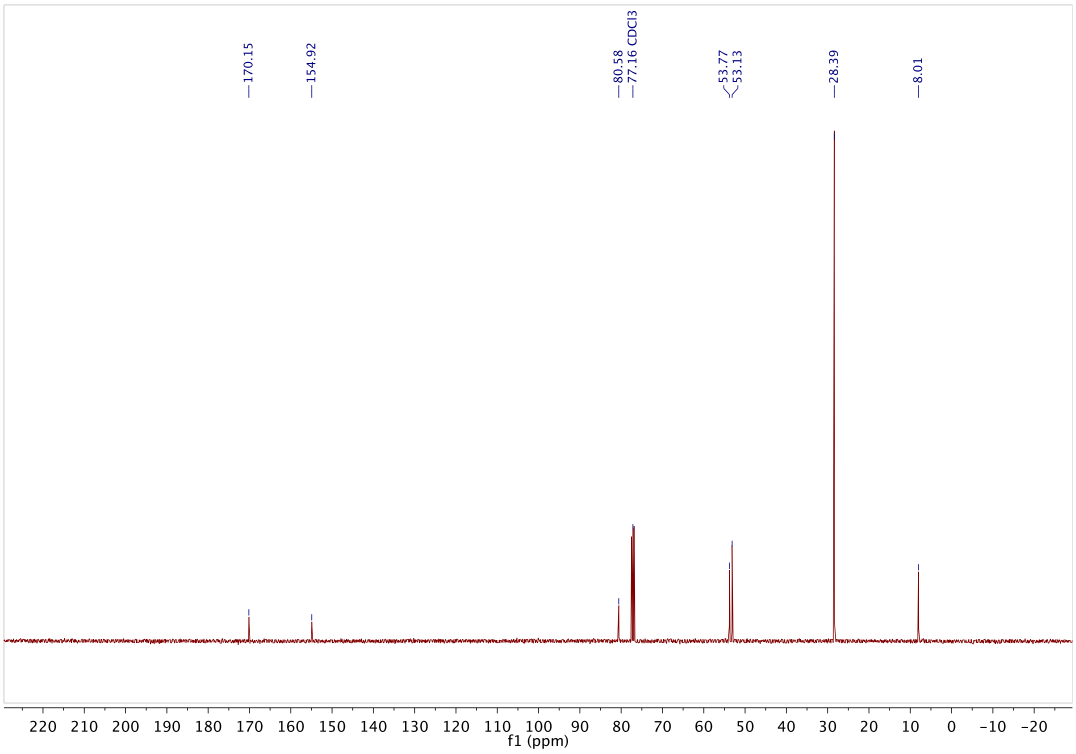

Methyl (*R*)-2-((tert-butoxycarbonyl)amino)-3-iodopropanoate, 7p□

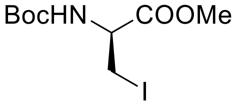

^{1}H NMR (400 MHz, $CDCl_3$)

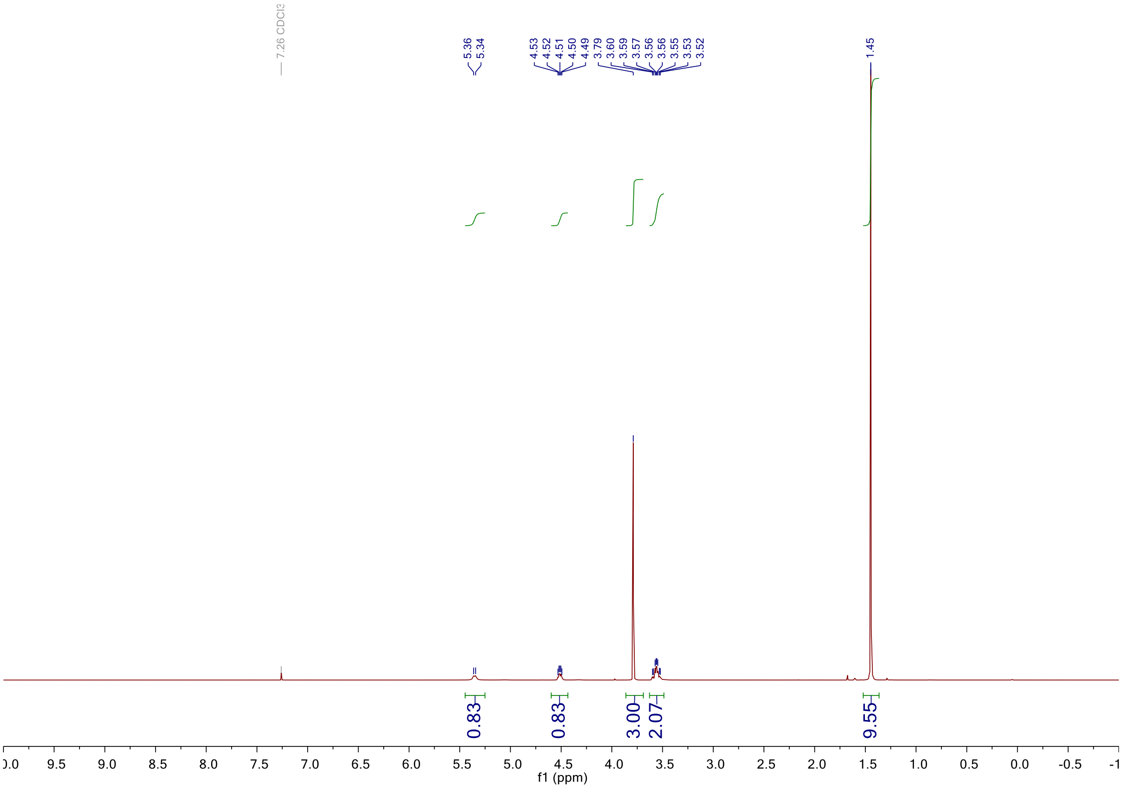

^{13}C NMR (101 MHz, $CDCl_3$)

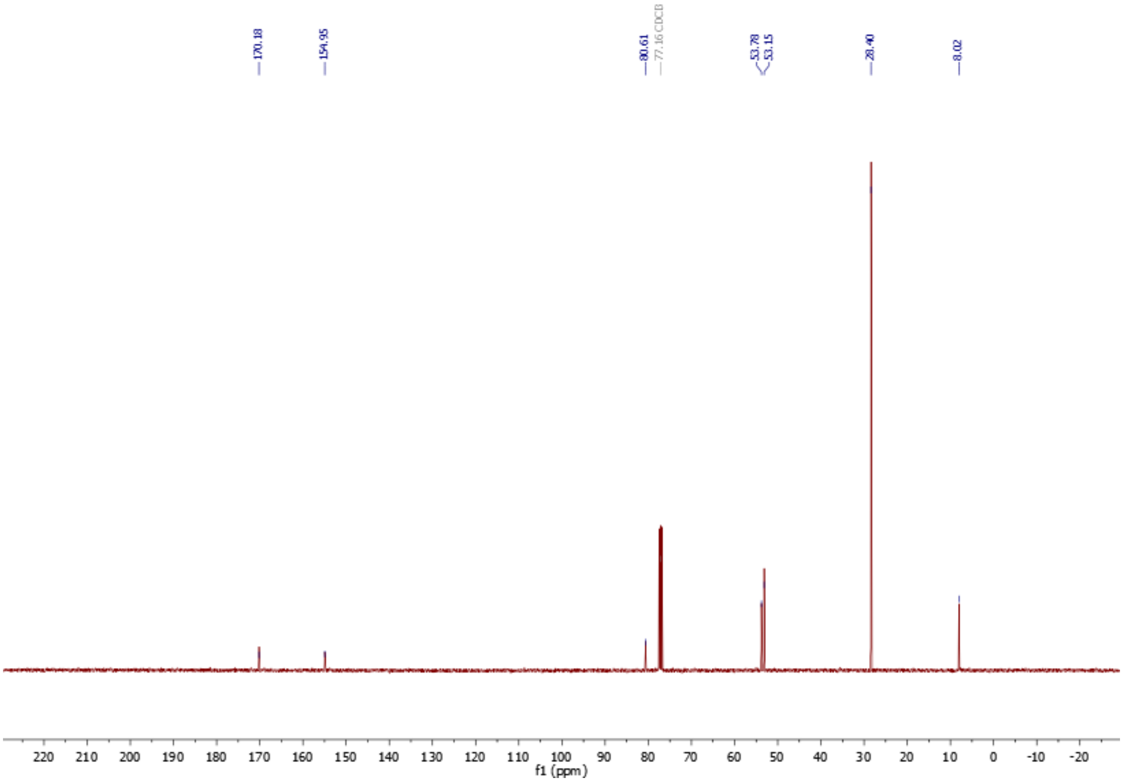

4-Nitrobenzyl iodide, 7r

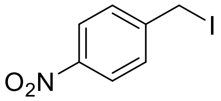

^{1}H NMR (400 MHz, $CDCl_3$)

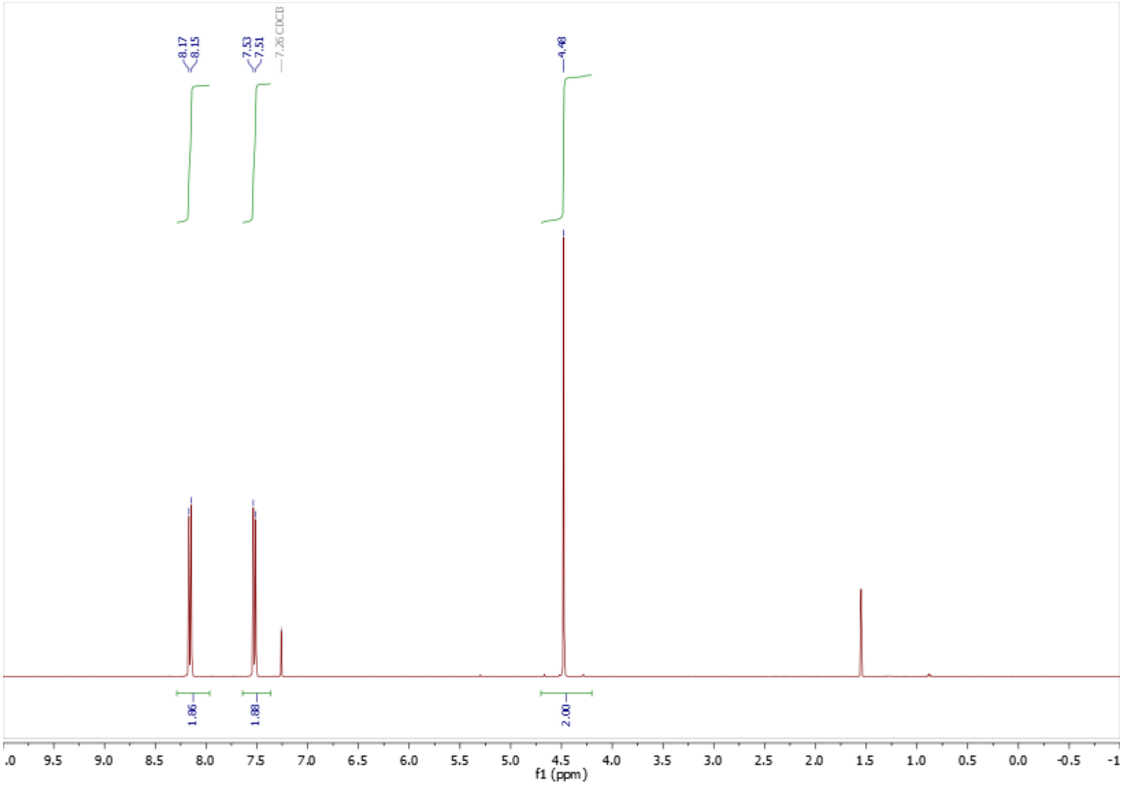

^{13}C NMR (101 MHz, $CDCl_3$)

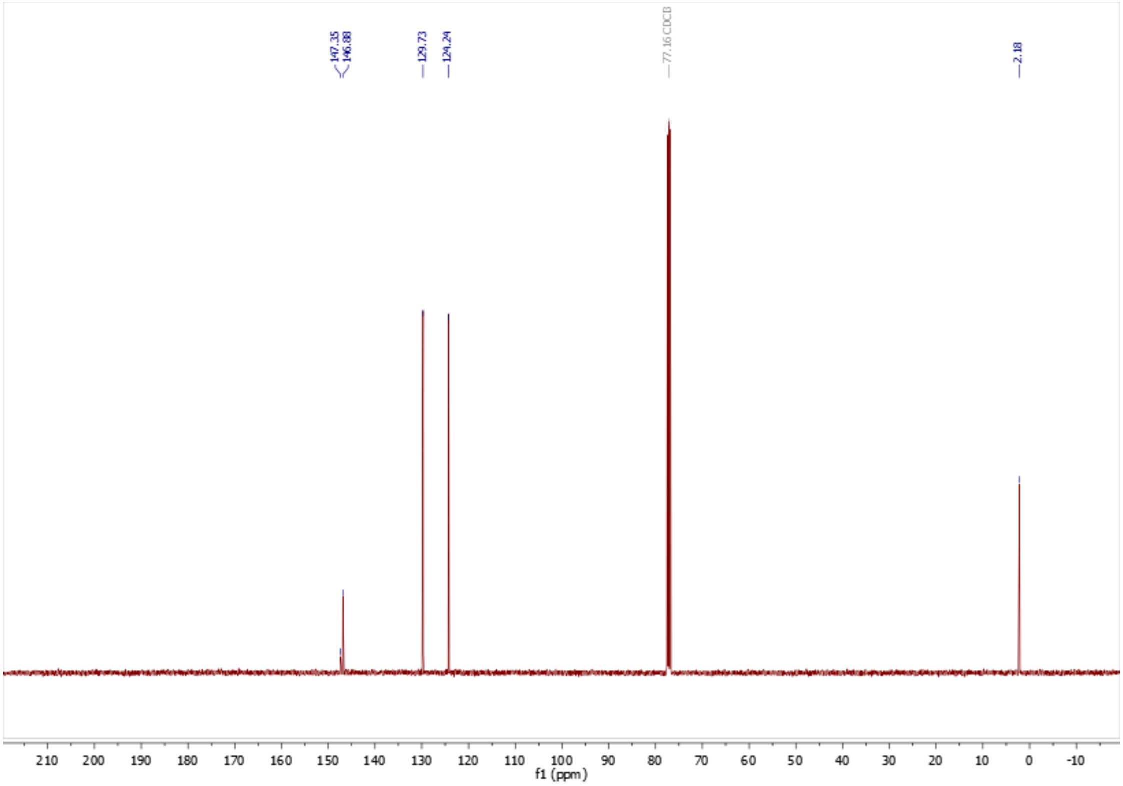

1-(Iodomethyl)-4-(trifluoromethyl)benzene, 7s

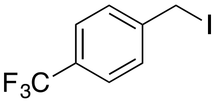

¹H NMR (400 MHz, $CDCl_3$)

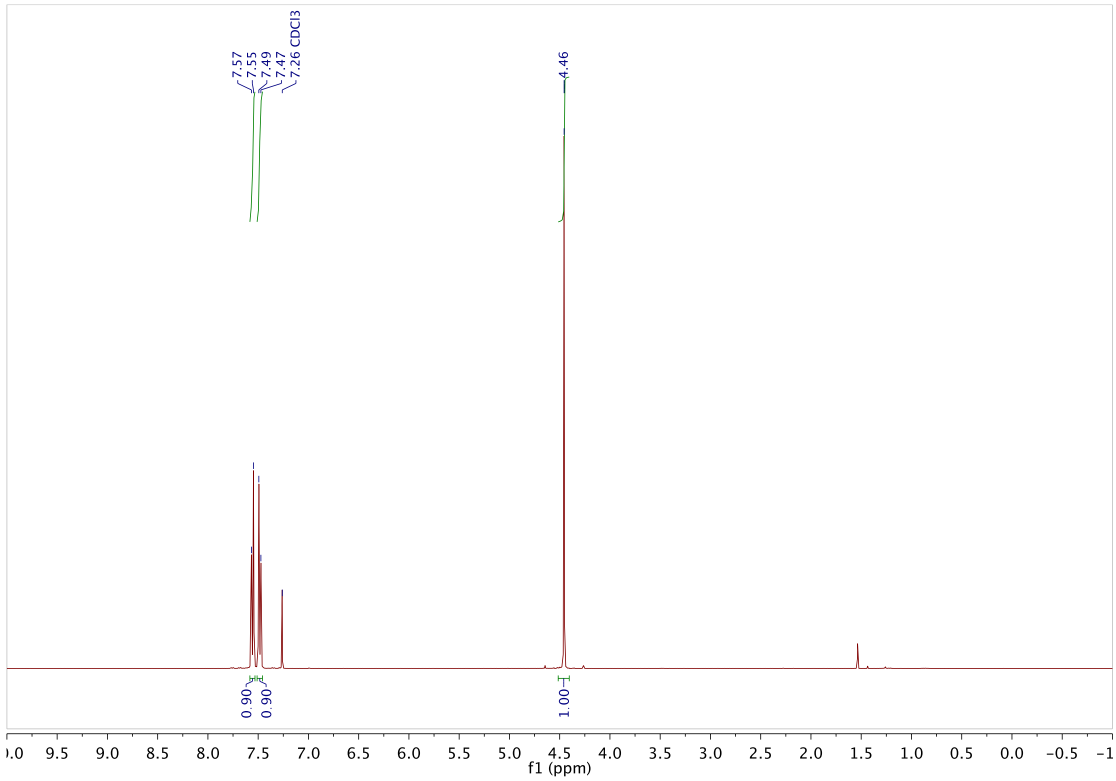

¹³C NMR (101 MHz, $CDCl_3$)

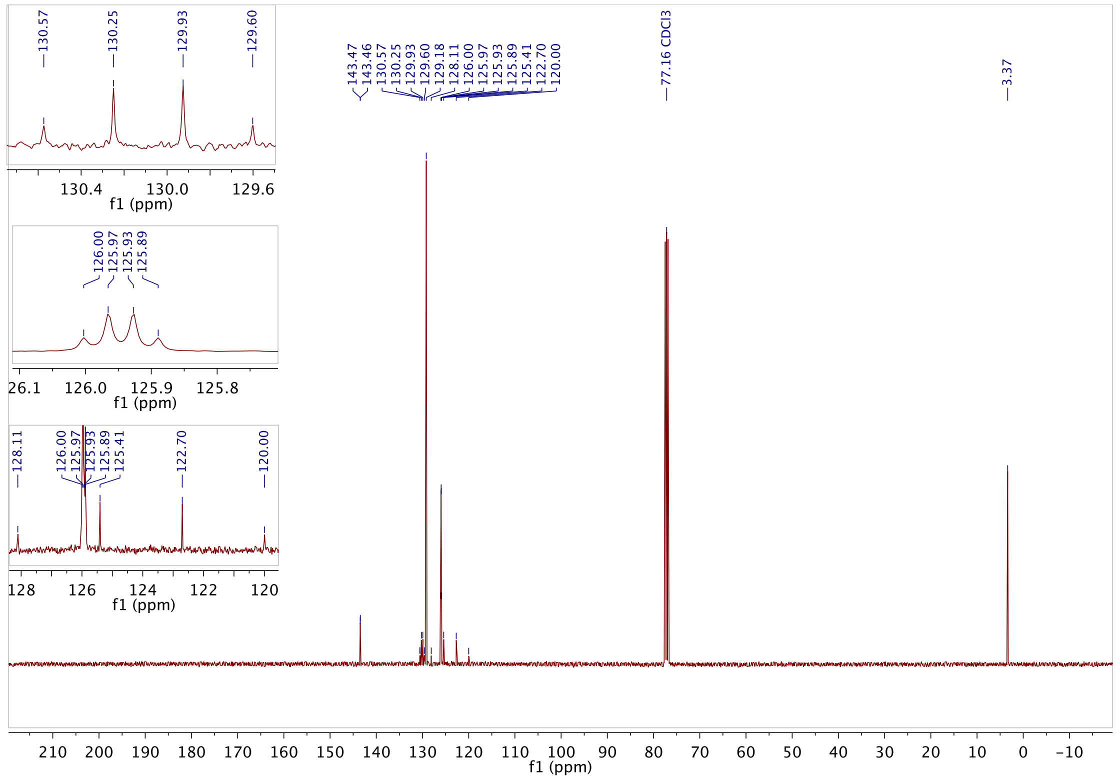

^{19}**F NMR** (377 MHz, $CDCl_3$)

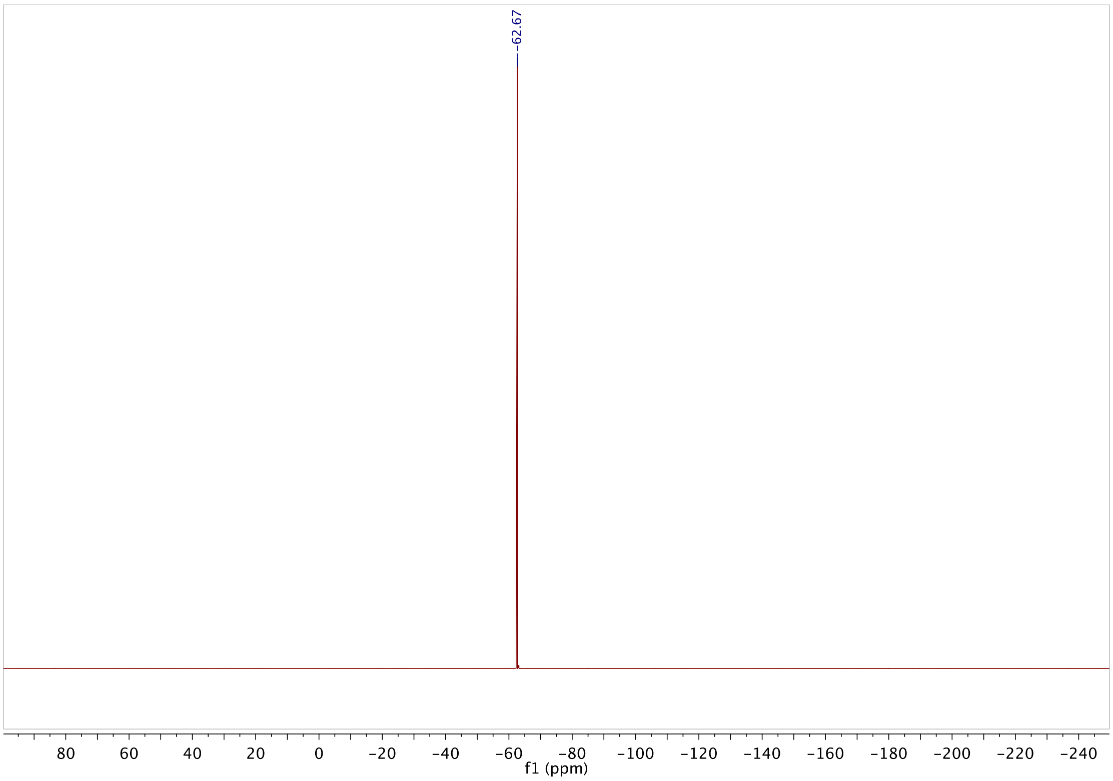

1-(Iodomethyl)-3,5-bis(trifluoromethyl)benzene, 7t

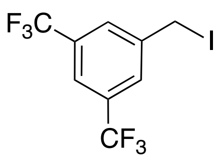

^{1}H NMR (400 MHz, CDCl$_3$)

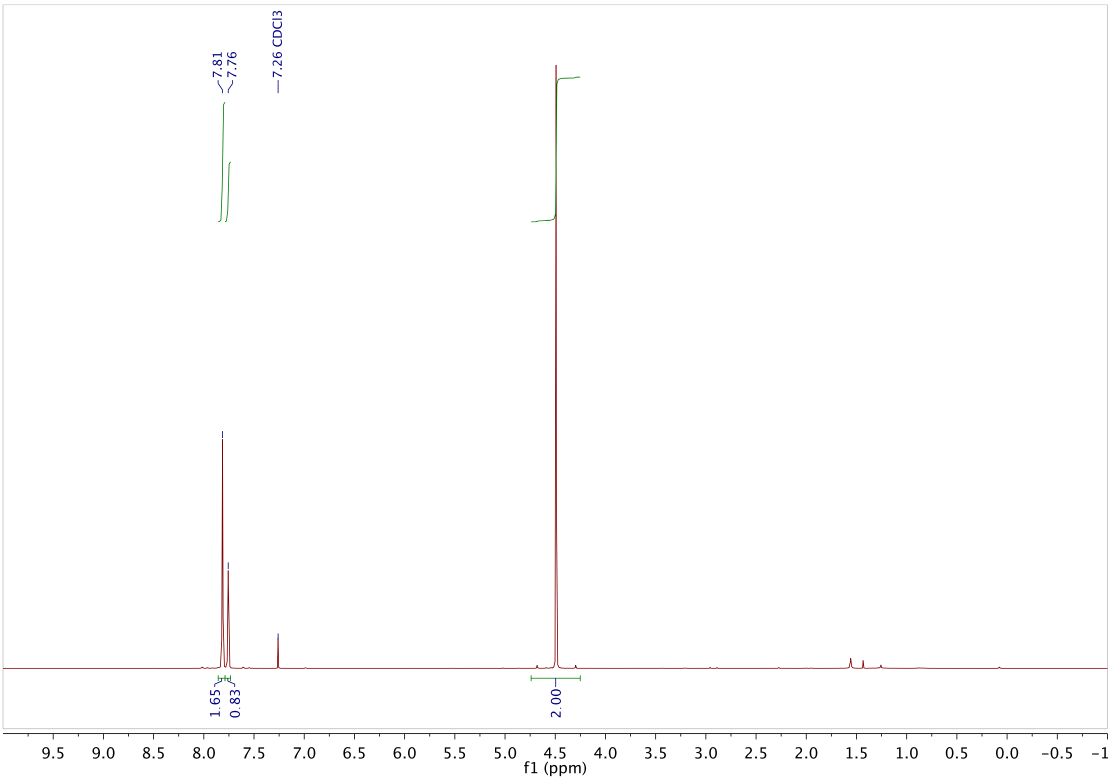

^{13}C NMR (101 MHz, CDCl$_3$)

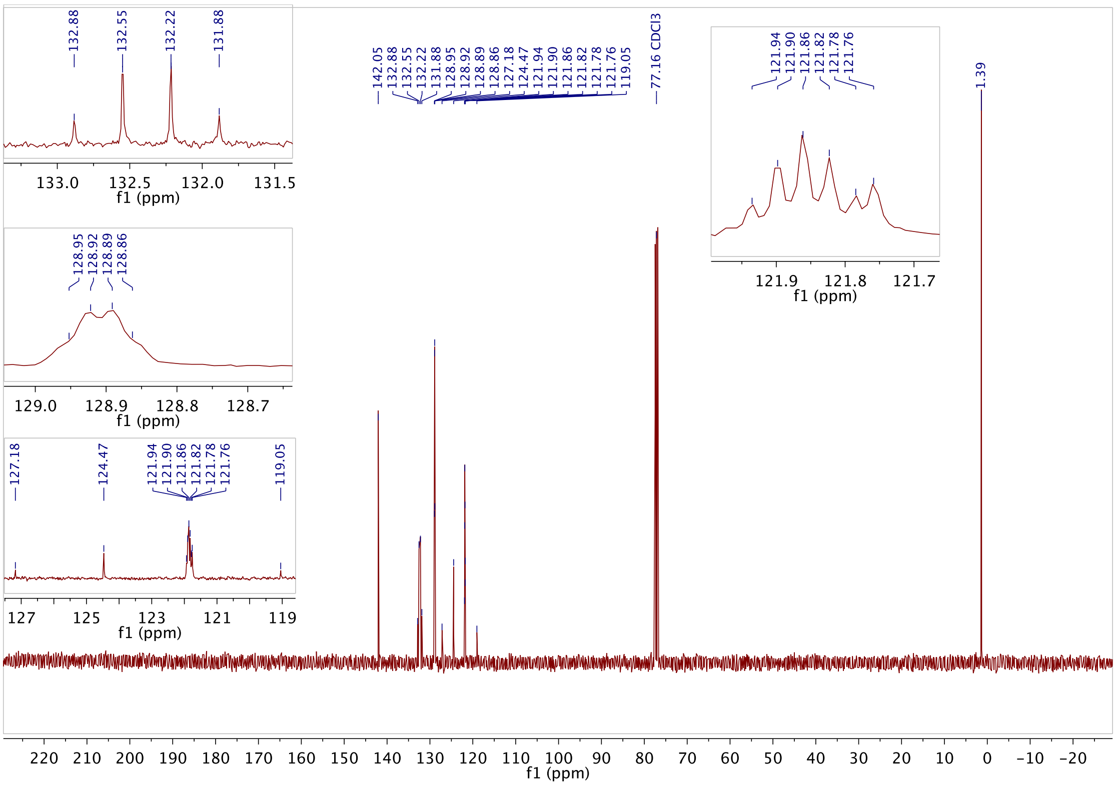

^{19}F NMR (377 MHz, $CDCl_3$)

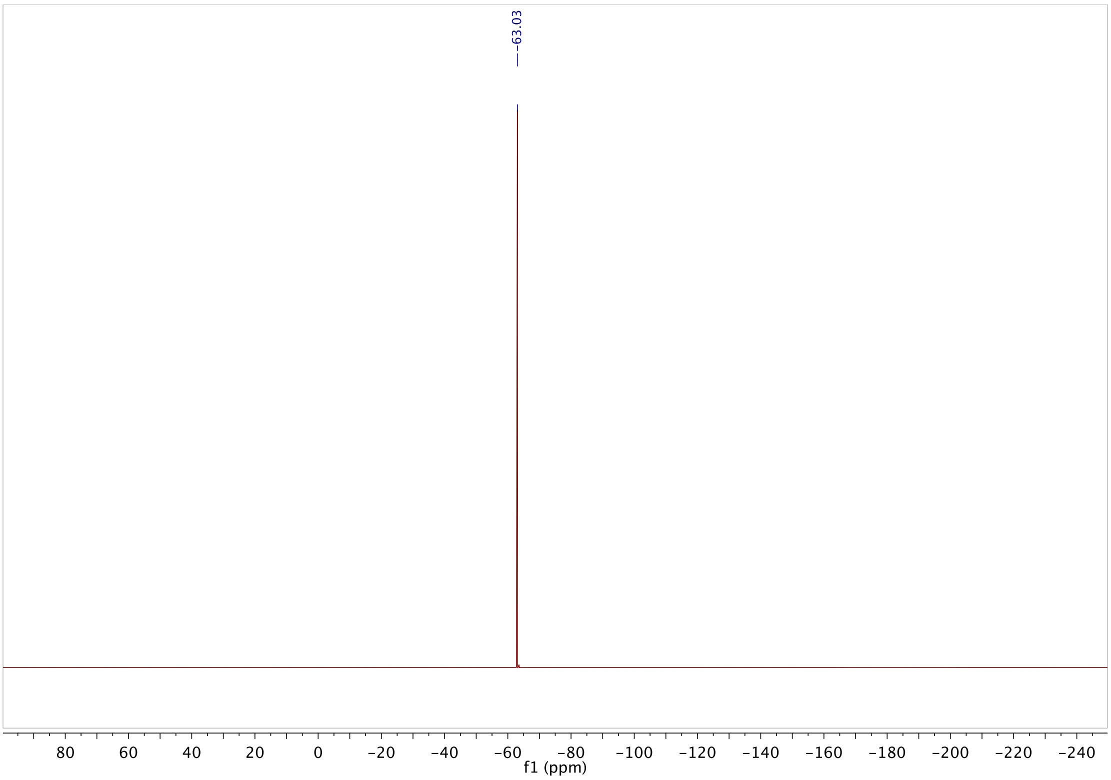

2-(Iodomethyl)benzonitrile, 7u

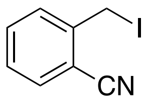

^{1}H NMR (400 MHz, $CDCl_3$)

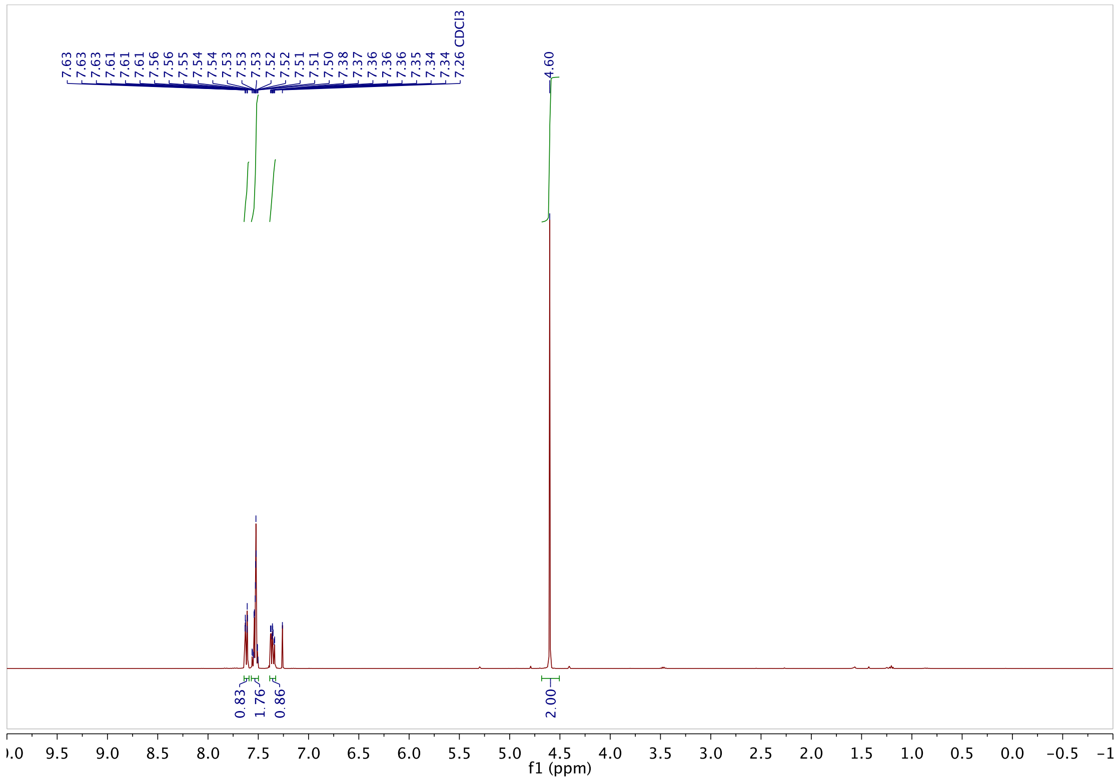

^{13}C NMR (101 MHz, $CDCl_3$)

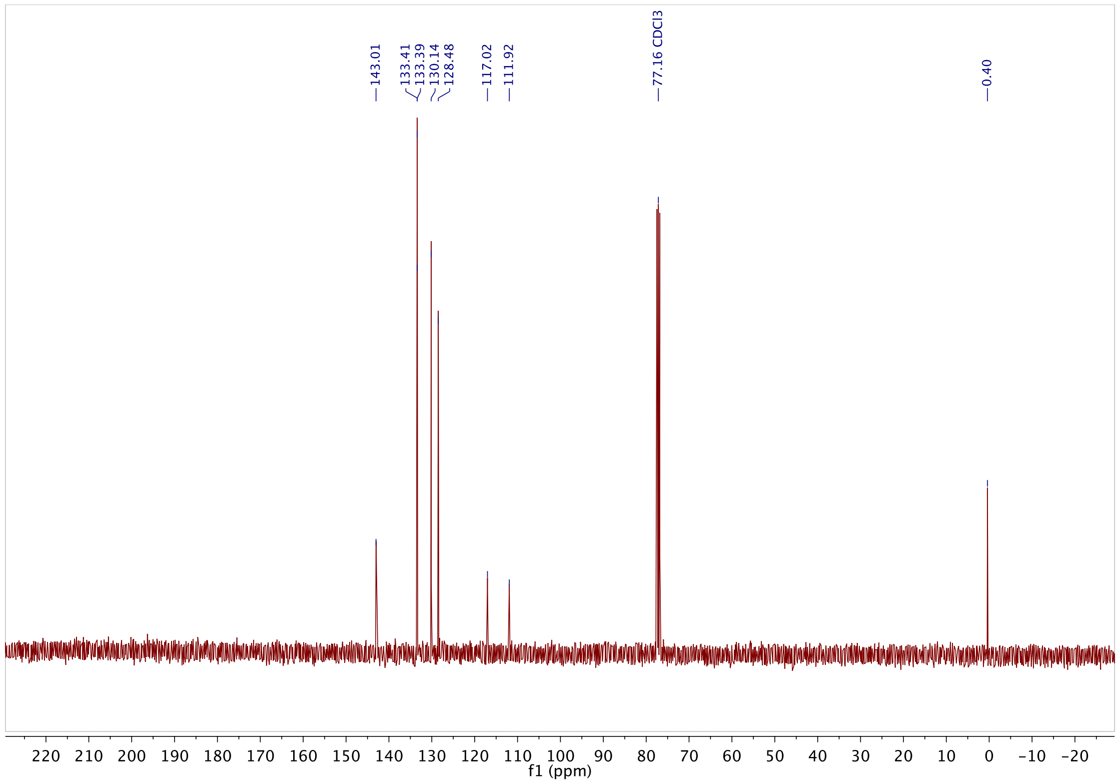

(Iodomethyl)cyclopropane, 7v

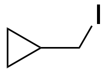

^{1}H NMR (400 MHz, $CDCl_3$)

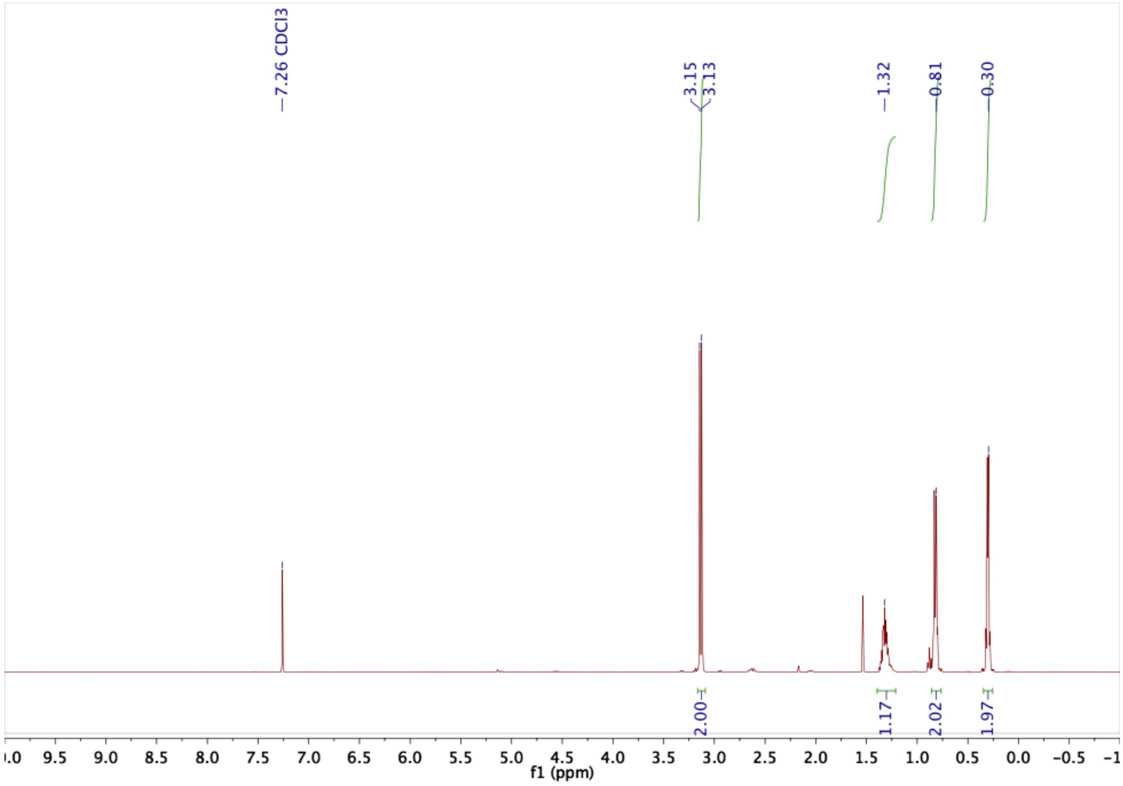

^{13}C NMR (101 MHz, $CDCl_3$)

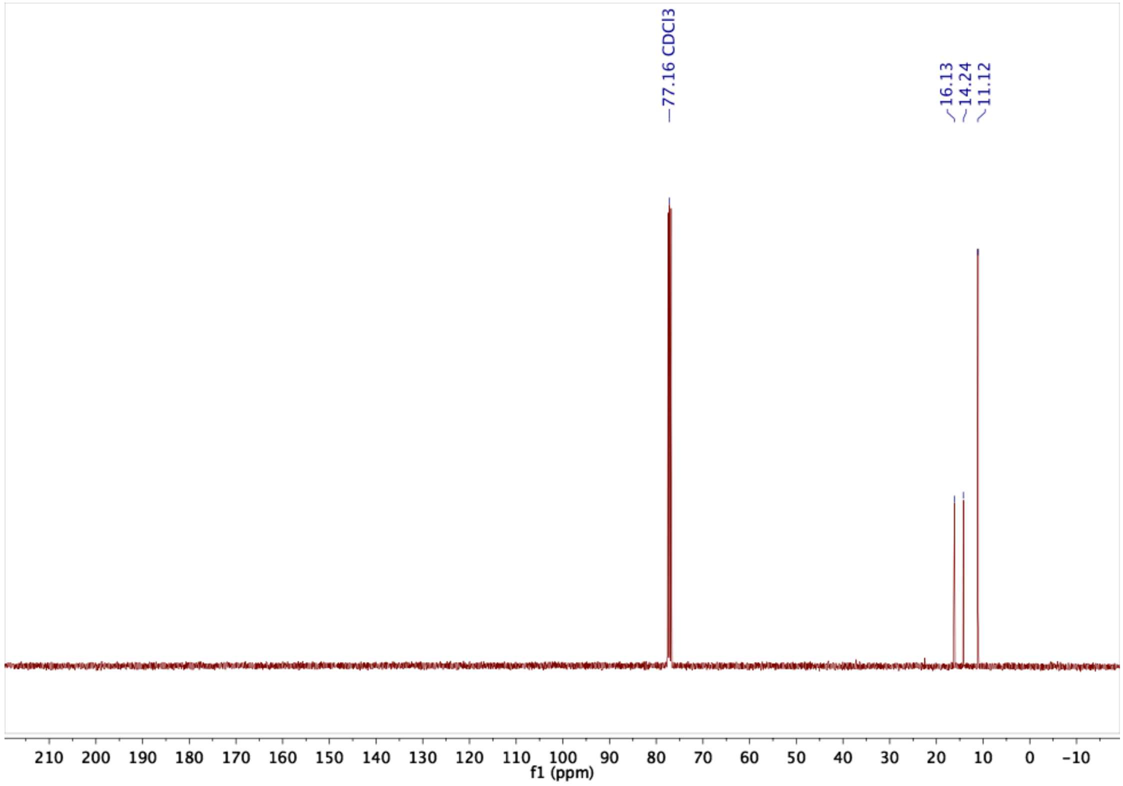

Dimethyl bromomalonate, 8c

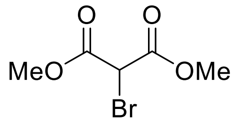

^{1}H NMR (400 MHz, $CDCl_3$)

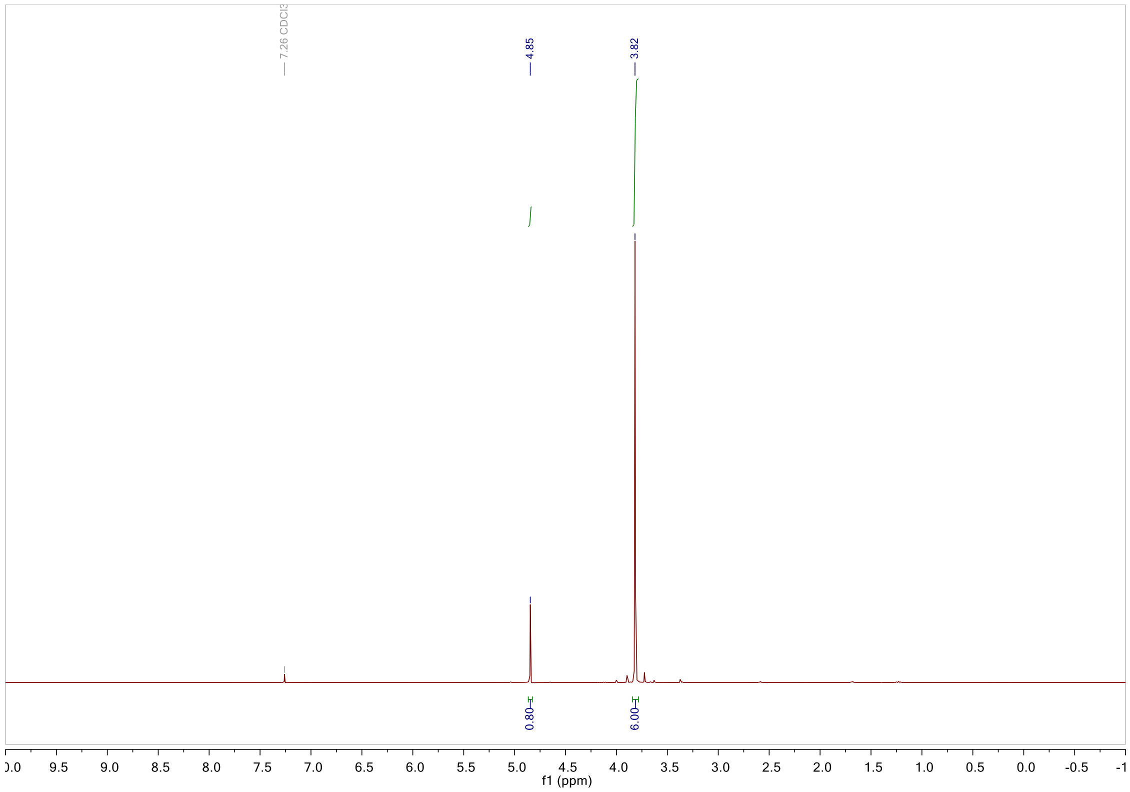

^{13}C NMR (101 MHz, $CDCl_3$)

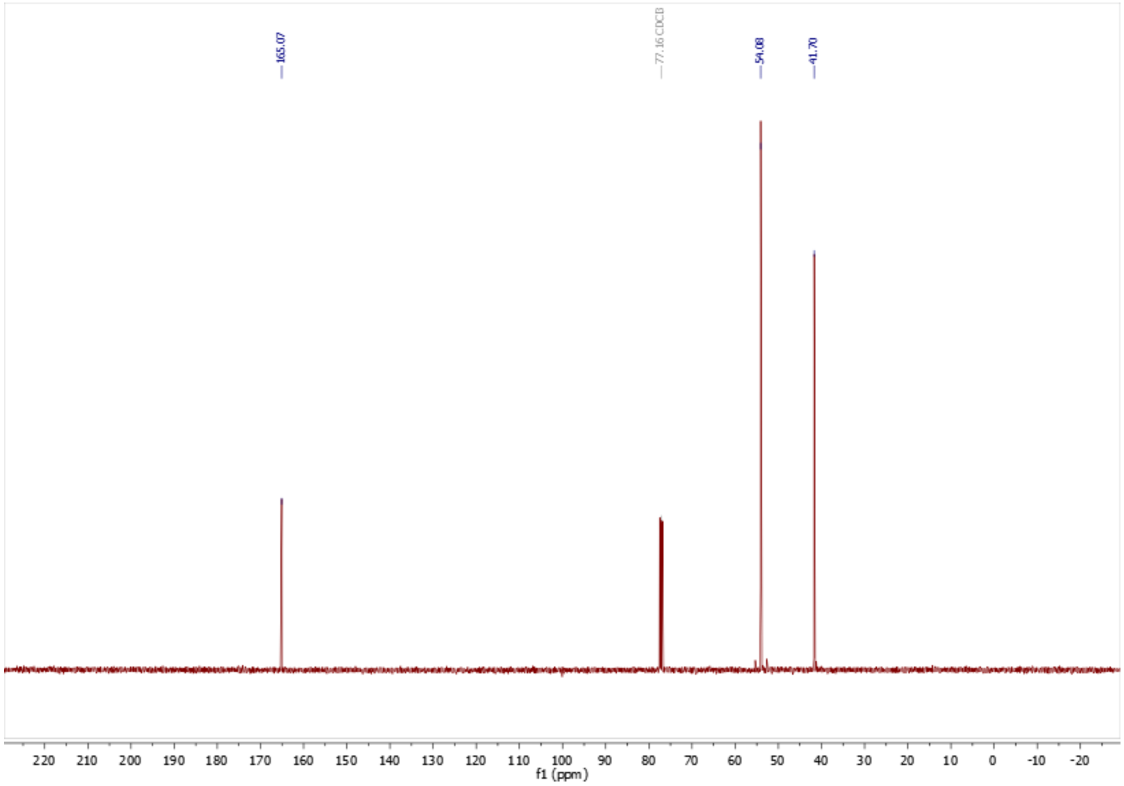

Diethyl 2-bromo-2-methylmalonate, 8d

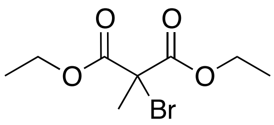

^{1}H NMR (400 MHz, $CDCl_3$)

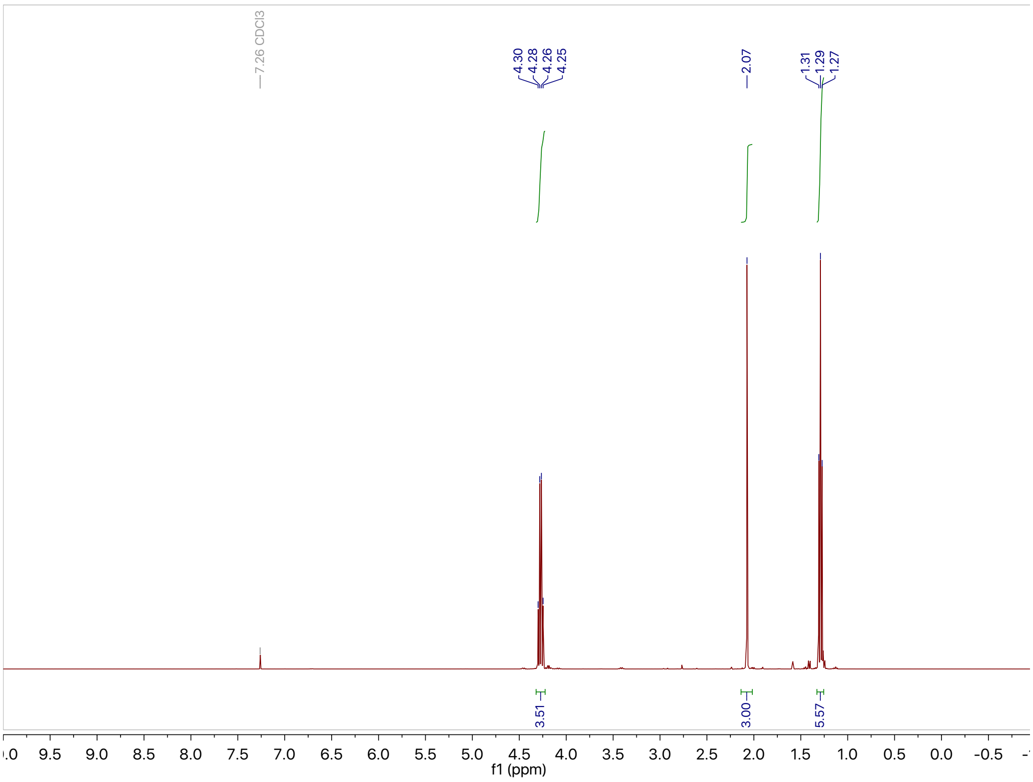

^{13}C NMR (101 MHz, $CDCl_3$)

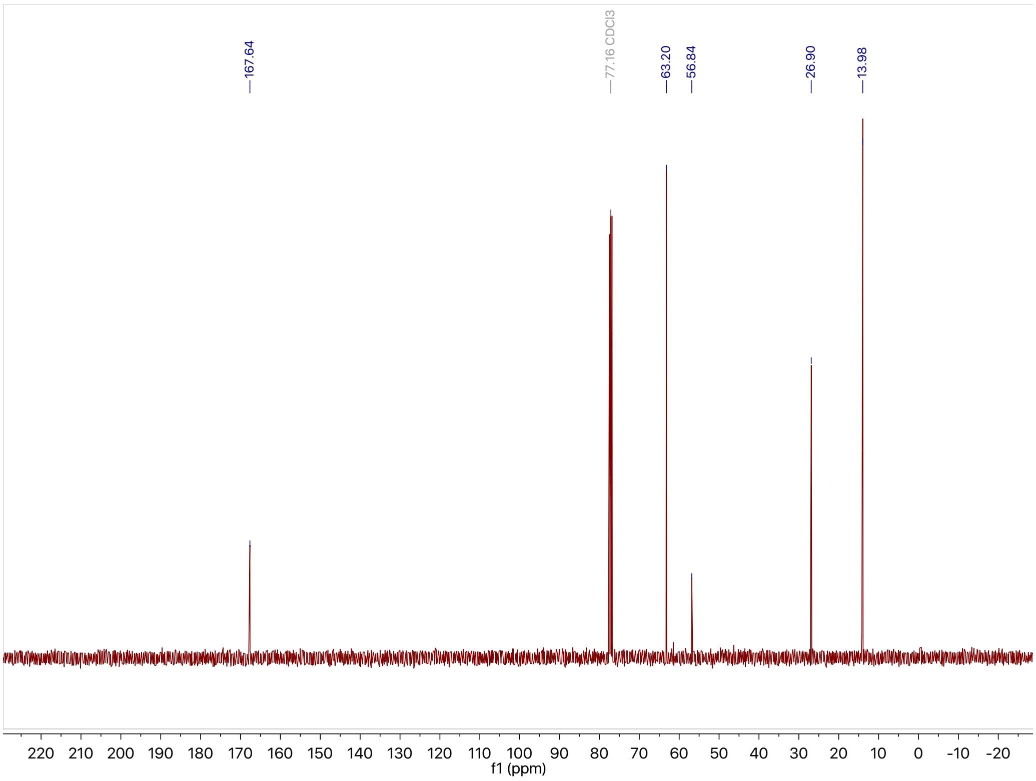

2-bromo-1,3-diphenylpropane-1,3-dione, 8e

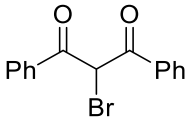

[1]**H NMR** (400 MHz, $CDCl_3$)

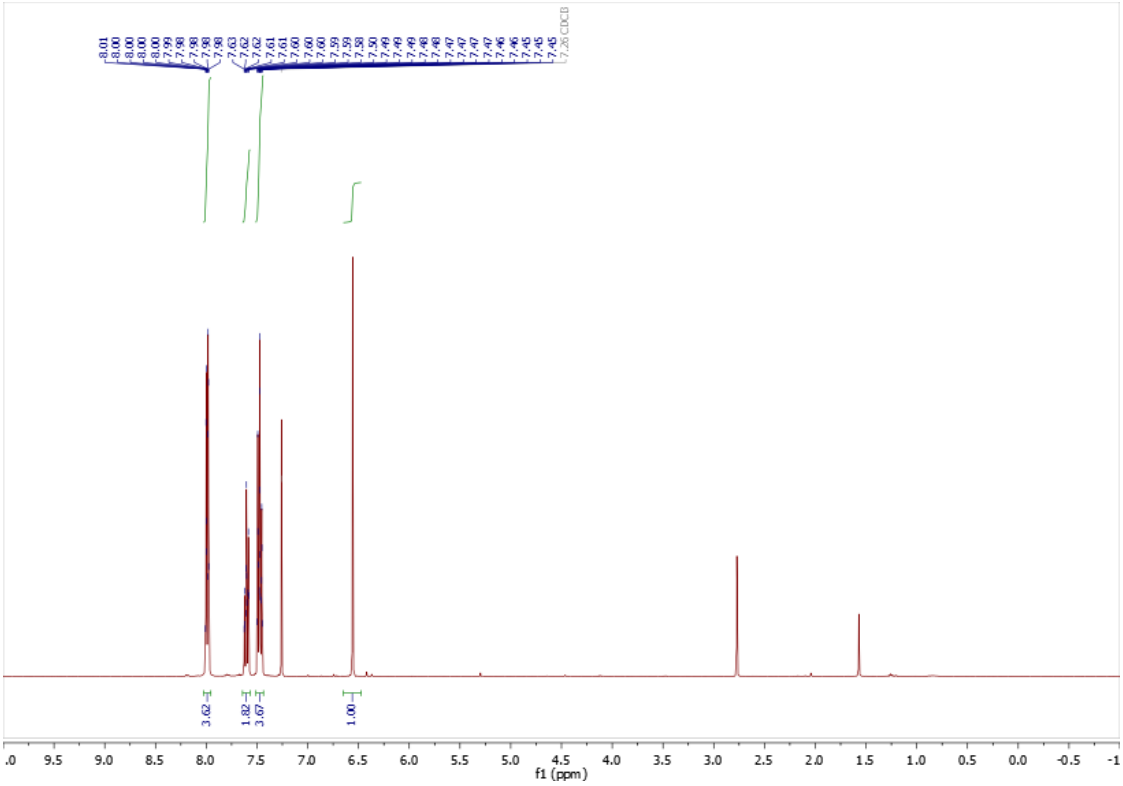

[13]**C NMR** (101 MHz, $CDCl_3$)

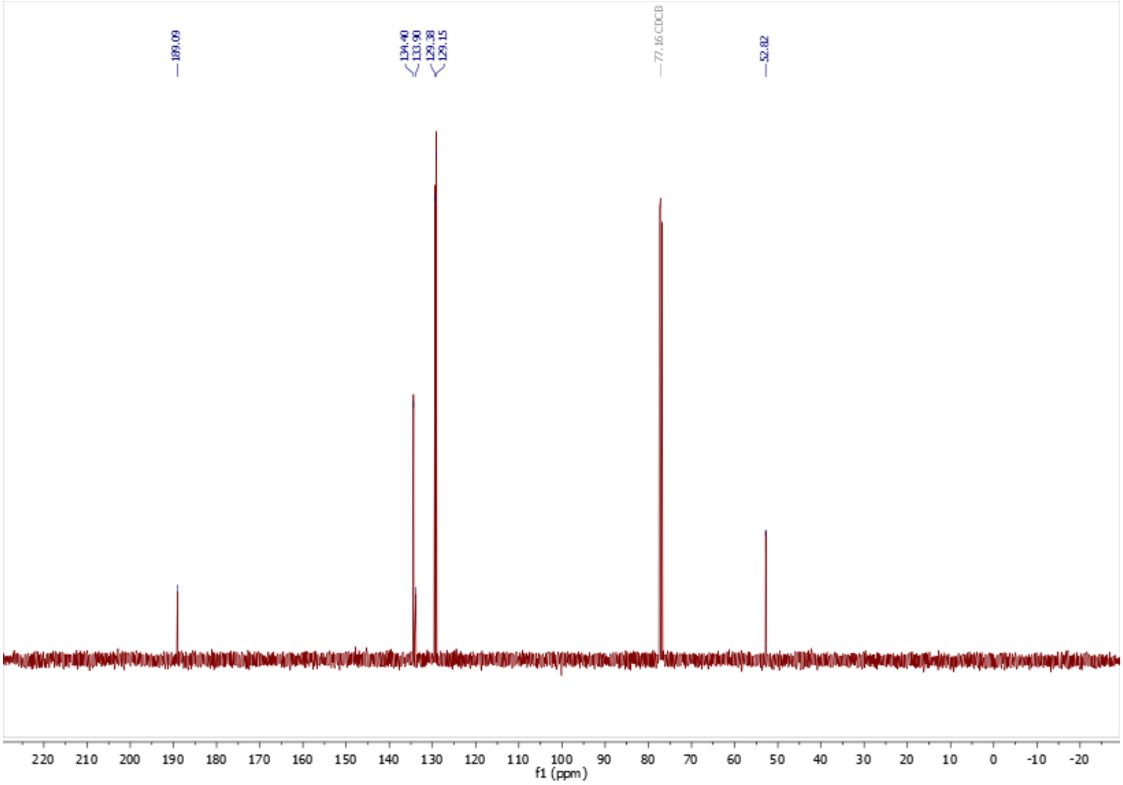

1-phenyl-2-tosylethan-1-one, S3

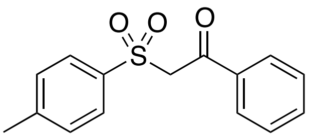

[1]**H NMR** (400 MHz, $CDCl_3$)

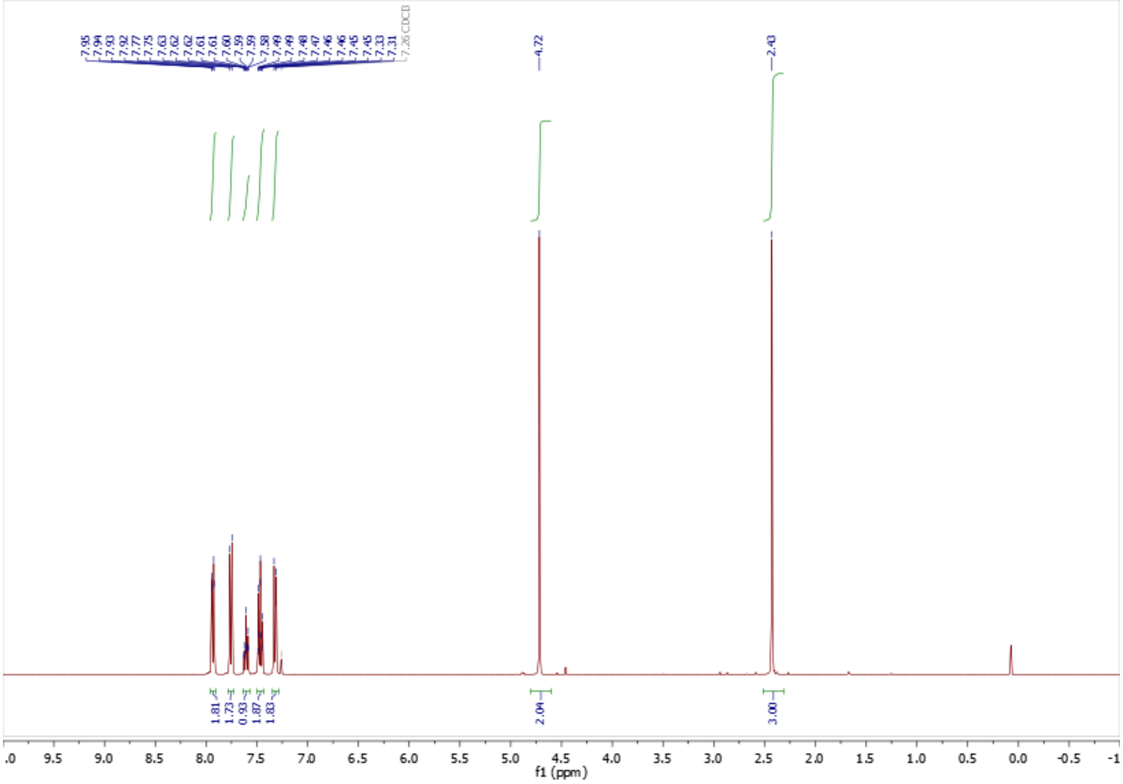

[13]**C NMR** (101 MHz, $CDCl_3$)

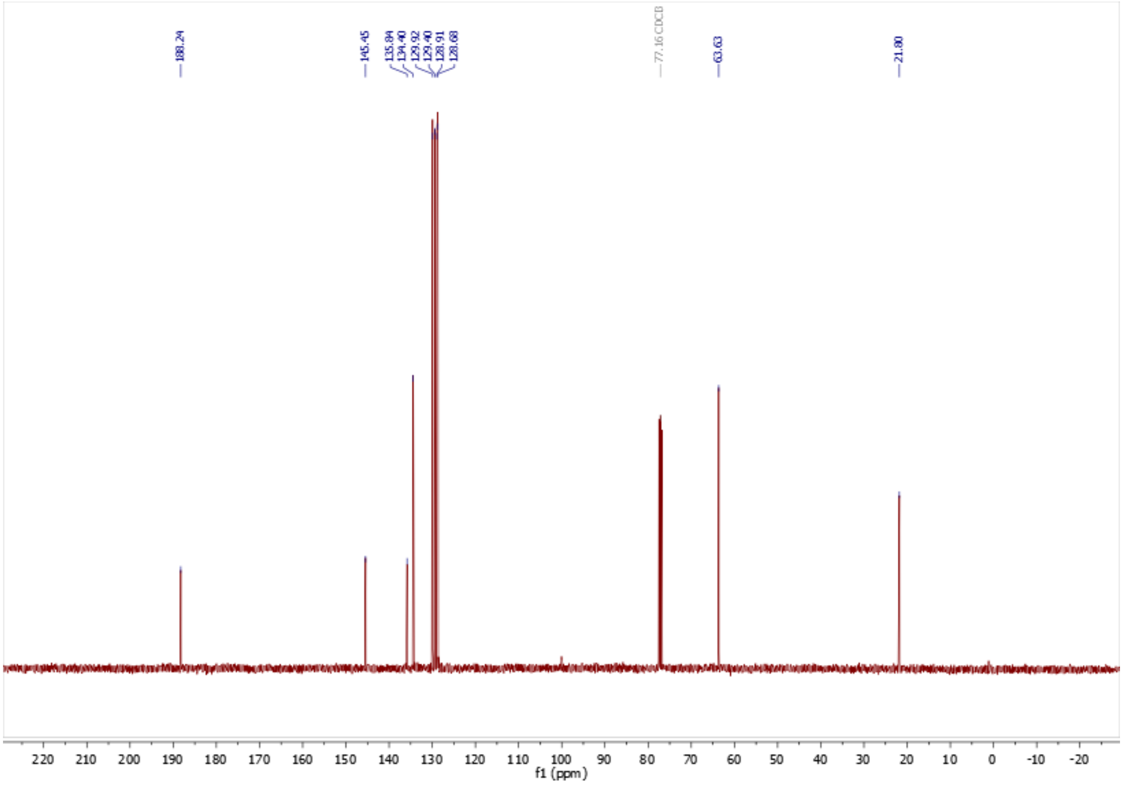

2-Bromo-1-phenyl-2-tosylethan-1-one, 8f

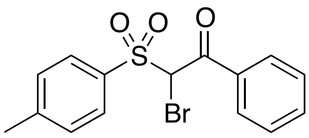

^{1}H NMR (400 MHz, $CDCl_3$)

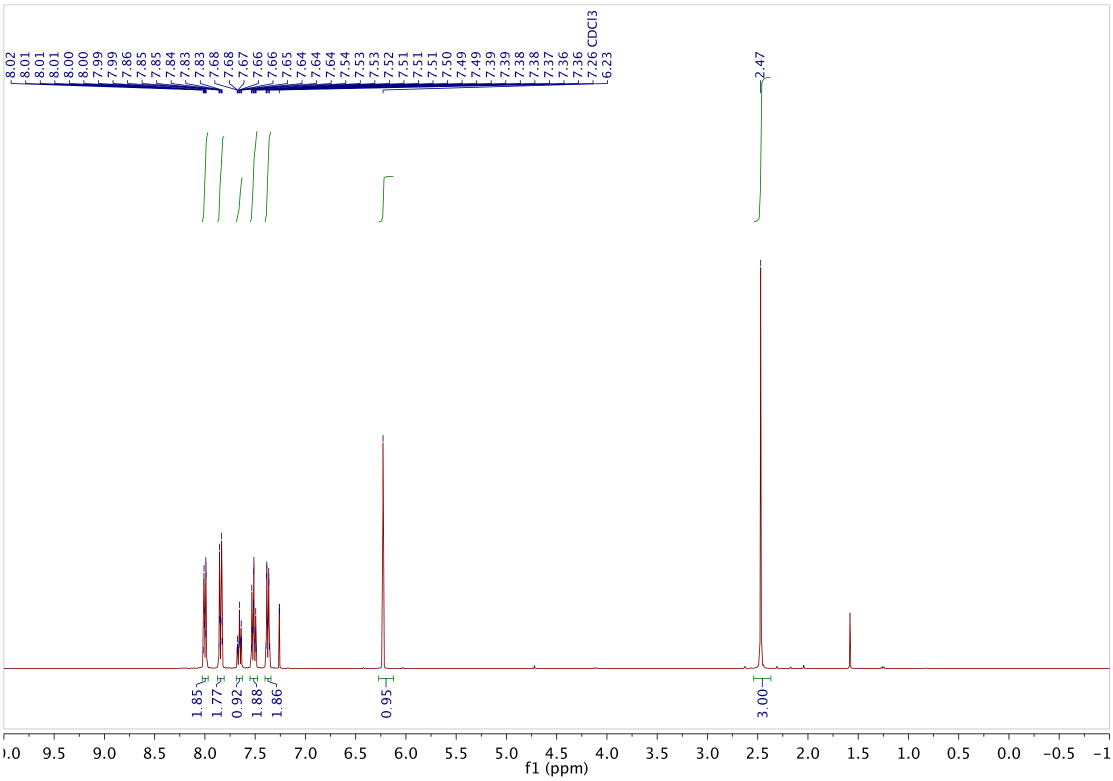

^{13}C NMR (101 MHz, $CDCl_3$)

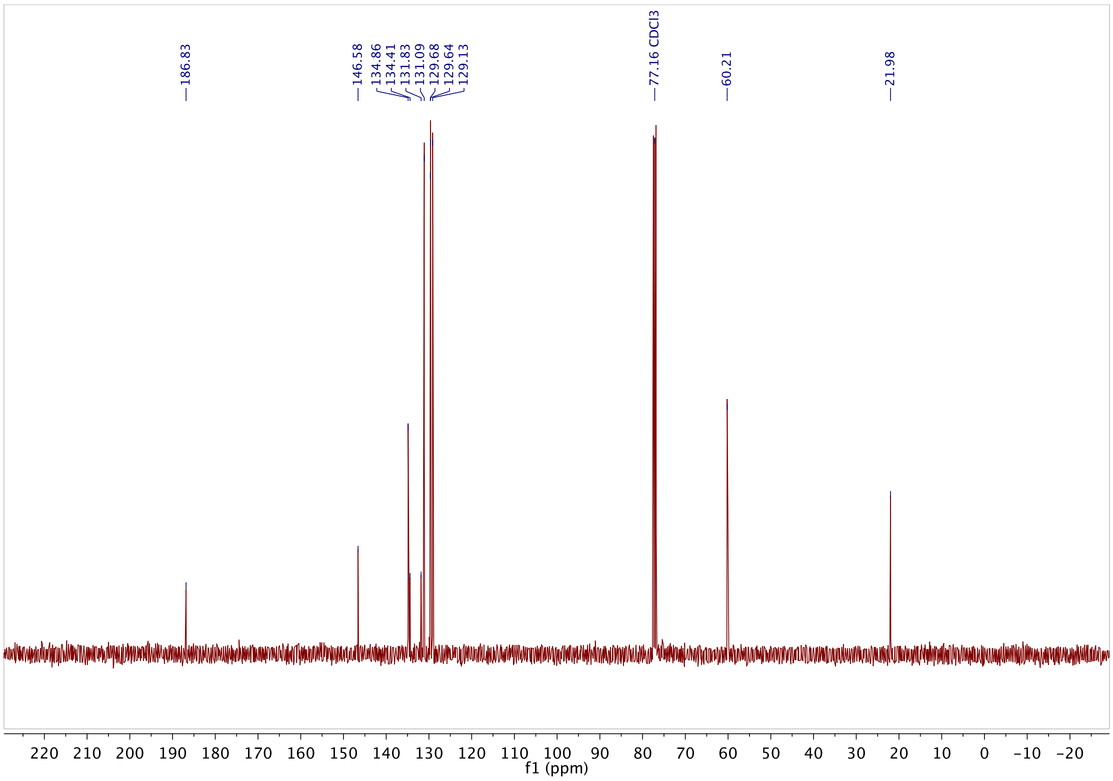

2′-iodo-2′-deoxyuridine, S4

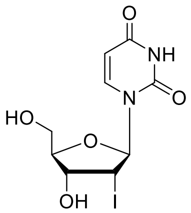

^{1}H NMR (400 MHz, CD_3OD)

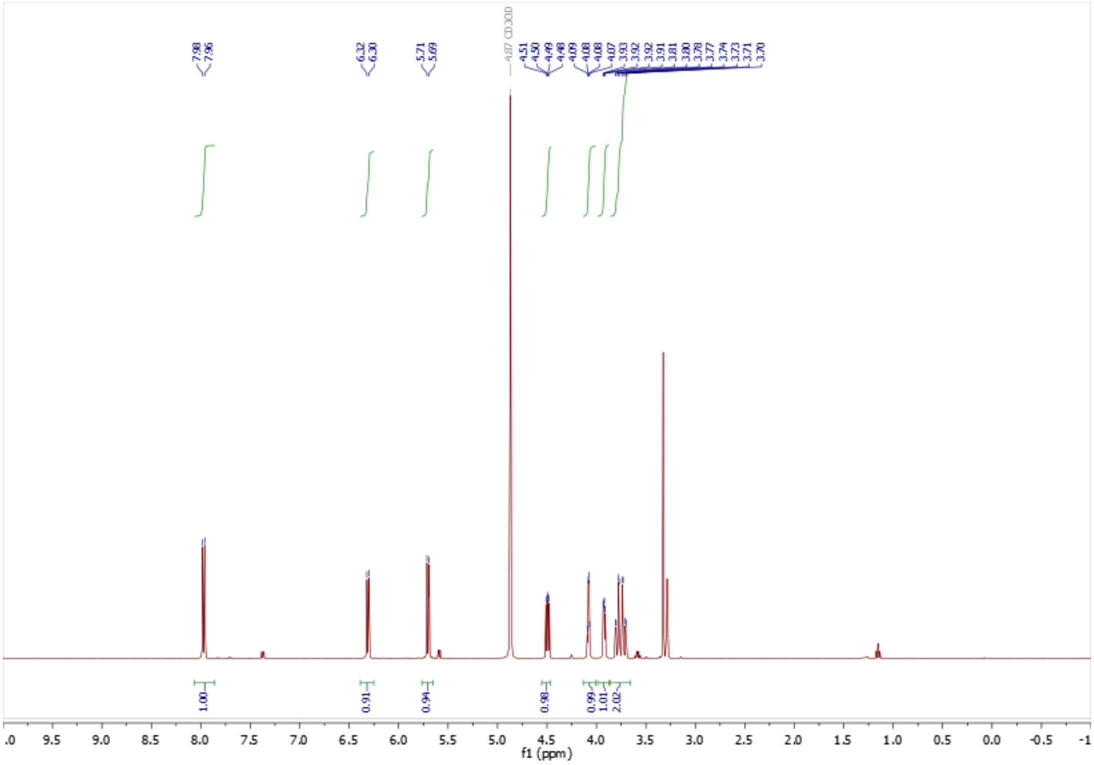

^{13}C NMR (101 MHz, CD_3OD)

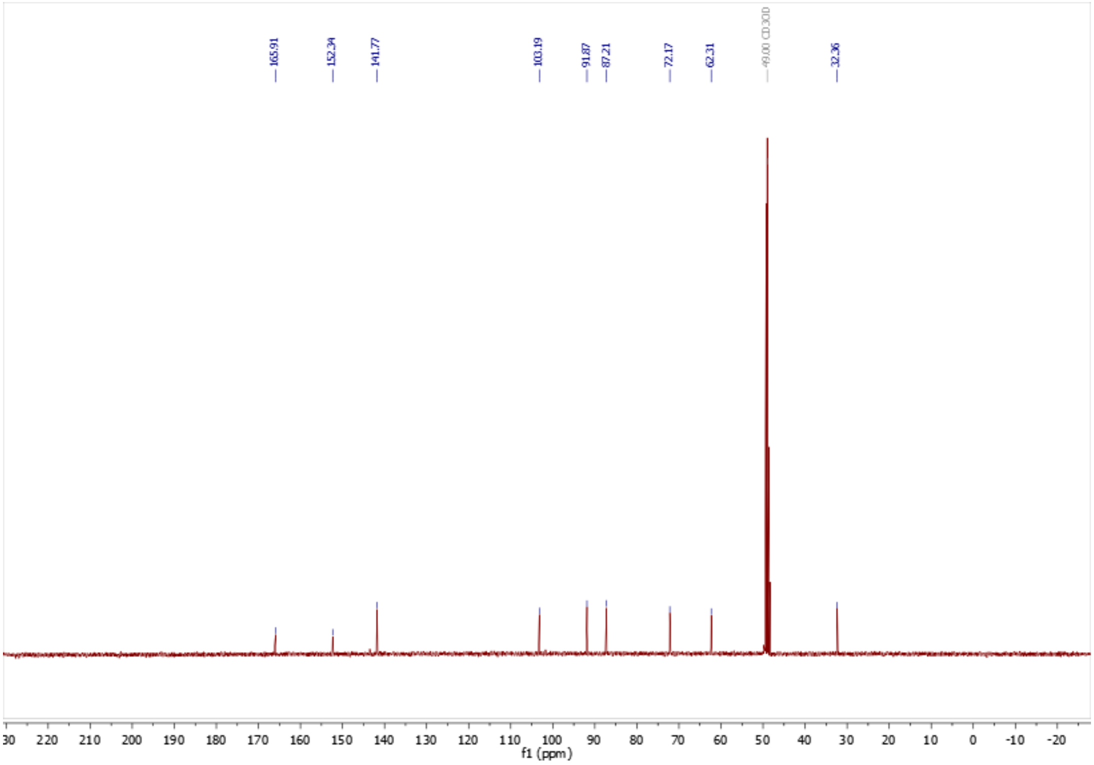

3′-5′-*O*-di(*tert*-butyldimethylsilyl)-2′-iodo-2′-deoxyuridine, S5

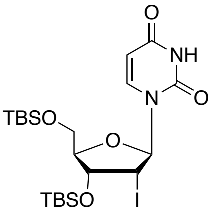

^{1}H NMR (400 MHz, $CDCl_3$)

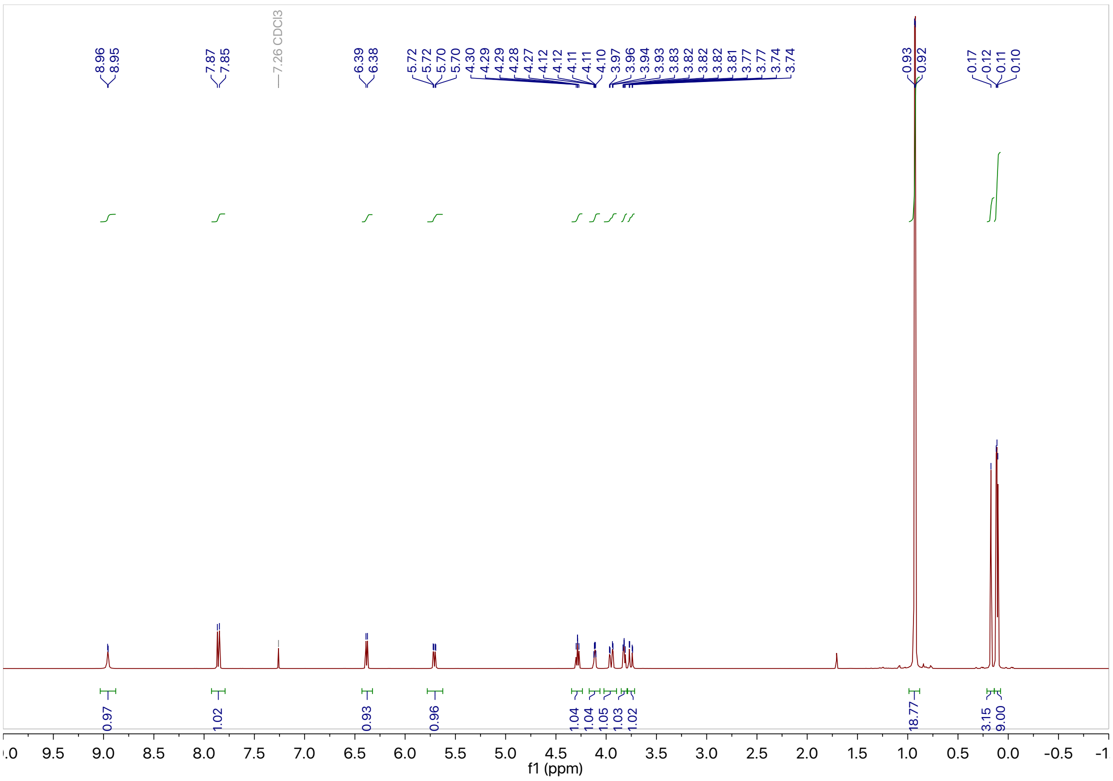

^{13}C NMR (101 MHz, $CDCl_3$)

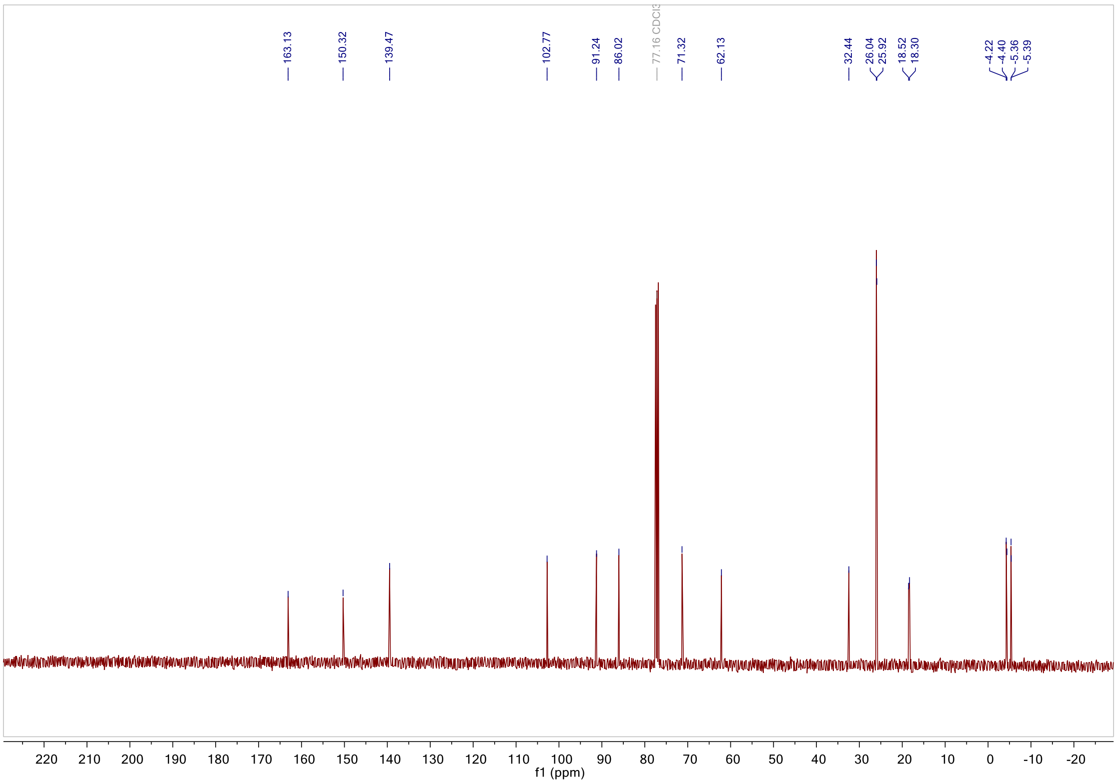

3′-*O*-(*tert*-butyldimethylsilyl)-2′-iodo-2′-deoxyuridine, 7w

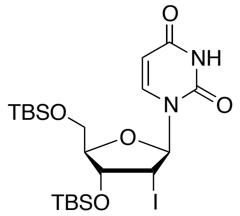

^{1}H NMR (400 MHz, MeOD)

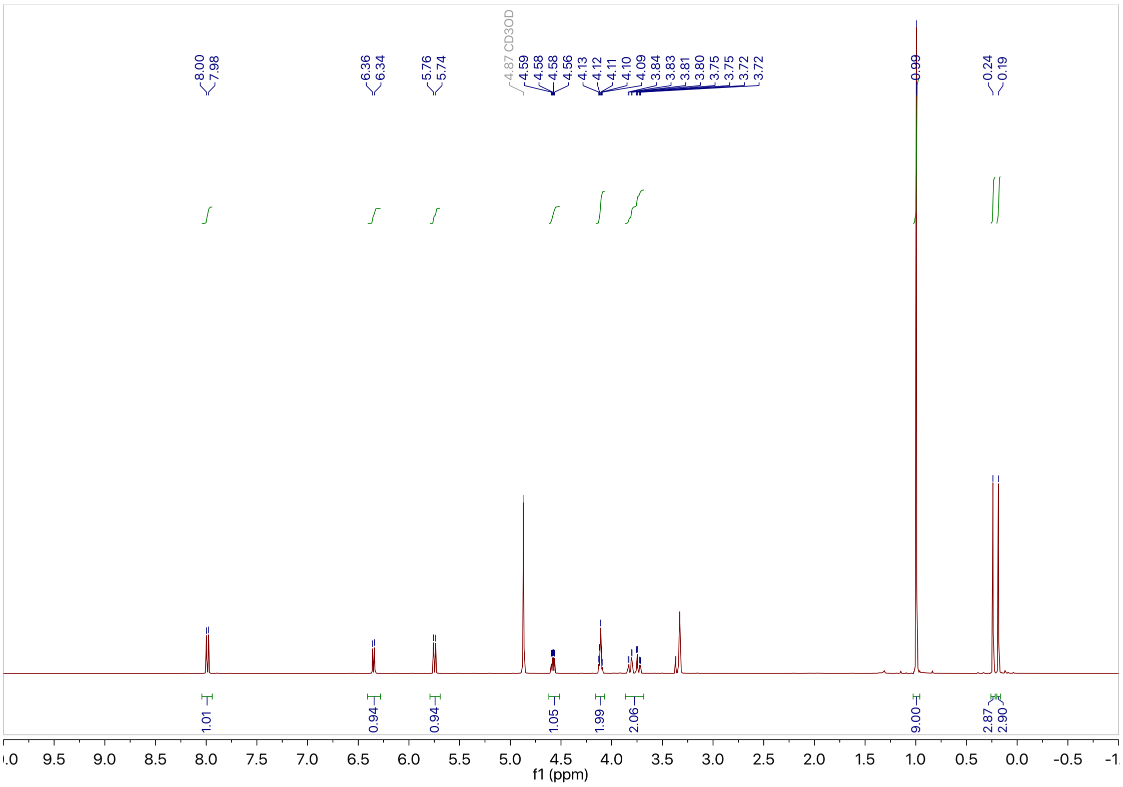

^{13}C NMR (101 MHz, MeOD)

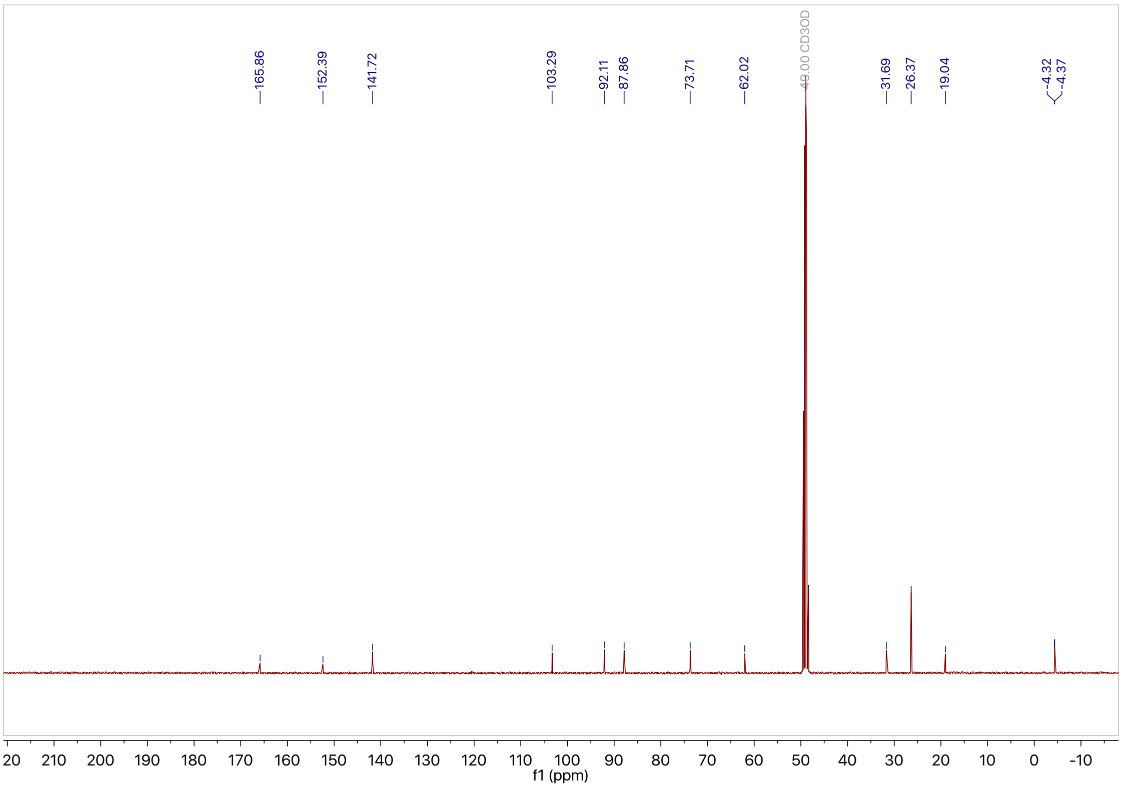

2-iodoethyl p-toluenesulfonate, 7y

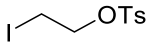

^{1}**H NMR** (400 MHz, $CDCl_3$)

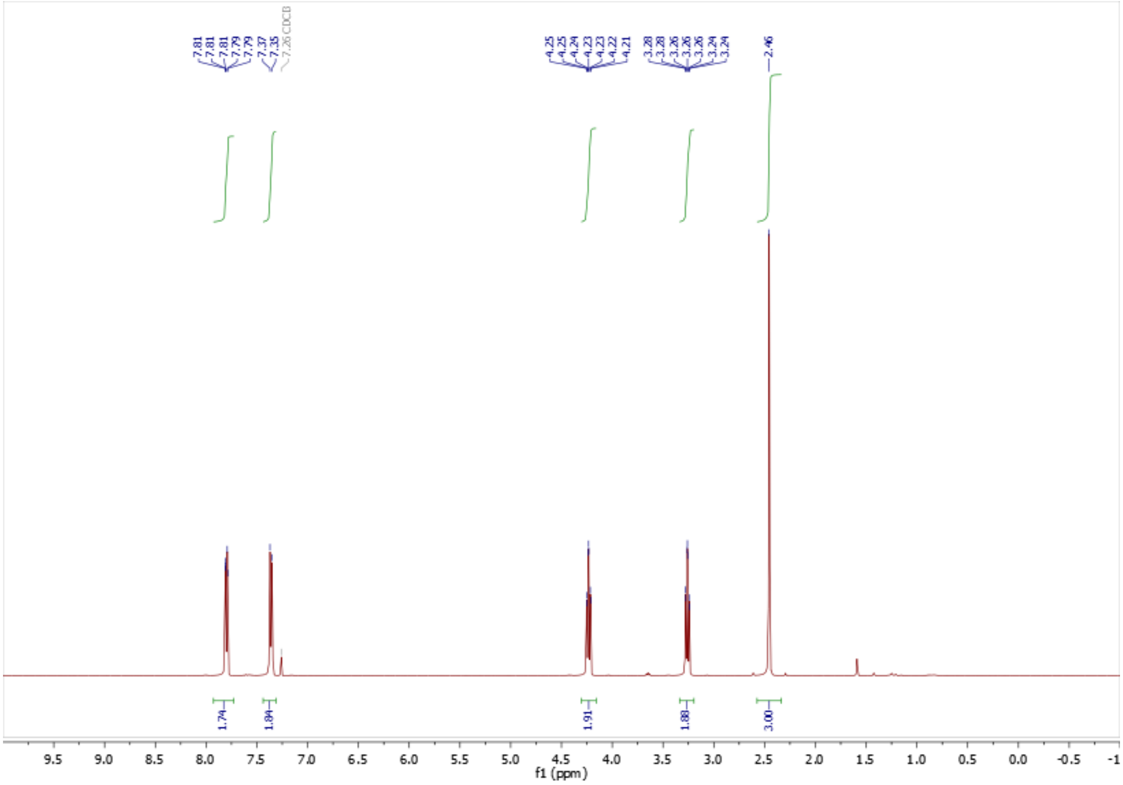

^{13}**C NMR** (101 MHz, $CDCl_3$)

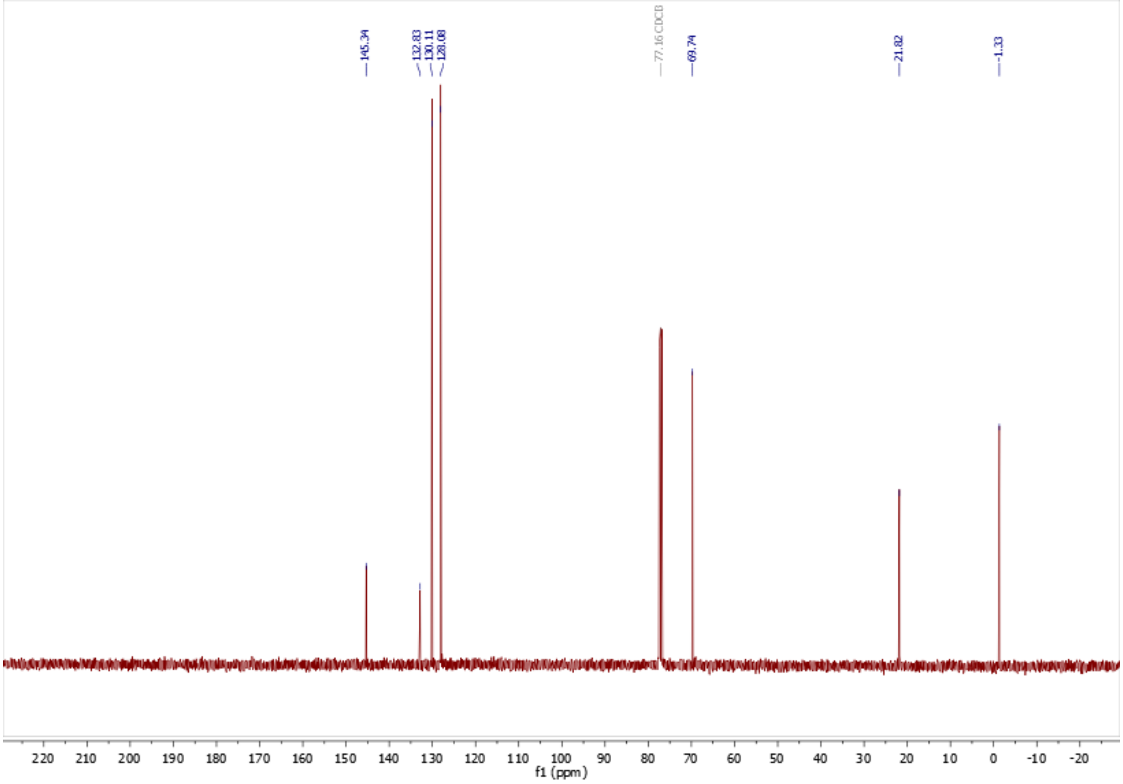

Boc-Asp(O-*t*Bu)-Ser-OMe, 15

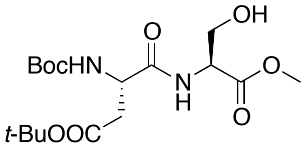

^{1}H NMR (400 MHz, $CDCl_3$)

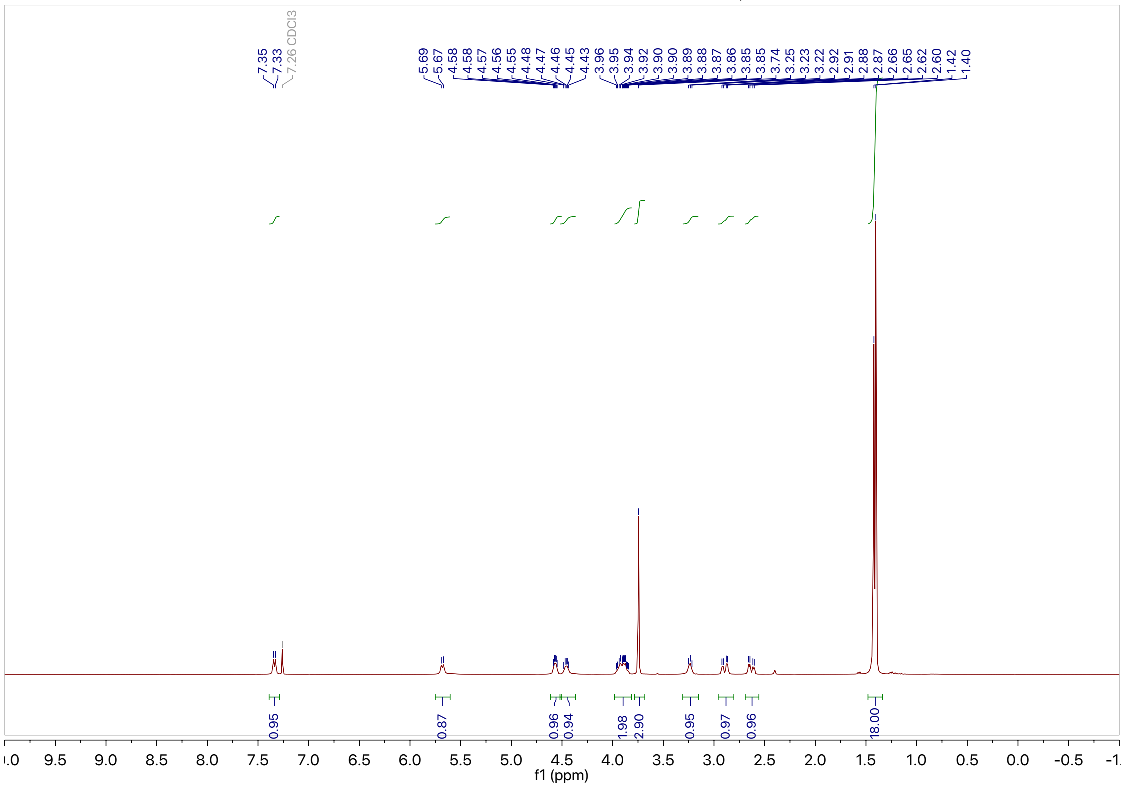

^{13}C NMR (101 MHz, $CDCl_3$)

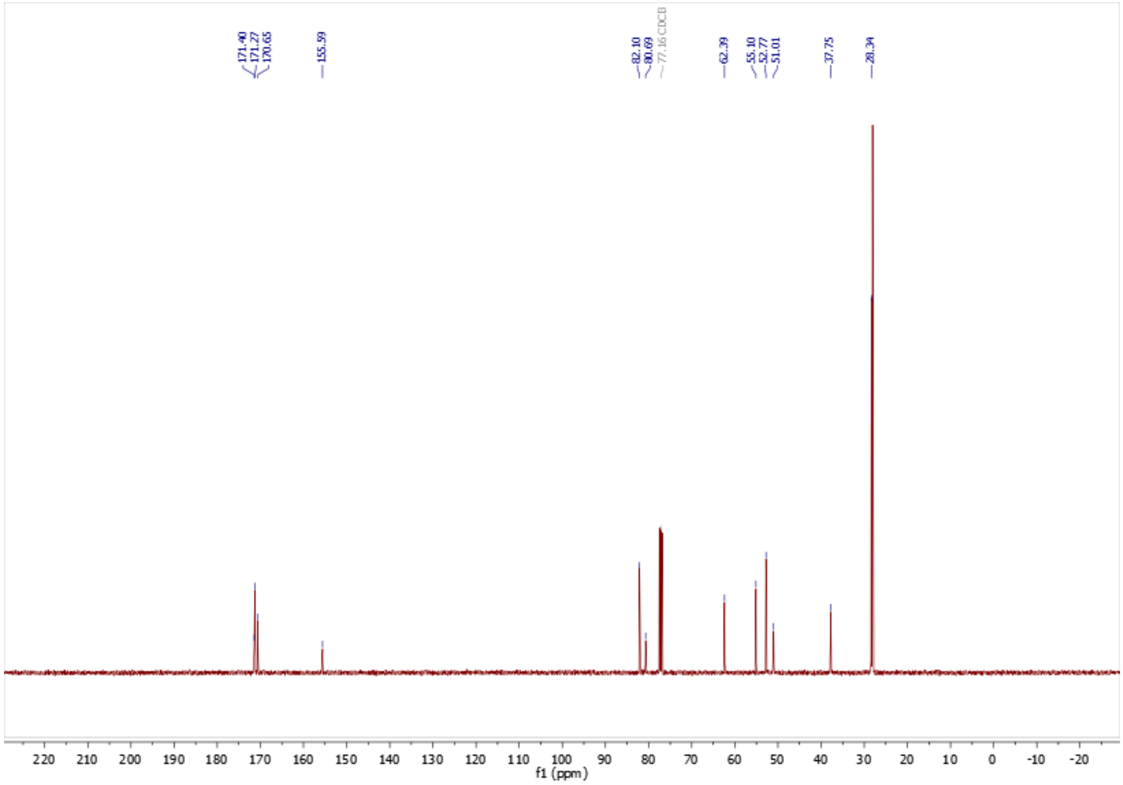

1-benzyl-*N*-phenylpiperidin-4-amine, S6

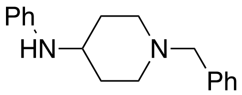

^{1}H NMR (400 MHz, $CDCl_3$)

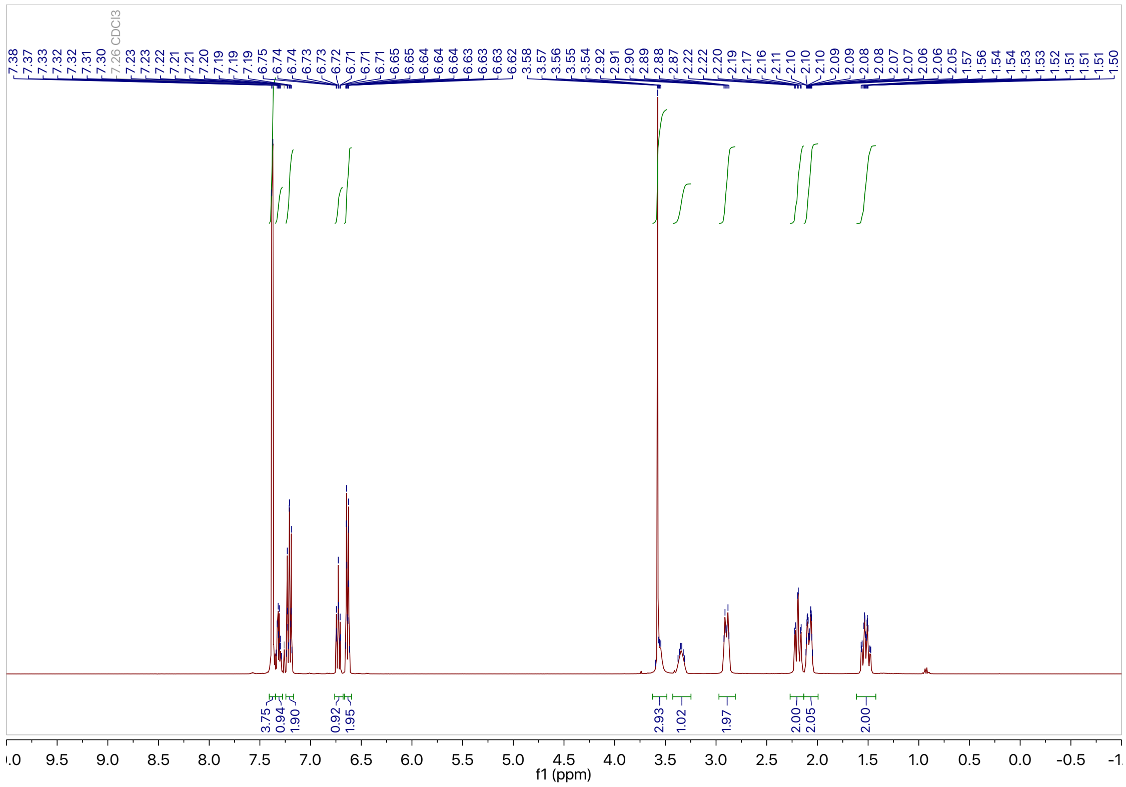

^{13}C NMR (101 MHz, $CDCl_3$)

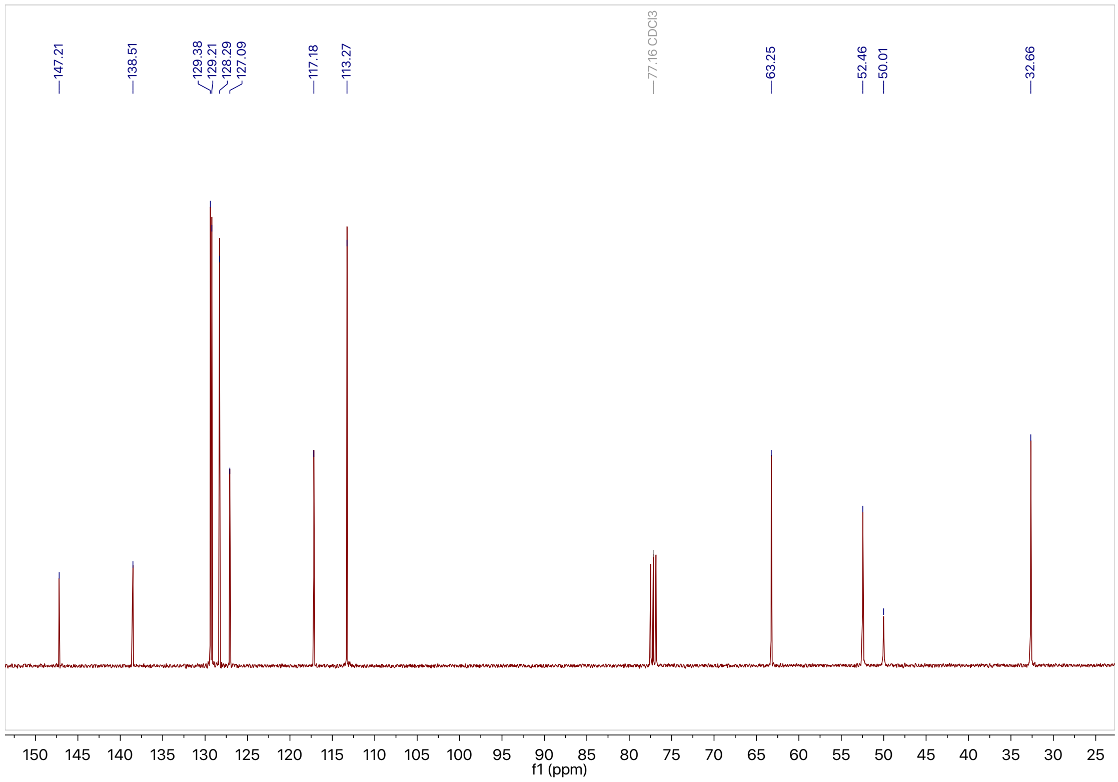

***N*-(1-benzylpiperidin-4-yl)-*N*-phenylpropionamide, S7**

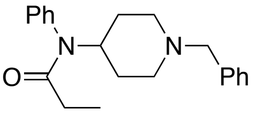

^{1}H NMR (400 MHz, $CDCl_3$)

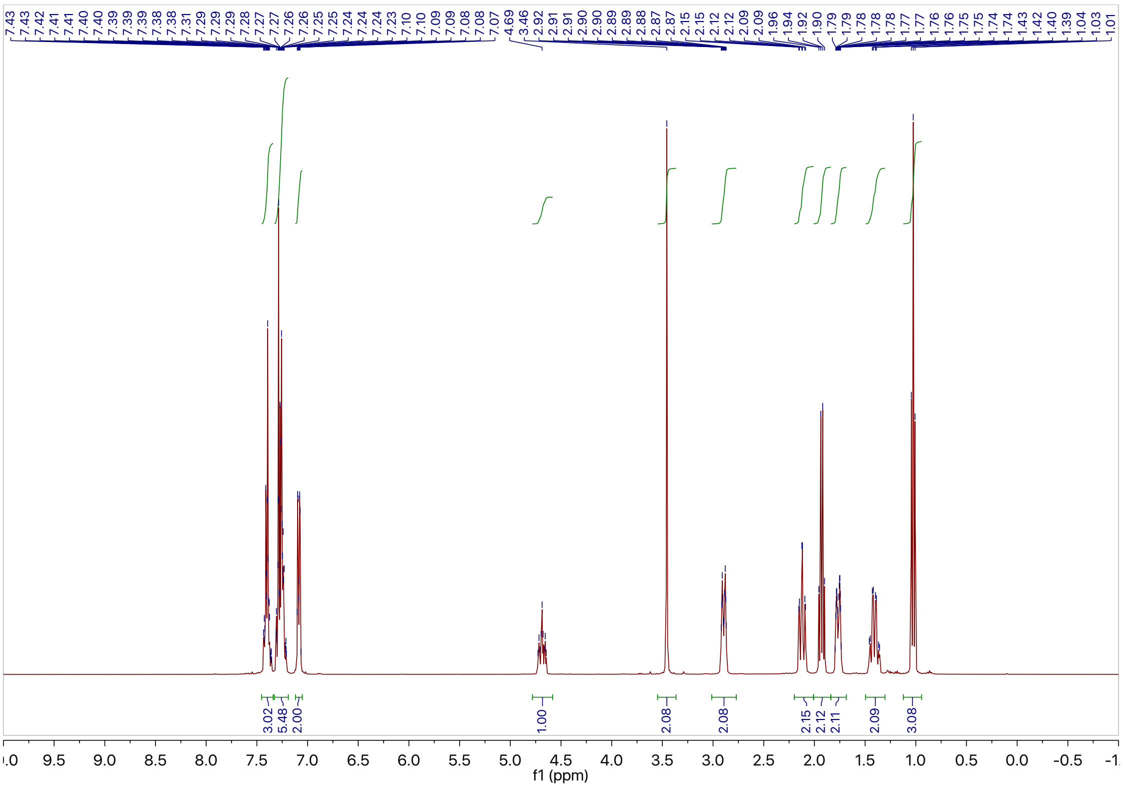

^{13}C NMR (101 MHz, $CDCl_3$)

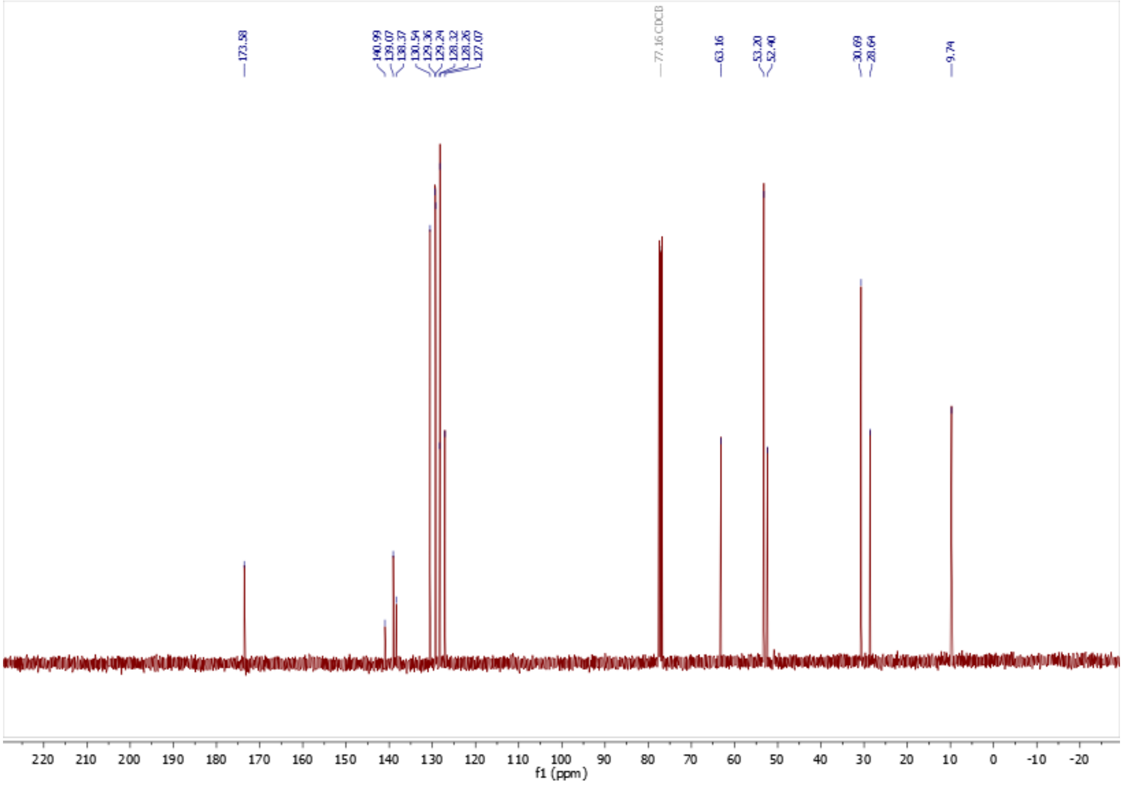

N-phenyl-*N*-(piperidin-4-yl)propionamide, 17

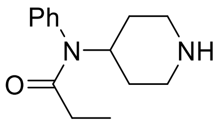

^{1}H NMR (400 MHz, CD_3OD)

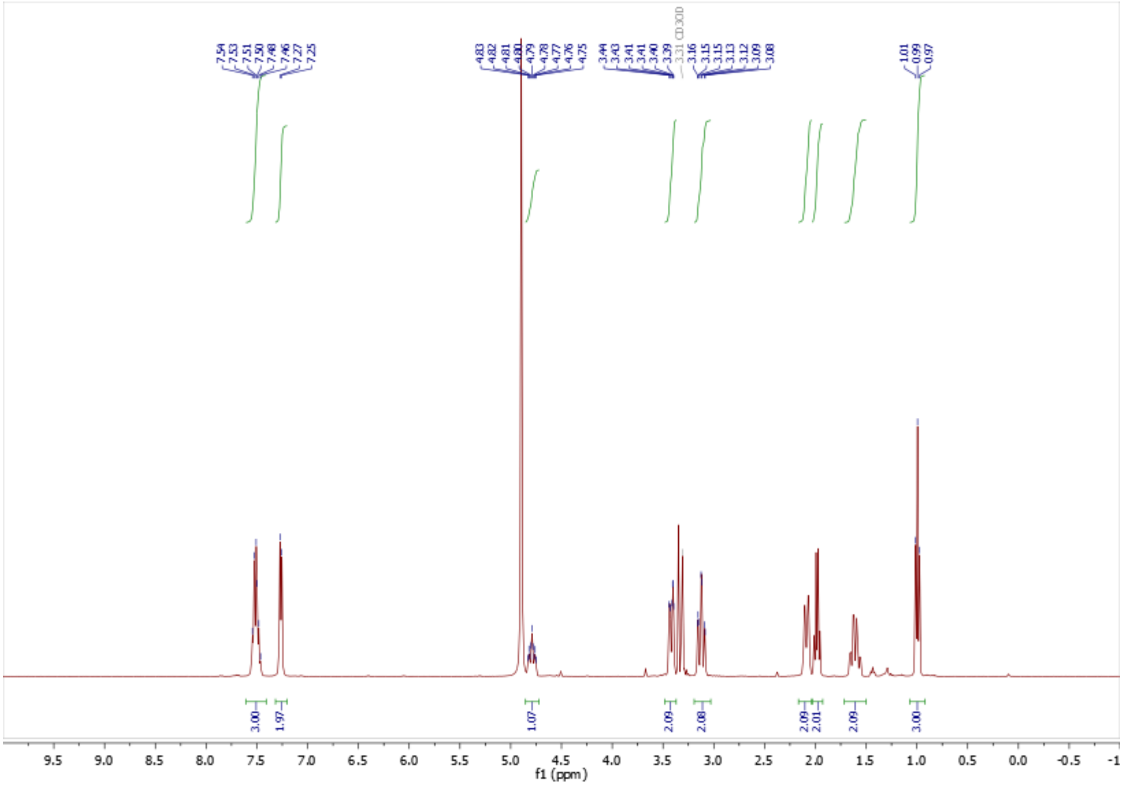

^{13}C NMR (101 MHz, CD_3OD)

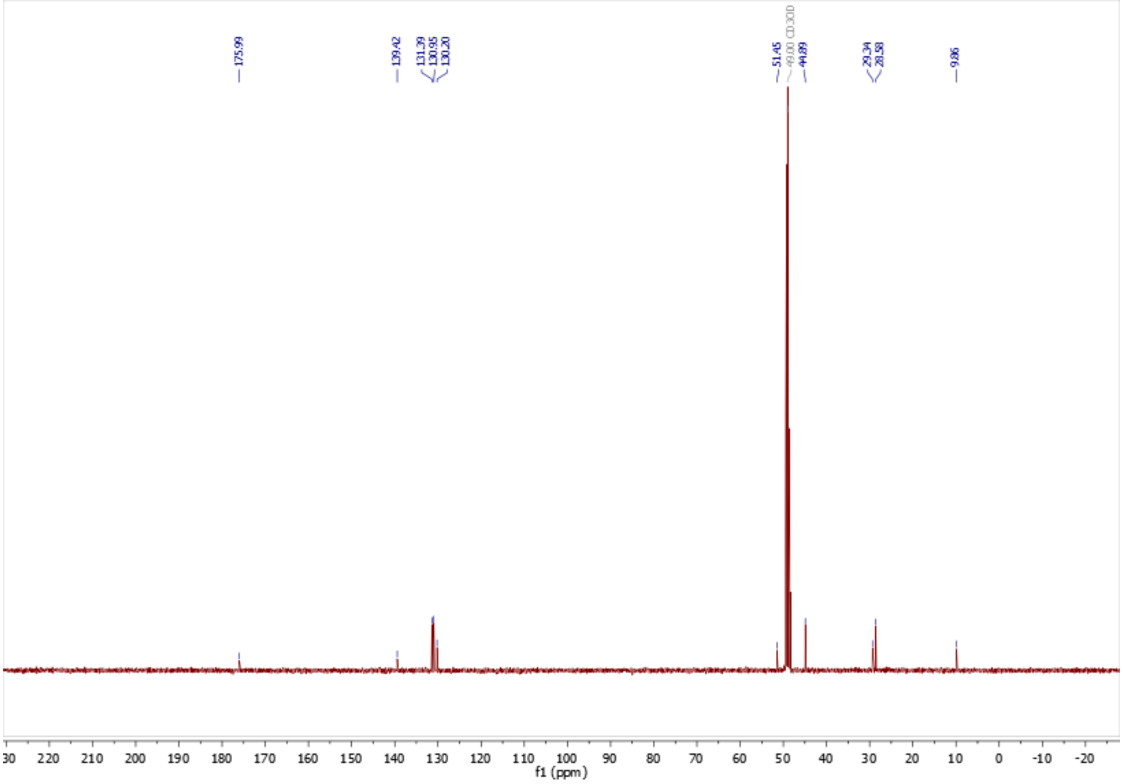

Supplement: Supplementary file 1 [file SC-009-C8SC01355A-s001.pdf]
